# Dual Inhibitors of P-gp and Carbonic Anhydrase XII (hCA XII) against Tumor Multidrug Resistance with Piperazine Scaffold †

**DOI:** 10.3390/molecules29143290

**Published:** 2024-07-11

**Authors:** Laura Braconi, Chiara Riganti, Astrid Parenti, Marta Cecchi, Alessio Nocentini, Gianluca Bartolucci, Marta Menicatti, Marialessandra Contino, Nicola Antonio Colabufo, Dina Manetti, Maria Novella Romanelli, Claudiu T. Supuran, Elisabetta Teodori

**Affiliations:** 1 Section of Pharmaceutical and Nutraceutical Sciences, Department of Neuroscience, Psychology, Drug Research and Child Health, University of Florence, via Ugo Schiff 6, 50019 Sesto Fiorentino, Italy; alessio.nocentini@unifi.it (A.N.); gianluca.bartolucci@unifi.it (G.B.); marta.menicatti@unifi.it (M.M.); dina.manetti@unifi.it (D.M.); novella.romanelli@unifi.it (M.N.R.); claudiu.supuran@unifi.it (C.T.S.); 2 Oncological Pharmacology Laboratory and Molecular Biotechnology Center “Guido Tarone”, Department of Oncology, University of Turin, Piazza Nizza 44, 10126 Torino, Italy; chiara.riganti@unito.it; 3 Section of Clinical Pharmacology and Oncology, Department of Health Sciences, University of Florence, Viale Pieraccini 6, 50139 Firenze, Italy; astrid.parenti@unifi.it; 4 Section of Pharmacology and Toxicology, Department of Neuroscience, Psychology, Drug Research and Child Health, University of Florence, Viale Pieraccini 6, 50139 Firenze, Italy; marta.cecchi@unifi.it; 5 Department of Pharmacy-Drug Sciences, University of Bari “A. Moro”, via Orabona 4, 70125 Bari, Italy; marialessandra.contino@uniba.it (M.C.); nicolaantonio.colabufo@uniba.it (N.A.C.)

**Keywords:** MDR reversers, P-gp modulators, CA XII inhibitors, K562/DOX, HT29/DOX, A549/DOX, hybrid compounds, multitarget ligands, dual P-gp/CA XII inhibitory activity, selective chemosensitizers

## Abstract

A new series of piperazine derivatives were synthesized and studied with the aim of obtaining dual inhibitors of P-glycoprotein (P-gp) and carbonic anhydrase XII (hCA XII) to synergistically overcome the P-gp-mediated multidrug resistance (MDR) in cancer cells expressing the two proteins, P-gp and hCA XII. Indeed, these hybrid compounds contain both P-gp and hCA XII binding groups on the two nitrogen atoms of the heterocyclic ring. All compounds showed good inhibitory activity on each protein (P-gp and hCA XII) studied individually, and many of them showed a synergistic effect in the resistant HT29/DOX and A549/DOX cell lines which overexpress both the target proteins. In particular, compound **33** displayed the best activity by enhancing the cytotoxicity and intracellular accumulation of doxorubicin in HT29/DOX and A549/DOX cells, thus resulting as promising P-gp-mediated MDR reverser with a synergistic mechanism. Furthermore, compounds **13**, **27** and **32** induced collateral sensitivity (CS) in MDR cells, as they were more cytotoxic in resistant cells than in the sensitive ones; their CS mechanisms were extensively investigated.

## 1. Introduction

Multidrug resistance (MDR) is a type of acquired resistance displayed by cancer cells that show reduced sensitivity to structurally and mechanistically unrelated anticancer drugs [1]. This phenomenon is one of the main problems in the chemotherapy of malignant tumors. MDR is mainly due to the overexpression on the cell membrane of some ATP binding cassette (ABC) proteins such as P-glycoprotein (P-gp), Multidrug-Resistance-associated Protein-1 (MRP1), and Breast Cancer Resistance Protein (BCRP). These transporter proteins work as efflux pumps, reducing the intracellular concentration of anticancer drugs below their active dose lowering their therapeutic efficacy [2].

P-glycoprotein (P-gp) is the most studied ABC transporter in clinical research; it is overexpressed in many blood and solid tumors causing reduced chemotherapeutic responses [3]. Due to the extensive involvement of P-gp in MDR, the co-administration of efficient P-gp inhibitors with chemotherapeutic agents has been proposed as a powerful therapeutic strategy to overcome MDR [4]. Over the years, many P-gp inhibitors have been identified which have been classified into three generations according to their chronology and characteristics [5,6,7]. Some of these compounds have reached clinical evaluation; however, in most cases, the results obtained were disappointing due to unexpected adverse effects and toxicity, which led to the early conclusion of many trials [8,9]. As a result, currently, no compounds have been approved for clinical use. Therefore, there is still an urgent need to find potent and effective MDR reversal agents with minimal adverse effects.

One of the causes of the toxicity of P-gp inhibitors is the presence of this transporter in many healthy tissues where it is responsible for several physiological and pharmacological effects [10,11], and the ability of P-gp inhibitors to affect the pharmacokinetics of co-administered chemotherapeutic agents [12]. A strategy to improve the therapeutic efficacy of drugs may involve the development of molecules that simultaneously inhibit two drug targets, thus exploiting synergistic therapeutic effects.

It has recently been reported that P-gp is co-localized and physically associated with the XII isoform of human carbonic anhydrase (hCA XII) on the membrane of several resistant tumor cells. The hCA XII isoform belongs to the family of carbonic anhydrases (CAs, EC 4.1.1.1) which are metalloenzymes that catalyze the conversion of carbon dioxide to bicarbonate and a proton, playing crucial functions related to pH regulation, homeostasis, and metabolism, and their inhibition leads to pharmacological responses [13].

hCA XII is a tumor-associated enzyme overexpressed in many solid and hypoxic tumors that is associated with their progression and metastasis formation [14,15,16]. It maintains an alkaline intracellular pH and an extracellular acidosis that favors the growth of tumor cells, compromising that of normal cells [17,18]. The intracellular alkalinization maintained by hCA XII is optimal for P-gp efflux activity; thus, the high expression of hCA XII in some chemoresistant P-gp-positive tumor cells contributes to MDR [19]. Therefore, inhibition of hCA XII causes a decrease in intracellular pH which reduces the ATPase activity of P-gp and consequently the efflux activity of the transporter [13,19].

Based on these observations, it is possible to develop a strategy that allows selective targeting of the efflux effect of P-gp in resistant tumor cells overexpressing both P-gp and hCA XII. Therefore, dual inhibitors of P-gp and hCA XII may serve as useful chemosensitizers to overcome P-gp-mediated MDR in tumor cells.

**Figure 1:**
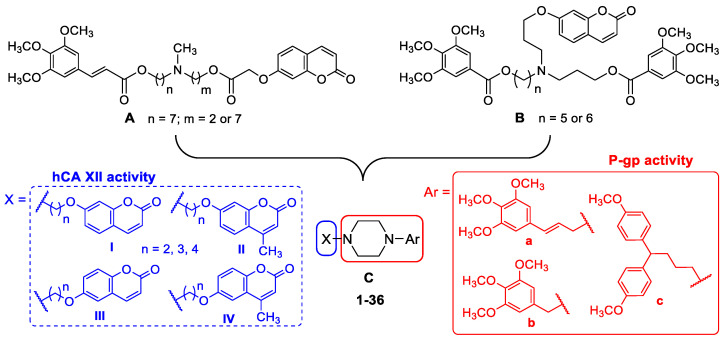
Structures of the leads (**A**,**B**) and general structure of piperazine derivatives **1**–**36**, synthesized in this study (**C**). For the structure of final compounds **1**–**36** see Table 1.

In previous papers [19,20], we reported the design and synthesis of a new series of *N*,*N*-bis(alkanol)amine aryl diesters able to modulate the activity of P-gp and hCA XII in cancer cells that overexpress both proteins (Figure 1, structures **A** and **B** of the most potent compounds of the series). These derivatives are characterized by the presence of both P-gp and hCA XII-binding groups to achieve a synergistic action to overcome the resistance. All compounds showed a multitarget effect being able to modulate the activity of both P-gp and hCA XII taken individually. Moreover, many of these hybrid derivatives showed a synergistic effect in cell lines that overexpress both proteins (LoVo/DOX, HT29/DOX, and A549/DOX cell lines), enhancing the cytotoxicity effect of doxorubicin in these resistant cells. These compounds can be considered promising chemosensitizer agents for selective inhibition in MDR cancer cells overexpressing both P-gp and hCA XII proteins.

Based on these encouraging results, we decided to continue our project on dual P-gp/hCA XII inhibitors with the aim of better investigating their structure–activity relationships. For this purpose, we synthesized new compounds containing a scaffold present in several MDR reversers [12,21], i.e., the piperazine ring, bearing arylalkyl and aryloxyalkyl substituents on the two nitrogen atoms. Therefore, three different methoxy-substituted arylalkyl moieties, such as the (*E*)-3-(3,4,5-trimethoxyphenyl)vinyl (**a**), 3,4,5-trimethoxyphenyl (**b**) or the 4,4-bis(4-methoxyphenyl)butyl (**c**) ones, which conferred good inhibitory effects on P-gp [21] were inserted on a nitrogen atom, leading to compounds **1**–**36** (Figure 1, structure **C**). Moreover, to maintain a selective activity towards hCA XII, we introduced a coumarin group through a 2-, 3- or 4-methylene chain on the second nitrogen atom of the piperazine scaffold by an ethereal bond. Especially, in addition to the 7-hydroxy-2*H*-chromen-2-one (**I**) used in the previous series [19,20], in these new compounds we introduced other different coumarins as 7-hydroxy-4-methyl-2*H*-chromen-2-one (**II**) and 6-hydroxy-2*H*-chromen-2-one (**III**) which showed inhibitory activity on hCA XII [22,23]; moreover, 6-hydroxy-4-methyl-2*H*-chromen-2-one (**IV**) was chosen to evaluate the influence of a methyl group also in coumarin **III** (Figure 1, structure **C**).

The synthesized compounds **1**–**36**, as dihydrochlorides, were first tested to evaluate their inhibitory effects on P-gp and hCA XII proteins, taken individually. The P-gp modulating activity was measured by evaluating the doxorubicin cytotoxicity enhancement on K562/DOX cells that overexpress only P-gp [24]. The hCA inhibition activity was measured on four different hCA isoforms: the cytosolic hCA I and hCA II, and the tumor-associated transmembrane hCA IX and hCA XII isoforms, to evaluate their hCA selectivity profiles. Eight selected compounds were then tested in doxorubicin-resistant human adenocarcinoma colon cells (HT29/DOX) and in doxorubicin-resistant non-small cell lung cancer cells (A549/DOX), that overexpress both P-gp and hCA XII [19], to verify the synergistic effect on the MDR reversal activity due to the dual P-gp/hCA XII inhibition.

Moreover, three compounds were further investigated as collateral sensitivity (CS)-promoting agents since they were more cytotoxic in resistant HT29/DOX and A549/DOX cells than in the parental ones. Therefore, we evaluated their effects on the intracellular amount of ROS and peroxidized lipids, P-gp catalytic activity, and membrane fluidity in HT29/DOX and A549/DOX cells.

## 2. Results

### 2.1. Chemistry

**Scheme 1:**
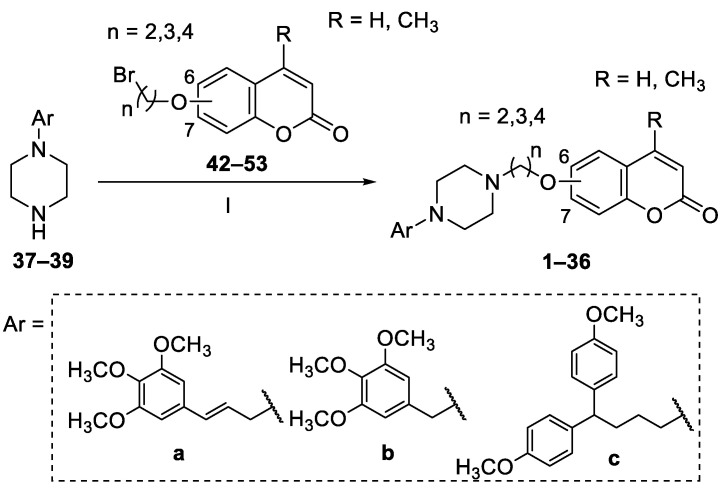
Reagents and conditions: (I) K_2_CO_3_, dry acetonitrile, 60 °C, overnight.

**Scheme 2:**
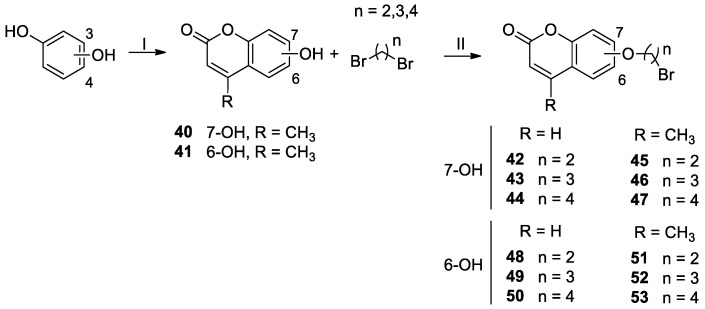
Reagents and conditions: (I) conc. H_2_SO_4_, ethyl acetoacetate, rt, 15 min or 4 days; (II) K_2_CO_3_, acetone or acetonitrile, reflux, overnight.

The reaction pathway used to obtain the piperazine derivatives **1**–**36** is described in Scheme 1. The proper methoxy-substituted arylalkyl piperazines (**37**–**39**) were synthesized as previously reported [21] and N-alkylated with the suitable bromoalkoxy-2H-chromen-2-ones (**42**–**53**, described in Scheme 2) in dry acetonitrile, yielding final compounds **1**–**36** with good yields.

The bromoalkoxy-2*H*-chromen-2-ones (**42**–**53**) were obtained by alkylation of the suitable hydroxy-2*H*-chromen-2-one with the proper dibromoalkane (1,2-dibromoethane, 1,3-dibromopropane or 1,4-dibromobutane) in acetone or acetonitrile, as reported in Scheme 2. Compound **43** has been previously reported [20], and **42**, **44**–**53** had been previously described [25,26,27,28,29], but in some cases obtained in different ways (see Section 4 for details). Compounds 7-hydroxy-2*H*-chromen-2-one and 6-hydroxy-2*H*-chromen-2-one are commercially available, while the previously described 4-methylated analogues (7-hydroxy-4-methyl-2*H*-chromen-2-one **40** [30] and 6-hydroxy-4-methyl-2*H*-chromen-2-one **41** [31]) were obtained following the procedure reported in [30], by condensation of resorcinol or hydroquinone, respectively, with ethyl acetoacetate under acidic conditions (Scheme 2).

### 2.2. CA Inhibitory Activity

The CA inhibitory activity of the new compounds **1**–**36**, evaluated by a stopped-flow CO_2_ hydrase assay [32], is reported in Table 1. Four hCA isoforms were used in this assay: the cytosolic hCA I and II and the tumor-associated transmembrane hCA IX and XII isoforms. Acetazolamide (AAZ) was used as a standard inhibitor.

The results confirm that the coumarin group directs activity only towards hCA IX and XII [33]. Indeed, all these derivatives inhibited both hCA IX and XII at nanomolar concentrations, while they were inactive against off-target hCA I and II isoforms.

The interaction with hCA IX and hCA XII isoforms seems to be influenced by the type of coumarin inserted on the piperazine nitrogen, indeed compounds **10**–**12**, **22**–**24**, **34**–**36**, bearing the 6-alkoxy-4-methyl-2*H*-chromen-2-one residue (**IV**), were the least potent on these two isoforms.

For compounds bearing the other coumarin groups, the activity was influenced by the methoxy-substituted arylalkyl moieties (**a**, **b**, or **c**). The most potent derivatives on the hCA IX and hCA XII isoforms carried the (*E*)-3-(3,4,5-trimethoxyphenyl)vinyl (**a**) and the 3,4,5-trimethoxyphenyl (**b**) groups (**1**–**9** and **13**–**21**, respectively), regardless of linker length which affects neither potency nor selectivity. Most compounds were more active towards hCA XII than hCA IX, except for **4** and **15**–**18**. Notably, derivatives **9**, **13**, and **19** showed the highest potency towards hCA XII, with K_i_ values < 10 nM (K_i_ = 7.3 nM, 6.2 nM and 5.8 nM, respectively), comparable to that of the reference compound AAZ (K_i_ = 5.7 nM). Derivative **9** has the (*E*)-3-(3,4,5-trimethoxyphenyl)vinyl residue (**a**) and 6-hydroxy-2*H*-chromen-2-one (**III**) linked by a 4-methylene chain, while **13** and **19** have the 3,4,5-trimethoxyphenyl group (**b**) and 7-hydroxy-2*H*-chromen-2-one (**I**) or 6-hydroxy-2*H*-chromen-2-one (**III**), respectively, connected by a 2-methylene chain.

### 2.3. Doxorubicin Cytotoxicity Enhancement Assay in K562/DOX Cells

The P-gp modulating activity of compounds **1**–**36** was measured by evaluating the cytotoxicity enhancement of the co-administered doxorubicin in K562/DOX cells. K562 is a highly undifferentiated erythroleukemia cell line [34]. The doxorubicin-resistant K562/DOX cells overexpress almost exclusively the transporter membrane protein P-gp [24]. Since doxorubicin is a P-gp substrate and is expelled out of the cell by the pump, it is generally inactive in tumor cells that express P-gp.

Compounds were first studied at 1 and 3 µM concentrations to evaluate their intrinsic cytotoxicity in both the parental K562 and the resistant K562/DOX cell lines, using the MTT assay [35]. All compounds had no intrinsic cytotoxicity in the parental line. Most of them showed a toxicity of approximately 20% in the resistant cells at the two concentrations tested, while compounds **7**, **9**, **25**, and **27** reduced the cellular viability by 30–50% at 3 µM (Supplementary Materials, Figure S1).

Compounds **1**–**36** were studied at 1 and 3 µM concentrations to evaluate their ability to decrease the IC_50_ value of co-administered doxorubicin in K562/DOX cells. The results are reported in Table 1 as RF (Reversal Fold) values that are the ratio between the IC_50_ value of doxorubicin alone and in the presence of the studied compounds: the higher the RF value, the higher the MDR reversal activity. Verapamil (Ver) was used as a reference compound.

All these derivatives enhanced the cytotoxicity of doxorubicin to different extents, except compound **7,** which shows RF = 1 for both concentrations, and most of them showed higher RF values than those of verapamil.

Results indicated that compounds with the 3,4,5-trimethoxyphenyl (**b**) group (**13**–**24**) were less potent both at 1 and 3 µM concentrations. Almost all compounds with the (*E*)-3-(3,4,5-trimethoxyphenyl)vinyl (**a**) residue and 7-(alkoxy)-2*H*-chromen-2-one (**I**) or 7-(alkoxy)-4-methyl-2*H*-chromen-2-one (**II**) (**2**, **3** and **4**–**6**, respectively) showed higher RF values than those of verapamil at both the tested concentrations, regardless of the linker length. All compounds with the 4,4-bis(4-methoxyphenyl)butyl (**c**) residue were the most potent compounds; in particular, compounds **28**, **29**, **35** and **36** showed RF values higher than 10.0 when used at 1 µM, and compounds **25**, **27**–**30**, **33** and **36** showed RF values higher than 20.0 when used at 3 µM.

A thorough analysis of the obtained results seems to indicate the absence of a correlation between the structural requirements necessary to modulate the efflux activity of P-gp and those to inhibit hCA XII.

### 2.4. Doxorubicin Cytotoxicity Enhancement Assay in HT29/DOX and A549/DOX Cells

Compounds **1**–**36** were also tested in doxorubicin-resistant human adenocarcinoma colon cells (HT29/DOX) and in doxorubicin-resistant non-small cell lung cancer cells (A549/DOX), that overexpress both P-gp and hCA XII [19], to study the effect of these dual P-gp/hCA XII inhibitors in a specific environment where the two target proteins coexist.

The expression levels of P-gp and hCA XII in sensitive HT29 and A549 cells and their resistant counterparts (HT29/DOX and A549/DOX cells) were checked by immunoblotting analysis, as previously reported [20]. The resistant sublines also showed increased expression of MRP1, another transporter involved in doxorubicin resistance, which, however, was not associated with hCA XII nor was affected in its efflux activity by hCA XII [19,20].

All compounds were preliminarily studied to evaluate their intrinsic cytotoxicity at different concentrations (from 10 nM to 50 µM) in the parental HT29 and A549 and the resistant HT29/DOX and A549/DOX cells using the MTT assay [35].

Most compounds showed less than 35% toxicity in parental HT29 and A549 cells at 1 µM, except for **12**, **27**, **29**, **32**, **35**, and **36** (Supplementary Materials, Figures S2 and S3). On the resistant counterparts (HT29/DOX and A549/DOX cells), compounds showed a toxicity of less than 35% with the exception of **8**, **12**, **15**, **25**, **27**, **29**, **32**, **35**, and **36** (Supplementary Materials, Figures S4 and S5).

Based on the results obtained in the intrinsic cytotoxicity assays, the compounds with the best profiles in terms of potency on P-gp in the K562/DOX cells test and selectivities towards the hCA XII isoform, **1**, **2**, **4**–**6**, **14**, **19** and **33**, were selected for further evaluation in HT29/DOX and A549/DOX cell lines in co-administration with doxorubicin.

**Figure 2:**
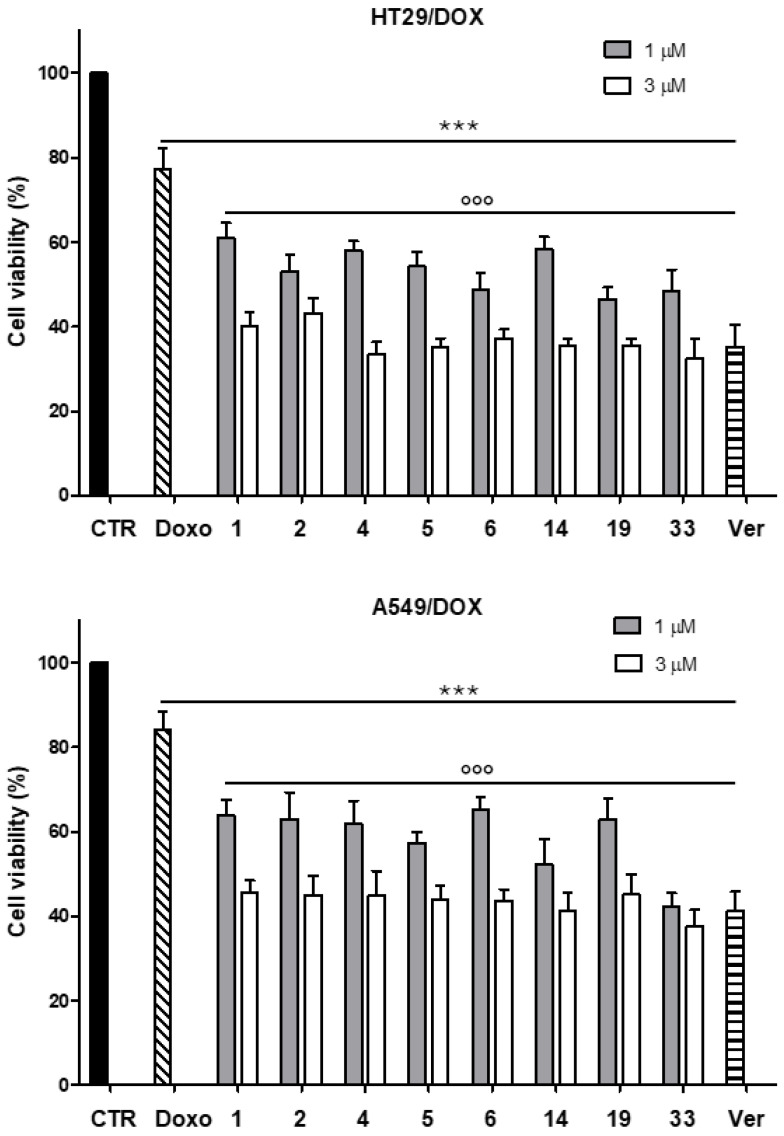
Antiproliferative activity in HT29/DOX (**top**) and A549/DOX (**bottom**) cells of doxorubicin (Doxo) at 5 μM, alone and in co-administration with selected derivatives at 1 μM and 3 μM, or verapamil (Ver) at 1 mM, measured after 48 h. Each bar represents the mean ± SD of three independent experiments, with technical triplicates. Control (CTR) is 100% cell growth. One-way analysis of variance (ANOVA) analysis: *** *p* < 0.0001 vs. control; °°° *p* < 0.0001 vs. doxorubicin alone.

These compounds were then assayed at 1 and 3 µM concentrations in co-administration with doxorubicin at a concentration of 5 µM, which is known to discriminate well between sensitive and resistant cells [36]. In HT29 and A549 cells, which express low levels of P-gp [20], the tested compounds did not significantly increase the cytotoxicity of doxorubicin used alone (Supplementary Materials, Figure S6). Otherwise, in the resistant counterparts (HT29/DOX and A549/DOX), which displayed high levels of P-gp and hCA XII, the selected compounds at both 1 and 3 µM concentrations enhanced the cytotoxicity of doxorubicin, measured as the percentage of cell growth, in a dose-dependent way (Figure 2). In general, compounds were more active in HT29/DOX than in A549/DOX cells at both concentrations. In particular, compounds **4**, **5**, **14**, and **19** at 3 µM concentration were the most potent in doxorubicin-treated HT29/DOX cells causing a reduction in cell viability by 65.0%. Moreover, in the A549/DOX cell line, the co-administration of **1**, **2**, **4**–**6**, **14** and **19**, tested at 3 µM, led to the highest enhancement of doxorubicin toxicity reaching a reduction of nearly 55.0% in cell viability. In both tested cell lines, the best compound was derivative **33**, which, at the highest concentration, in combination with the anticancer drug, caused a reduction in cell viability of 67.6% in HT29/DOX cells and 62.6% in A549/DOX cells. Interestingly, most compounds at 3 µM achieved the same potency as verapamil tested at 1 mM concentration.

### 2.5. Doxorubicin Accumulation Assay in HT29/DOX and A549/DOX Cells

Furthermore, we evaluated the intracellular accumulation of 5 µM doxorubicin in HT29/DOX and A549/DOX cells alone and in the presence of derivatives **1**, **2**, **4**–**6**, **14**, **19** and **33**, studied at 1 and 3 µM. This test allowed us to investigate if the reduced viability measured in the co-administration assay was due to different retention of the anticancer drug within the cells. In the parental HT29 and A549 cell lines, we measured a greater amount of intracellular doxorubicin compared to their resistant counterparts, which was not enhanced by our selected compounds (Supplementary Materials, Figure S7). However, in HT29/DOX and A549/DOX cells, derivatives **1**, **2**, **4**–**6**, **14**, **19** and **33** were able to increase the intracellular retention of the anticancer drug, in a dose-dependent way. Intracellular doxorubicin accumulation was lower in A549/DOX than in HT29/DOX cells probably due to a slightly greater basal expression of the transporter protein MRP1 [20], which can contribute to doxorubicin efflux [2].

**Figure 3:**
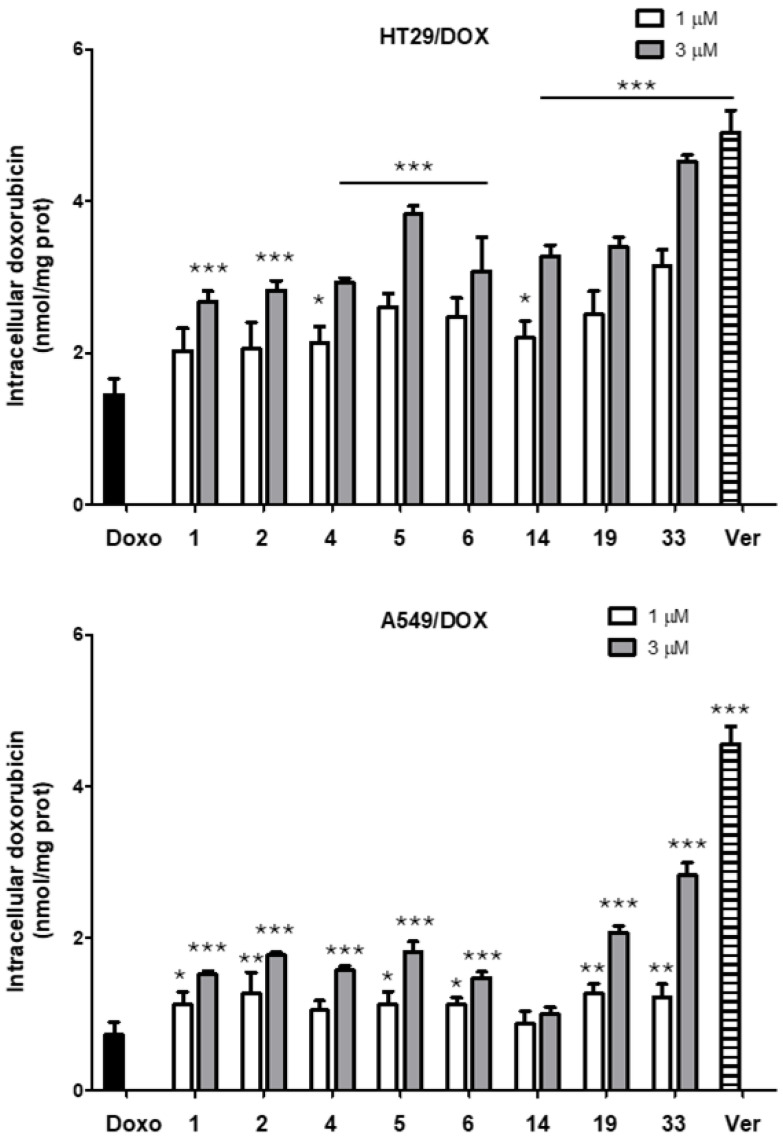
Intracellular accumulation of doxorubicin in HT29/DOX (**top**) and A549/DOX (**bottom**) cells, incubated 24 h with doxorubicin (Doxo) at 5 µM, alone and in co-administration with selected derivatives at 1 μM and 3 μM, or verapamil (Ver) at 1 mM. Each bar represents the mean ± SD of three independent experiments, with technical duplicates. One-way analysis of variance (ANOVA) analysis: * *p* < 0.05, ** *p* < 0.001, *** *p* < 0.0001 vs. doxorubicin alone.

The most potent molecules were **5**, **19**, and **33** in both cell lines (Figure 3). In particular, at the highest concentration, compound **33** allowed a 3- or 4-fold greater accumulation of doxorubicin in HT29/DOX or A549/DOX cells, respectively, showing a potency comparable to that of verapamil tested at 1 mM in HT29/DOX (Figure 3).

### 2.6. Collateral Sensitivity Studies

Recently, it emerged that P-gp-expressing cells are paradoxically more sensitive to specific compounds than their counterpart with no level of P-gp [37,38,39]. This phenomenon, called “collateral sensitivity” (CS), relies on different mechanisms, among which the increased generation of reactive oxygen species (ROS) that damage the structural cell components, including the plasma membrane lipid where P-gp is embedded, and the increased membrane fluidity [40,41,42].

**Figure 4:**
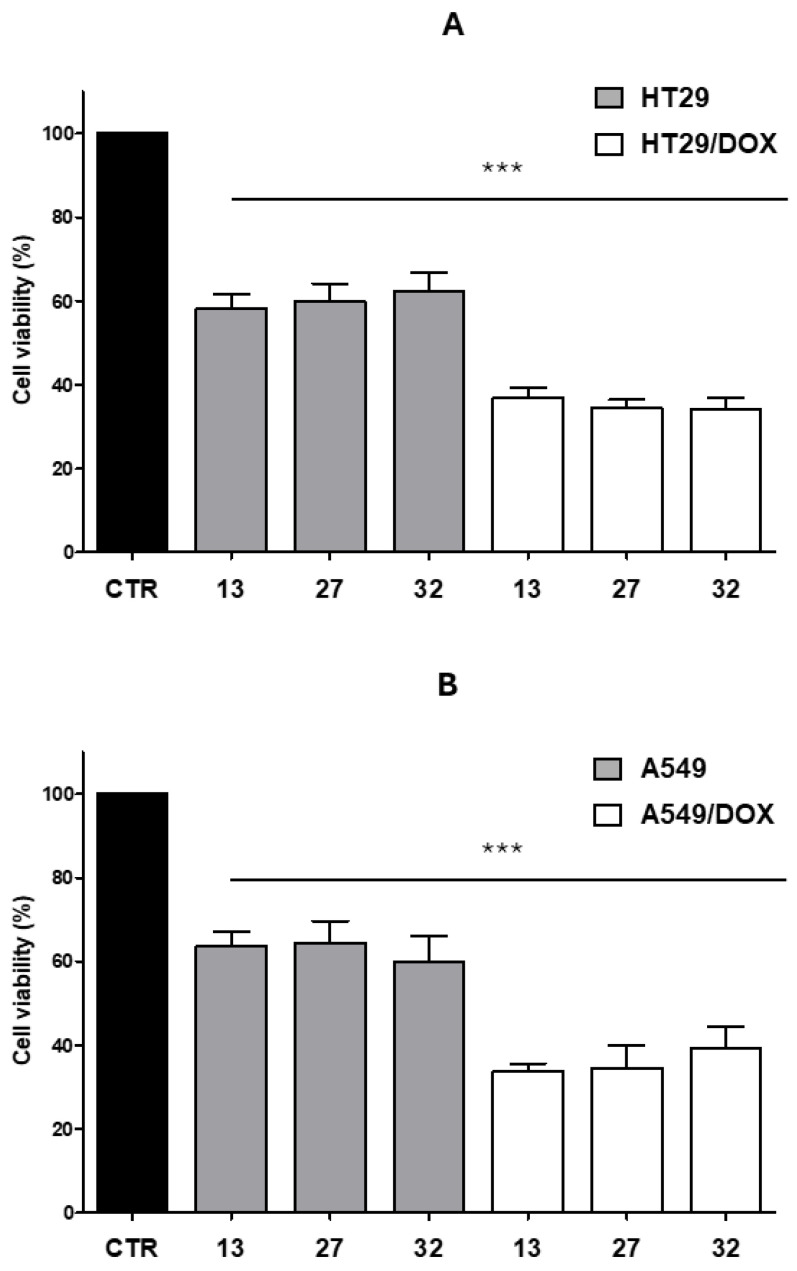
Antiproliferative activity in (**A**) HT29 or HT29/DOX cells and (**B**) A549 or A549/DOX cells of derivatives **13**, **27**, and **32** (1 μM) measured after 48 h. Each bar represents the mean ± SD of three independent experiments, with technical triplicates. Control (CTR) is 100% cell growth. One-way analysis of variance (ANOVA) analysis: *** *p* < 0.0001 vs. control.

Interestingly we noticed that compounds **13**, **27**, and **32** administered alone were 15%-20% more cytotoxic in the resistant HT29/DOX and A549/DOX cells than in the parental sensitive cells (Figure 4). We thus hypothesized that these compounds could be potential CS inducers.

**Figure 5:**
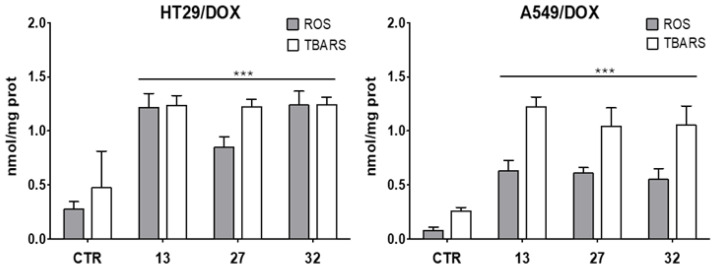
Effects on ROS or TBARS levels of **13**, **27** and **32** at 1 μM in HT29/DOX (**left**) and A549/DOX (**right**) cell lines. Each bar represents the mean ± SD of three independent experiments, with technical duplicates. Control (CTR) is ROS and TBARS levels in both HT29/DOX and A549/DOX cells. One-way analysis of variance (ANOVA) analysis: *** *p* < 0.0001 vs. control.

For this purpose, we tested the selected compounds in HT29/DOX and A549/DOX cells to investigate possible mechanisms through which they could work as potential CS inducers. Resistant cells often display a peculiar sensitivity to compounds able to increase ROS [43]. Interestingly, in both HT29/DOX and A549/DOX cells, compounds **13**, **27**, and **32** significantly enhanced the intracellular amount of ROS and peroxidized lipids, thiobarbituric reactive substances (TBARS), considered indexes of oxidative stress [44] (Figure 5). In particular, in HT29/DOX, they were able to increase the levels of ROS and TBARS by 3–4 and 2.6 times, respectively, compared to the control, while in A549/DOX cells, these compounds caused a major increase in both oxidative stress indexes (7–8 times for ROS, and 4 times for TBARS).

It is noteworthy that some coumarin-derivatives may act as anticancer agents thanks to their ability to inhibit thioredoxin reductase 1, eliciting oxidative stress [45,46]. This mechanism can explain the increase in ROS and TBARS as well as the cytotoxic potential of the compounds against chemoresistant cells that are even more susceptible to ROS than chemosensitive cells [42]. However, for the moment, this aspect has not yet been explored in depth for these new compounds.

**Figure 6:**
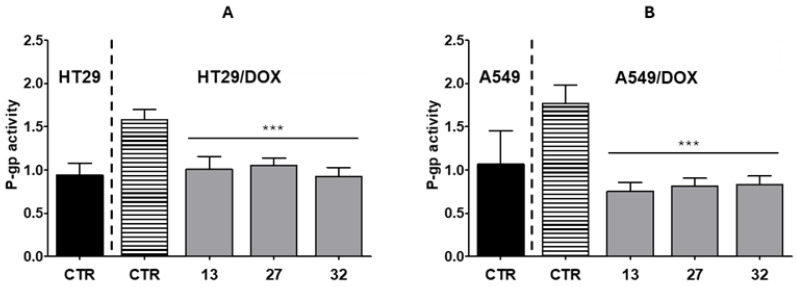
P-gp activity in (**A**) HT29 or HT29/DOX cells and (**B**) A549 or A549/DOX cells, in the absence (CTR) or presence of **13**, **27** and **32** at 1 μM. Each bar represents the mean ± SD of three independent experiments, with technical duplicates. One-way analysis of variance (ANOVA) analysis: *** *p* < 0.0001 vs. control in resistant cells (HT29/DOX or A549/DOX).

Oxidized lipids, besides triggering cell death, e.g., via ferroptosis [47], are important to modulate the activity of P-gp. Resistant cells usually have a lower amount of oxidizable fatty acid incorporated in the plasma membrane and this lipid composition creates an optimal environment for P-gp catalytic activity [48]. However, every factor increasing the amount of oxidized lipids alters the chemical–physical properties of the membrane impairing P-gp conformation, drug binding, and release [48]. A similar scenario also occurred in HT29/DOX and A549/DOX cell lines treated with compounds **13**, **27**, and **32,** which lowered the catalytic efficacy of P-gp in resistant cells to levels comparable to drug-sensitive HT29 and A549 cells (Figure 6).

**Figure 7:**
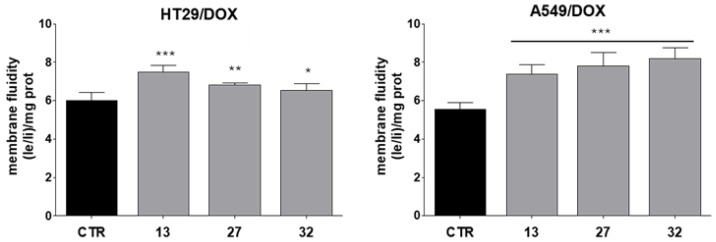
Membrane fluidity in HT29/DOX (**left**) and A549/DOX (**right**) cells, in the absence (CTR) or presence of **13**, **27** and **32** at 1 μM. Each bar represents the mean ± SD of three independent experiments, with technical duplicates. One-way analysis of variance (ANOVA) analysis: * *p* < 0.05, ** *p* < 0.001 *** *p* < 0.0001 vs. control.

Similarly, P-gp activity is high in plasma membrane rich in saturated fatty acid that allows a proper conformation of the transporter [48]. By contrast, a more fluid membrane reduces the catalytic activity of the transporter [49,50]. This happens, for instance, when the ratio between polyunsaturated and saturated fatty acid is increased [49] or when specific compounds act as membrane fluidifiers [42]. This seems also to be the case of compounds **13**, **27** and **32**, which increased membrane fluidity of HT29/DOX and A549/DOX cells (Figure 7), displaying an additional mechanism by which they can act as CS inducers and by which they reduce the activity of P-gp.

### 2.7. Transport Inhibition of Fluorescent Probes in MDCK Transfected Cells

The derivatives studied as collateral sensitizers, **13**, **27** and **32**, together with compound **33**, which displayed the highest potency in doxorubicin co-administration assays, were further tested in three Madin–Darby Canine Kidney (MDCK) transfected cell lines that overexpress P-gp, MRP1 or BCRP.

The MDR interaction potency of these compounds was studied by detecting the inhibition of the P-gp-, MRP1- or BCRP-mediated efflux of a substrate in cells overexpressing the studied pumps. In detail, we measured the following: (i) the transport inhibition of the profluorescent probe calcein-AM (P-gp and MRP1 substrate) in MDCK-MDR1 and MDCK-MRP1 cells (P-gp- and MRP1-overexpressing cells, respectively); (ii) the transport inhibition of the fluorescent probe Hoechst 33342 (BCRP substrate) in MDCK-BCRP cells (BCRP-overexpressing cells) [51].

The results, reported in Table 2, showed that CS inducers **13**, **27**, and **32** modulated the P-gp-mediated transport of calcein-AM, with EC_50_ values in the micromolar range, demonstrating their specific influence on the activity of this efflux pump in an MDCK-MDR1 model, in which only P-gp is present. Also, compound **33** confirmed its effect as a P-gp modulator, displaying an EC_50_ value of 3.02 µM.

Regarding MRP1 inhibition, only compound **13** displayed an EC_50_ value in the micromolar range, while all these derivatives were not active on BCRP.

## 3. Discussion and Conclusions

In this work, we described the design, synthesis, and biological activity of novel *N^1^*,*N^4^*-disubstituted piperazine derivatives able to reverse the P-gp-mediated MDR in cancer cells overexpressing both P-gp and hCA XII transmembrane proteins. In several MDR cancer cells P-gp is co-localized with hCA XII isoform and the catalytic activity of hCA XII modulates the efflux activity of P-gp. Indeed, the structure of these dual inhibitors contains both P-gp and hCA XII binding moieties to synergistically overcome MDR. Therefore, these compounds had a piperazine ring carrying on one nitrogen atom three different methoxy-substituted arylalkyl moieties, which conferred good inhibitory activity on P-gp, and on the second nitrogen atom four different coumarin groups, to maintain a selectivity towards hCA XII, linked to the heterocycle by an alkoxy chain.

All compounds were able to inhibit the hCA XII isoform at nanomolar concentrations, to different extents: indeed, derivatives carrying the coumarin group **IV** were the least active, while the (*E*)-3-(3,4,5-trimethoxyphenyl)vinyl (**a**) and 3,4,5-trimethoxyphenyl (**b**) residues conferred higher potency on hCA XII, regardless of linker length. Moreover, most of our new derivatives displayed good inhibitory effects on P-gp, by increasing the cytotoxicity of doxorubicin in resistant K562/DOX cells, which overexpress only the transporter. All compounds with the 4,4-bis(4-methoxyphenyl)butyl (**c**) residue were the most potent ones, also showing RF values higher than 10.0 or 20.0, when used at 1 or 3 µM, respectively.

Selected compounds (**1**, **2**, **4**–**6**, **14**, **19** and **33**) were also studied in doxorubicin-resistant human adenocarcinoma colon cells (HT29/DOX) and in doxorubicin-resistant non-small cell lung cancer cells (A549/DOX), that overexpress both P-gp and hCA XII. The results showed that, in a specific environment where the two target proteins coexist, these compounds enhanced the cytotoxicity of the co-administered doxorubicin in a dose-dependent way, demonstrating a synergic effect on the MDR reversal activity due to the dual P-gp/hCA XII inhibition. In particular, compounds **4**, **5**, **14**, **19** and **33** were the most potent in both the doxorubicin-treated HT29/DOX and A549/DOX cell lines, when tested at a concentration of 3 µM. As regards SARs, it is not possible to find a certain correlation between the nature of the substituents on the two nitrogen atoms and the activity of these compounds, since their structures are quite different.

Moreover, we demonstrated that the reduced viability measured in the doxorubicin co-administration assay in the presence of our compounds was due to different retention of the anticancer drug within the cells. Indeed, all tested compounds were able to increase intracellular doxorubicin in HT29/DOX or A549/DOX cells, and the most potent derivative was **33**, which allowed the greatest accumulation of the drug in both resistant cell lines.

Evidence that these compounds (**4**, **5**, **14**, **19**, **33**) had higher activity in HT29/DOX and A549/DOX cells, that overexpress both P-gp and hCA XII, than in K562/DOX cells, overexpressing only P-gp, demonstrates that they were endowed with a synergistic mechanism on the two target proteins.

Together, these results confirm that most of our new *N^1^*,*N^4^*-disubstituted piperazine derivatives act as dual P-gp/hCA XII inhibitors. Among them, compound **33** resulted as a promising P-gp-mediated MDR reverser with a synergistic mechanism. Indeed, although it did not prove to be the most potent compound on the two target proteins taken individually (Ki = 46.8 nM on hCA XII isoform and RF = 9.9 and 31.7, at 1 and 3 µM concentrations, respectively, in K562/DOX cells, devoid of hCA XII), compound **33** displayed the greatest potency in enhancing the cytotoxicity and accumulation of doxorubicin in assays performed in cancer cells expressing both P-gp and hCA XII.

Interestingly, compounds **13**, **27** and **32** showed an unexpectedly higher cytotoxicity in resistant cells than in the sensitive ones, appearing as possible CS inducers. The mechanistic investigations revealed that they acted as multitarget agents: indeed, they induced oxidative stress and increased the amount of peroxidized plasma membrane lipids; they altered the chemical-physical properties of the membrane by increasing its fluidity and creating an unfavorable lipid environment for the activity of P-gp. Notwithstanding, compounds **13**, **27** and **32** proved to be more potent cytotoxic agents against P-gp-expressing cells than their sensitive counterparts due to increased generation of ROS and lipoperoxides, and an increased membrane fluidity, three events that are sufficient to induce CS in drug-resistant cells [41,42].

In conclusion, these results confirm our starting hypothesis that compounds containing both P-gp and hCA XII binding groups on the two nitrogen atoms of the piperazine ring were able to synergistically overcome MDR by acting as dual P-gp/hCA XII inhibitors. However, these piperazine derivatives, bearing arylalkyl and aryloxyalkyl substituents on the two nitrogen atoms, were generally less potent on the two proteins, P-gp and hCA XII, taken individually, than the previously synthesized *N*,*N*-bis(alkanol)amine aryl derivatives containing ester functions. Therefore, a possible development of this project could be the synthesis of new *N^1^*,*N^4^*-disubstituted piperazine derivatives containing aryl ester moieties to improve the potency of this series of molecules.

Furthermore, three collateral-sensitizing compounds were identified that could be considered interesting leads for the development of selective cytotoxic agents for MDR cells.

## 4. Materials and Methods

### 4.1. Chemistry

All melting points were taken on a Büchi apparatus and are uncorrected. NMR spectra were recorded on a Bruker Avance 400 spectrometer (400 MHz for ^1^H-NMR, 100 MHz for ^13^C-NMR). ^1^H and ^13^C NMR spectra were measured at room temperature (25 °C) in an appropriate solvent. ^1^H and ^13^C chemical shifts are expressed in ppm (δ) referenced to TMS. Spectral data are reported using the following abbreviations: s = singlet, d = doublet, dd = doublet of doublets, dt = doublet of triplets, t = triplet, bs = broad singlet, m = multiplet, and coupling constants are reported in Hz, followed by integration. Assignments of the ^13^C signals were performed using the attached proton test (APT) technique.

Chromatographic separations were performed on a silica gel column by flash chromatography (Kieselgel 40, 0.040–0.063 mm; Merck, Darmstadt, Germany). Yields are given after purification unless otherwise stated. The high-resolution mass spectrometry (HRMS) analysis was performed with a Thermo Finnigan LTQ Orbitrap mass spectrometer equipped with an electrospray ionization source (ESI). The accurate mass/charge ratio measure was carried out by introducing, via a syringe pump at 10 µL min^−1^, the sample solution (1.0 µg mL^−1^ in mQ water/acetonitrile 50:50), and the signal of the positive ions was acquired. The proposed experimental conditions allowed to monitoring the protonated molecules of studied compounds ([M+H]^+^ species), that were measured with a proper dwell time to achieve 60,000 units of resolution at Full Width at Half Maximum (FWHM). The elemental composition of each compound was calculated based on its measured accurate mass/charge ratio, accepting only results with an attribution error less than 2.5 ppm and a not integer double bond/ring equivalents (RDB) value, to consider only the protonated species [52]. The final compounds were checked by HPLC/diode array detection (DAD) analysis and were found a purity ≥ 95%. HPLC/DAD conditions of the analytical method, the chromatograms and UV spectra of final compounds **1**–**36** are included in the Supplementary Materials (Figures S8–S47).

Compounds were named following IUPAC rules as applied by ChemBioDraw Ultra 14.0 software. When reactions were performed in anhydrous conditions, the mixtures were maintained under nitrogen. Free bases **1**–**36** were transformed into the corresponding dihydrochlorides by treatment with a solution of acetyl chloride (2–3 equiv.) in anhydrous CH_3_OH. The salts were crystallized from abs. ethanol/petroleum ether.

#### 4.1.1. General Procedures for the Synthesis of Piperazine Derivatives **1**–**36**

To a solution of the proper methoxy-substituted aryl piperazine (**37**–**39**) [21] (1 equiv.) in dry acetonitrile, K_2_CO_3_ (1.2 equiv.) and the adequate bromoalkoxy-2*H*-chromen-2-one (**42**–**53**) (1.2 equiv.) were added. The mixture was stirred at 60 °C overnight, then the solvent was removed under reduced pressure and the residue was treated with CH_2_Cl_2_. The organic layer was washed twice with 10% NaOH solution, dried over Na_2_SO_4_ and concentrated under vacuum. Finally, the residue was purified by flash chromatography using the proper eluting system, yielding the desired compound as an oil.

*(E)-7-(2-(4-(3-(3,4,5-Trimethoxyphenyl)allyl)piperazin-1-yl)ethoxy)-2H-chromen-2-one* **1**. Following the general procedure, compound **1** (0.020 g, yield: 30.6%) was synthesized as a pale-yellow oil, starting from **37** [21] (0.040 g, 0.14 mmol) and **42** (0.044 g, 0.16 mmol) in 2.5 mL of dry acetonitrile. Free base: chromatographic eluent: CH_2_Cl_2_/CH_3_OH/NH_4_OH 95:5:0.5. ^1^H-NMR (400 MHz, CDCl_3_) δ: 7.62 (d, *J* = 9.6 Hz, 1H, C*H*=CH); 7.35 (d, *J* = 8.4 Hz, 1H, CH arom.); 6.84–6.80 (m, 2H, CH arom.); 6.60 (s, 2H, CH arom.); 6.44 (d, *J* = 16.0 Hz, 1H, C*H*=CH); 6.25–6.15 (m, 2H, C*H*=CH); 4.15 (t, *J* = 5.6 Hz, 2H, OCH_2_); 3.85 (s, 6H, OCH_3_); 3.82 (s, 3H, OCH_3_); 3.17 (d, *J* = 6.4 Hz, 2H, NCH_2_); 2.86 (t, *J* = 5.6 Hz, 2H, NCH_2_); 2.78–2.50 (m, 8H, NCH_2_) ppm. ^13^C-NMR (100 MHz, CDCl_3_) δ: 161.96 (C); 161.15 (C); 155.85 (C); 153.31 (C); 143.36 (CH); 137.81 (C); 133.11 (CH); 132.55 (C); 128.73 (CH); 125.87 (CH); 113.18 (CH); 113.00 (CH) 112.63 (C); 103.37 (CH); 101.50 (CH); 66.56 (CH_2_); 60.91 (OCH_3_); 60.87 (CH_2_); 56.84 (CH_2_); 56.05 (OCH_3_); 53.59 (CH_2_); 53.09 (CH_2_) ppm. ESI-HRMS (*m*/*z*) calculated for [M+H]^+^ ion species C_27_H_33_N_2_O_6_ = 481.2333, found 481.2339. Dihydrochloride: white solid; mp 140–143 °C.

*(E)-7-(3-(4-(3-(3,4,5-Trimethoxyphenyl)allyl)piperazin-1-yl)propoxy)-2H-chromen-2-one* **2**. Following the general procedure, compound **2** (0.040 g, yield: 33.9%) was synthesized as a pale-yellow oil, starting from **37** [21] (0.070 g, 0.24 mmol) and **43** [20] (0.081 g, 0.29 mmol) in 4.0 mL of dry acetonitrile. Free base: chromatographic eluent: CH_2_Cl_2_/CH_3_OH/NH_4_OH 95:5:0.5. ^1^H-NMR (400 MHz, CDCl_3_) δ: 7.61 (d, *J* = 9.2 Hz, 1H, C*H*=CH); 7.34 (d, *J* = 8.4 Hz, 1H, CH arom.); 6.83–6.80 (m, 2H, CH arom.); 6.60 (s, 2H, CH arom.); 6.44 (d, *J* = 15.6 Hz, 1H, C*H*=CH); 6.24–6.14 (m, 2H, C*H*=CH); 4.07 (t, *J* = 5.6 Hz, 2H, OCH_2_); 3.85 (s, 6H, OCH_3_); 3.82 (s, 3H, OCH_3_); 3.16 (d, *J* = 6.8 Hz, 2H, NCH_2_); 2.75–2.38 (m, 10H, NCH_2_); 2.03–1.95 (m, 2H, CH_2_) ppm. ^13^C-NMR (100 MHz, CDCl_3_) δ: 162.26 (C); 161.26 (C); 155.88 (C); 153.28 (C); 143.46 (CH); 137.67 (C); 132.99 (CH); 132.60 (C); 128.72 (CH); 126.06 (CH); 112.98 (CH); 112.45 (C); 103.27 (CH); 101.34 (CH); 66.84 (CH_2_); 60.94 (CH_2_); 60.92 (OCH_3_); 56.03 (OCH_3_); 54.87 (CH_2_); 53.21 (CH_2_); 26.48 (CH_2_) ppm. ESI-HRMS (*m*/*z*) calculated for [M+H]^+^ ion species C_28_H_35_N_2_O_6_ = 495.2490, found 495.2481. Dihydrochloride**:** white solid; mp 240–243 °C.

*(E)-7-(4-(4-(3-(3,4,5-Trimethoxyphenyl)allyl)piperazin-1-yl)butoxy)-2H-chromen-2-one* **3**. Following the general procedure, compound **3** (0.010 g, yield: 23.0%) was synthesized as a pale-yellow oil, starting from **37 [21]** (0.025 g, 0.086 mmol) and **44** (0.030 g, 0.10 mmol) in 2.0 mL of dry acetonitrile. Free base: chromatographic eluent: CH_2_Cl_2_/CH_3_OH/NH_4_OH 95:5:0.5. ^1^H-NMR (400 MHz, CDCl_3_) δ: 7.62 (d, *J* = 9.6 Hz, 1H, C*H*=CH); 7.35 (d, *J* = 8.4 Hz, 1H, CH arom.); 6.83–6.79 (m, 2H, CH arom.); 6.60 (s, 2H, CH arom.); 6.47 (d, *J* = 16.0 Hz, 1H, C*H*=CH); 6.25–6.14 (m, 2H, C*H*=CH); 4.03 (t, *J* = 6.4 Hz, 2H, OCH_2_); 3.86 (s, 6H, OCH_3_); 3.83 (s, 3H, OCH_3_); 3.27 (d, *J* = 6.4 Hz, 2H, NCH_2_); 2.90–2.52 (m, 10H, NCH_2_); 1.88–1.75 (m, 4H, CH_2_) ppm. ^13^C-NMR (100 MHz, CDCl_3_) δ: 162.28 (C); 161.24 (C); 155.93 (C); 153.32 (C); 143.41 (CH); 133.17 (CH); 132.56 (C); 128.71 (CH); 125.88 (CH); 112.99 (CH); 112.44 (C); 103.38 (CH); 101.31 (CH); 68.32 (CH_2_); 60.92 (OCH_3_); 60.88 (CH_2_); 58.03 (CH_2_); 56.06 (OCH_3_); 53.09 (CH_2_); 29.68 (CH_2_); 26.95 (CH_2_); 23.26 (CH_2_) ppm. ESI-HRMS (*m*/*z*) calculated for [M+H]^+^ ion species C_29_H_37_N_2_O_6_ = 509.2646, found 509.2641. Dihydrochloride: white solid; mp 160–163 °C.

*(E)-4-Methyl-7-(2-(4-(3-(3,4,5-trimethoxyphenyl)allyl)piperazin-1-yl)ethoxy)-2H-chromen-2-one* **4**. Following the general procedure, compound **4** (0.020 g, yield: 29.5%) was synthesized as a pale-yellow oil, starting from **37 [21]** (0.040 g, 0.14 mmol) and **45** (0.046 g, 0.16 mmol) in 5.0 mL of dry acetonitrile. Free base: chromatographic eluent: EtOAc/CH_3_OH/NH_4_OH 99:1:0.1. TLC: CH_2_Cl_2_/CH_3_OH/NH_4_OH 95:5:0.5. ^1^H-NMR (400 MHz, CDCl_3_) δ: 7.48 (d, *J* = 8.8 Hz, 1H, CH arom.); 6.86 (dd, *J* = 8.8, 2.4 Hz, 1H, CH arom.); 6.81 (d, *J* = 2.4 Hz, 1H, CH arom.); 6.60 (s, 2H, CH arom.); 6.45 (d, *J* = 15.6, 1H, C*H*=CH); 6.18 (dt, *J* = 15.6, 6.8 Hz, 1H, C*H*=CH); 6.13 (s, 1H, C*H*=C); 4.16 (t, *J* = 5.6 Hz, 2H, OCH_2_); 3.85 (s, 6H, OCH_3_); 3.83 (s, 3H, OCH_3_); 3.17 (d, *J* = 6.8 Hz, 2H, NCH_2_); 2.86 (t, *J* = 5.6 Hz, 2H, NCH_2_); 2.76–2.45 (m, 8H, CH_2_); 2.39 (s, 3H, CH_3_) ppm. ^13^C-NMR (100 MHz, CDCl_3_) δ: 161.77 (C); 161.25 (C); 155.24 (C); 153.32 (C); 152.49 (C); 133.18 (CH); 132.56 (C); 125.51 (CH); 113.70 (C); 112.70 (CH); 112.06 (CH); 103.37 (CH); 101.52 (CH); 66.51 (CH_2_); 60.93 (OCH_3_); 60.88 (CH_2_); 56.87 (CH_2_); 56.06 (OCH_3_); 53.58 (CH_2_); 53.09 (CH_2_); 18.67 (CH_3_) ppm. ESI-HRMS (*m*/*z*) calculated for [M+H]^+^ ion species C_28_H_35_N_2_O_6_ = 495.2490, found 495.2490. Dihydrochloride: white solid; mp 139–141 °C.

*(E)-4-Methyl-7-(3-(4-(3-(3,4,5-trimethoxyphenyl)allyl)piperazin-1-yl)propoxy)-2H-chromen-2-one* **5**. Following the general procedure, compound **5** (0.030 g, yield: 57.3%) was synthesized as a pale-yellow oil, starting from **37 [21]** (0.030 g, 0.10 mmol) and **46** (0.036 g, 0.12 mmol) in 5.0 mL of dry acetonitrile. Free base: chromatographic eluent: CH_2_Cl_2_/2-propanol/NH_4_OH 95:5:0.5. TLC: CH_2_Cl_2_/CH_3_OH/NH_4_OH 95:5:0.5. ^1^H-NMR (400 MHz, CDCl_3_) δ: 7.47 (d, *J* = 8.8 Hz, 1H, CH arom.); 6.85–6.81 (m, 2H, CH arom.); 6.61 (s, 2H, CH arom); 6.45 (d, *J* = 16.0 Hz, 1H, C*H*=CH); 6.20 (dt, *J* = 15.6, 6.8 Hz, 1H, C*H*=CH); 6.12 (s, 1H, C*H*=C); 4.07 (t, *J* = 6.4 Hz, 2H, OCH_2_); 3.85 (s, 6H, OCH_3_); 3.83 (s, 3H, OCH_3_); 3.16 (d, *J* = 6.8 Hz, 2H, NCH_2_); 2.85–2.43 (m, 10H, NCH_2_); 2.39 (s, 3H, CH_3_); 2.04–1.98 (m, 2H, CH_2_) ppm. ^13^C-NMR (100 MHz, CDCl_3_) δ: 162.08 (C); 161.37 (C); 155.29 (C); 153.30 (C); 152.59 (C); 133.06 (CH); 132.59 (C); 125.48 (CH); 113.51 (C); 112.70 (CH); 111.90 (CH); 103.29 (CH); 101.35 (CH); 66.80 (CH_2_); 61.23 (CH_2_); 60.88 (OCH_3_); 56.04 (OCH_3_); 54.90 (CH_2_); 53.18 (CH_2_); 26.50 (CH_2_); 18.70 (CH_3_) ppm. ESI-HRMS (*m*/*z*) calculated for [M+H]^+^ ion species C_29_H_37_N_2_O_6_ = 509.2646, found 509.2646. Dihydrochloride: white solid; mp 70–73 °C.

*(E)-4-Methyl-7-(4-(4-(3-(3,4,5-trimethoxyphenyl)allyl)piperazin-1-yl)butoxy)-2H-chromen-2-one* **6**. Following the general procedure, compound **6** (0.050 g, yield: 44.3%) was synthesized as a pale-yellow oil, starting from **37 [21]** (0.063 g, 0.22 mmol) and **47** (0.080 g, 0.26 mmol) in 3.0 mL of dry acetonitrile. Free base: chromatographic eluent: CH_2_Cl_2_/2-propanol/NH_4_OH 93:7:0.7. TLC: CH_2_Cl_2_/CH_3_OH/NH_4_OH 95:5:0.5. ^1^H-NMR (400 MHz, CDCl_3_) δ: 7.47 (d, *J* = 8.8 Hz, 1H, CH arom.); 6.82 (dd, *J* = 8.8, 2.4 Hz, 1H, CH arom.); 6.79 (d, *J* = 2.4 Hz, 1H, CH arom.); 6.60 (s, 2H, CH arom); 6.45 (d, *J* = 16.0 Hz, 1H, C*H*=CH); 6.19 (dt, *J* = 16.0, 6.8 Hz, 1H, C*H*=CH); 6.11 (s, 1H, C*H*=C); 4.03 (t, *J* = 6.4 Hz, 2H, OCH_2_); 3.85 (s, 6H, OCH_3_); 3.82 (s, 3H, OCH_3_); 3.16 (d, *J* = 6.8 Hz, 2H, NCH_2_); 2.78–2.41 (m, 10H, NCH_2_); 2.38 (s, 3H, CH_3_); 1.87–1.80 (m, 2H, CH_2_); 1.73–1.65 (m, 2H, CH_2_) ppm. ESI-HRMS (*m*/*z*) calculated for [M+H]^+^ ion species C_30_H_39_N_2_O_6_ = 523.2803, found 523.2811. Dihydrochloride: yellow solid; mp 180–183 °C.

*(E)-6-(2-(4-(3-(3,4,5-Trimethoxyphenyl)allyl)piperazin-1-yl)ethoxy)-2H-chromen-2-one* **7**. Following the general procedure, compound **7** (0.050 g, yield: 55.9%) was synthesized as a pale-yellow oil, starting from **37 [21]** (0.054 g, 0.19 mmol) and **48** (0.060 g, 0.22 mmol) in 5.0 mL of dry acetonitrile. Free base: chromatographic eluent: CH_2_Cl_2_/2-propanol/NH_4_OH 95:5:0.5. TLC: CH_2_Cl_2_/CH_3_OH/NH_4_OH 95:5:0.5. ^1^H-NMR (400 MHz, CDCl_3_) δ: 7.60 (d, *J* = 9.6 Hz, 1H, C*H*=CH); 7.20 (d, *J* = 8.8 Hz, 1H, CH arom); 7.08 (dd, *J* = 8.8, 2.8 Hz, 1H, CH arom); 6.89 (d, *J* = 2.8 Hz, 1H, CH arom); 6.57 (s, 2H, CH arom); 6.42 (d, *J* = 15.6 Hz, 1H, C*H*=CH); 6.37 (d, *J* = 9.6 Hz, 1H, C*H*=CH); 6.17 (dt, *J* = 15.6, 6.8 Hz, 1H, CH=C*H*); 4.10 (t, *J* = 5.6 Hz, 2H, OCH_2_); 3.82 (s, 6H, OCH_3_); 3.80 (s, 3H, OCH_3_); 3.15 (d, *J* = 6.8 Hz, 2H, NCH_2_); 2.82 (t, *J* = 5.6 Hz, 2H, NCH_2_); 2.77–2.40 (m, 8H, NCH_2_) ppm. ^13^C-NMR (100 MHz, CDCl_3_) δ: 160.91 (C); 155.20 (C); 153.29 (C); 148.49 (C); 143.18 (CH); 137.79 (C); 133.32 (CH); 132.47 (C); 125.53 (CH); 119.98 (CH); 119.15 (C); 117.81 (CH); 117.05 (CH); 110.98 (CH); 103.35 (CH); 66.61 (CH_2_); 60.89 (OCH_3_); 60.80 (CH_2_); 57.03 (CH_2_); 56.03 (OCH_3_); 53.45 (CH_2_); 52.99 (CH_2_) ppm. ESI-HRMS (*m*/*z*) calculated for [M+H]^+^ ion species C_27_H_33_N_2_O_6_ = 481.2333, found 481.2330. Dihydrochloride: yellow solid; mp 240–243 °C.

*(E)-6-(3-(4-(3-(3,4,5-Trimethoxyphenyl)allyl)piperazin-1-yl)propoxy)-2H-chromen-2-one* **8**. Following the general procedure, compound **8** (0.050 g, yield: 68.8%) was synthesized as a pale-yellow oil, starting from **37 [21]** (0.043 g, 0.15 mmol) and **49** (0.050 g, 0.18 mmol) in 5.0 mL of dry acetonitrile. Free base: chromatographic eluent: CH_2_Cl_2_/2-propanol/NH_4_OH 95:5:0.5. TLC: CH_2_Cl_2_/CH_3_OH/NH_4_OH 95:5:0.5. ^1^H-NMR (400 MHz, CDCl_3_) δ: 7.61 (d, *J* = 9.6 Hz, 1H, C*H*=CH); 7.21 (d, *J* = 8.8 Hz, 1H, CH arom); 7.06 (dd, *J* = 8.8, 2.8 Hz, 1H, CH arom); 6.88 (d, *J* = 2.8 Hz, 1H, CH arom); 6.58 (s, 2H, CH arom); 6.42 (d, *J* = 15.6 Hz, 1H, C*H*=CH); 6.37 (d, *J* = 9.6 Hz, 1H, CH=C*H*); 6.17 (dt, *J* = 15.6, 6.4 Hz, 1H, CH=C*H*); 4.00 (t, *J* = 6.4 Hz, 2H, OCH_2_); 3.83 (s, 6H, OCH_3_); 3.80 (s, 3H, OCH_3_); 3.13 (d, *J* = 6.4 Hz, 2H, NCH_2_); 2.70–2.20 (m, 10H, NCH_2_); 2.00–1.94 (m, 2H, CH_2_) ppm. ^13^C-NMR (100 MHz, CDCl_3_) δ: 160.98 (C); 155.47 (C); 153.28 (C); 148.36 (C); 143.24 (CH); 137.71 (C); 133.04 (CH); 132.56 (C); 125.95 (CH); 119.90 (CH); 119.14 (C); 117.78 (CH); 116.99 (CH); 110.78 (CH); 103.29 (CH); 66.93 (CH_2_); 60.89 (CH_2_); 56.03 (OCH_3_); 55.00 (CH_2_); 53.16 (CH_2_); 26.65 (CH_2_) ppm. ESI-HRMS (*m*/*z*) calculated for [M+H]^+^ ion species C_28_H_35_N_2_O_6_ = 495.2490, found 495.2496. Dihydrochloride: yellow solid; mp 242–244 °C.

*(E)-6-(4-(4-(3-(3,4,5-Trimethoxyphenyl)allyl)piperazin-1-yl)butoxy)-2H-chromen-2-one* **9**. Following the general procedure, compound **9** (0.060 g, yield: 57.6%) was synthesized as a pale-yellow oil, starting from **37** [21] (0.060 g, 0.21 mmol) and **50** (0.073 g, 0.25 mmol) in 5.0 mL of dry acetonitrile. Free base: chromatographic eluent: CH_2_Cl_2_/2-propanol/NH_4_OH 95:5:0.5. TLC: CH_2_Cl_2_/CH_3_OH/NH_4_OH 95:5:0.5. ^1^H-NMR (400 MHz, CDCl_3_) δ: 7.63 (d, *J* = 9.6 Hz, 1H, C*H*=CH); 7.25 (d, *J* = 9.2 Hz, 1H, CH arom); 7.09 (dd, *J* = 9.2, 2.8 Hz, 1H, CH arom); 6.89 (d, *J* = 2.8 Hz, 1H, CH arom); 6.61 (s, 2H, CH arom); 6.47–6.41 (m, 2H, C*H*=CH); 6.19 (dt, *J* = 15.6, 6.4 Hz, 1H, CH=C*H*); 4.00 (t, *J* = 6.0 Hz, 2H, OCH_2_); 3.86 (s, 6H, OCH_3_); 3.83 (s, 3H, OCH_3_); 3.16 (d, *J* = 6.4 Hz, 2H, NCH_2_); 2.80–2.40 (m, 10H, NCH_2_); 1.86–1.79 (m, 2H, CH_2_); 1.74–1.70 (m, 2H, CH_2_) ppm. ^13^C-NMR (100 MHz, CDCl_3_) δ: 160.96 (C); 155.47 (C); 153.26 (C); 148.30 (C); 143.25 (CH); 137.71 (C); 132.99 (CH); 132.57 (C); 125.95 (CH); 119.86 (CH); 119.13 (C); 117.74 (CH); 116.93 (CH); 110.74 (CH); 103.32 (CH); 68.39 (CH_2_); 60.89 (CH_2_); 58.11 (CH_2_); 56.02 (OCH_3_); 53.11 (CH_2_); 27.16 (CH_2_); 23.34 (CH_2_) ppm. ESI-HRMS (*m*/*z*) calculated for [M+H]^+^ ion species C_29_H_37_N_2_O_6_ = 509.2646, found 509.2640. Dihydrochloride: yellow solid; mp 250–252 °C.

*(E)-4-Methyl-6-(2-(4-(3-(3,4,5-trimethoxyphenyl)allyl)piperazin-1-yl)ethoxy)-2H-chromen-2-one* **10**. Following the general procedure, compound **10** (0.040 g, yield: 33.8%) was synthesized as a pale-yellow oil, starting from **37** [21] (0.070 g, 0.24 mmol) and **51** (0.081 g, 0.29 mmol) in 5.8 mL of dry acetonitrile. Free base: chromatographic eluent: CH_2_Cl_2_/2-propanol/NH_4_OH 95:5:0.5. TLC: CH_2_Cl_2_/CH_3_OH/NH_4_OH 95:5:0.5. ^1^H-NMR (400 MHz, CDCl_3_) δ: 7.23 (d, *J* = 9.2 Hz, 1H, CH arom); 7.08 (dd, *J* = 9.2, 2.8 Hz, 1H, CH arom); 7.01 (d, *J* = 2.8 Hz, 1H, CH arom); 6.59 (s, 2H, CH arom); 6.44 (d, *J* = 15.6 Hz, 1H, C*H*=CH); 6.26 (s, 1H, C*H*=C); 6.18 (dt, *J* = 15.6, 6.4 Hz, 1H, CH=C*H*); 4.13 (t, *J* = 6.4 Hz, 2H, OCH_2_); 3.84 (s, 6H, OCH_3_); 3.81 (s, 3H, OCH_3_); 3.16 (d, *J* = 6.4 Hz, 2H, NCH*_2_*); 2.85 (t, *J* = 6.4 Hz, 2H, NCH_2_); 2.77–2.50 (m, 8H, NCH_2_); 2.38 (s, 3H, CH_3_) ppm. ^13^C-NMR (100 MHz, CDCl_3_) δ: 160.86 (C); 155.11 (C); 153.30 (C); 151.93 (C); 147.95 (C); 137.89 (C); 133.39 (CH); 132.44 (C); 125.41 (CH); 120.43 (C); 119.20 (CH); 117.90 (CH); 115.47 (CH); 108.68 (CH); 103.44 (CH); 66.60 (CH_2_); 60.88 (OCH_3_); 60.78 (CH_2_); 57.07 (CH_2_); 56.06 (OCH_3_); 53.44 (CH_2_); 52.97 (CH_2_); 18.68 (CH_3_) ppm. ESI-HRMS (*m*/*z*) calculated for [M+H]^+^ ion species C_28_H_35_N_2_O_6_ = 495.2490, found 495.2482. Dihydrochloride: yellow solid; mp 230–232 (d) °C.

*(E)-4-Methyl-6-(3-(4-(3-(3,4,5-trimethoxyphenyl)allyl)piperazin-1-yl)propoxy)-2H-chromen-2-one* **11**. Following the general procedure, compound **11** (0.040 g, yield: 38.4%) was synthesized as a pale-yellow oil, starting from **37 [21]** (0.060 g, 0.21 mmol) and **52** (0.073 g, 0.25 mmol) in 5.0 mL of dry acetonitrile. Free base: chromatographic eluent: CH_2_Cl_2_/2-propanol/NH_4_OH 95:5:0.5. TLC: CH_2_Cl_2_/CH_3_OH/NH_4_OH 95:5:0.5. ^1^H-NMR (400 MHz, CDCl_3_) δ: 7.21 (d, *J* = 9.2 Hz, 1H, CH arom); 7.07 (dd, *J* = 9.2, 2.8 Hz, 1H, CH arom); 6.98 (d, *J* = 2.8 Hz, 1H, CH arom); 6.58 (s, 2H, CH arom); 6.42 (d, *J* = 15.6 Hz, 1H, C*H*=CH); 6.25 (s, 1H, C*H*=C); 6.17 (dt, *J* = 15.6, 6.4 Hz, 1H, CH=C*H*); 4.03 (t, *J* = 6.4 Hz, 2H, OCH_2_); 3.83 (s, 6H, OCH_3_); 3.80 (s, 3H, OCH_3_); 3.14 (d, *J* = 6.4 Hz, 2H, NCH_2_); 2.73–2.43 (m, 10H, NCH_2_); 2.38 (s, 3H, CH_3_); 2.02–1.95 (m, 2H, CH_2_) ppm. ^13^C-NMR (100 MHz, CDCl_3_) δ: 160.88 (C); 155.37 (C); 153.32 (C); 151.93 (C); 147.85 (C); 137.89 (C); 133.12 (CH); 132.53 (C); 125.79 (CH); 120.43 (C); 119.09 (CH); 117.86 (CH); 115.43 (CH); 108.56 (CH); 103.46 (CH); 66.98 (CH_2_); 60.87 (OCH_3_); 60.84 (CH_2_); 56.06 (OCH_3_); 55.00 (CH_2_); 53.14 (CH_2_); 53.08 (CH_2_); 26.68 (CH_2_); 18.67 (CH_3_) ppm. ESI-HRMS (*m*/*z*) calculated for [M+H]^+^ ion species C_29_H_37_N_2_O_6_ = 509.2646, found 509.2644. Dihydrochloride: yellow solid; mp 234–236 (d) °C.

*(E)-4-Methyl-6-(4-(4-(3-(3,4,5-trimethoxyphenyl)allyl)piperazin-1-yl)butoxy)-2H-chromen-2-one* **12**. Following the general procedure, compound **12** (0.050 g, yield: 46.7%) was synthesized as a pale-yellow oil, starting from **37 [21]** (0.060 g, 0.21 mmol) and **53** (0.077 g, 0.25 mmol) in 5.0 mL of dry acetonitrile. Free base: chromatographic eluent: CH_2_Cl_2_/2-propanol/NH_4_OH 95:5:0.5. TLC: CH_2_Cl_2_/CH_3_OH/NH_4_OH 95:5:0.5. ^1^H-NMR (400 MHz, CDCl_3_) δ: 7.22 (d, *J* = 9.2 Hz, 1H, CH arom); 7.06 (dd, *J* = 9.2, 2.8 Hz, 1H, CH arom); 6.98 (d, *J* = 2.8 Hz, 1H, CH arom); 6.59 (s, 2H, CH arom); 6.42 (d, *J* = 15.6 Hz, 1H, C*H*=CH); 6.26 (s, 1H, C*H*=C); 6.17 (dt, *J* = 15.6, 6.4 Hz, 1H, CH=C*H*); 3.99 (t, *J* = 6.4 Hz, 2H, OCH_2_); 3.84 (s, 6H, OCH_3_); 3.81 (s, 3H, OCH_3_); 3.14 (d, *J* = 6.4 Hz, 2H, NCH_2_); 2.70–2.40 (m, 10H, NCH_2_); 2.38 (s, 3H, CH_3_); 1.85–1.78 (m, 2H, CH_2_); 1.72–1.67 (m, 2H, CH_2_) ppm. ^13^C-NMR (100 MHz, CDCl_3_) δ: 160.95 (C); 155.40 (C); 153.31 (C); 151.96 (C); 147.80 (C); 137.81 (C); 133.03 (CH); 132.57 (C); 125.95 (CH); 120.44 (C); 119.04 (CH); 117.88 (CH); 115.44 (CH); 108.46 (CH); 103.38 (CH); 68.45 (CH_2_); 60.91 (CH_2_); 58.15 (CH_2_); 56.05 (OCH_3_); 53.14 (CH_2_); 27.26 (CH_2_); 23.38 (CH_2_); 18.70 (CH_3_) ppm. ESI-HRMS (*m*/*z*) calculated for [M+H]^+^ ion species C_30_H_39_N_2_O_6_ = 523.2803, found 523.2797. Dihydrochloride: yellow solid; mp 204–207 °C.

*7-(2-(4-(3,4,5-Trimethoxybenzyl)piperazin-1-yl)ethoxy)-2H-chromen-2-one* **13**. Following the general procedure, compound **13** (0.12 g, yield: 78.0%) was synthesized as a yellow oil, starting from **38 [21]** (0.090 g, 0.34 mmol) and **42** (0.11 g, 0.40 mmol) in 5.0 mL of dry acetonitrile. Free base: chromatographic eluent: CH_2_Cl_2_/CH_3_OH/NH_4_OH 95:5:0.5. ^1^H-NMR (400 MHz, CDCl_3_) δ: 7.58 (d, *J* = 9.2 Hz, 1H, C*H*=CH); 7.31 (d, *J* = 8.4 Hz, 1H, CH arom.); 6.79 (dd, *J* = 8.8, 2.4 Hz, 1H, CH arom.); 6.75 (d, *J* = 2.4 Hz, 1H, CH arom.); 6.52 (s, 2H, CH arom.); 6.18 (d, *J* = 9.2 Hz, 1H, C*H*=CH); 4.11 (t, *J* = 5.6 Hz, 2H, OCH_2_); 3.81 (s, 6H, OCH_3_); 3.78 (s, 3H, OCH_3_); 3.42 (s, 2H, NCH_2_); 2.81 (t, *J* = 5.6 Hz, 2H, NCH_2_); 2.70–2.55 (m, 4H, NCH_2_); 2.54–2.35 (m, 4H, NCH_2_) ppm. ^13^C-NMR (100 MHz, CDCl_3_) δ: 161.94 (C); 161.10 (C); 155.80 (C); 153.07 (C); 143.39 (CH); 136.97 (C); 133.63 (C); 128.76 (CH); 113.08 (CH); 112.94 (CH); 112.60 (C); 105.89 (CH); 101.48 (CH); 66.59 (CH_2_); 63.11 (CH_2_); 60.78 (OCH_3_); 56.81 (CH_2_); 56.11 (OCH_3_); 53.57 (CH_2_); 52.89 (CH_2_) ppm. ESI-HRMS (*m*/*z*) calculated for [M+H]^+^ ion species C_25_H_31_N_2_O_6_ = 455.2177, found 455.2177. Dihydrochloride: white solid; mp 239–241 °C.

*7-(3-(4-(3,4,5-Trimethoxybenzyl)piperazin-1-yl)propoxy)-2H-chromen-2-one* **14**. Following the general procedure, compound **14** (0.11 g, yield: 89.7%) was synthesized as a yellow oil, starting from **38** [21] (0.070 g, 0.26 mmol) and **43 [20]** (0.090 g, 0.32 mmol) in 5.0 mL of dry acetonitrile. Free base: chromatographic eluent: CH_2_Cl_2_/2-propanol/NH_4_OH 95:5:0.5. ^1^H-NMR (400 MHz, CDCl_3_) δ: 7.60 (d, *J* = 9.2 Hz, 1H, C*H*=CH); 7.32 (d, *J* = 8.4 Hz, 1H, CH arom.); 6.83–6.77 (m, 2H, CH arom.); 6.53 (s, 2H, CH arom.); 6.20 (d, *J* = 9.2 Hz, 1H, C*H*=CH); 4.05 (t, *J* = 5.6 Hz, 2H, OCH_2_); 3.82 (s, 6H, OCH_3_); 3.80 (s, 3H, OCH_3_); 3.42 (s, 2H, NCH_2_); 2.66–2.35 (m, 10H, NCH_2_); 2.00–1.94 (m, 2H, CH_2_) ppm. ^13^C-NMR (100 MHz, CDCl_3_) δ: 162.28 (C); 161.19 (C); 155.88 (C); 153.06 (C); 143.43 (CH); 136.90 (C); 133.98 (C); 128.71 (CH); 112.94 (CH); 112.44 (C); 105.84 (CH); 101.38 (CH); 66.88 (CH_2_); 63.21 (CH_2_); 60.81 (OCH_3_); 56.11 (OCH_3_); 54.86 (CH_2_); 53.25 (CH_2_); 53.07 (CH_2_); 26.51 (CH_2_) ppm. ESI-HRMS (*m*/*z*) calculated for [M+H]^+^ ion species C_26_H_33_N_2_O_6_ = 469.2333, found 469.2331. Dihydrochloride: white solid; mp 220–223 °C.

*7-(4-(4-(3,4,5-Trimethoxybenzyl)piperazin-1-yl)butoxy)-2H-chromen-2-one* **15**. Following the general procedure, compound **15** (0.12 g, yield: 82.9%) was synthesized as a yellow oil, starting from **38** [21] (0.080 g, 0.30 mmol) and **44** (0.10 g, 0.36 mmol) in 5.0 mL of dry acetonitrile. Free base: chromatographic eluent: CH_2_Cl_2_/2-propanol/NH_4_OH 95:5:0.5. ^1^H-NMR (400 MHz, CDCl_3_) δ: 7.61 (d, *J* = 9.2 Hz, 1H, C*H*=CH); 7.33 (d, *J* = 8.4 Hz, 1H, CH arom.); 6.81–6.77 (m, 2H, CH arom.); 6.54 (s, 2H, CH arom.); 6.21 (d, *J* = 9.2 Hz, 1H, C*H*=CH); 4.01 (t, *J* = 5.6 Hz, 2H, OCH_2_); 3.83 (s, 6H, OCH_3_); 3.81 (s, 3H, OCH_3_); 3.42 (s, 2H, NCH_2_); 2.66–2.27 (m, 10H, NCH_2_); 1.85–1.78 (m, 2H, CH_2_); 1.70–1.64 (m, 2H, CH_2_) ppm. ^13^C-NMR (100 MHz, CDCl_3_) δ: 162.28 (C); 161.27 (C); 155.90 (C); 153.05 (C); 143.48 (CH); 136.82 (C); 133.98 (C); 128.73 (CH); 112.98 (CH); 112.94 (CH); 112.40 (C); 105.78 (CH); 101.27 (CH); 68.33 (CH_2_); 63.23 (CH_2_); 60.84 (OCH_3_); 58.10 (CH_2_); 56.11 (OCH_3_); 53.22 (CH_2_); 53.06 (CH_2_); 26.99 (CH_2_); 23.32 (CH_2_) ppm. ESI-HRMS (*m*/*z*) calculated for [M+H]^+^ ion species C_27_H_35_N_2_O_6_ = 483.2490, found 483.2484. Dihydrochloride: white solid; mp 215–218 °C.

*4-Methyl-7-(2-(4-(3,4,5-trimethoxybenzyl)piperazin-1-yl)ethoxy)-2H-chromen-2-one* **16**. Following the general procedure, compound **16** (0.090 g, yield: 94.7%) was synthesized as a pale-yellow oil, starting from **38 [21]** (0.054 g, 0.20 mmol) and **45** (0.056 g, 0.24 mmol) in 5.0 mL of dry acetonitrile. Free base: chromatographic eluent: CH_2_Cl_2_/2-propanol/NH_4_OH 95:5:0.5. ^1^H-NMR (400 MHz, CDCl_3_) δ: 7.44 (d, *J* = 8.8 Hz, 1H, CH arom.); 6.82 (dd, *J* = 8.8, 2.4 Hz, 1H, CH arom.); 6.77 (d, *J* = 2.4 Hz, 1H, CH arom.); 6.53 (s, 2H, CH arom.); 6.08 (s, 1H, C*H*=C); 4.13 (t, *J* = 5.6 Hz, 2H, OCH_2_); 3.82 (s, 6H, OCH_3_); 3.80 (s, 3H, OCH_3_); 3.42 (s, 2H, NCH_2_); 2.82 (t, *J* = 5.6 Hz, 2H, NCH_2_); 2.73–2.39 (m, 8H, NCH_2_); 2.35 (s, 3H, CH_3_) ppm. ^13^C-NMR (100 MHz, CDCl_3_) δ: 161.78 (C); 161.21 (C); 155.20 (C); 153.07 (C); 152.51 (C); 136.95 (C); 133.83 (C); 125.51 (CH); 113.64 (C); 112.65 (CH); 111.96 (CH); 105.86 (CH); 101.51 (CH); 66.56 (CH_2_); 63.16 (CH_2_); 60.80 (OCH_3_); 56.87 (CH_2_); 56.12 (OCH_3_); 53.66 (CH2); 52.94 (CH_2_); 18.62 (CH_3_) ppm. ESI-HRMS (*m*/*z*) calculated for [M+H]^+^ ion species C_26_H_33_N_2_O_6_ = 469.2333, found 469.2325. Dihydrochloride: white solid; mp 231–234 °C.

*4-Methyl-7-(3-(4-(3,4,5-trimethoxybenzyl)piperazin-1-yl)propoxy)-2H-chromen-2-one* **17**. Following the general procedure, compound **17** (0.050 g, yield: 55.4%) was synthesized as a yellow oil, starting from **38 [21]** (0.050 g, 0.19 mmol) and **46** (0.066 g, 0.22 mmol) in 5.0 mL of dry acetonitrile. Free base: chromatographic eluent: CH_2_Cl_2_/2-propanol/NH_4_OH 95:5:0.5. ^1^H-NMR (400 MHz, CDCl_3_) δ: 7.46 (d, *J* = 8.8 Hz, 1H, CH arom.); 6.83–6.80 (m, 2H, CH arom.); 6.55 (s, 2H, CH arom.); 6.10 (s, 1H, C*H*=C); 4.06 (t, *J* = 6.0 Hz, 2H, OCH_2_); 3.84 (s, 6H, OCH_3_); 3.81 (s, 3H, OCH_3_); 3.43 (s, 2H, NCH_2_); 2.64–2.40 (m, 10H, NCH_2_); 2.37 (s, 3H, CH_3_); 2.00–1.97 (m, 2H, CH_2_) ppm. ^13^C-NMR (100 MHz, CDCl_3_) δ: 162.09 (C); 161.29 (C); 155.28 (C); 153.07 (C); 152.54 (C); 136.94 (C); 133.91 (C); 125.47 (CH); 113.49 (C); 112.64 (CH); 111.87 (CH); 105.86 (CH); 101.40 (CH); 66.84 (CH_2_); 63.20 (CH_2_); 60.83 (OCH_3_); 56.13 (OCH_3_); 54.88 (CH_2_); 53.22 (CH_2_); 53.03 (CH_2_); 26.50 (CH_2_); 18.64 (CH_3_) ppm. ESI-HRMS (*m*/*z*) calculated for [M+H]^+^ ion species C_27_H_35_N_2_O_6_ = 483.2490, found 483.2489. Dihydrochloride: white solid; mp 233–236 °C.

*4-Methyl-7-(4-(4-(3,4,5-trimethoxybenzyl)piperazin-1-yl)butoxy)-2H-chromen-2-one* **18**. Following the general procedure, compound **18** (0.060 g, yield: 64.6%) was synthesized as a pale-yellow oil, starting from **38 [21]** (0.050 g, 0.19 mmol) and **47** (0.069 g, 0.22 mmol) in 5.0 mL of dry acetonitrile. Free base: chromatographic eluent: CH_2_Cl_2_/2-propanol/NH_4_OH 95:5:0.5. ^1^H-NMR (400 MHz, CDCl_3_) δ: 7.43 (d, *J* = 8.8 Hz, 1H, CH arom.); 6.80–6.74 (m, 2H, CH arom.); 6.52 (s, 2H, CH arom.); 6.06 (s, 1H, C*H*=C); 3.99 (t, *J* = 6.4 Hz, 2H, OCH_2_); 3.81 (s, 6H, OCH_3_); 3.78 (s, 3H, OCH_3_); 3.42 (s, 2H, NCH_2_); 2.70–2.37 (m, 10H, NCH_2_); 2.34 (s, 3H, CH_3_); 1.83–1.77 (m, 2H, CH_2_); 1.73–1.64 (m, 2H, CH_2_) ppm. ^13^C-NMR (100 MHz, CDCl_3_) δ: 162.06 (C); 161.26 (C); 155.25 (C); 153.05 (C); 152.57 (C); 136.95 (C); 133.65 (C); 125.49 (CH); 113.43 (C); 112.60 (CH); 111.79 (CH); 105.92 (CH); 101.30 (CH); 68.22 (CH_2_); 63.07 (CH_2_); 60.79 (OCH_3_); 57.92 (CH_2_); 56.11 (OCH_3_); 52.97 (CH_2_); 52.76 (CH_2_); 26.95 (CH_2_); 23.09 (CH_2_); 18.61 (CH_3_) ppm. ESI-HRMS (*m*/*z*) calculated for [M+H]^+^ ion species C_28_H_37_N_2_O_6_ = 497.2646, found 497.2655. Dihydrochloride: white solid; mp 216–219 °C.

*6-(2-(4-(3,4,5-Trimethoxybenzyl)piperazin-1-yl)ethoxy)-2H-chromen-2-one* **19**. Following the general procedure, compound **19** (0.080 g, yield: 67.2%) was synthesized as a pale-yellow oil, starting from **38** [21] (0.070 g, 0.26 mmol) and **48** (0.084 g, 0.32 mmol) in 5.0 mL of dry acetonitrile. Free base: chromatographic eluent: CH_2_Cl_2_/2-propanol/NH_4_OH 93:7:0.7. TLC: CH_2_Cl_2_/CH_3_OH/NH_4_OH 95:5:0.5. ^1^H-NMR (400 MHz, CDCl_3_) δ: 7.60 (d, *J* = 9.6 Hz, 1H, C*H*=CH); 7.19 (d, *J* = 8.8 Hz, 1H, CH arom.); 7.06 (dd, *J* = 8.8, 2.4 Hz, 1H, CH arom.); 6.88 (d, *J* = 2.4 Hz, 1H, CH arom.); 6.52 (s, 2H, CH arom.); 6.36 (d, *J* = 9.6 Hz, 1H, C*H*=CH); 4.09 (t, *J* = 5.6 Hz, 2H, OCH_2_); 3.81 (s, 6H, OCH_3_); 3.79 (s, 3H, OCH_3_); 3.41 (s, 2H, NCH_2_); 2.80 (t, *J* = 5.6 Hz, 2H, NCH_2_); 2.72–2.32 (m, 8H, NCH_2_) ppm. ^13^C-NMR (100 MHz, CDCl_3_) δ: 160.91 (C); 155.24 (C); 153.05 (C); 148.45 (C); 143.23 (CH); 136.85 (C); 133.88 (C); 120.00 (CH); 119.14 (C); 117.78 (CH); 117.01 (CH); 110.95 (CH); 105.77 (CH); 66.71 (CH_2_); 63.17 (CH_2_); 60.82 (OCH_3_); 57.09 (CH_2_); 56.10 (OCH_3_); 53.68 (CH_2_); 52.96 (CH_2_) ppm. ESI-HRMS (*m*/*z*) calculated for [M+H]^+^ ion species C_25_H_31_N_2_O_6_ = 455.2177, found 455.2176. Dihydrochloride: white solid; mp 180–182 °C.

*6-(3-(4-(3,4,5-Trimethoxybenzyl)piperazin-1-yl)propoxy)-2H-chromen-2-one* **20**. Following the general procedure, compound **20** (0.050 g, yield: 71.1%) was synthesized as a pale-yellow oil, starting from **38** [21] (0.040 g, 0.15 mmol) and **49** (0.051 g, 0.18 mmol) in 5.0 mL of dry acetonitrile. Free base: chromatographic eluent: CH_2_Cl_2_/2-propanol/NH_4_OH 95:5:0.5. TLC: CH_2_Cl_2_/CH_3_OH/NH_4_OH 95:5:0.5. ^1^H-NMR (400 MHz, CDCl_3_) δ: 7.63 (d, *J* = 9.6 Hz, 1H, C*H*=CH); 7.24 (d, *J* = 8.8 Hz, 1H, CH arom.); 7.09 (dd, *J* = 8.8, 2.4 Hz, 1H, CH arom.); 6.90 (d, *J* = 2.4 Hz, 1H, CH arom.); 6.55 (s, 2H, CH arom.); 6.41 (d, *J* = 9.6 Hz, 1H, C*H*=CH); 4.03 (t, *J* = 6.4 Hz, 2H, OCH_2_); 3.85 (s, 6H, OCH_3_); 3.82 (s, 3H, OCH_3_); 3.44 (s, 2H, NCH_2_); 2.67–2.40 (m, 10H, NCH_2_); 2.02–1.94 (m, 2H, CH_2_) ppm. ^13^C-NMR (100 MHz, CDCl_3_) δ: 160.98 (C); 155.51 (C); 153.09 (C); 148.40 (C); 143.19 (CH); 136.94 (C); 133.98 (C); 119.91 (CH); 119.16 (C); 117.82 (CH); 117.05 (CH); 110.81 (CH); 105.84 (CH); 67.03 (CH_2_); 63.24 (CH_2_); 60.85 (OCH_3_); 56.14 (OCH_3_); 55.05 (CH_2_); 53.31 (CH_2_); 53.09 (CH_2_); 26.74 (CH_2_) ppm. ESI-HRMS (*m*/*z*) calculated for [M+H]^+^ ion species C_26_H_33_N_2_O_6_ = 469.2333, found 469.2332. Dihydrochloride: white solid; mp 243–245 °C.

*6-(4-(4-(3,4,5-Trimethoxybenzyl)piperazin-1-yl)butoxy)-2H-chromen-2-one* **21**. Following the general procedure, compound **21** (0.040 g, yield: 55.3%) was synthesized as a pale-yellow oil, starting from **38** [21] (0.040 g, 0.15 mmol) and **50** (0.053 g, 0.18 mmol) in 5.0 mL of dry acetonitrile. Free base: chromatographic eluent: CH_2_Cl_2_/2-propanol/NH_4_OH 95:5:0.5. TLC: CH_2_Cl_2_/CH_3_OH/NH_4_OH 95:5:0.5. ^1^H-NMR (400 MHz, CDCl_3_) δ: 7.63 (d, *J* = 9.2 Hz, 1H, C*H*=CH); 7.25 (d, *J* = 8.8 Hz, 1H, CH arom.); 7.08 (dd, *J* = 8.8, 2.8 Hz, 1H, CH arom.); 6.88 (d, *J* = 2.8 Hz, 1H, CH arom.); 6.55 (s, 2H, CH arom.); 6.41 (d, *J* = 9.2 Hz, 1H, C*H*=CH); 3.99 (t, *J* = 6.0 Hz, 2H, OCH_2_); 3.85 (s, 6H, OCH_3_); 3.82 (s, 3H, OCH_3_); 3.43 (s, 2H, NCH_2_); 2.67–2.40 (m, 10H, NCH_2_); 1.83–1.78 (m, 2H, CH_2_); 1.72–1.66 (m, 2H, CH_2_) ppm. ^13^C-NMR (100 MHz, CDCl_3_) δ: 161.03 (C); 155.51 (C); 153.07 (C); 148.36 (C); 143.23 (CH); 136.85 (C); 133.98 (C); 119.88 (CH); 119.16 (C); 117.84 (CH); 117.05 (CH); 110.71 (CH); 105.78 (CH); 68.43 (CH_2_); 63.27 (CH_2_); 60.86 (OCH_3_); 58.21 (CH_2_); 56.12 (OCH_3_); 53.25 (CH_2_); 53.09 (CH_2_); 27.23 (CH_2_); 23.45 (CH_2_) ppm. ESI-HRMS (*m*/*z*) calculated for [M+H]^+^ ion species C_27_H_35_N_2_O_6_ = 483.2490, found 483.2487. Dihydrochloride: white solid; mp 237–239 °C.

*4-Methyl-6-(2-(4-(3,4,5-trimethoxybenzyl)piperazin-1-yl)ethoxy)-2H-chromen-2-one* **22**. Following the general procedure, compound **22** (0.050 g, yield: 52.0%) was synthesized as a pale-yellow oil, starting from **38** [21] (0.050 g, 0.19 mmol) and **51** (0.064 g, 0.23 mmol) in 4.2 mL of dry acetonitrile. Free base: chromatographic eluent: CH_2_Cl_2_/CH_3_OH/NH_4_OH 95:5:0.5. ^1^H-NMR (400 MHz, CDCl_3_) δ: 7.23 (d, *J* = 9.2 Hz, 1H, CH arom); 7.10 (dd, *J* = 9.2, 2.8 Hz, 1H, CH arom); 7.03 (d, *J* = 2.8 Hz, 1H, CH arom); 6.55 (s, 2H, CH arom); 6.27 (s, 1H, C*H*=C); 4.13 (t, *J* = 6.0 Hz, 2H, OCH_2_); 3.84 (s, 6H, OCH_3_); 3.82 (s, 3H, OCH_3_); 3.45 (s, 2H, NCH_2_); 2.84 (t, *J* = 6.0 Hz, 2H, NCH_2_); 2.71–2.58 (m, 4H, NCH_2_); 2.57–2.47 (m, 4H, NCH_2_); 2.38 (s, 3H, CH_3_) ppm. ^13^C-NMR (100 MHz, CDCl_3_) δ: 160.91 (C); 155.13 (C); 153.10 (C); 151.93 (C); 147.97 (C); 133.66 (C); 120.45 (C); 119.20 (CH); 117.93 (CH); 115.50 (CH); 108.66 (CH); 105.95 (CH); 66.67 (CH_2_); 63.17 (CH_2_); 60.83 (OCH_3_); 57.14 (CH_2_); 56.15 (OCH_3_); 53.65 (CH_2_); 52.90 (CH_2_); 18.71 (CH_3_) ppm. ESI-HRMS (*m*/*z*) calculated for [M+H]^+^ ion species C_26_H_33_N_2_O_6_ = 469.2333, found 469.2342. Dihydrochloride: yellow solid; mp 240–242 (d) °C.

*4-Methyl-6-(3-(4-(3,4,5-trimethoxybenzyl)piperazin-1-yl)propoxy)-2H-chromen-2-one* **23**. Following the general procedure, compound **23** (0.050 g, yield: 55.4%) was synthesized as a pale-yellow oil, starting from **38** [21] (0.050 g, 0.19 mmol) and **52** (0.066 g, 0.23 mmol) in 4.2 mL of dry acetonitrile. Free base: chromatographic eluent: CH_2_Cl_2_/CH_3_OH/NH_4_OH 95:5:0.5. ^1^H-NMR (400 MHz, CDCl_3_) δ: 7.21 (d, *J* = 9.2 Hz, 1H, CH arom); 7.07 (dd, *J* = 9.2, 2.8 Hz, 1H, CH arom); 6.98 (d, *J* = 2.8 Hz, 1H, CH arom); 6.53 (s, 2H, CH arom); 6.25 (s, 1H, C*H*=C); 4.03 (t, *J* = 6.4 Hz, 2H, OCH_2_); 3.83 (s, 6H, OCH_3_); 3.80 (s, 3H, OCH_3_); 3.42 (s, 2H, NCH_2_); 2.70–2.40 (m, 10H, NCH_2_); 2.38 (s, 3H, CH_3_); 2.01–1.94 (m, 2H, CH_2_) ppm. ^13^C-NMR (100 MHz, CDCl_3_) δ: 160.94 (C); 155.38 (C); 153.06 (C); 152.01 (C); 147.80 (C); 136.92 (C); 133.90 (C); 120.41 (C); 119.11 (CH); 117.86 (CH); 115.41 (CH); 108.48 (CH); 105.85 (CH); 67.02 (CH_2_); 63.20 (CH_2_); 60.82 (OCH_3_); 56.12 (OCH_3_); 55.06 (CH_2_); 53.27 (CH_2_); 53.03 (CH_2_); 26.74 (CH_2_); 18.71 (CH_3_) ppm. ESI-HRMS (*m*/*z*) calculated for [M+H]^+^ ion species C_27_H_35_N_2_O_6_ = 483.2490, found 483.2497. Dihydrochloride: yellow solid; mp 244–246 (d) °C.

*4-Methyl-6-(4-(4-(3,4,5-trimethoxybenzyl)piperazin-1-yl)butoxy)-2H-chromen-2-one* **24**. Following the general procedure, compound **24** (0.060 g, yield: 64.4%) was synthesized as a pale-yellow oil, starting from **38** [21] (0.050 g, 0.19 mmol) and **53** (0.070 g, 0.23 mmol) in 4.2 mL of dry acetonitrile. Free base: chromatographic eluent: CH_2_Cl_2_/CH_3_OH/NH_4_OH 95:5:0.5. ^1^H-NMR (400 MHz, CDCl_3_) δ: 7.24 (d, *J* = 9.2 Hz, 1H, CH arom); 7.07 (dd, *J* = 9.2, 2.8 Hz, 1H, CH arom); 6.99 (d, *J* = 2.8 Hz, 1H, CH arom); 6.55 (s, 2H, CH arom); 6.28 (s, 1H, C*H*=C); 4.00 (t, *J* = 6.4 Hz, 2H, OCH_2_); 3.84 (s, 6H, OCH_3_); 3.82 (s, 3H, OCH_3_); 3.43 (s, 2H, NCH_2_); 2.60–2.39 (m, 10H, NCH_2_); 2.38 (s, 3H, CH_3_); 1.86–1.79 (m, 2H, CH_2_); 1.73–1.65 (m, 2H, CH_2_) ppm. ^13^C-NMR (100 MHz, CDCl_3_) δ: 160.93 (C); 155.39 (C); 153.04 (C); 152.01 (C); 147.75 (C); 136.90 (C); 133.91 (C); 120.40 (C); 119.06 (CH); 117.83 (CH); 115.37 (CH); 108.41 (CH); 105.85 (CH); 68.44 (CH_2_); 63.20 (CH_2_); 60.80 (OCH_3_); 58.16 (CH_2_); 56.11 (OCH_3_); 53.19 (CH_2_); 53.02 (CH_2_); 27.26 (CH_2_); 23.39 (CH_2_); 18.69 (CH_3_) ppm. ESI-HRMS (*m*/*z*) calculated for [M+H]^+^ ion species C_28_H_37_N_2_O_6_ = 497.2646, found 497.2653. Dihydrochloride: yellow solid; mp 236–238 (d) °C.

*7-(2-(4-(4,4-Bis(4-methoxyphenyl)butyl)piperazin-1-yl)ethoxy)-2H-chromen-2-one* **25**. Following the general procedure, compound **25** (0.060 g, yield: 78.5%) was synthesized as a pale-yellow oil, starting from **39** [21] (0.050 g, 0.14 mmol) and **42** (0.045 g, 0.17 mmol) in 5.0 mL of dry acetonitrile. Free base: chromatographic eluent: CH_2_Cl_2_/2-propanol/NH_4_OH 96:4:0.6. ^1^H-NMR (400 MHz, CDCl_3_) δ: 7.61 (d, *J* = 9.2 Hz, 1H, C*H*=CH); 7.34 (d, *J* = 8.4 Hz, 1H, CH arom.); 7.11 (d, *J* = 8.8 Hz, 4H, CH arom.); 6.84–6.78 (m, 6H, CH arom.); 6.23 (d, *J* = 9.2 Hz, 1H, C*H*=CH); 4.13 (t, *J* = 6.0 Hz, 2H, OCH_2_); 3.78 (t, *J* = 8.0 Hz, 1H, CH); 3.75 (s, 6H, OCH_3_); 2.82 (t, *J* = 6.0 Hz, 2H, NCH_2_); 2.71–2.54 (m, 4H, NCH_2_); 2.53–2.42 (m, 4H, NCH_2_); 2.41–2.34 (m, 2H, NCH_2_); 2.00–1.94 (m, 2H, CH_2_); 1.50–1.42 (m, 2H, CH_2_) ppm. ^13^C-NMR (100 MHz, CDCl_3_) δ: 161.97 (C); 161.20 (C); 157.81 (C); 155.85 (C); 143.40 (CH); 137.54 (C); 128.75 (CH); 128.60 (CH); 113.79 (CH); 113.16 (CH); 113.01 (CH); 112.62 (C); 101.50 (CH); 66.55 (CH_2_); 58.47 (CH_2_); 56.83 (CH_2_); 55.22 (OCH_3_); 53.49 (CH_2_); 53.01 (CH_2_); 49.55 (CH); 33.89 (CH_2_); 25.25 (CH_2_) ppm. ESI-HRMS (*m*/*z*) calculated for [M+H]^+^ ion species C_33_H_39_N_2_O_5_ = 543.2853, found 543.2859. Dihydrochloride: white solid; mp 206–209 °C.

*7-(3-(4-(4,4-Bis(4-methoxyphenyl)butyl)piperazin-1-yl)propoxy)-2H-chromen-2-one* **26**. Following the general procedure, compound **26** (0.040 g, yield: 57.4%) was synthesized as a yellow oil, starting from **39** [21] (0.050 g, 0.14 mmol) and **43** [20] (0.045 g, 0.17 mmol) in 5.0 mL of dry acetonitrile. Free base: chromatographic eluent: CH_2_Cl_2_/2-propanol/NH_4_OH 96:4:0.6. ^1^H-NMR (400 MHz, CDCl_3_) δ: 7.61 (d, *J* = 9.2 Hz, 1H, C*H*=CH); 7.34 (d, *J* = 8.4 Hz, 1H, CH arom.); 7.11 (d, *J* = 8.8 Hz, 4H, CH arom.); 6.83–6.70 (m, 6H, CH arom.); 6.23 (d, *J* = 9.2 Hz, 1H, C*H*=CH); 4.06 (t, *J* = 6.0 Hz, 2H, OCH_2_); 3.79 (t, *J* = 8.0 Hz, 1H, CH); 3.76 (s, 6H, OCH_3_); 2.63–2.26 (m, 12H, NCH_2_); 2.05–1.88 (m, 4H, CH_2_); 1.50–1.36 (m, 2H, CH_2_) ppm. ^13^C-NMR (100 MHz, CDCl_3_) δ: 162.27 (C); 161.19 (C); 157.83 (C); 155.91 (C); 143.40 (CH); 137.53 (C); 128.71 (CH); 128.60 (CH); 113.80 (CH); 113.01 (CH); 112.94 (CH); 112.47 (C); 101.40 (CH); 66.83 (CH_2_); 58.45 (CH_2_); 55.22 (OCH_3_); 54.82 (CH_2_); 53.02 (CH_2_); 49.54 (CH); 33.89 (CH_2_); 26.43 (CH_2_); 25.18 (CH_2_) ppm. ESI-HRMS (*m*/*z*) calculated for [M+H]^+^ ion species C_34_H_41_N_2_O_5_ = 557.3010, found 557.3006. Dihydrochloride: white solid; mp 196–199 °C.

*7-(4-(4-(4,4-Bis(4-methoxyphenyl)butyl)piperazin-1-yl)butoxy)-2H-chromen-2-one* **27**. Following the general procedure, compound **27** (0.040 g, yield: 62.6%) was synthesized as a pale-yellow oil, starting from **39** [21] (0.040 g, 0.11 mmol) and **44** (0.039 g, 0.13 mmol) in 5.0 mL of dry acetonitrile. Free base: chromatographic eluent: CH_2_Cl_2_/2-propanol/NH_4_OH 95:5:0.5. ^1^H-NMR (400 MHz, CDCl_3_) δ: 7.61 (d, *J* = 9.2 Hz, 1H, C*H*=CH); 7.34 (d, *J* = 8.4 Hz, 1H, CH arom.); 7.11 (d, *J* = 8.8 Hz, 4H, CH arom.); 6.81–6.78 (m, 6H, CH arom.); 6.23 (d, *J* = 9.2 Hz, 1H, C*H*=CH); 4.01 (t, *J* = 6.0 Hz, 2H, OCH_2_); 3.78 (t, *J* = 8.0 Hz, 1H, CH); 3.75 (s, 6H, OCH_3_); 2.66–2.33 (m, 12H, NCH_2_); 2.00–1.94 (m, 2H, CH_2_); 1.85–1.78 (m, 2H, CH_2_); 1.70–1.63 (m, 2H, CH_2_); 1.48–1.42 (m, 2H, CH_2_) ppm. ^13^C-NMR (100 MHz, CDCl_3_) δ: 162.29 (C); 161.28 (C); 157.80 (C); 155.91 (C); 143.46 (CH); 137.56 (C); 128.73 (CH); 128.60 (CH); 113.78 (CH); 112.97 (CH); 112.42 (C); 101.31 (CH); 68.33 (CH_2_); 58.53 (CH_2_); 58.07 (CH_2_); 55.23 (OCH_3_); 53.06 (CH_2_); 49.55 (CH); 33.93 (CH_2_); 26.98 (CH_2_); 25.30 (CH_2_); 23.28 (CH_2_) ppm. ESI-HRMS (*m*/*z*) calculated for [M+H]^+^ ion species C_35_H_43_N_2_O_5_ = 571.3166, found 571.3157. Dihydrochloride: white solid; mp 188–191 °C.

*7-(2-(4-(4,4-Bis(4-methoxyphenyl)butyl)piperazin-1-yl)ethoxy)-4-methyl-2H-chromen-2-one* **28**. Following the general procedure, compound **28** (0.070 g, yield: 74.0%) was synthesized as a pale-yellow oil, starting from **39** [21] (0.060 g, 0.17 mmol) and **45** (0.057 g, 0.20 mmol) in 5.0 mL of dry acetonitrile. Free base: chromatographic eluent: CH_2_Cl_2_/2-propanol/NH_4_OH 97:3:0.3. ^1^H-NMR (400 MHz, CDCl_3_) δ: 7.47 (d, *J* = 8.8 Hz, 1H, CH arom.); 7.11 (d, *J* = 8.8 Hz, 4H, CH arom.); 6.85 (dd, *J* = 8.8, 2.4 Hz, 1H, CH arom.); 6.82–6.76 (m, 5H, CH arom.); 6.12 (s, 1H, C*H*=C); 4.13 (t, *J* = 5.6 Hz, 2H, OCH_2_); 3.79 (t, *J* = 7.6 Hz, 1H, CH); 3.75 (s, 6H, OCH_3_); 2.82 (t, *J* = 5.6 Hz, 2H, NCH_2_); 2.73–2.51 (m, 4H, NCH_2_); 2.50–2.31 (m, 9H, CH_2_ and CH_3_); 2.00–1.95 (m, 2H, CH_2_); 1.50–1.38 (m, 2H, CH_2_) ppm. ^13^C-NMR (100 MHz, CDCl_3_) δ: 161.83 (C); 161.21 (C); 157.83 (C); 155.26 (C); 152.44 (C); 137.62 (C); 128.60 (CH); 125.48 (CH); 114.55 (C); 113.79 (CH); 113.66 (C); 112.67 (CH); 112.02 (CH); 101.56 (CH); 66.56 (CH_2_); 58.54 (CH_2_); 56.90 (CH_2_); 55.22 (OCH_3_); 53.68 (CH_2_); 53.11 (CH_2_); 49.59 (CH); 33.95 (CH_2_); 25.41 (CH_2_); 18.61 (CH_3_) ppm. ESI-HRMS (*m*/*z*) calculated for [M+H]^+^ ion species C_34_H_41_N_2_O_5_= 557.3010, found 557.3006. Dihydrochloride: white solid; mp 147–150 °C.

*7-(3-(4-(4,4-Bis(4-methoxyphenyl)butyl)piperazin-1-yl)propoxy)-4-methyl-2H-chromen-2-one* **29**. Following the general procedure, compound **29** (0.050 g, yield: 51.8%) was synthesized as a pale-yellow oil, starting from **39** [21] (0.060 g, 0.17 mmol) and **46** (0.059 g, 0.20 mmol) in 5.0 mL of dry acetonitrile. Free base: chromatographic eluent: CH_2_Cl_2_/2-propanol/NH_4_OH 95:5:0.5. ^1^H-NMR (400 MHz, CDCl_3_) δ: 7.46 (d, *J* = 8.8 Hz, 1H, CH arom.); 7.11 (d, *J* = 8.8 Hz, 4H, CH arom.); 6.84 (dd, *J* = 8.8, 2.4 Hz, 1H, CH arom.); 6.80–6.76 (m, 5H, CH arom.); 6.11 (s, 1H, C*H*=C); 4.05 (t, *J* = 5.6 Hz, 2H, OCH_2_); 3.79 (t, *J* = 7.6 Hz, 1H, CH); 3.75 (s, 6H, OCH_3_); 2.65–2.30 (m, 15H, CH_2_ and CH_3_); 2.00–1.95 (m, 4H, CH_2_); 1.47–1.40 (m, 2H, CH_2_) ppm. ^13^C-NMR (100 MHz, CDCl_3_) δ: 162.12 (C); 161.27 (C); 157.82 (C); 155.29 (C); 152.51 (C); 137.62 (C); 128.60 (CH); 125.46 (CH); 114.55 (C); 113.79 (CH); 113.49 (C); 112.63 (CH); 111.87 (CH); 101.43 (CH); 66.87 (CH_2_); 58.56 (CH_2_); 55.21 (OCH_3_); 54.90 (CH_2_); 53.20 (CH_2_); 49.57 (CH); 33.96 (CH_2_); 26.52 (CH_2_); 25.38 (CH_2_); 18.61 (CH_3_) ppm. ESI-HRMS (*m*/*z*) calculated for [M+H]^+^ ion species C_35_H_43_N_2_O_5_= 571.3166, found 571.3174. Dihydrochloride: white solid; mp 176–178 °C.

*7-(4-(4-(4,4-Bis(4-methoxyphenyl)butyl)piperazin-1-yl)butoxy)-4-methyl-2H-chromen-2-one* **30**. Following the general procedure, compound **30** (0.050 g, yield: 75.7%) was synthesized as a pale-yellow oil, starting from **39** [21] (0.040 g, 0.11 mmol) and **47** (0.042 g, 0.14 mmol) in 5.0 mL of dry acetonitrile. Free base: chromatographic eluent: CH_2_Cl_2_/CH_3_OH/NH_4_OH 95:5:0.5. ^1^H-NMR (400 MHz, CDCl_3_) δ: 7.46 (d, *J* = 8.8 Hz, 1H, CH arom.); 7.11 (d, *J* = 8.8 Hz, 4H, CH arom.); 6.87–6.75 (m, 6H, CH arom.); 6.11 (s, 1H, C*H*=C); 4.02 (t, *J* = 5.6 Hz, 2H, OCH_2_); 3.78 (t, *J* = 7.6 Hz, 1H, CH); 3.75 (s, 6H, OCH_3_); 2.50–2.40 (m, 13H, CH_2_ and CH_3_); 2.03–1.90 (m, 4H, CH_2_); 1.86–1.76 (m, 2H, CH_2_); 1.70–1.60 (m, 2H, CH_2_); 1.50–1.39 (m, 2H, CH_2_) ppm. ^13^C-NMR (100 MHz, CDCl_3_) δ: 162.12 (C); 161.35 (C); 157.80 (C); 155.30 (C); 152.57 (C); 137.61 (C); 128.60 (CH); 125.47 (CH); 113.77 (CH); 113.45 (C); 112.66 (CH); 111.85 (CH); 101.33 (CH); 68.32 (CH_2_); 58.59 (CH_2_); 58.14 (CH_2_); 55.22 (OCH_3_); 53.19 (CH_2_); 49.57 (CH); 33.97 (CH_2_); 27.03 (CH_2_); 25.40 (CH_2_); 23.35 (CH_2_); 18.65 (CH_3_) ppm. ESI-HRMS (*m*/*z*) calculated for [M+H]^+^ ion species C_36_H_45_N_2_O_5_ = 585.3323, found 585.3322. Dihydrochloride: white solid; mp 58–61 °C.

*6-(2-(4-(4,4-Bis(4-methoxyphenyl)butyl)piperazin-1-yl)ethoxy)-2H-chromen-2-one* **31**. Following the general procedure, compound **31** (0.040 g, yield: 65.2%) was synthesized as a pale-yellow oil, starting from **39** [21] (0.040 g, 0.11 mmol) and **48** (0.036 g, 0.14 mmol) in 5.0 mL of dry acetonitrile. Free base: chromatographic eluent: CH_2_Cl_2_/CH_3_OH/NH_4_OH 95:5:0.5. ^1^H-NMR (400 MHz, CDCl_3_) δ: 7.62 (d, *J* = 9.6 Hz, 1H, C*H*=CH); 7.24 (d, *J* = 8.8 Hz, 1H, CH arom); 7.12–7.08 (m, 5H, CH arom); 6.90 (d, *J* = 2.8 Hz, 1H, CH arom); 6.79 (d, *J* = 8.4 Hz, 4H, CH arom); 6.41 (d, *J* = 9.6 Hz, 1H, CH=C*H*); 4.10 (t, *J* = 6.0 Hz, 2H, OCH_2_); 3.79 (t, *J* = 8.0 Hz, 1H, CH); 3.75 (s, 6H, OCH_3_); 2.82 (t, *J* = 6.0 Hz, 2H, NCH_2_); 2.74–2.34 (m, 10H, CH_2_); 2.01–1.95 (m, 2H, CH_2_); 1.53–1.41 (m, 2H, CH_2_) ppm. ^13^C-NMR (100 MHz, CDCl_3_) δ: 160.94 (C); 157.83 (C); 155.22 (C); 148.54 (C); 143.18 (CH); 137.47 (C); 128.59 (CH); 120.00 (CH); 119.17 (C); 117.87 (CH); 117.11 (CH); 114.52 (C); 113.80 (CH); 111.00 (CH); 66.65 (CH_2_); 58.41 (CH_2_); 57.02 (CH_2_); 55.23 (OCH_3_); 53.30 (CH_2_); 52.95 (CH_2_); 49.52 (CH); 33.84 (CH_2_); 25.10 (CH_2_) ppm. ESI-HRMS (*m*/*z*) calculated for [M+H]^+^ ion species C_33_H_39_N_2_O_5_ = 543.2853, found 543.2845. Dihydrochloride: yellow solid; mp 238–241 °C.

*6-(3-(4-(4,4-Bis(4-methoxyphenyl)butyl)piperazin-1-yl)propoxy)-2H-chromen-2-one* **32**. Following the general procedure, compound **32** (0.050 g, yield: 79.5%) was synthesized as a pale-yellow oil, starting from **39** [21] (0.040 g, 0.11 mmol) and **49** (0.038 g, 0.14 mmol) in 5.0 mL of dry acetonitrile. Free base: chromatographic eluent: CH_2_Cl_2_/CH_3_OH/NH_4_OH 95:5:0.5. ^1^H-NMR (400 MHz, CDCl_3_) δ: 7.61 (d, *J* = 9.6 Hz, 1H, C*H*=CH); 7.23 (d, *J* = 9.2 Hz, 1H, CH arom); 7.11 (d, *J* = 8.4 Hz, 4H, CH arom); 7.08 (dd, *J* = 9.2, 2.8 Hz, 1H, CH arom); 6.89 (d, *J* = 2.8 Hz, 1H, CH arom); 6.79 (d, *J* = 8.4 Hz, 4H, CH arom); 6.39 (d, *J* = 9.6 Hz, 1H, CH=C*H*); 4.01 (t, *J* = 6.4 Hz, 2H, OCH_2_); 3.78 (t, *J* = 8.0 Hz, 1H, CH); 3.74 (s, 6H, OCH_3_); 2.68–2.32 (m, 12H, CH_2_); 2.00–1.94 (m, 4H, CH_2_); 1.48–1.40 (m, 2H, CH_2_) ppm. ^13^C-NMR (100 MHz, CDCl_3_) δ: 161.01 (C); 157.80 (C); 155.48 (C); 148.38 (C); 143.25 (CH); 137.55 (C); 128.60 (CH); 119.93 (CH); 119.16 (C); 117.80 (CH); 117.01 (CH); 114.55 (C); 113.78 (CH); 110.79 (CH); 66.96 (CH_2_); 58.54 (CH_2_); 55.23 (OCH_3_); 55.02 (CH_2_); 53.12 (CH_2_); 49.56 (CH); 33.92 (CH_2_); 26.65 (CH_2_); 25.33 (CH_2_) ppm. ESI-HRMS (*m*/*z*) calculated for [M+H]^+^ ion species C_34_H_41_N_2_O_5_ = 557.3010, found 557.3019. Dihydrochloride: yellow solid; mp 225–227 °C.

*6-(4-(4-(4,4-Bis(4-methoxyphenyl)butyl)piperazin-1-yl)butoxy)-2H-chromen-2-one* **33**. Following the general procedure, compound **33** (0.050 g, yield: 51.8%) was synthesized as a pale-yellow oil, starting from **39** [21] (0.060 g, 0.17 mmol) and **50** (0.060 g, 0.20 mmol) in 5.0 mL of dry acetonitrile. Free base: chromatographic eluent: CH_2_Cl_2_/2-propanol/NH_4_OH 95:5:0.5. TLC: CH_2_Cl_2_/CH_3_OH/NH_4_OH 95:5:0.5. ^1^H-NMR (400 MHz, CDCl_3_) δ: 7.62 (d, *J* = 9.6 Hz, 1H, C*H*=CH); 7.23 (d, *J* = 8.8 Hz, 1H, CH arom); 7.11 (d, *J* = 8.8 Hz, 4H, CH arom); 7.07 (dd, *J* = 8.8, 2.8 Hz, 1H, CH arom); 6.88 (d, *J* = 2.8 Hz, 1H, CH arom); 6.79 (d, *J* = 8.8 Hz, 4H, CH arom); 6.40 (d, *J* = 9.6 Hz, 1H, CH=C*H*); 3.97 (t, *J* = 6.0 Hz, 2H, OCH_2_); 3.78 (t, *J* = 8.0 Hz, 1H, CH); 3.74 (s, 6H, OCH_3_); 2.68–2.12 (m, 12H, CH_2_); 2.00–1.94 (m, 2H, CH_2_); 1.82–1.77 (m, 2H, CH_2_); 1.70–1.62 (m, 2H, CH_2_); 1.47–1.39 (m, 2H, CH_2_) ppm. ^13^C-NMR (100 MHz, CDCl_3_) δ: 161.02 (C); 157.80 (C); 155.50 (C); 148.36 (C); 143.25 (CH); 137.57 (C); 128.60 (CH); 119.89 (CH); 119. 16 (C); 117.83 (CH); 117.03 (CH); 114.52 (C); 113.77 (CH); 110.73 (CH); 68.43 (CH_2_); 58.57 (CH_2_); 58.17 (CH_2_); 55.23 (OCH_3_); 53.13 (CH_2_); 49.57 (CH); 33.94 (CH_2_); 27.20 (CH_2_); 25.37 (CH_2_); 23.39 (CH_2_) ppm. ESI-HRMS (*m*/*z*) calculated for [M+H]^+^ ion species C_35_H_43_N_2_O_5_ = 571.3166, found 571.3156. Dihydrochloride: yellow solid; mp 234–236 °C.

*6-(2-(4-(4,4-Bis(4-methoxyphenyl)butyl)piperazin-1-yl)ethoxy)-4-methyl-2H-chromen-2-one* **34**. Following the general procedure, compound **34** (0.070 g, yield: 63.8%) was synthesized as a pale-yellow oil, starting from **39** [21] (0.070 g, 0.20 mmol) and **51** (0.067 g, 0.24 mmol) in 5.0 mL of dry acetonitrile. Free base: chromatographic eluent: CH_2_Cl_2_/2-propanol/NH_4_OH 95:5:0.5. TLC: CH_2_Cl_2_/CH_3_OH/NH_4_OH 95:5:0.5. ^1^H-NMR (400 MHz, CDCl_3_) δ: 7.23 (d, *J* = 8.8 Hz, 1H, CH arom); 7.12–7.07 (m, 5H, CH arom); 7.03 (d, *J* = 2.8 Hz, 1H, CH arom); 6.79 (d, *J* = 8.8 Hz, 4H, CH arom); 6.27 (s, 1H, C*H*=C); 4.11 (t, *J* = 6.0 Hz, 2H, OCH_2_); 3.78 (t, *J* = 8.0 Hz, 1H, CH); 3.74 (s, 6H, OCH_3_); 2.81 (t, *J* = 6.0 Hz, 2H, NCH_2_); 2.70–2.52 (m, 4H, NCH_2_); 2.51–2.32 (m, 9H, CH_2_ and CH_3_); 2.00–1.94 (m, 2H, CH_2_); 1.47–1.42 (m, 2H, CH_2_) ppm. ^13^C-NMR (100 MHz, CDCl_3_) δ: 160.94 (C); 157.81 (C); 155.16 (C); 151.97 (C); 147.95 (C); 137.58 (C); 128.59 (CH); 120.44 (C); 119.24 (CH); 117.92 (CH); 115.48 (CH); 114.54 (C); 113.79 (CH); 108.67 (CH); 66.62 (CH_2_); 58.53 (CH_2_); 57.15 (CH_2_); 55.22 (OCH_3_); 53.64 (CH_2_); 53.08 (CH_2_); 49.56 (CH); 33.92 (CH_2_); 25.36 (CH_2_); 18.72 (CH_3_) ppm. ESI-HRMS (*m*/*z*) calculated for [M+H]^+^ ion species C_34_H_41_N_2_O_5_ = 557.3010, found 557.3017. Dihydrochloride: yellow solid; mp 218–221 °C.

*6-(3-(4-(4,4-Bis(4-methoxyphenyl)butyl)piperazin-1-yl)propoxy)-4-methyl-2H-chromen-2-one* **35**. Following the general procedure, compound **35** (0.070 g, yield: 72.6%) was synthesized as a pale-yellow oil, starting from **39** [21] (0.060 g, 0.17 mmol) and **52** (0.060 g, 0.20 mmol) in 5.0 mL of dry acetonitrile. Free base: chromatographic eluent: CH_2_Cl_2_/2-propanol/NH_4_OH 95:5:0.5. TLC: CH_2_Cl_2_/CH_3_OH/NH_4_OH 95:5:0.5. ^1^H-NMR (400 MHz, CDCl_3_) δ: 7.22 (d, *J* = 9.2 Hz, 1H, CH arom); 7.12–7.06 (m, 5H, CH arom); 6.99 (d, *J* = 2.8 Hz, 1H, CH arom); 6.79 (d, *J* = 8.8 Hz, 4H, CH arom); 6.26 (s, 1H, C*H*=C); 4.03 (t, *J* = 6.4 Hz, 2H, OCH_2_); 3.78 (t, *J* = 8.0 Hz, 1H, CH); 3.74 (s, 6H, OCH_3_); 2.55–2.31 (m, 15H, CH_2_ and CH_3_); 2.00–1.94 (m, 4H, CH_2_); 1.47–1.39 (m, 2H, CH_2_) ppm. ^13^C-NMR (100 MHz, CDCl_3_) δ: 160.95 (C); 157.81 (C); 155.40 (C); 152.01 (C); 147.82 (C); 137.60 (C); 128.60 (CH); 120.43 (C); 119.13 (CH); 117.87 (CH); 115.42 (CH); 114.56 (C); 113.78 (CH); 108.53 (CH); 67.05 (CH_2_); 58.57 (CH_2_); 55.21 (OCH_3_); 55.07 (CH_2_); 53.26 (CH_2_); 53.18 (CH_2_); 49.57 (CH); 33.96 (CH_2_); 26.74 (CH_2_); 25.39 (CH_2_); 18.71 (CH_3_) ppm. ESI-HRMS (*m*/*z*) calculated for [M+H]^+^ ion species C_35_H_43_N_2_O_5_ = 571.3166, found 571.3163. Dihydrochloride: yellow solid; mp 236–239 °C.

*6-(4-(4-(4,4-Bis(4-methoxyphenyl)butyl)piperazin-1-yl)butoxy)-4-methyl-2H-chromen-2-one* **36**. Following the general procedure, compound **36** (0.030 g, yield: 45.4%) was synthesized as a pale-yellow oil, starting from **39** [21] (0.040 g, 0.11 mmol) and **53** (0.042 g, 0.14 mmol) in 3.4 mL of dry acetonitrile. Free base: chromatographic eluent: CH_2_Cl_2_/2-propanol/NH_4_OH 95:5:0.5. TLC: CH_2_Cl_2_/CH_3_OH/NH_4_OH 95:5:0.5. ^1^H-NMR (400 MHz, CDCl_3_) δ: 7.25 (d, *J* = 9.2 Hz, 1H, CH arom); 7.12–7.08 (m, 5H, CH arom); 6.99 (d, *J* = 2.8 Hz, 1H, CH arom); 6.80 (d, *J* = 8.8 Hz, 4H, CH arom); 6.29 (s, 1H, C*H*=C); 4.00 (t, *J* = 6.4 Hz, 2H, OCH_2_); 3.78 (t, *J* = 6.4 Hz, 1H, CH); 3.76 (s, 6H, OCH_3_); 2.66–2.41 (m, 15H, CH_2_ and CH_3_); 2.00–1.95 (m, 2H, CH_2_); 1.85–1.78 (m, 2H, CH_2_); 1.75–1.69 (m, 2H, CH_2_); 1.50–1.42 (m, 2H, CH_2_) ppm. ^13^C-NMR (100 MHz, CDCl_3_) δ: 157.84 (C); 155.36 (C); 151.97 (C); 147.86 (C); 137.43 (C); 128.57 (CH); 120.46 (C); 119.06 (CH); 117.92 (CH); 115.47 (CH); 113.81 (CH); 108.46 (CH); 68.36 (CH_2_); 58.20 (CH_2_); 57.87 (CH_2_); 55.22 (OCH_3_); 52.51 (CH_2_); 49.47 (CH); 33.80 (CH_2_); 27.18 (CH_2_); 24.91 (CH_2_); 23.05 (CH_2_); 18.72 (CH_3_) ppm. ESI-HRMS (*m*/*z*) calculated for [M+H]^+^ ion species C_36_H_45_N_2_O_5_ = 585.3323, found 585.3313. Dihydrochloride: yellow solid; mp 240–242 °C.

#### 4.1.2. 7-Hydroxy-4-methyl-2H-chromen-2-one **40** [30]

Following the procedure described by Narella et al. [30], a solution of resorcinol (0.40 g, 3.63 mmol) in ethyl acetoacetate (0.5 mL, 3.99 mmol) was added dropwise at 0 °C in 4.0 mL of concentrated H_2_SO_4_. The mixture was stirred at rt for 15 min, and then cold water was added. A solid precipitated, which was filtrated under reduced pressure and dried, obtaining **40** (0.51 g, yield 79.9%) as a pale-yellow solid. TLC: CH_2_Cl_2_/CH_3_OH 95:5. Mp: 182–185 °C. ^1^H-NMR (400 MHz, DMSO-d_6_) δ: 7.53 (d, *J* = 8.8 Hz, 1H, CH arom.); 6.75 (d, *J* = 8.8 Hz, 1H, CH arom.); 6.64 (s, 1H, CH arom.); 6.05 (s, 1H, C*H*=C); 2.29 (s, 3H, CH_3_) ppm.

#### 4.1.3. 6-Hydroxy-4-methyl-2H-chromen-2-one **41** [31]

Following the procedure described by Narella et al. [30], with slight modifications, 13.0 mL of concentrated H_2_SO_4_ was added dropwise at 0 °C to a suspension of hydroquinone (2.50 g, 22.73 mmol) in ethyl acetoacetate (3.47 mL, 27.27 mmol). The mixture was stirred at rt for 4 days, and then cold water was added. A solid precipitated, which was filtrated under reduced pressure and dried, obtaining **41** (0.92 g, yield 23.0%) as a yellow solid. TLC: CH_2_Cl_2_/CH_3_OH 95:5. Mp: 235–237 °C. ^1^H-NMR (400 MHz, DMSO-d_6_) δ: 9.72 (bs, 1H, OH); 7.22 (d, *J* = 9.6 Hz, 1H, CH arom); 7.01–6.98 (m, 2H, CH arom); 6.34 (s, 1H, C*H*=C); 2.35 (s, 3H, CH_3_) ppm.

#### 4.1.4. General Procedure for the Synthesis of the Bromoalkoxy-2H-chromen-2-ones **42** and **44**–**53**

To a solution of the proper hydroxy-2*H*-chromen-2-one (1 equiv.) in acetone or acetonitrile, K_2_CO_3_ (3 equiv.) and the proper dibromoalkane (5 equiv.) were added. The reaction was refluxed overnight, then it was cooled to rt, and the solvent was removed under reduced pressure. The mixture was dissolved in CH_2_Cl_2_, and the organic phase was washed twice with water and with a saturated solution of NaHCO_3_, then dried over Na_2_SO_4_ and concentrated under vacuum. Finally, the residue was washed three times with Et_2_O, yielding the desired compound as a pure solid.

*7-(2-Bromoethoxy)-2H-chromen-2-one* **42** [25]. Following the general procedure, compound **42** (0.30 g, yield: 90.7%) was synthesized as a white solid, from 7-hydroxy-2*H*-chromen-2-one (0.20 g, 1.23 mmol) and 1,2-dibromoethane (0.61 mL, 6.50 mmol) in 15.0 mL of acetone. TLC: CH_2_Cl_2_/CH_3_OH 90:10. Mp: 134–136 °C. ^1^H-NMR (400 MHz, CDCl_3_) δ: 7.63 (d, *J* = 9.2 Hz, 1H, C*H*=CH); 7.38 (d, *J* = 8.4 Hz, 1H, CH arom.); 6.86 (dd, *J* = 8.4, 2.4 Hz, 1H, CH arom.); 6.80 (d, *J* = 2.4 Hz, 1H, CH arom.); 6.26 (d, *J* = 9.2 Hz, 1H, C*H*=CH); 4.35 (t, *J* = 6.4 Hz, 2H, OCH_2_); 3.67 (t, *J* = 6.4 Hz, 2H, CH_2_Br) ppm.

*7-(4-Bromobutoxy)-2H-chromen-2-one* **44** [25]. Following the general procedure, compound **44** (0.33 g, yield: 86.0%) was synthesized as a white solid, from 7-hydroxy-2*H*-chromen-2-one (0.21 g, 1.30 mmol) and 1,4-dibromobutane (0.77 mL, 6.50 mmol) in 15.0 mL of acetone. TLC: CH_2_Cl_2_/CH_3_OH 90:10. Mp: 105–107 °C. ^1^H-NMR (400 MHz, CDCl_3_) δ: 7.62 (d, *J* = 9.2 Hz, 1H, C*H*=CH); 7.36 (d, *J* = 8.4 Hz, 1H, CH arom.); 6.82 (dd, *J* = 8.4, 2.4 Hz, 1H, CH arom.); 6.78 (d, *J* = 2.4 Hz, 1H, CH arom.); 6.24 (d, *J* = 9.2 Hz, 1H, C*H*=CH); 4.04 (t, *J* = 6.0 Hz, 2H, OCH_2_); 3.49 (t, *J* = 6.0 Hz, 2H, CH_2_Br); 2.10–2.03 (m, 2H, CH_2_); 2.02–1.96 (m, 2H, CH_2_) ppm.

*7-(2-Bromoethoxy)-4-methyl-2H-chromen-2-one* **45** [26]. Following the general procedure, compound **45** (0.13 g, yield: 62.4%) was synthesized as a white solid, from **40** (0.13 g, 0.74 mmol) and 1,2-dibromoethane (0.51 mL, 5.95 mmol) in 10.0 mL of acetone. TLC: CH_2_Cl_2_/CH_3_OH 90:10. Mp: 139–141 °C. ^1^H-NMR (400 MHz, CDCl_3_) δ: 7.51 (d, *J* = 8.8 Hz, 1H, CH arom.); 6.91 (dd, *J* = 8.8, 2.4 Hz, 1H, CH arom.); 6.81 (d, *J* = 2.4 Hz, 1H, CH arom.); 6.15 (s, 1H, C*H*=C); 4.35 (t, *J* = 6.0 Hz, 2H, OCH_2_); 3.61 (t, *J* = 6.0 Hz, 2H, CH_2_Br); 2.39 (s, 3H, CH_3_) ppm.

*7-(3-Bromopropoxy)-4-methyl-2H-chromen-2-one* **46** [26]. Following the general procedure, compound **46** (0.19 g, yield: 74.8%) was synthesized as a white solid, from **40** (0.15 g, 0.85 mmol) and 1,3-dibromopropane (0.43 mL, 4.26 mmol) in 11.0 mL of acetone. TLC: CH_2_Cl_2_/CH_3_OH 90:10. Mp: 75–78 °C. ^1^H-NMR (400 MHz, CDCl_3_) δ: 7.50 (d, *J* = 8.8 Hz, 1H, CH arom.); 6.86 (dd, *J* = 8.8, 2.4 Hz, 1H, CH arom.); 6.82 (d, *J* = 2.4 Hz, 1H, CH arom.); 6.13 (s, 1H, C*H*=C); 4.23 (t, *J* = 6.0 Hz, 2H, OCH_2_); 3.61 (t, *J* = 6.0 Hz, 2H, CH_2_Br); 2.39 (s, 3H, CH_3_); 2.36–2.32 (m, 2H, CH_2_) ppm.

*7-(4-Bromobutoxy)-4-methyl-2H-chromen-2-one* **47** [26]. Following the general procedure, compound **47** (0.16 g, yield: 76.3%) was synthesized as a white solid, from **40** (0.12 g, 0.68 mmol) and 1,4-dibromobutane (0.40 mL, 3.40 mmol) in 9.0 mL of acetone. TLC: CH_2_Cl_2_/CH_3_OH 90:10. Mp: 63–65 °C. ^1^H-NMR (400 MHz, CDCl_3_) δ: 7.49 (d, *J* = 8.4 Hz, 1H, CH arom.); 6.85 (dd, *J* = 8.8, 2.4 Hz, 1H, CH arom.); 6.79 (d, *J* = 2.4 Hz, 1H, CH arom.); 6.13 (s, 1H, C*H*=C); 4.05 (t, *J* = 6.0 Hz, 2H, OCH_2_); 3.49 (t, *J* = 6.0 Hz, 2H, CH_2_Br); 2.39 (s, 3H, CH_3_); 2.09–2.03 (m, 2H, CH_2_); 2.02–1.96 (m, 2H, CH_2_) ppm.

*6-(2-Bromoethoxy)-2H-chromen-2-one* **48** [27]. Following the general procedure, compound **48** (0.26 g, yield: 64.7%) was synthesized as a white solid, from 6-hydroxy-2*H*-chromen-2-one (0.25 g, 1.51 mmol) and 1,2-dibromoethane (0.66 mL, 7.72 mmol) in 10.0 mL of acetonitrile. TLC: CH_2_Cl_2_/CH_3_OH 95:5. Mp: 96–98 °C. ^1^H-NMR (400 MHz, CDCl_3_) δ: 7.64 (d, *J* = 9.6 Hz, 1H, C*H*=CH); 7.27 (d, *J* = 9.2 Hz, 1H, CH arom.); 7.13 (dd, *J* = 9.2, 2.8 Hz, 1H, CH arom.); 6.95 (d, *J* = 2.8 Hz, 1H, CH arom.); 6.44 (d, *J* = 9.6 Hz, 1H, C*H*=CH); 4.32 (t, *J* = 5.6 Hz, 2H, OCH_2_); 3.66 (t, *J* = 5.6 Hz, 2H, CH_2_Br) ppm.

*6-(3-Bromopropoxy)-2H-chromen-2-one* **49** [28]. Following the general procedure, compound **49** (0.15 g, yield: 74.6%) was synthesized as a white solid, from 6-hydroxy-2*H*-chromen-2-one (0.12 g, 0.71 mmol) and 1,3-dibromopropane (0.36 mL, 3.57 mmol) in 10.0 mL of acetonitrile. TLC: CH_2_Cl_2_/CH_3_OH 95:5. Mp: 97–99 °C. ^1^H-NMR (400 MHz, CDCl_3_) δ: 7.65 (d, *J* = 9.6 Hz, 1H, C*H*=CH); 7.27 (d, *J* = 9.2 Hz, 1H, CH arom.); 7.11 (dd, *J* = 9.2, 2.8 Hz, 1H, CH arom.); 6.93 (d, *J* = 2.8 Hz, 1H, CH arom.); 6.43 (d, *J* = 9.6 Hz, 1H, C*H*=CH); 4.14 (t, *J* = 5.6 Hz, 2H, OCH_2_); 3.62 (t, *J* = 5.6 Hz, 2H, CH_2_Br); 2.37–2.31 (m, 2H, CH_2_) ppm.

*6-(4-Bromobutoxy)-2H-chromen-2-one* **50** [28]. Following the general procedure, compound **50** (0.20 g, yield: 87.6%) was synthesized as a white solid, from 6-hydroxy-2*H*-chromen-2-one (0.13 g, 0.77 mmol) and 1,4-dibromobutane (0.46 mL, 3.85 mmol) in 10.0 mL of acetonitrile. TLC: CH_2_Cl_2_/CH_3_OH 95:5. Mp: 114–116 °C. ^1^H-NMR (400 MHz, CDCl_3_) δ: 7.64 (d, *J* = 9.6 Hz, 1H, C*H*=CH); 7.26 (d, *J* = 9.2 Hz, 1H, CH arom); 7.09 (dd, *J* = 9.2, 2.8 Hz, 1H, CH arom); 6.90 (d, *J* = 2.8 Hz, 1H, CH arom); 6.43 (d, *J* = 9.6 Hz, 1H, CH=C*H*); 4.02 (t, *J* = 6.4 Hz, 2H, CH_2_O); 3.50 (t, *J* = 6.4 Hz, 2H, CH_2_Br); 2.10–2.06 (m, 2H, CH_2_); 2.00–1.95 (m, 2H, CH_2_) ppm.

*6-(2-Bromoethoxy)-4-methyl-2H-chromen-2-one* **51** [29]. Following the general procedure, compound **51** (0.34 g, yield: 64.2%) was synthesized as a yellow solid, from **41** (0.33 g, 1.87 mmol) and 1,2-dibromoethane (0.80 mL, 9.37 mmol) in 50.0 mL of acetone. TLC: CH_2_Cl_2_/CH_3_OH 95:5. Mp: 133–135 °C. ^1^H-NMR (400 MHz, CDCl_3_) δ: 7.26 (d, *J* = 9.2 Hz, 1H, CH arom); 7.14 (dd, *J* = 9.2, 2.8 Hz, 1H, CH arom); 7.07 (d, *J* = 2.8 Hz, 1H, CH arom); 6.31 (s, 1H, C*H*=C); 4.34 (t, *J* = 6.0 Hz, 2H, CH_2_O); 3.66 (t, *J* = 6.0 Hz, 2H, CH_2_Br); 2.42 (s, 3H, CH_3_) ppm.

*6-(3-Bromopropoxy)-4-methyl-2H-chromen-2-one* **52** [29]. Following the general procedure, compound **52** (0.53 g, yield: 96.0%) was synthesized as a white solid, from **41** (0.33 g, 1.87 mmol) and 1,3-dibromopropane (0.95 mL, 9.37 mmol) in 50.0 mL of acetone. TLC: CH_2_Cl_2_/CH_3_OH 95:5. Mp: 90–92 °C. ^1^H-NMR (400 MHz, CDCl_3_) δ: 7.27 (d, *J* = 8.8 Hz, 1H, CH arom); 7.12 (dd, *J* = 8.8, 2.8 Hz, 1H, CH arom); 7.04 (d, *J* = 2.8 Hz, 1H, CH arom); 6.30 (s, 1H, C*H*=C); 4.16 (t, *J* = 6.0 Hz, 2H, CH_2_O); 3.63 (t, *J* = 6.0 Hz, 2H, CH_2_Br); 2.40 (s, 3H, CH_3_); 2.36–2.32 (m, 2H, CH_2_) ppm.

*6-(4-Bromobutoxy)-4-methyl-2H-chromen-2-one* **53** [29]. Following the general procedure, compound **53** (0.53 g, yield: 91.1%) was synthesized as a white solid, from **41** (0.33 g, 1.87 mmol) and 1,4-dibromobutane (1.12 mL, 9.37 mmol) in 50.0 mL of acetone. TLC: CH_2_Cl_2_/CH_3_OH 95:5. Mp: 100–102 °C. ^1^H-NMR (400 MHz, CDCl_3_) δ: 7.26 (d, *J* = 9.2 Hz, 1H, CH arom); 7.09 (dd, *J* = 9.2, 2.8 Hz, 1H, CH arom); 7.01 (d, *J* = 2.8 Hz, 1H, CH arom); 6.30 (s, 1H, C*H*=C); 4.04 (t, *J* = 6.4 Hz, 2H, CH_2_O); 3.50 (t, *J* = 6.4 Hz, 2H, CH_2_Br); 2.42 (s, 3H, CH_3_); 2.11–2.07 (m, 2H, CH_2_); 2.01–1.96 (m, 2H, CH_2_) ppm.

### 4.2. Biology and Enzymology

#### 4.2.1. CA Inhibition Assay

An SX.18MV-R Applied Photophysics (Oxford, UK) stopped-flow instrument has been used to assay the catalytic/inhibition of various CA isozymes [32]. Phenol Red (at a concentration of 0.2 mM) has been used as an indicator, working at the absorbance maximum of 557 nm, with 10 mM Hepes (pH 7.4) as buffer, 0.1 M Na_2_SO_4_ or NaClO_4_ (for maintaining constant the ionic strength; these anions are not inhibitory in the used concentration), following the CA-catalyzed CO_2_ hydration reaction for a period of 5–10 s. Saturated CO_2_ solutions in water at 25 °C were used as substrate. Stock solutions of inhibitors were prepared at a concentration of 10 μM (in DMSO-water 1:1, *v*/*v*) and dilutions up to 0.01 nM were carried out with the assay buffer mentioned above. At least 7 different inhibitor concentrations have been used for measuring the inhibition constant. Inhibitor and enzyme solutions were preincubated together for 6 h at room temperature prior to assay in order to allow for the formation of the E-I complex. Triplicate experiments were conducted for each inhibitor concentration, and the values reported throughout the paper are the mean of such results. The inhibition constants were obtained by non-linear least-squares methods using the Cheng–Prusoff equation, as reported earlier [53], and represent the mean from at least three different determinations. All CA isozymes used here were recombinant proteins obtained as reported earlier by our group and their concentrations in the assay system were 5.6–12 nM [54].

#### 4.2.2. Cell Lines and Cultures

The K562 leukemia cells were derived from a patient with chronic myelogenous leukemia [34] and the P-gp over-expressing K562/DOX cells were obtained from Prof. J. P. Marie (Hospital Hotel-Dieu, Paris, France). These cells were cultured in RPMI 1640 medium supplemented with 10% fetal calf serum (FCS; GIBCO, Grand Island, NY, USA) at 37 °C in a humidified incubator with 5% CO_2_. To maintain the resistance, every month, resistant cells were cultured for three days with 400 nM doxorubicin.

Human chemosensitive colon cancer HT29 cells and lung cancer A549 cells were purchased from ATCC (Manassas, VA, USA). Human HT29/DOX and A549/DOX were generated by stepwise selection in a medium with increasing concentration of doxorubicin, as reported in the literature [20], and maintained in a culture medium with a final concentration of 200 nM and 100 nM doxorubicin, respectively. All cell lines were authenticated by microsatellite analysis, using the PowerPlex kit (Promega Corporation, Madison, WI, USA; last authentication: January 2022). Cells were maintained in media supplemented with 10% *v*/*v* fetal bovine serum, 1% *v*/*v* penicillin-streptomycin, and 1% *v*/*v* L-glutamine.

MDCK-MDR1, MDCK-MRP1 and MDCK-BCRP cells are a gift of Prof. P. Borst, NKI-AVL Institute, Amsterdam, The Netherlands. Cells were grown in DMEM high glucose supplemented with 10% fetal bovine serum, 2 mM glutamine, 100 U/mL penicillin, and 100 µg/mL streptomycin in a humidified incubator at 37 °C with a 5% CO_2_ atmosphere. Cell culture reagents were purchased from Celbio s.r.l. (Milano, Italy). CulturePlate 96/wells plates were purchased from PerkinElmer Life Science (Waltham, MA, USA) and Falcon (BD Biosciences, Bedford, MA, USA). Calcein-AM and Hoechst 33342 (bisBenzimide H 33342 trihydrochloride) were obtained from Sigma-Aldrich (Milan, Italy). The other reagents were purchased from Sigma Merck Millipore.

#### 4.2.3. Drugs and Chemicals

Doxorubicin hydrochloride, verapamil hydrochloride, dimethylsulphoxide (DMSO) and 3-(4,5-dimethylthiazolyl-2)-2,5-diphenyl tetrazolium bromide (MTT) were purchased from Sigma-Aldrich (Milan, Italy). Stock solutions of the tested compounds as hydrochloride salts were prepared in DMSO at 10^−2^ M. Stock solutions of doxorubicin hydrochloride and verapamil hydrochloride were prepared in water at 10^−2^ M. All the stock solutions were then diluted with complete medium to obtain the 10× desired final maximum test concentrations.

#### 4.2.4. Intrinsic Cytotoxicity

The intrinsic toxicity of the studied compounds was determined by an MTT assay [35]. K562 and K562/DOX were exposed for 72 h with compounds at 1 and 3 µM concentrations while HT29, A549, HT29/DOX and A549/DOX cells were incubated for 48 h with compounds used at different concentrations (from 10 nM to 50 µM). The percentage of growth compared to the untreated control was transformed into histograms with the GraphPad Prism 5 program.

#### 4.2.5. Co-Administration Assays in K562/DOX, HT29, A549, HT29/DOX and A549/DOX Cells

K562/DOX cells were incubated for 72 h with different concentrations of doxorubicin in combination with 1 or 3 µM of all compounds. IC_50_ was the concentration killing 50% of cells and was determined graphically from relative survival curves obtained by GraphPad Prism 5 software (GraphPad, San Diego, CA, USA). HT29, A549, HT29/DOX and A549/DOX cells were incubated for 48 h with 5 µM doxorubicin, alone or in combination with 1 or 3 µM of selected compounds. Cell viability of the cell lines was measured by the MTT assay, using a Synergy HT microplate spectrofluorometer (Bio-Tek Instruments, Winooski, VT, USA). The absorbance of untreated cells was considered 100%; results were expressed as a percentage of viable treated cells versus the control untreated cells [51].

#### 4.2.6. Intracellular Doxorubicin Accumulation in HT29, A549, HT29/DOX and A549/DOX Cells

Intracellular doxorubicin was measured in 10,000 cells seeded into 96-well plates and incubated for 24 h with 5 µM doxorubicin with or without 1 µM and 3 µM of each compound. The intracellular drug content was measured fluorimetrically as detailed previously [51] using a Synergy HTX 96-well plate reader. The results were expressed as nmol doxorubicin/mg cell proteins based on the titration curve previously prepared.

#### 4.2.7. Calcein-AM Experiment

Experiments were performed as previously described [51]. MDCK-MDR1 and MDCK-MRP1 cells (30,000 cells per well) were seeded in a 96-well black CulturePlate in 100 µL of medium and allowed to grow overnight in a humidified atmosphere of 5% CO_2_ at 37 °C. Tested compounds (100 µL) in a final concentration ranging between 0.01 nM and 100 µM, solubilized in culture medium, were added to the monolayers. After 30 min in a humidified atmosphere of 5% CO_2_ at 37 °C, 100 µL of Calcein-AM in phosphate-buffered saline (PBS) was added to obtain a final concentration of 2.5 µM and the plate was incubated for 30 min. All wells were washed 3 times with 100 µL ice-cold PBS and read with Victor3 (PerkinElmer) after adding 100 µL of cold PBS. Excitation and emission wavelengths of 485 nm and 535 nm, respectively, were used. In these experimental conditions, cell Calcein-AM accumulation in the absence and in the presence of each tested compound was evaluated and compared to the basal level of fluorescence derived from untreated cells. The increase in fluorescence from the baseline level was measured in the treated wells. EC_50_ values were determined by fitting the rate of fluorescence increase versus log[dose].

#### 4.2.8. Hoechst 33342 Experiment

These experiments were conducted as previously described [51]. MDCK-BCRP cells (30,000 cells per well) were seeded in a black CulturePlate 96/well in 100 µL of medium and allowed to grow overnight in a humidified atmosphere of 5% CO_2_ at 37 °C. Tested compounds (100 µL) in a final concentration ranging between 0.01 nM and 100 µM, solubilized in culture medium, were added to the monolayers. After 30 min in a humidified atmosphere of 5% CO_2_ at 37 °C, 100 µL of Hoechst 33342 at the final concentration of 8 µM in PBS was added and the plate was incubated for 30 min. The supernatants were drained, and the cells were fixed for 20 min under protection from light using 100 µL per well of a 4% PFA solution. All wells were washed 3 times with 100 µL ice-cold PBS and read with Victor3 (PerkinElmer) after adding 100 µL of cold PBS. Excitation and emission wavelengths of 340/35 nm and 485/20 nm, respectively, were used. In these experimental conditions, the baseline fluorescence level of Hoechst 33342 was estimated using untreated cells and compared with the fluorescence emissions of cells treated with the tested compounds. EC_50_ values were determined by plotting the percent increase in fluorescence versus log[dose].

#### 4.2.9. Oxidative Stress Measurement

Selected compounds have been tested for their cytotoxic activity at 24 h on HT29/DOX and A549/DOX cells and on the parental HT29 and A549 cells by MTT assay. To evaluate the mechanisms involved in CS, reactive oxygen species (ROS) were measured using the fluorescent probe 5-(and-6)-chloromethyl-2′,7′-dichlorodihydro-fluorescein diacetate-acetoxymethyl ester (DCFDA-AM), as previously reported [55]. The results were expressed as nmol/mg cellular proteins. The amount of TBARS, as an index of lipoperoxidation, was measured with the Lipid Peroxidation (4-HNE) Assay Kit (Abcam, Cambridge, UK) that evaluates the 4-hydroxy-nonenale, one of the TBARS Results were expressed as nmoles/mg mitochondrial protein.

#### 4.2.10. P-gp ATPase Activity

Plasma-membrane vesicles enriched with P-gp were prepared by sequential centrifugation as detailed previously [56]. The ATPase activity of immunopurified P-gp was evaluated spectrophotometrically by measuring the absorbance of the phosphate hydrolyzed from ATP at 620 nm, using a Synergy HT Multi-Detection Microplate Reader (Bio-Tek Instruments). The absorbance was converted into μmol hydrolyzed phosphate/min/mg proteins, according to the titration curve previously prepared.

#### 4.2.11. Membrane Fluidity

Membrane fluidity was measured fluorometrically using the Membrane Fluidity Kit, Abcam, according to the manufacturer’s instructions.

#### 4.2.12. Statistical Analysis

All data in the tables and figures are provided as means ± SEM (Table 2) or SD. The results were analyzed by an ANOVA and Dunnett’s test, using Graph-Pad Prism (Graph-Pad software, San Diego, CA, USA).

## Figures and Tables

**Table 1:** Inhibitory activity on hCA I, II, IX and XII isoforms and doxorubicin cytotoxicity enhancement effect in K562/DOX cells of compounds **1**–**36** and of the two reference compounds acetazolamide (AAZ) and verapamil (Ver).

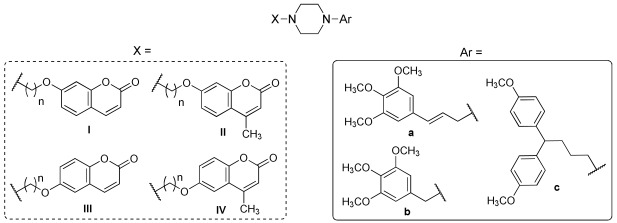									
				K_I_ (nM) ^1,2^				RF ^3^	
Cmpd	n	X	Ar	CA I	CA II	CA IX	CA XII	1 µM	3 µM
**1**	2	I	a	>10,000	>10,000	51.3	13.0	1.6	2.3
**2**	3	I	a	>10,000	>10,000	62.6	29.4	3.8	4.5
**3**	4	I	a	>10,000	>10,000	82.0	34.6	3.1	3.7
**4**	2	II	a	>10,000	>10,000	25.2	32.8	5.7	15.8
**5**	3	II	a	>10,000	>10,000	34.7	27.2	5.5	27.6
**6**	4	II	a	>10,000	>10,000	58.9	44.8	6.3	6.7
**7**	2	III	a	>10,000	>10,000	51.0	29.5	1.1	1.0
**8**	3	III	a	>10,000	>10,000	40.7	22.9	2.0	2.0
**9**	4	III	a	>10,000	>10,000	22.1	7.3	2.5	1.3
**10**	2	IV	a	>10,000	>10,000	360	422	1.0	1.4
**11**	3	IV	a	>10,000	>10,000	302	441	2.4	4.1
**12**	4	IV	a	>10,000	>10,000	104	140	1.9	2.7
**13**	2	I	b	>10,000	>10,000	23.7	6.2	1.0	1.9
**14**	3	I	b	>10,000	>10,000	40.1	13.6	1.0	3.4
**15**	4	I	b	>10,000	>10,000	29.0	42.9	1.2	2.3
**16**	2	II	b	>10,000	>10,000	8.2	51.9	1.0	1.4
**17**	3	II	b	>10,000	>10,000	16.1	60.8	2.6	2.1
**18**	4	II	b	>10,000	>10,000	28.5	72.1	1.9	3.2
**19**	2	III	b	>10,000	>10,000	84.7	5.8	1.4	2.2
**20**	3	III	b	>10,000	>10,000	72.0	22.4	2.3	2.4
**21**	4	III	b	>10,000	>10,000	50.3	15.9	3.9	4.1
**22**	2	IV	b	>10,000	>10,000	409	229	2.8	2.7
**23**	3	IV	b	>10,000	>10,000	242	304	1.6	1.8
**24**	4	IV	b	>10,000	>10,000	205	99.5	1.4	2.5
**25**	2	I	c	>10,000	>10,000	113	84.7	5.9	21.1
**26**	3	I	c	>10,000	>10,000	153	115	5.5	17.3
**27**	4	I	c	>10,000	>10,000	145	92.1	8.1	90.5
**28**	2	II	c	>10,000	>10,000	135	103	16.2	32.0
**29**	3	II	c	>10,000	>10,000	149	91.5	34.3	38.7
**30**	4	II	c	>10,000	>10,000	124	82.4	7.7	25.5
**31**	2	III	c	>10,000	>10,000	115	90.7	3.0	11.4
**32**	3	III	c	>10,000	>10,000	89.6	71.5	3.6	7.9
**33**	4	III	c	>10,000	>10,000	74.6	46.8	9.9	31.7
**34**	2	IV	c	>10,000	>10,000	666	851	5.7	14.3
**35**	3	IV	c	>10,000	>10,000	523	335	10.4	14.3
**36**	4	IV	c	>10,000	>10,000	332	298	19.0	31.7
**AAZ**				250.0	12.0	25.0	5.7	-	-
**Ver**				-	-	-	-	1.2	3.0

^1^ Mean from 3 different assays, by a stopped-flow technique (errors were in the range of ±5–10% of the reported values). ^2^ Incubation time of 6 h. ^3^ Inhibition of the P-gp transport activity in K562/DOX cells expressed as RF that is the ratio between the IC_50_ of doxorubicin alone and in the presence of modulators (RF = IC_50_ of doxorubicin–modulator/IC_50_ of doxorubicin + modulator).

**Table 2:** MDR activity profile of compounds **13**, **27**, **32** and **33** in MDCK-MDR1, MDCK-MRP1 and MDCK-BCRP cells, overexpressing P-gp, MRP1 and BCRP, respectively.

	EC_50_ µM ^1^		
Cmpd	MDR1	MRP1	BCRP
**13**	14.45 ± 0.25	52 ± 10	NA
**27**	3.99 ± 0.11	NA	NA
**32**	4.53 ± 0.17	NA	NA
**33**	3.02 ± 0.09	NA	NA

^1^ Values are the mean ± SEM of two independent experiments, with samples in triplicate. NA = not active.

## Data Availability

Data are contained within the article and Supplementary Materials.

## Supplementary Materials

The following supporting information can be downloaded at: https://www.mdpi.com/article/10.3390/molecules29143290/s1, ^1^H-NMR (400 MHz), ^13^C-APT-NMR (100 MHz) spectra of compounds **1**–**36**. Figure S1: cytotoxicity in K562/DOX cells of compounds **1**–**36**, Figure S2: cytotoxicity in HT29 cells of compounds **1**–**36**, Figure S3: cytotoxicity in A549 cells of compounds **1**–**36**, Figure S4: cytotoxicity in HT29/DOX cells of compounds **1**–**36**, Figure S5: cytotoxicity in A549/DOX cells of compounds **1**–**36**, Figure S6: antiproliferative activity in HT29 and A549 cells of doxorubicin alone and in co-administration with selected derivatives (**1**, **2**, **4**–**6**, **14**, **19**, **33**), Figure S7: intracellular accumulation in HT29 and A549 cells of doxorubicin alone and in co-administration of selected derivatives (**1**, **2**, **4**–**6**, **14**, **19**, **33**), Analytical method used to determine the purity of compounds **1**–**36**, Figures S8–S43: chromatographic profiles of HPLC-DAD analysis of compounds **1**–**36**, Figures S44–S47: UV spectra of compounds **1**–**36**.
