# Supplementary material for: Dual Inhibitors of P-gp and Carbonic Anhydrase XII (hCA XII) against Tumor Multidrug Resistance with Piperazine Scaffold
Source: Molecules. 2024 Jul 11;29(14):3290. doi: 10.3390/molecules29143290 (PMC11279465; doi:10.3390/molecules29143290)
Supplement: Supplementary file 1 [file molecules-29-03290-s001.zip › molecules-3058065-supplementary.pdf]

# Supplementary Materials

## Dual Inhibitors of P-gp and Carbonic Anhydrase XII (hCA XII) against Tumor Multidrug Resistance with Piperazine Scaffold

Laura Braconi [1,*], Chiara Riganti [2], Astrid Parenti [3], Marta Cecchi [4], Alessio Nocentini [1], Gianluca Bartolucci [1], Marta Menicatti [1], Marialessandra Contino [5], Nicola Antonio Colabufo [5], Dina Manetti [1], Maria Novella Romanelli [1], Claudiu T. Supuran [1] and Elisabetta Teodori [1,*]

[1] Department of Neuroscience, Psychology, Drug Research and Child Health - Section of Pharmaceutical and Nutraceutical Sciences, University of Florence, via Ugo Schiff 6, 50019 Sesto Fiorentino (FI), Italy; alessio.nocentini@unifi.it; gianluca.bartolucci@unifi.it; marta.menicatti@unifi.it; dina.manetti@unifi.it; novella.romanelli@unifi.it; claudiu.supuran@unifi.it

[2] Oncological Pharmacology Lab and Molecular Biotechnology Center "Guido Tarone", Department of Oncology, University of Turin, piazza Nizza 44, 10126 Torino, Italy; chiara.riganti@unito.it

[3] Department of Health Sciences - Section of Clinical Pharmacology and Oncology, University of Florence, Viale Pieraccini 6, 50139 Firenze, Italy; astrid.parenti@unifi.it

[4] Department of Neuroscience, Psychology, Drug Research and Child Health - Section of Pharmacology and Toxicology, University of Florence, Viale Pieraccini 6, 50139 Firenze, Italy; marta.cecchi@unifi.it

[5] Department of Pharmacy - Drug Sciences, University of Bari "A. Moro", via Orabona 4, 70125 Bari, Italy; marialessandra.contino@uniba.it; nicolaantonio.colabufo@uniba.it

* Correspondence: laura.braconi@unifi.it; elisabetta.teodori@unifi.it

**Table of Contents**

- $^{1}$H-NMR (400 MHz), $^{13}$C-APT- NMR (100 MHz) spectra of compounds **1-36** S2
- Figure S1: cytotoxicity in K562/DOX cells of compounds **1-36** S38
- Figure S2: cytotoxicity in HT29 cells of compounds **1-36** S39
- Figure S3: cytotoxicity in A549 cells of compounds **1-36** S40
- Figure S4: cytotoxicity in HT29/DOX cells of compounds **1-36** S41
- Figure S5: cytotoxicity in A549/DOX cells of compounds **1-36** S42
- Figure S6: antiproliferative activity in HT29 and A549 cells of doxorubicin alone and in co-administration with selected derivatives (**1**, **2**, **4-6**, **14**, **19**, **33**) S43
- Figure S7: intracellular accumulation in HT29 and A549 cells of doxorubicin alone and in co-administration of selected compounds (**1**, **2**, **4-6**, **14**, **19**, **33**) S44
- Analytical method used to determine the purity of compounds **1-36** S45
- Figures S8-S43: chromatographic profiles of HPLC-DAD analysis of compounds **1-36** S46
- Figures S44-S47: UV spectra of compounds **1-36** S64

$^{1}$H-NMR and $^{13}$C-APT-NMR spectra of compound **1**

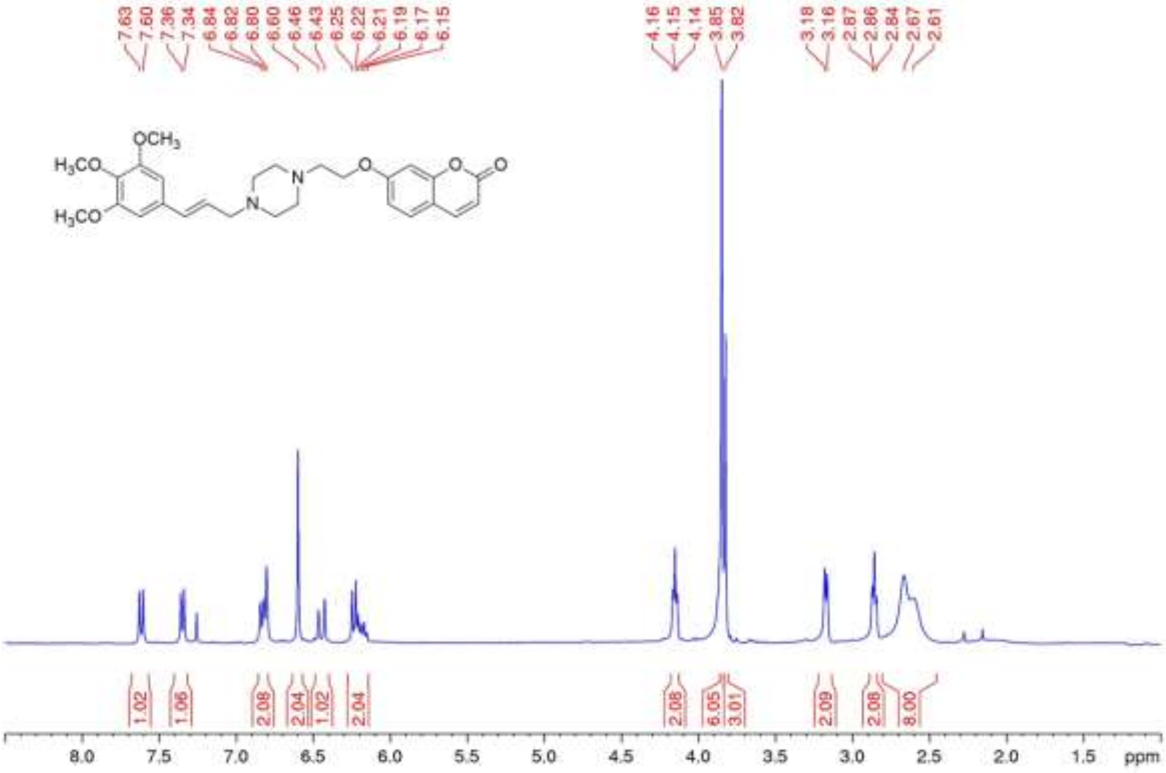


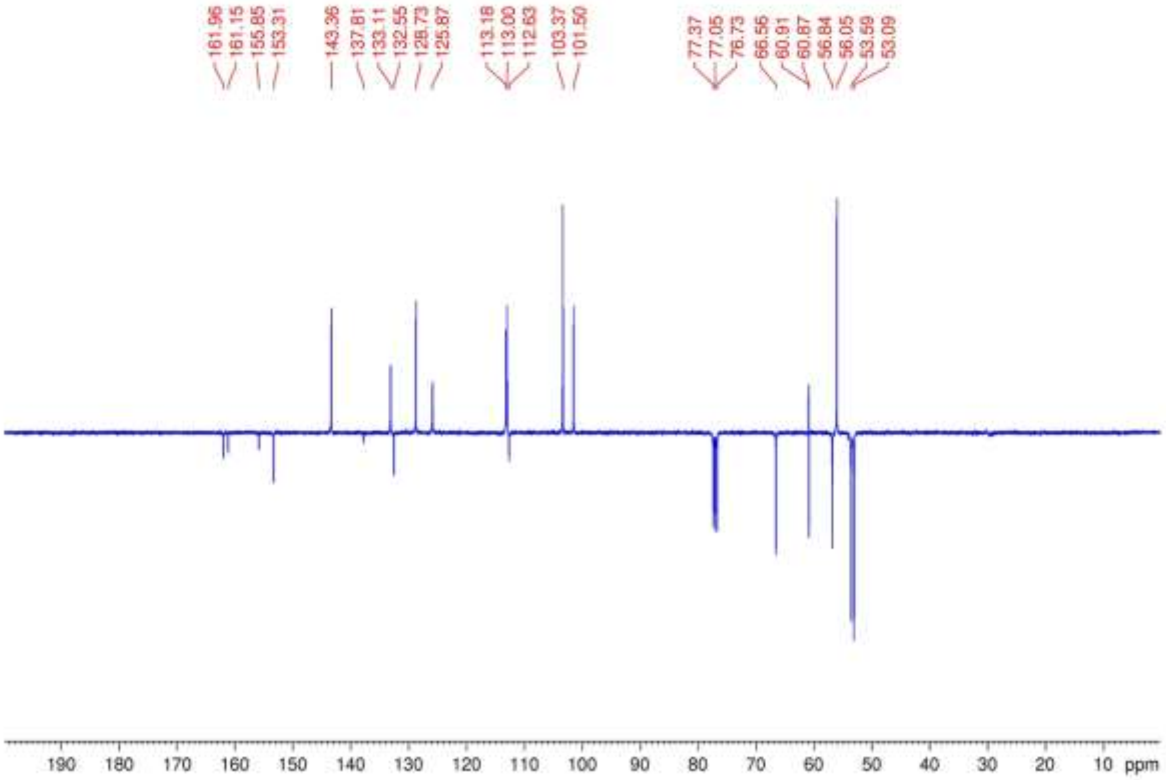

$^{1}$H-NMR and $^{13}$C-APT-NMR spectra of compound **2**

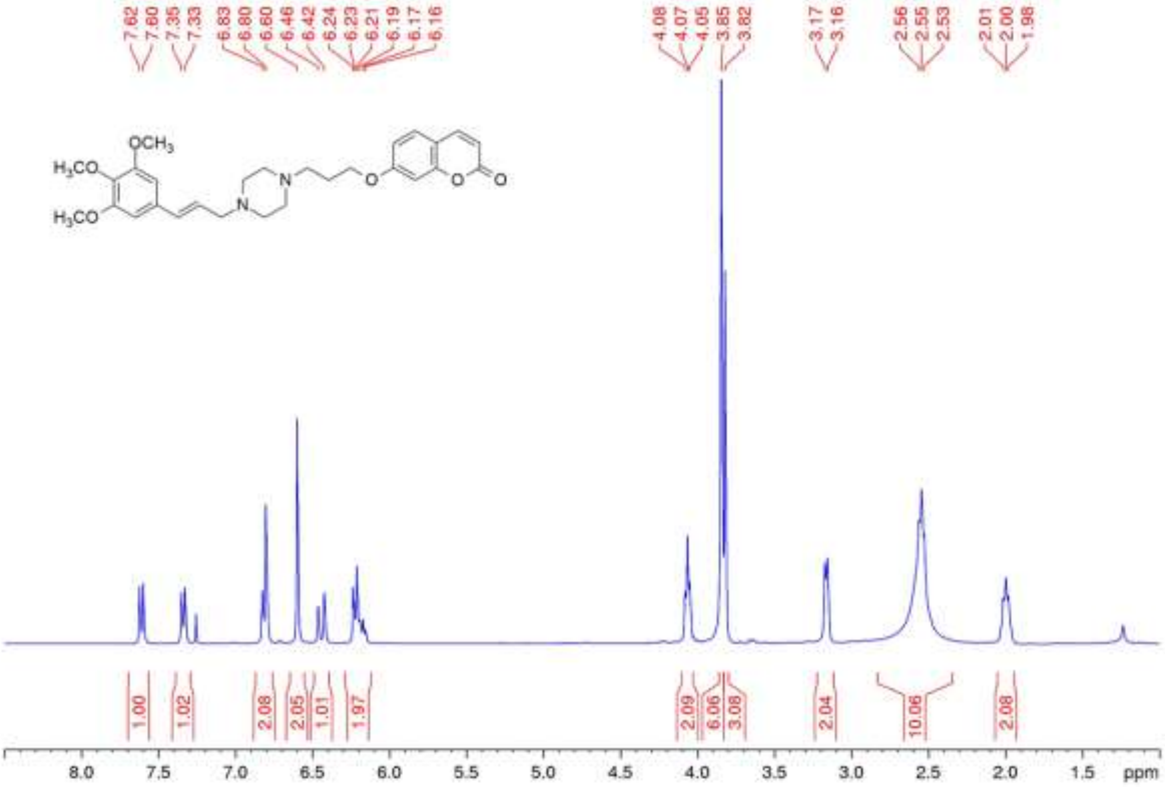


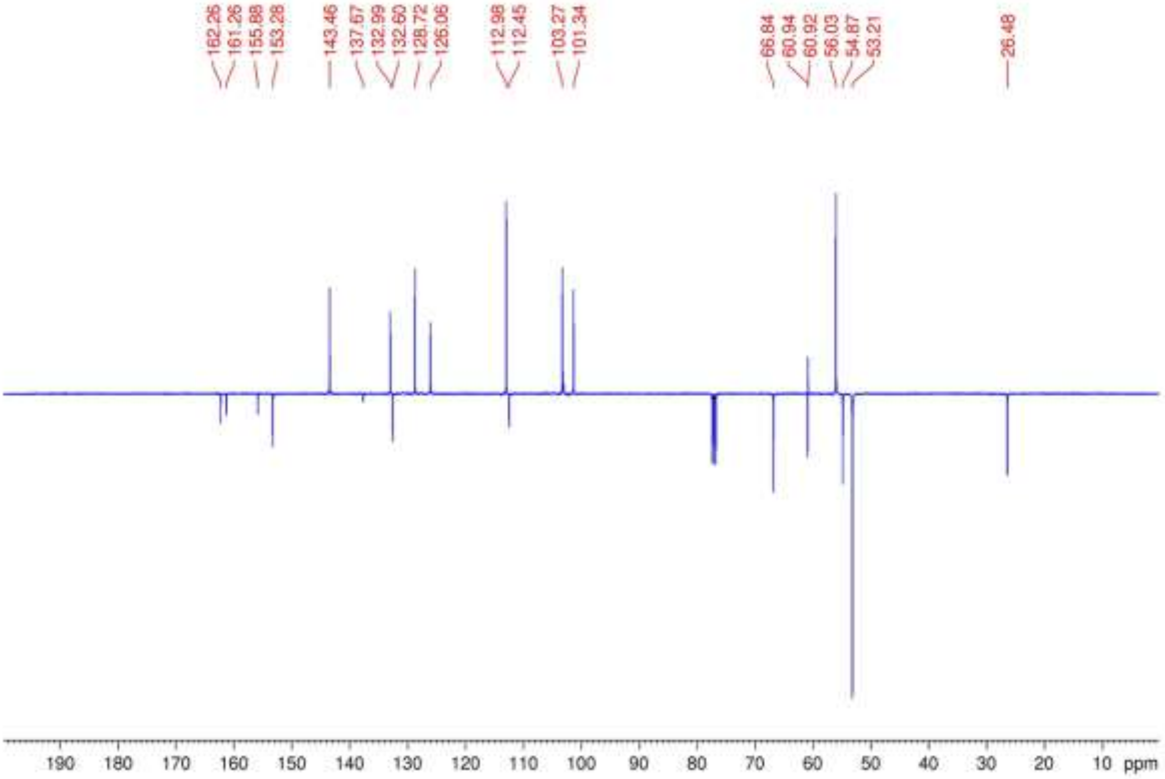

$^{1}$H-NMR and $^{13}$C-APT-NMR spectra of compound **3**

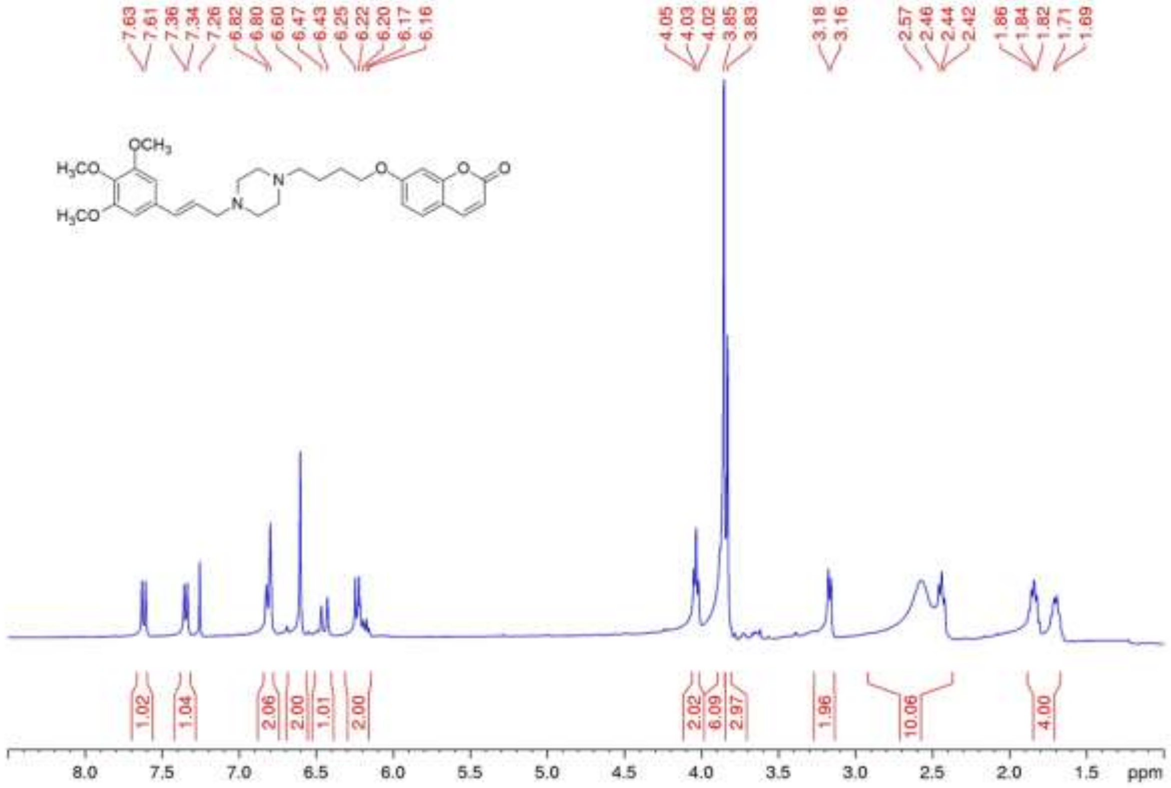


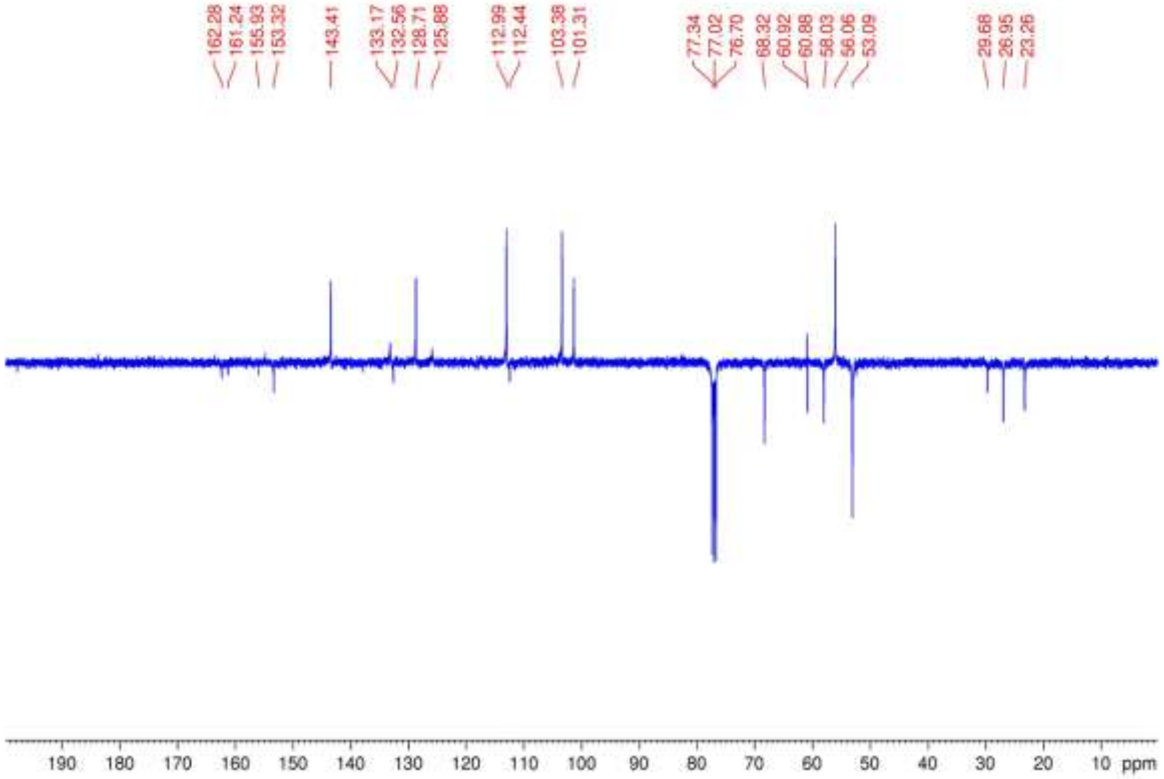

$^{1}$H-NMR and $^{13}$C-APT-NMR spectra of compound **4**

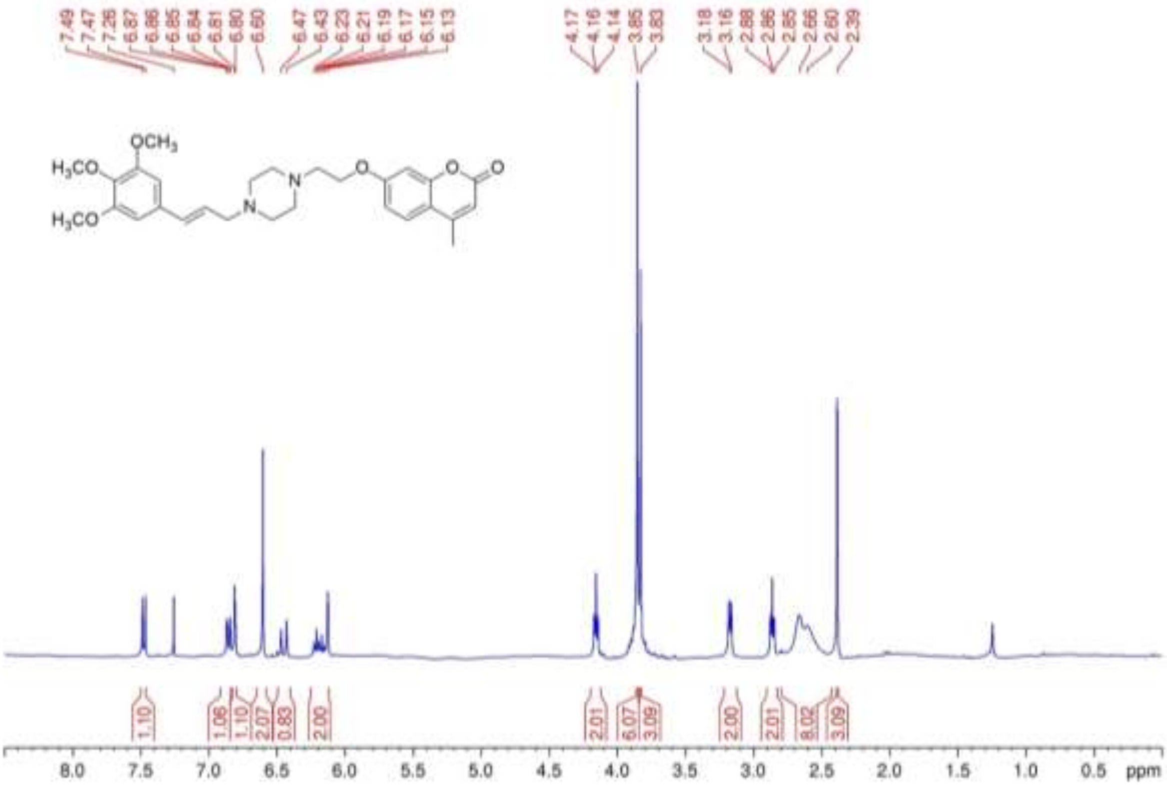


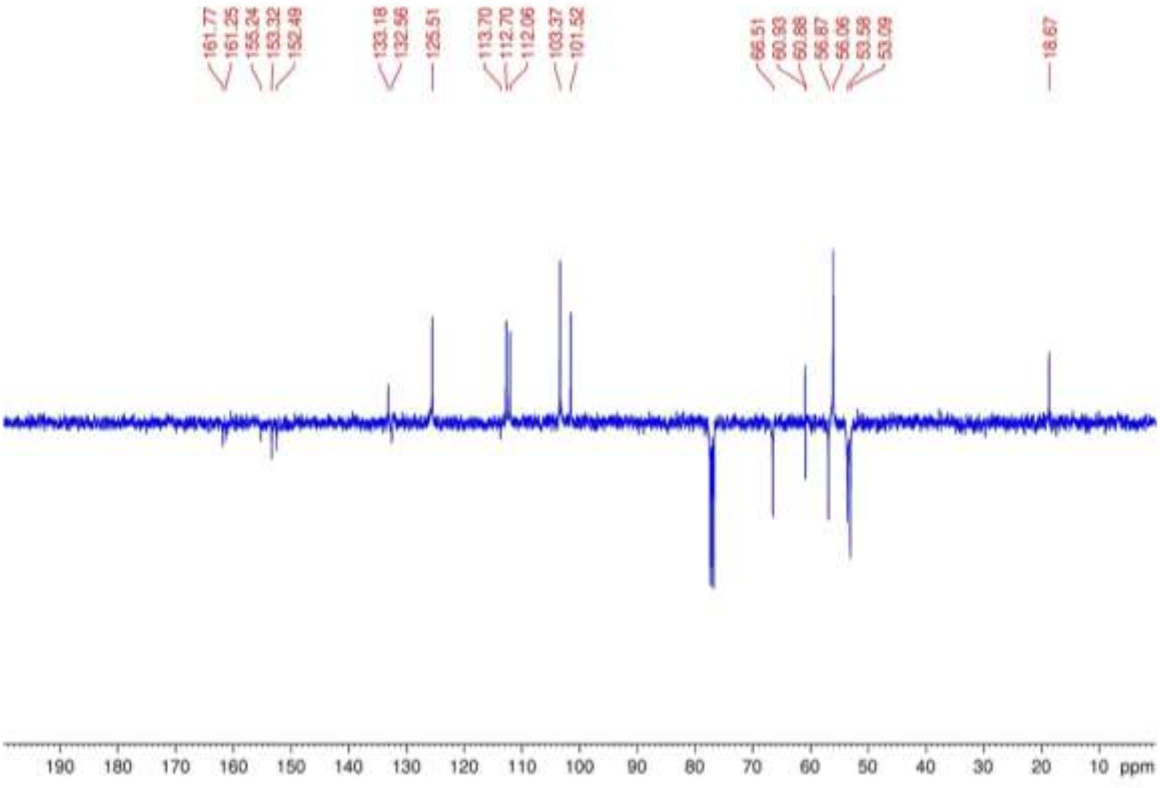

$^{1}$H-NMR and $^{13}$C-APT-NMR spectra of compound **5**

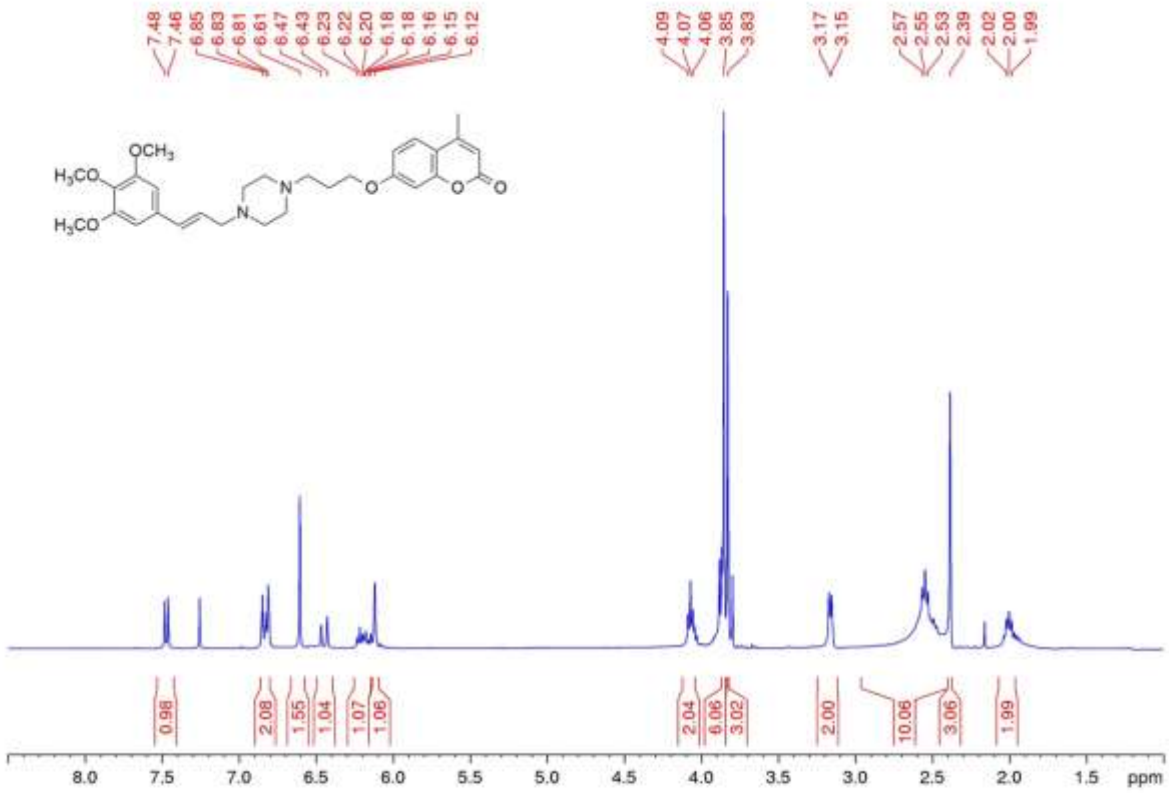

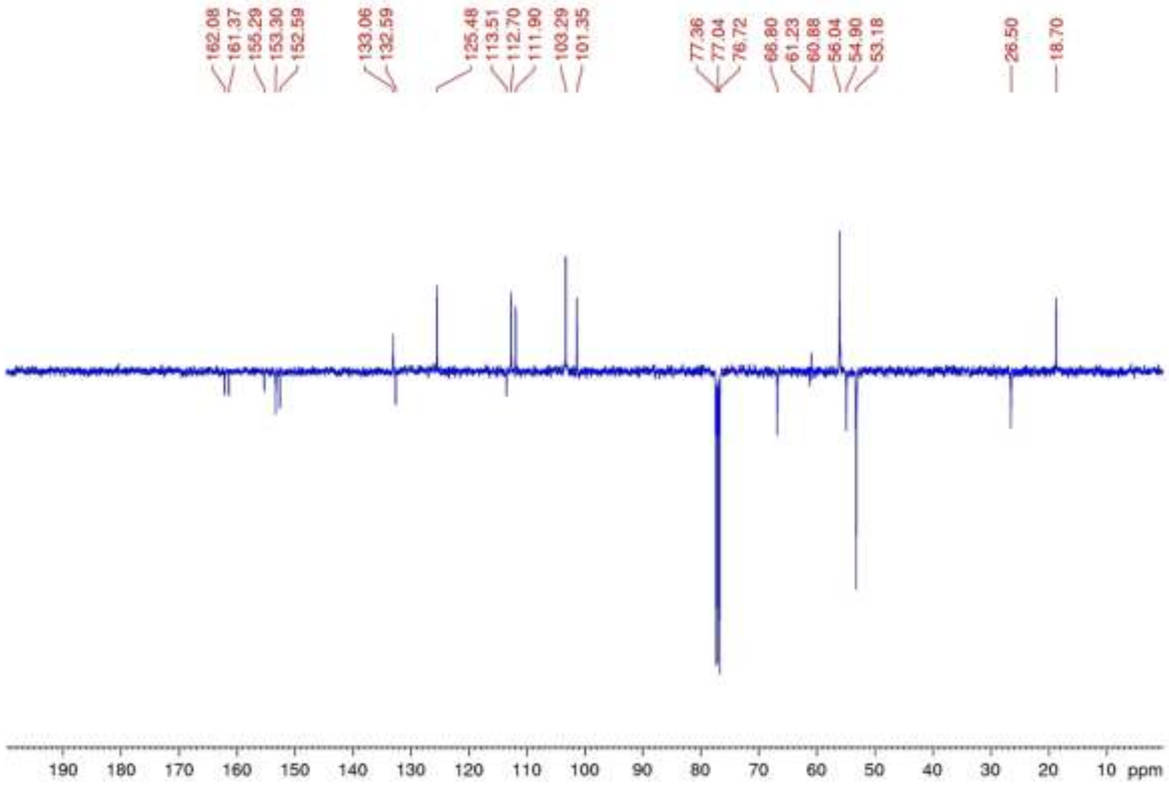

$^{1}$H-NMR spectrum of compound **6**

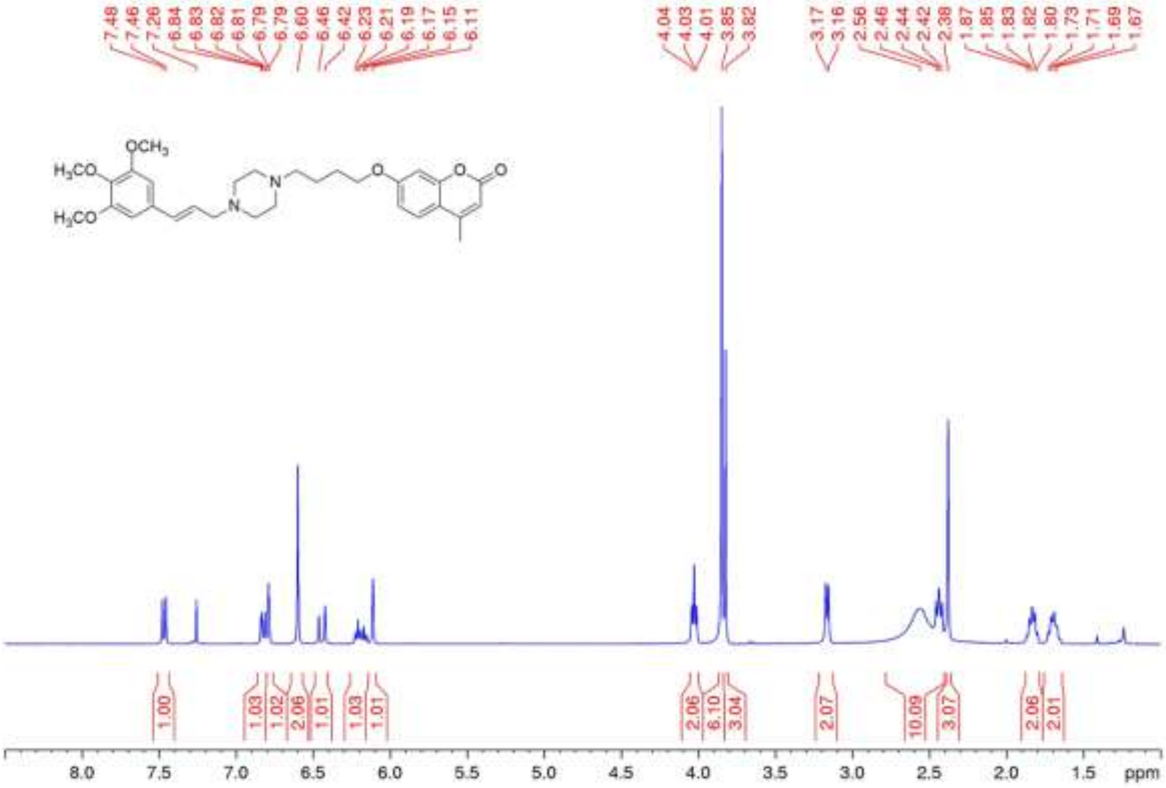

$^{1}$H-NMR and $^{13}$C-APT-NMR spectra of compound **7**

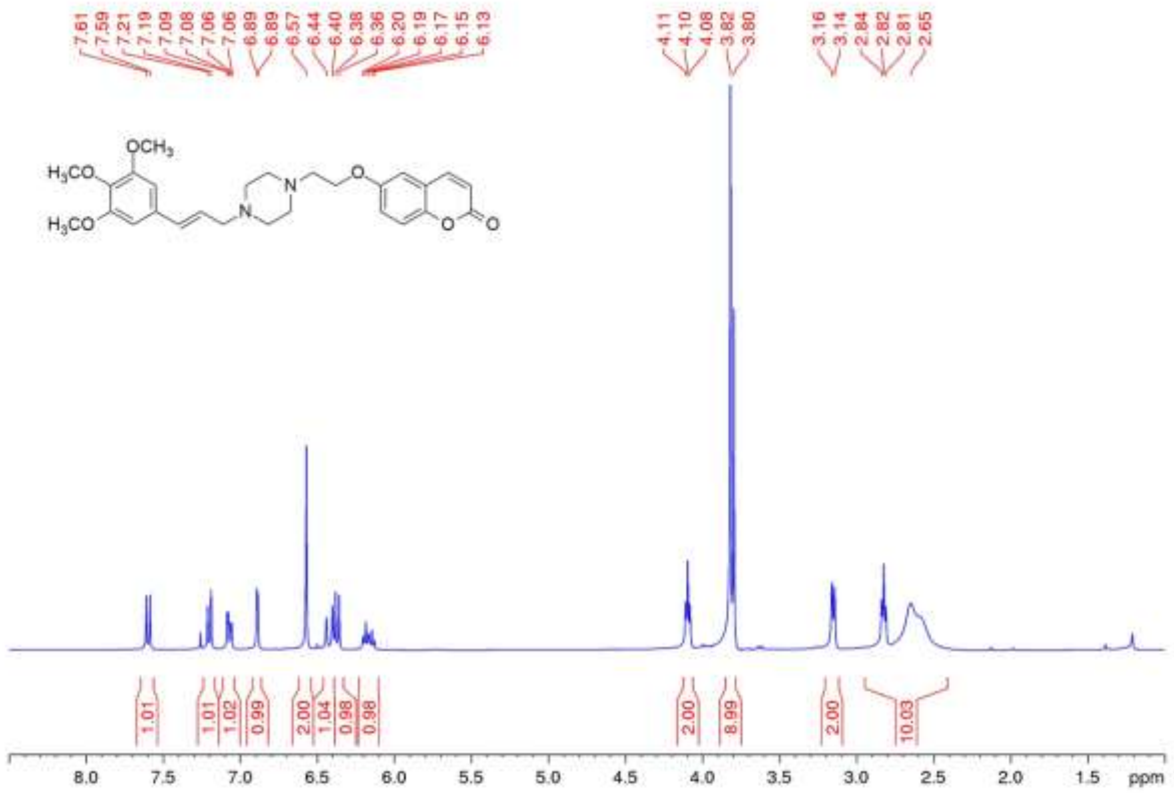

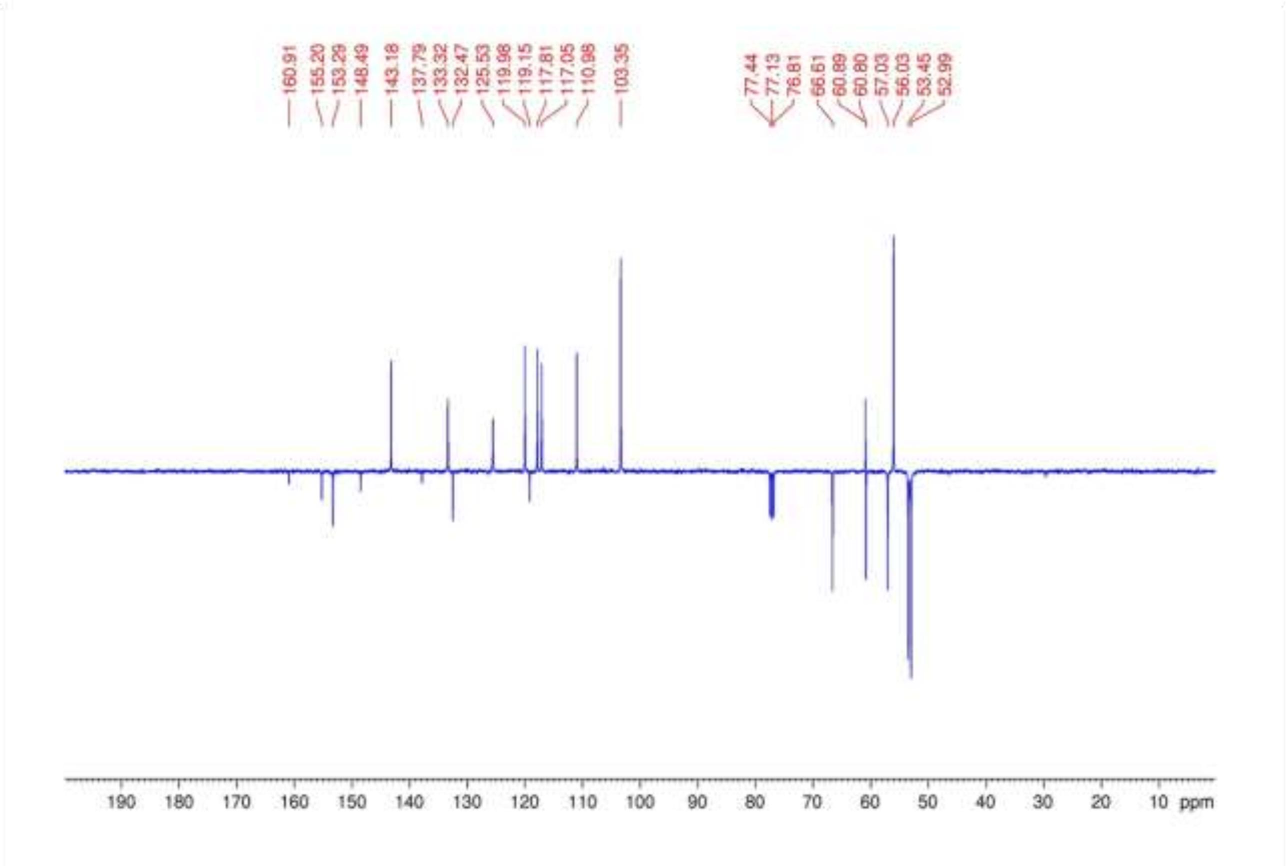

$^{1}$H-NMR and $^{13}$C-APT-NMR spectra of compound **8**

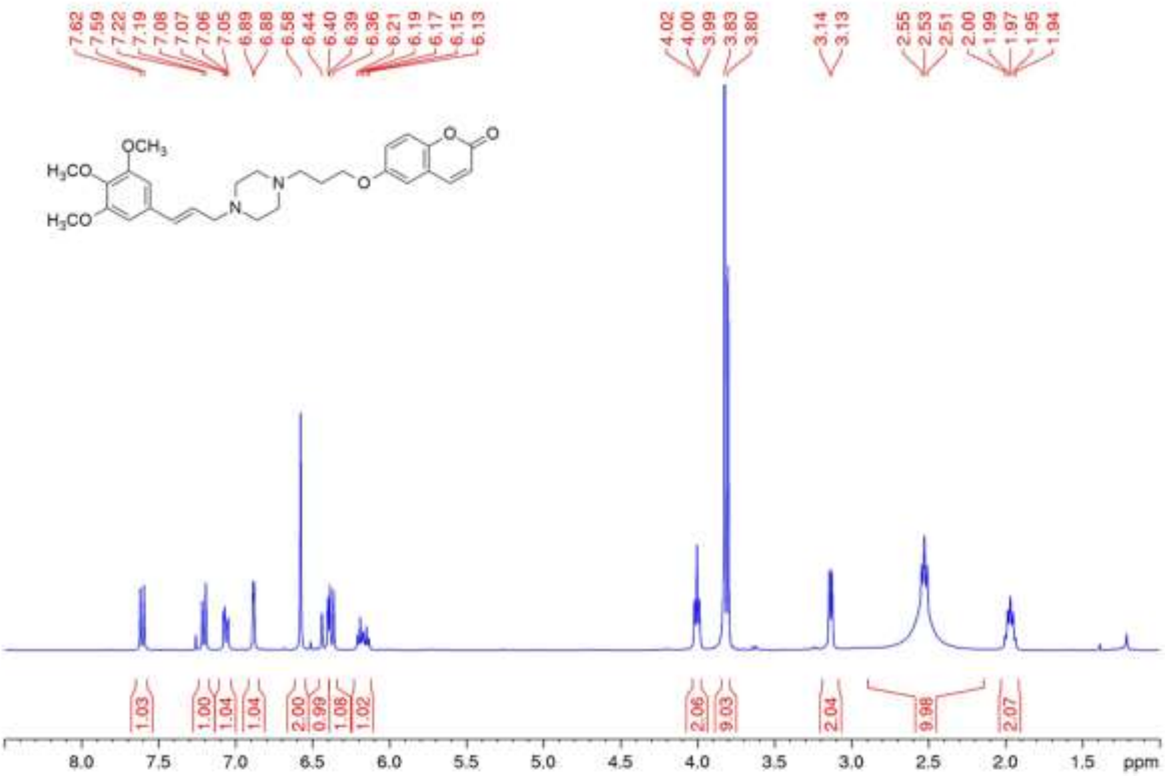


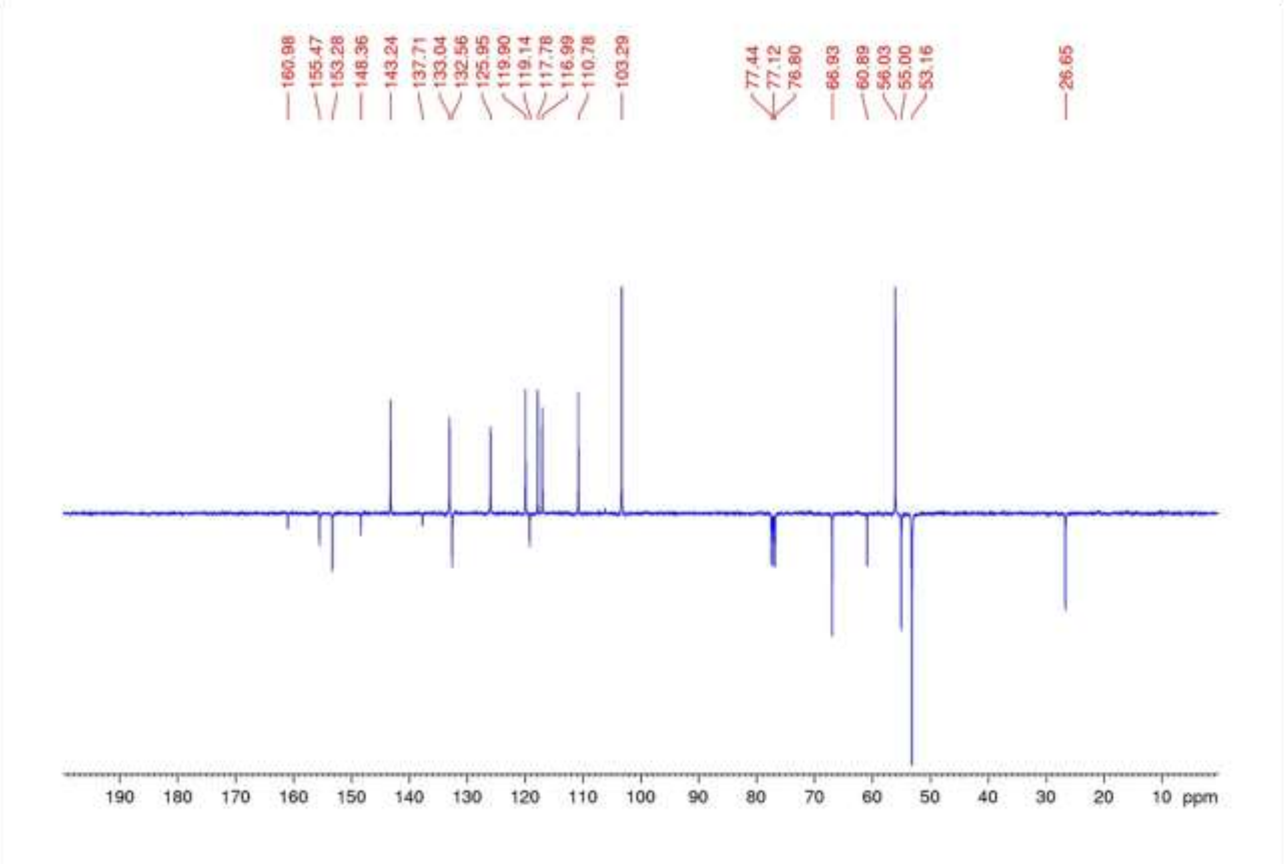

$^{1}$H-NMR and $^{13}$C-APT-NMR spectra of compound **9**

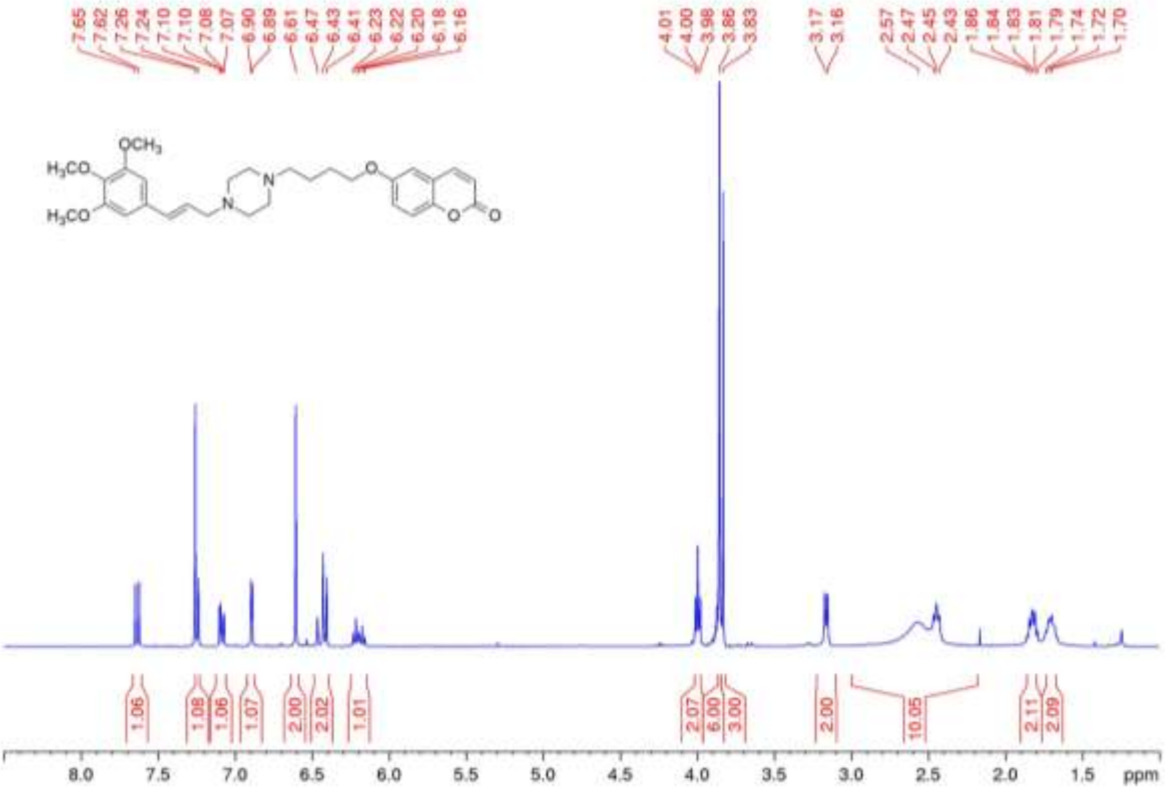


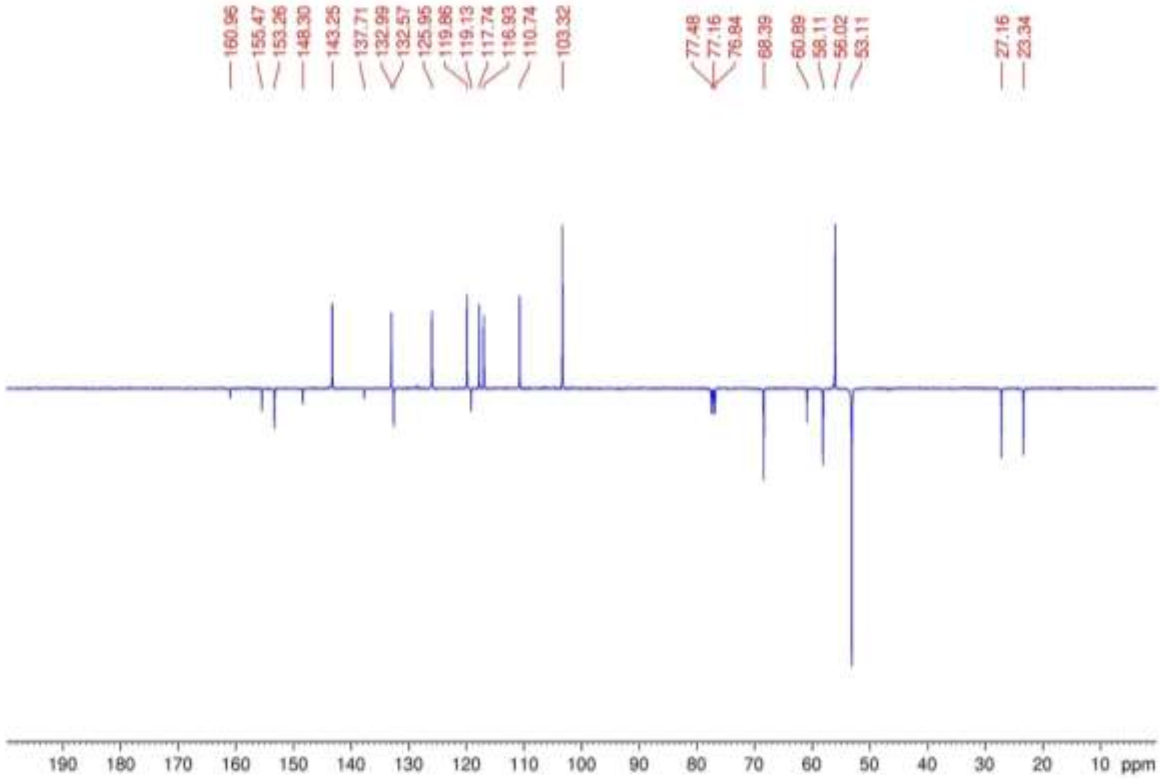

$^{1}$H-NMR and $^{13}$C-APT-NMR spectra of compound **10**

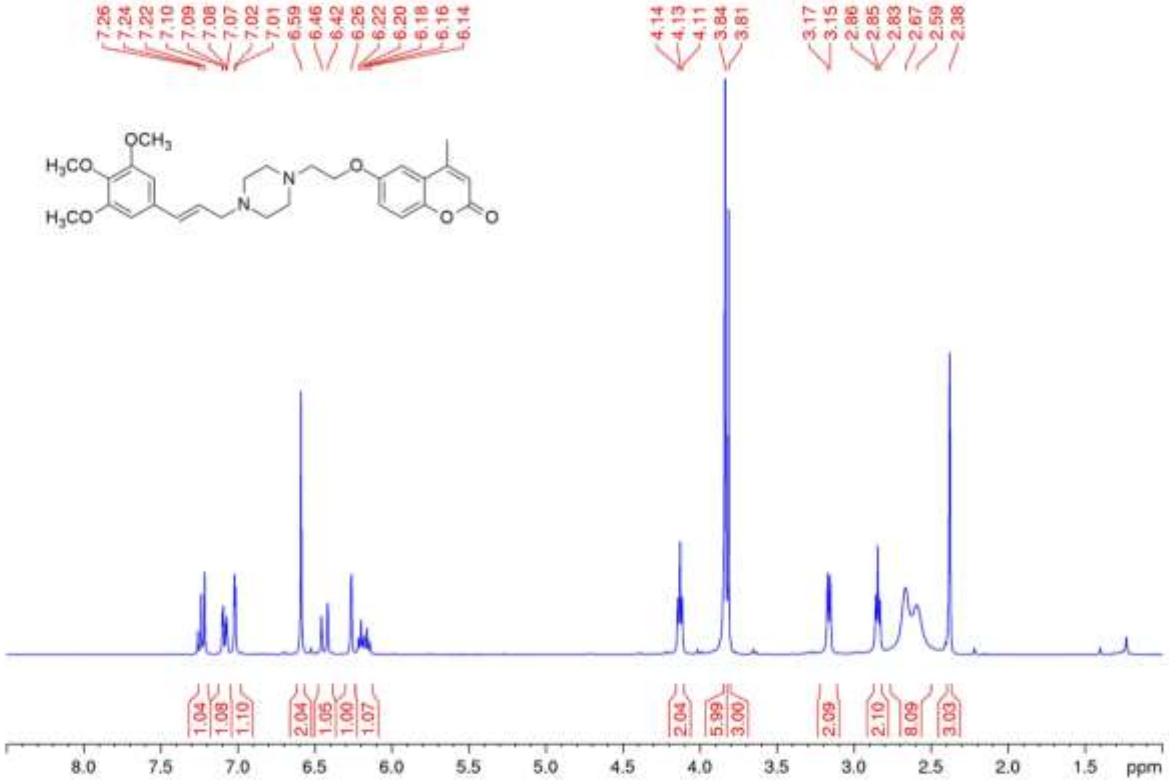


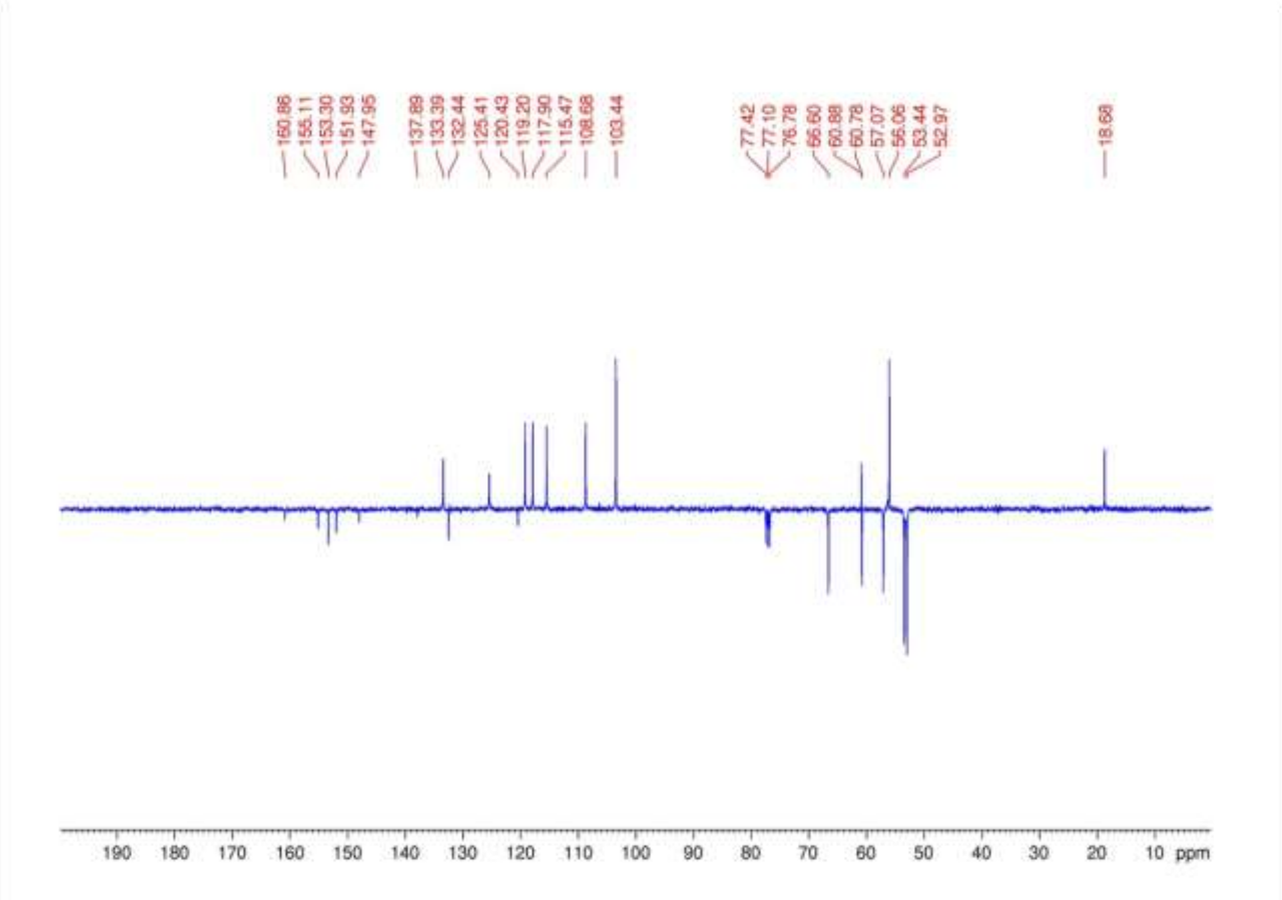

$^{1}$H-NMR and $^{13}$C-APT-NMR spectra of compound **11**

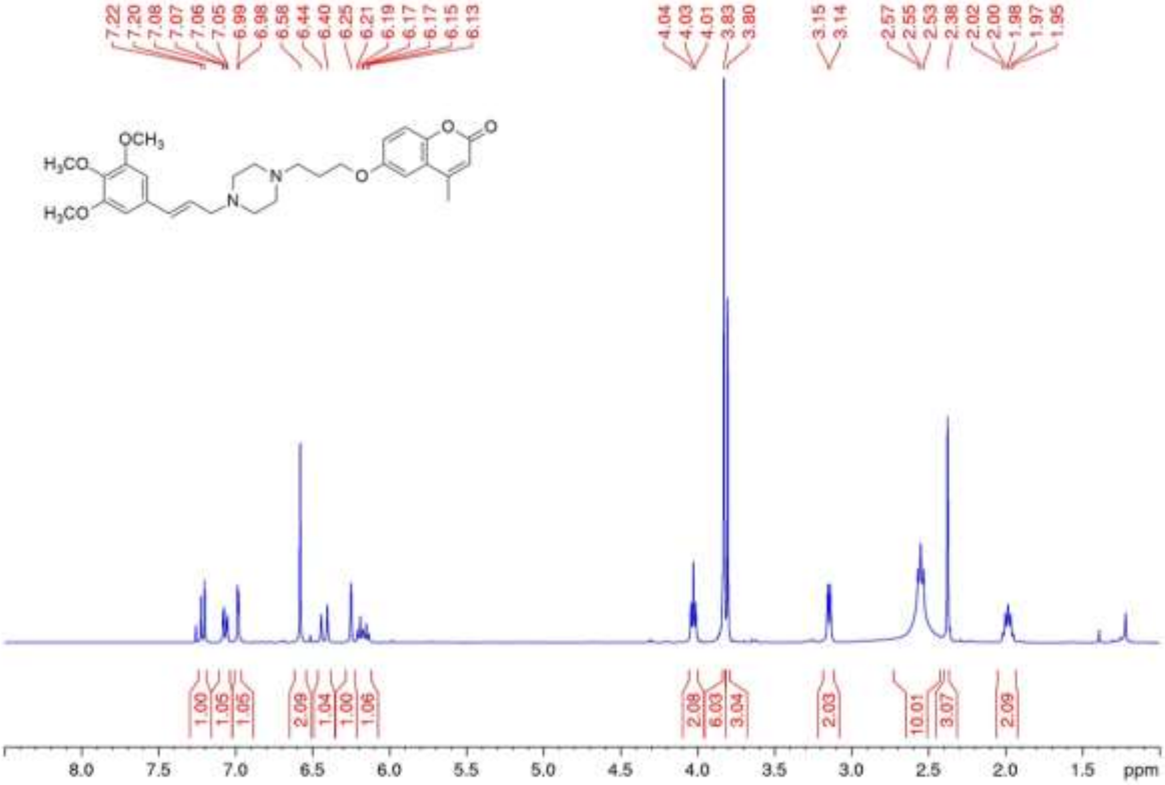


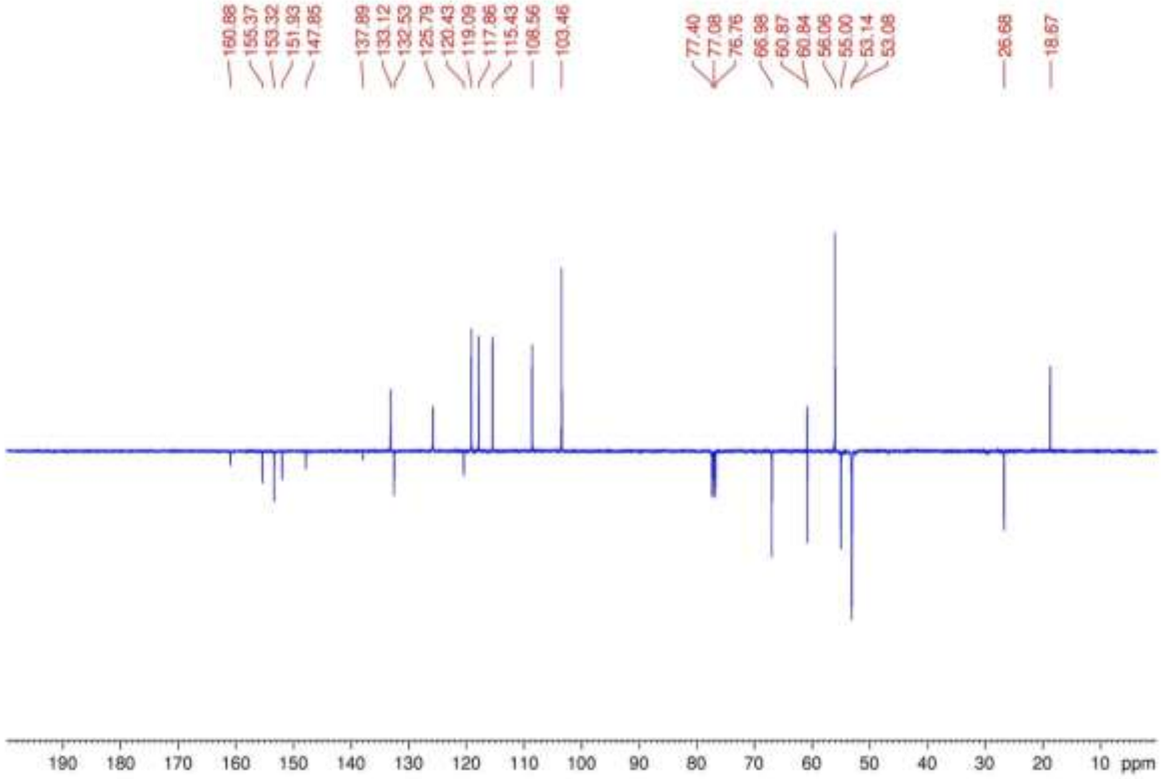

$^{1}$H-NMR and $^{13}$C-APT-NMR spectra of compound **12**

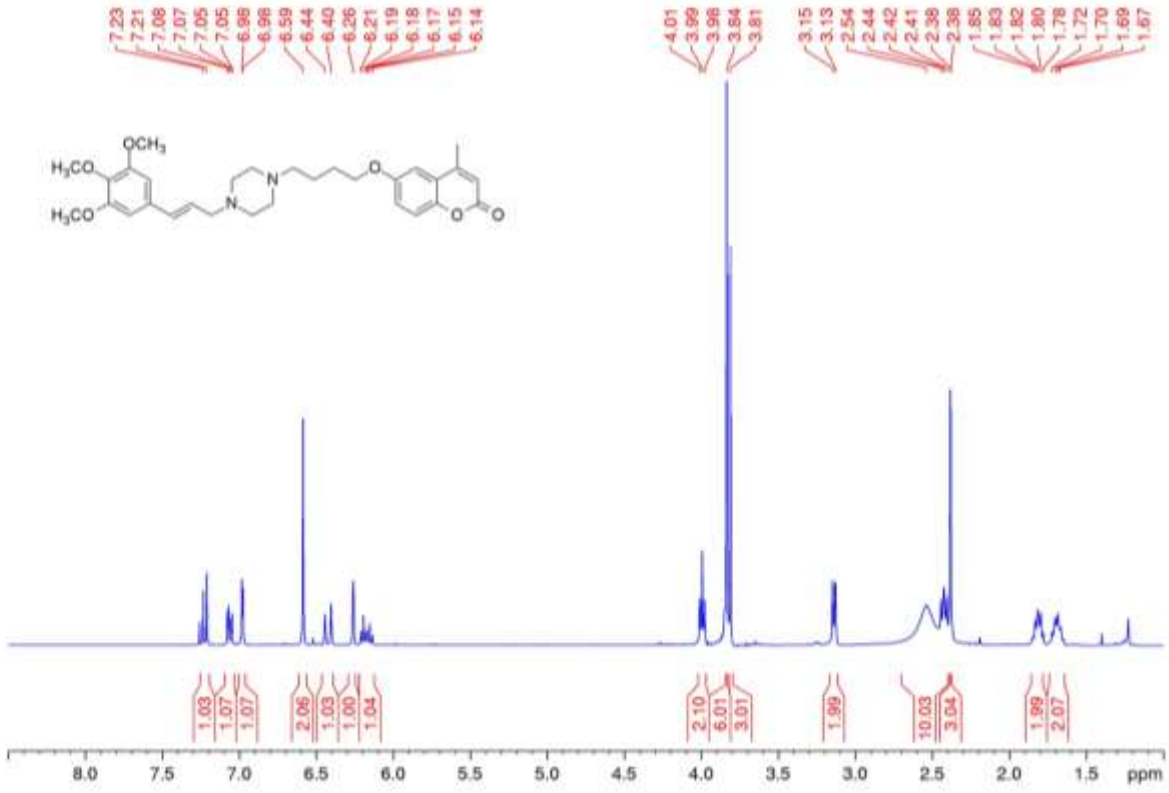

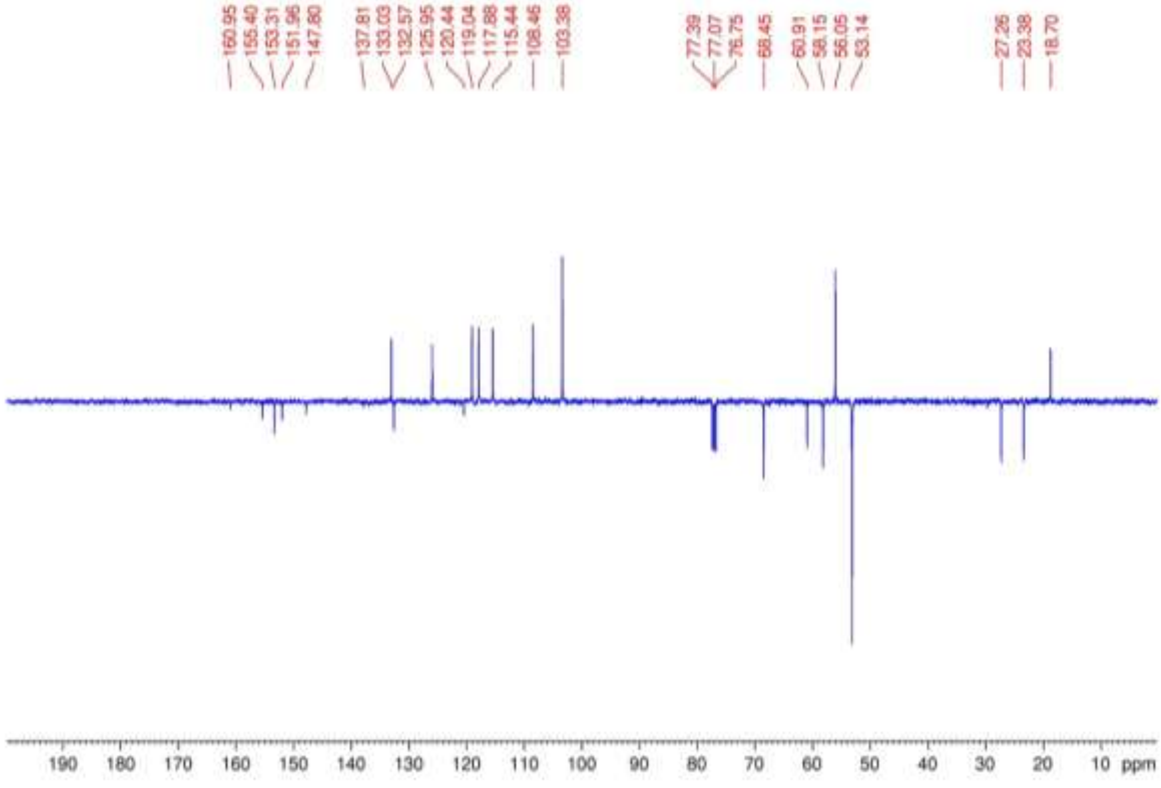

$^{1}$H-NMR and $^{13}$C-APT-NMR spectra of compound **13**

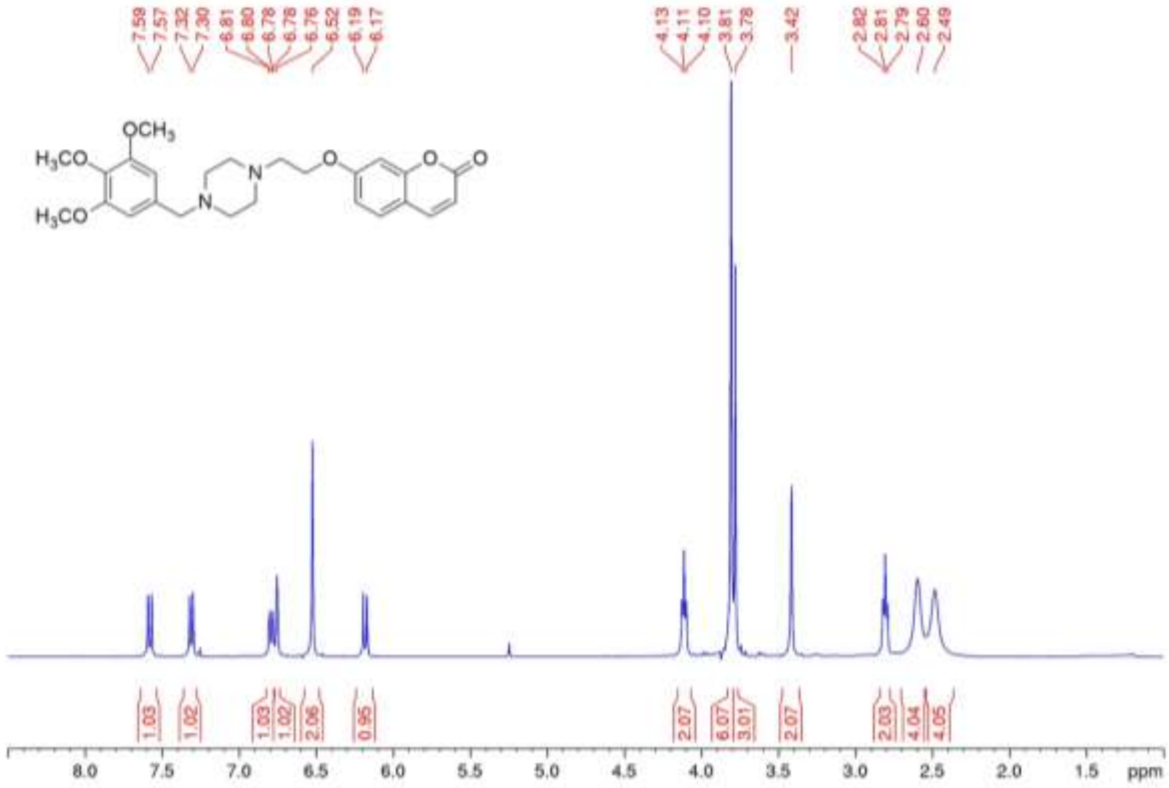


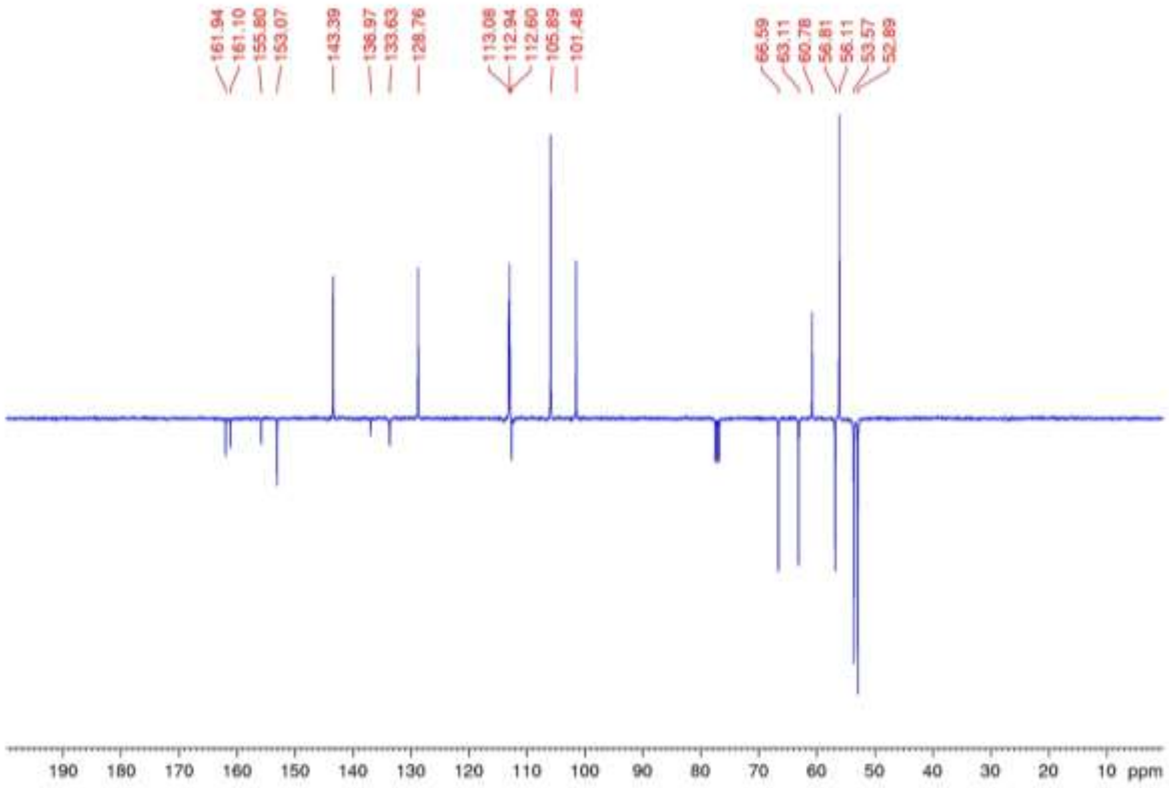

$^{1}$H-NMR and $^{13}$C-APT-NMR spectra of compound **14**

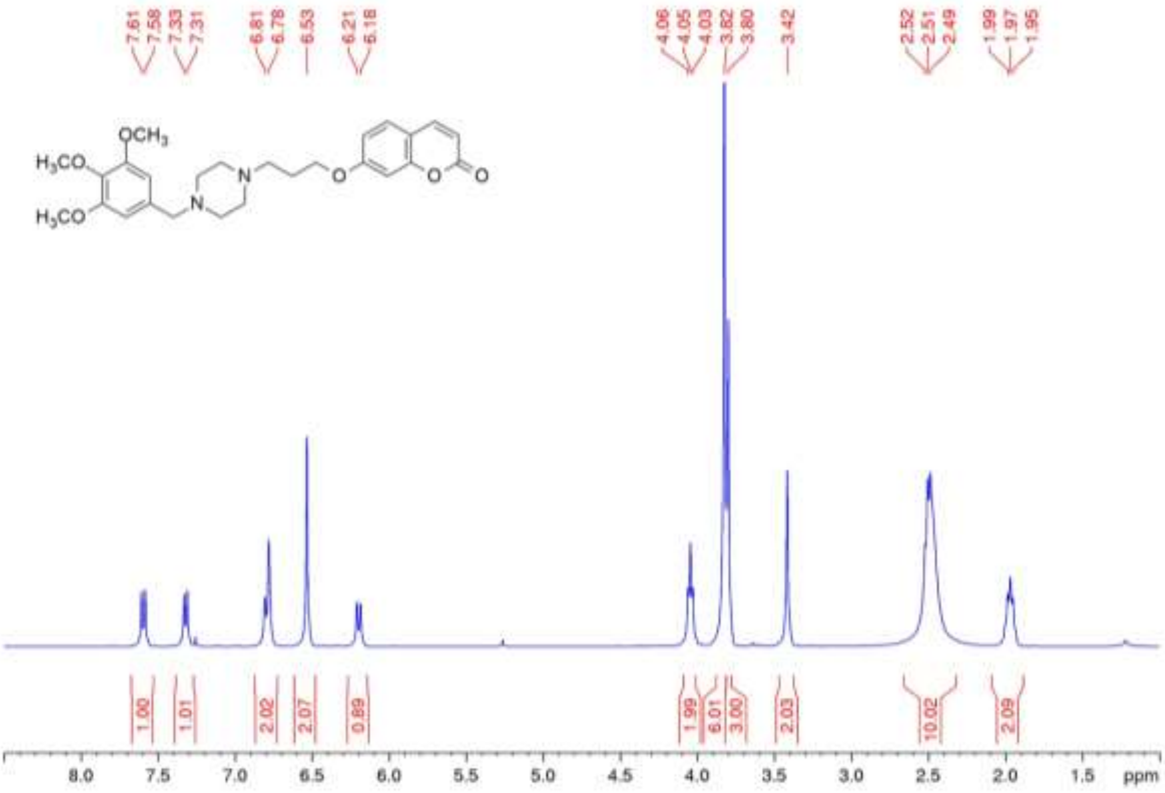


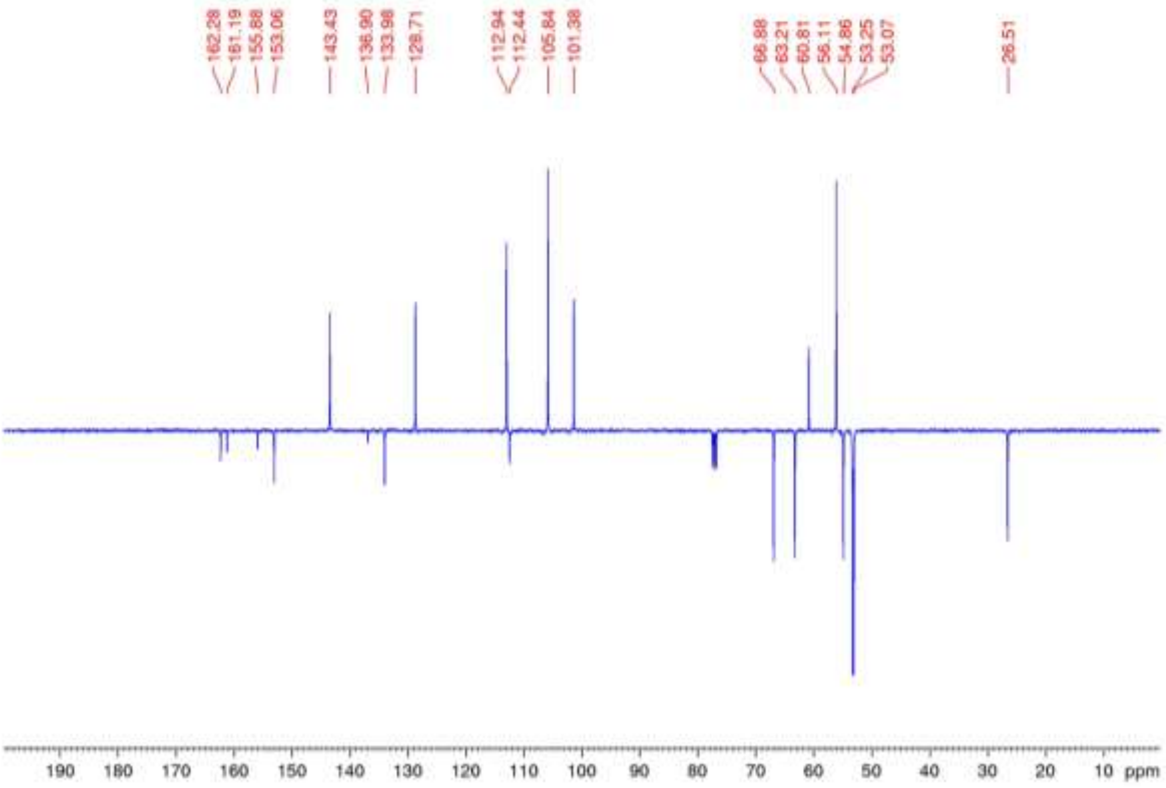

$^{1}$H-NMR and $^{13}$C-APT-NMR spectra of compound **15**

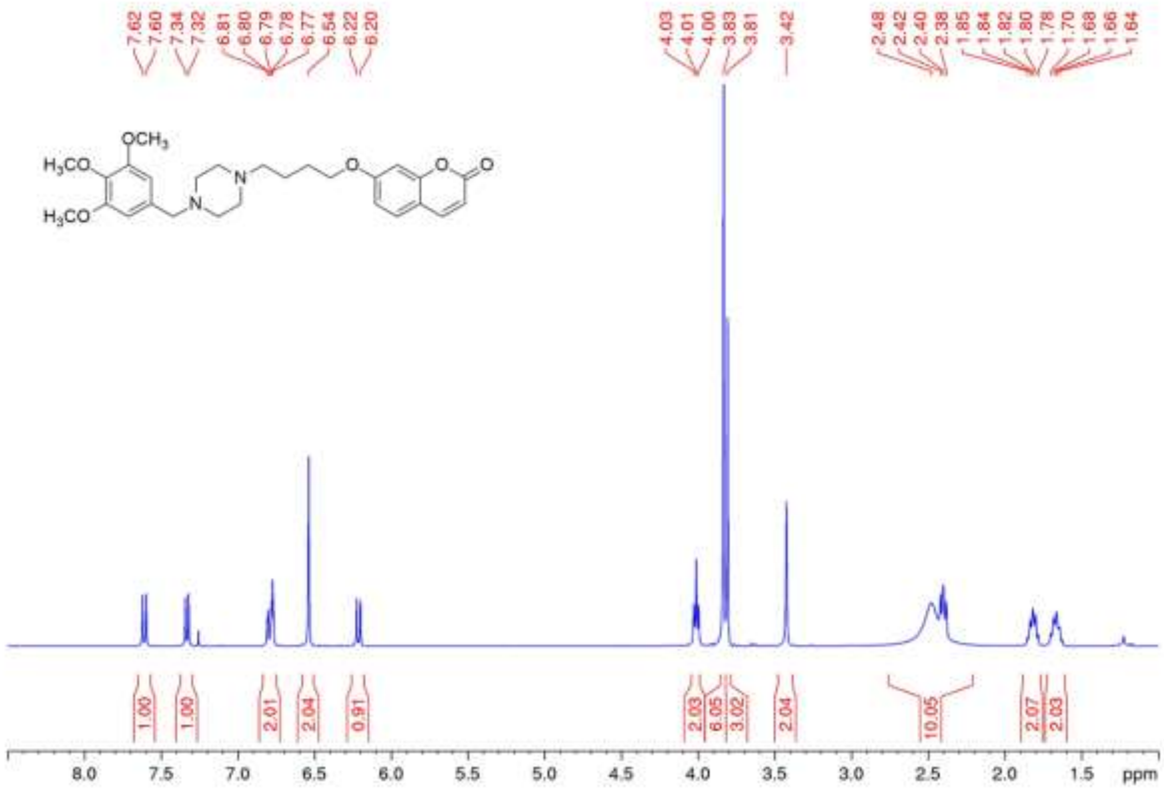


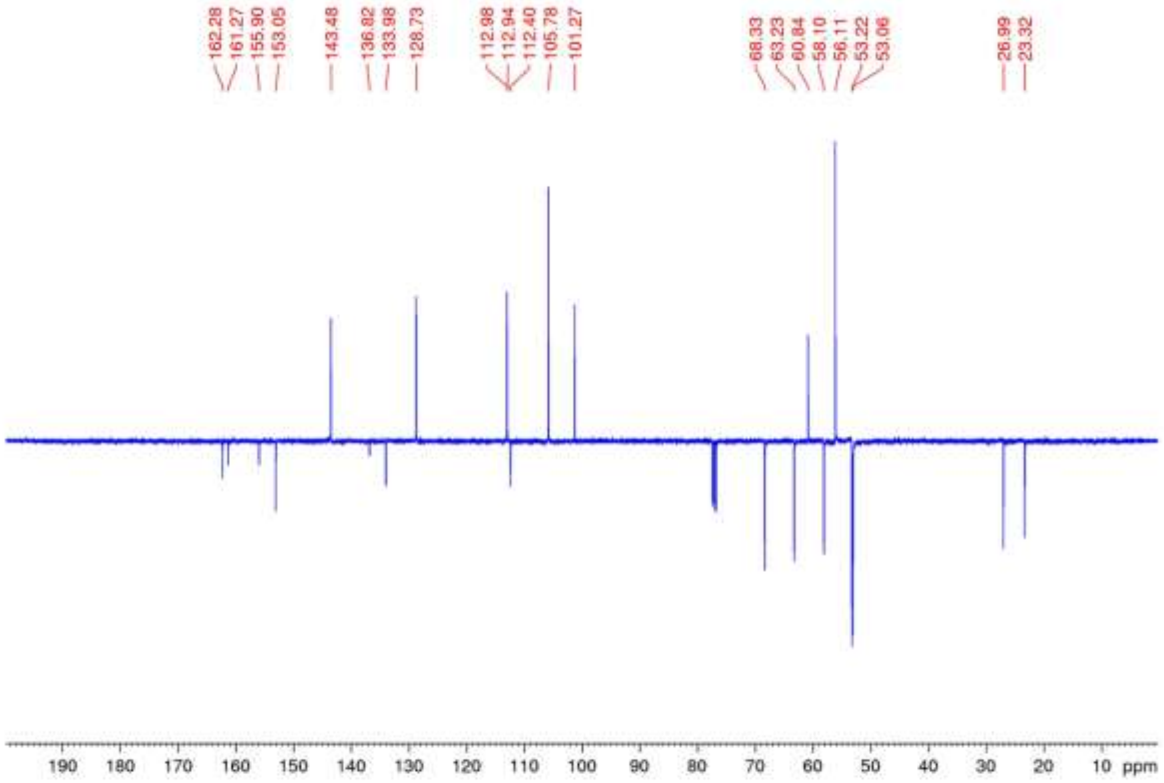

<sup></sup>

$^{1}$H-NMR and $^{13}$C-APT-NMR spectra of compound **16**

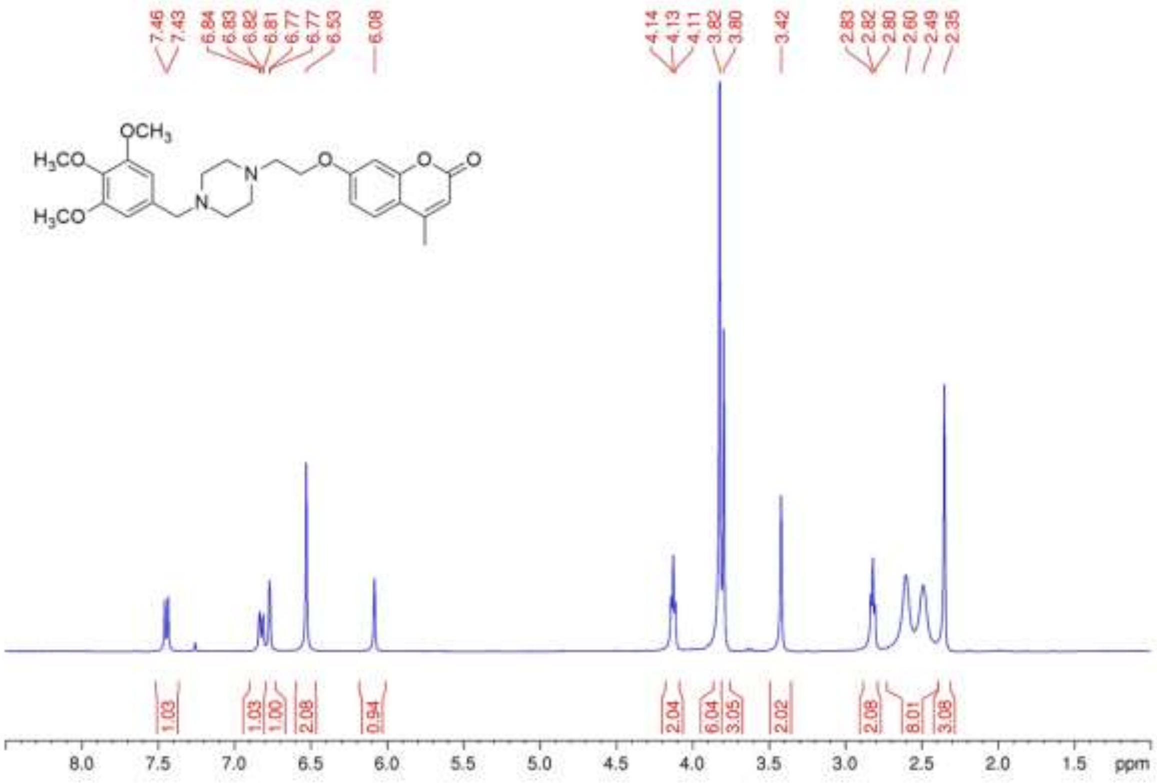

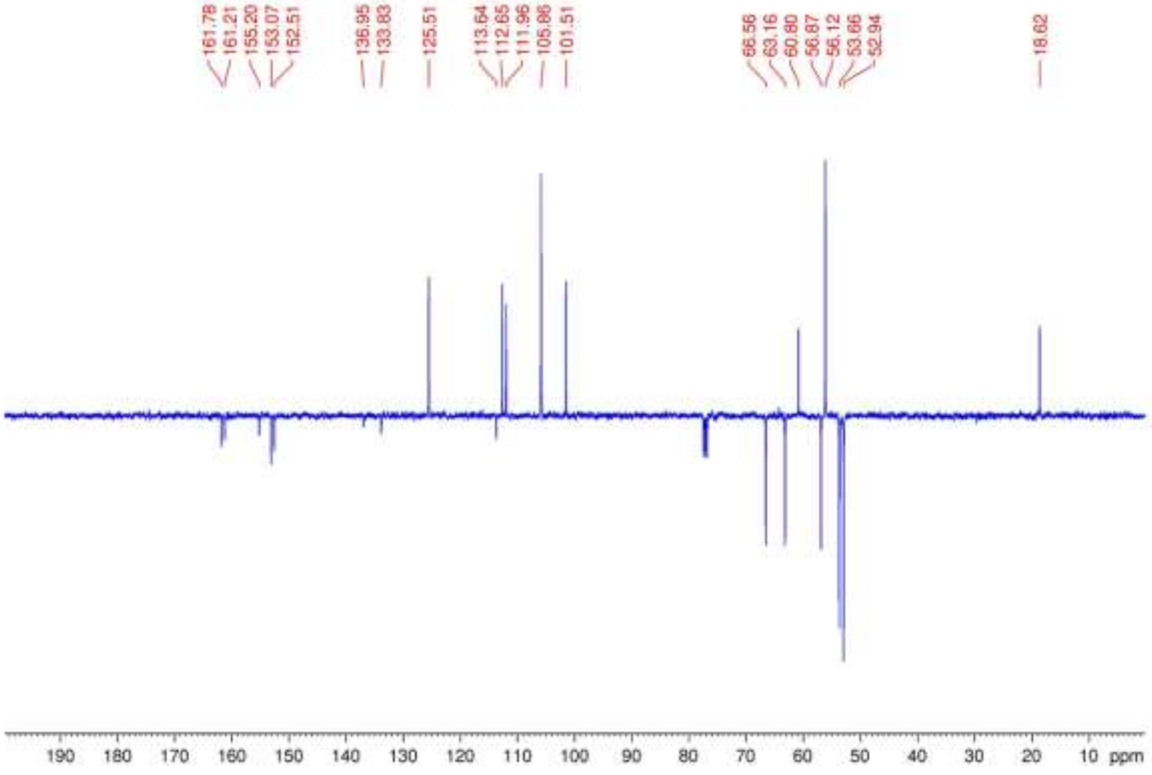

$^{1}H$-NMR and $^{13}C$-APT-NMR spectra of compound **17**

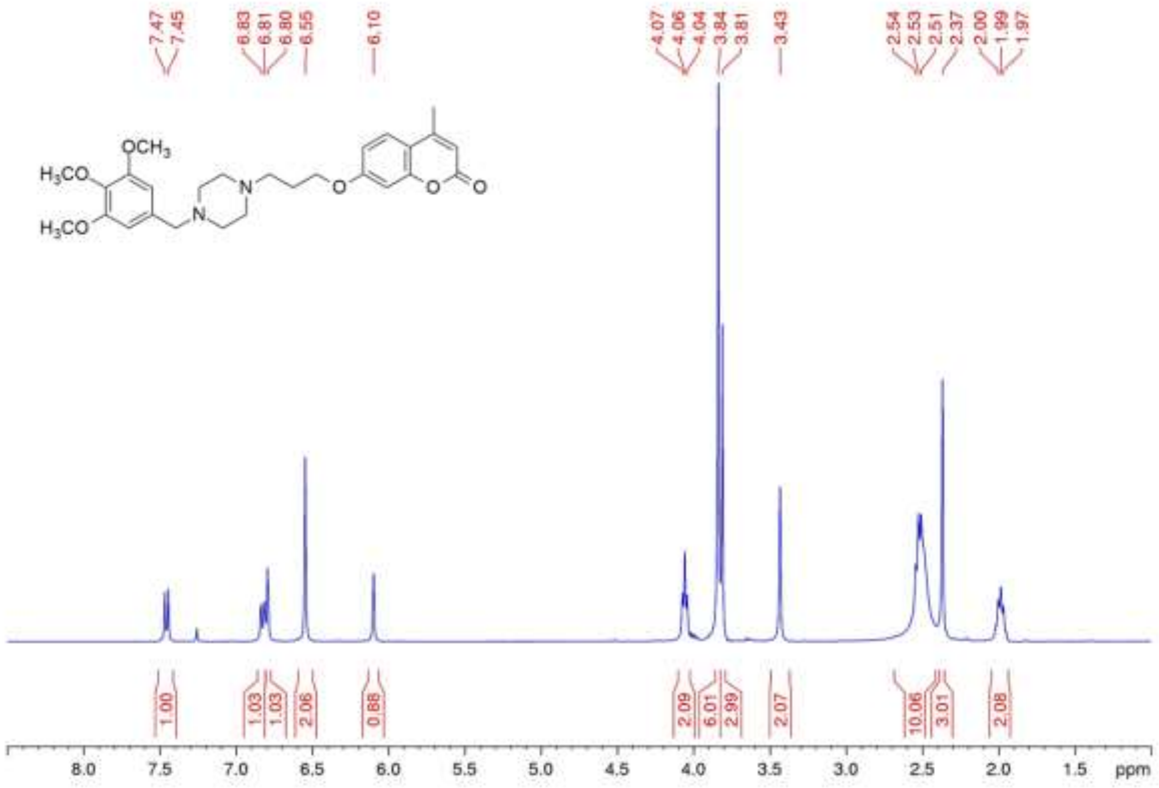


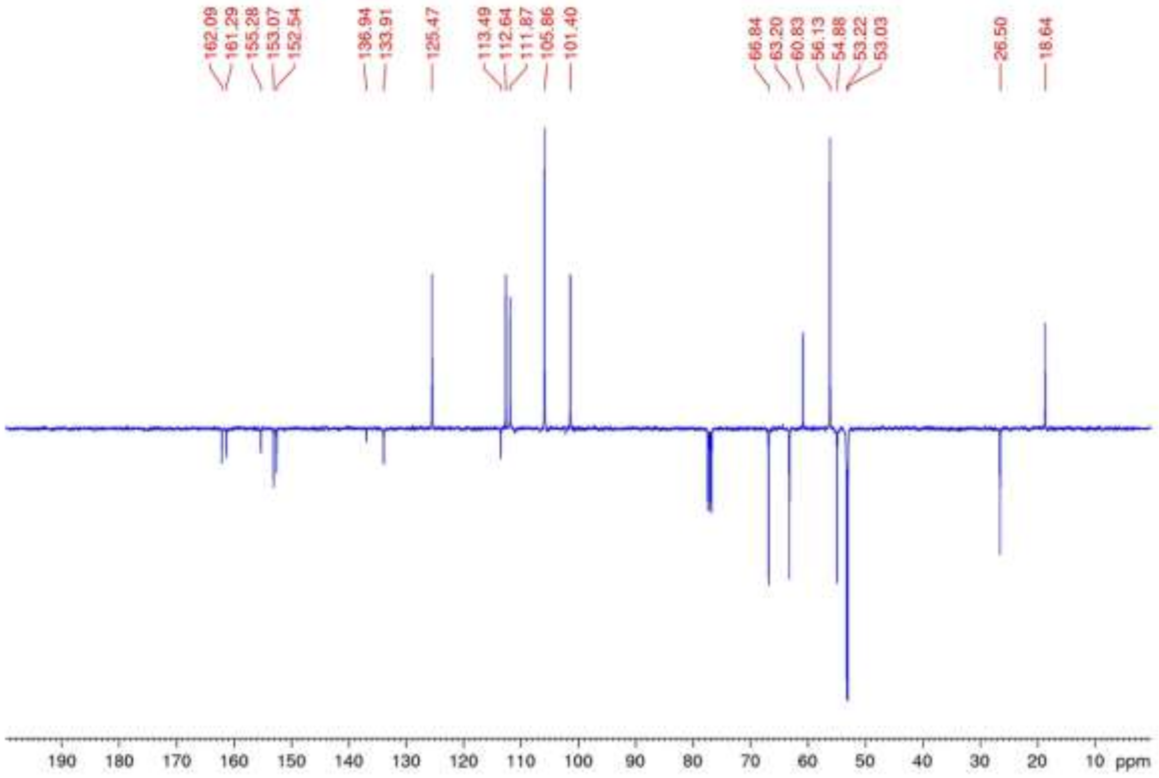

$^{1}$H-NMR and $^{13}$C-APT-NMR spectra of compound **18**

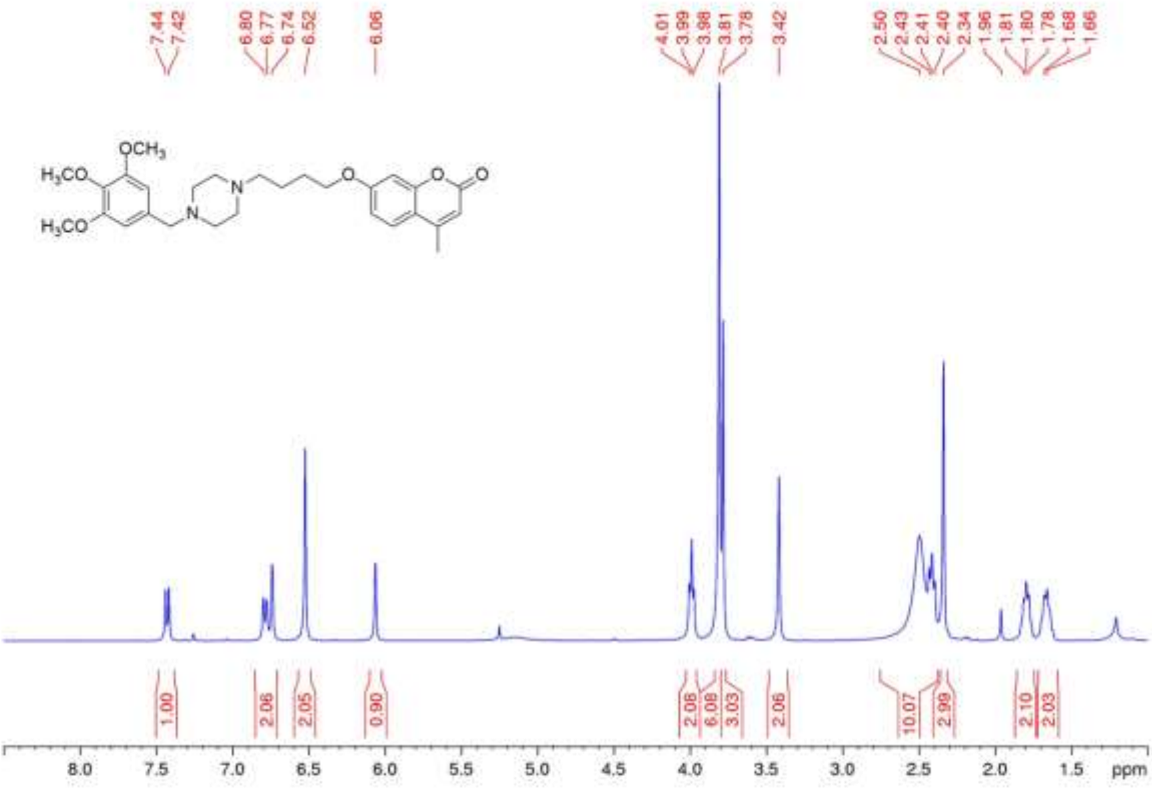


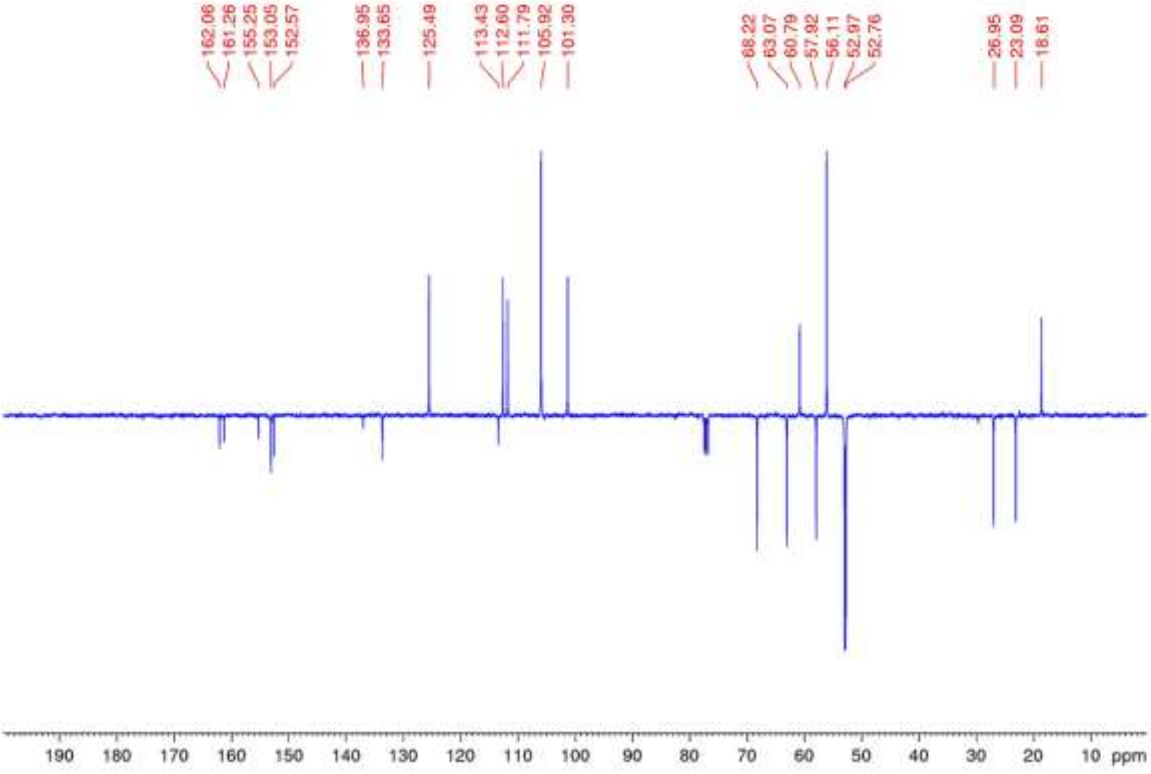

$^{1}$H-NMR and $^{13}$C-APT-NMR spectra of compound **19**

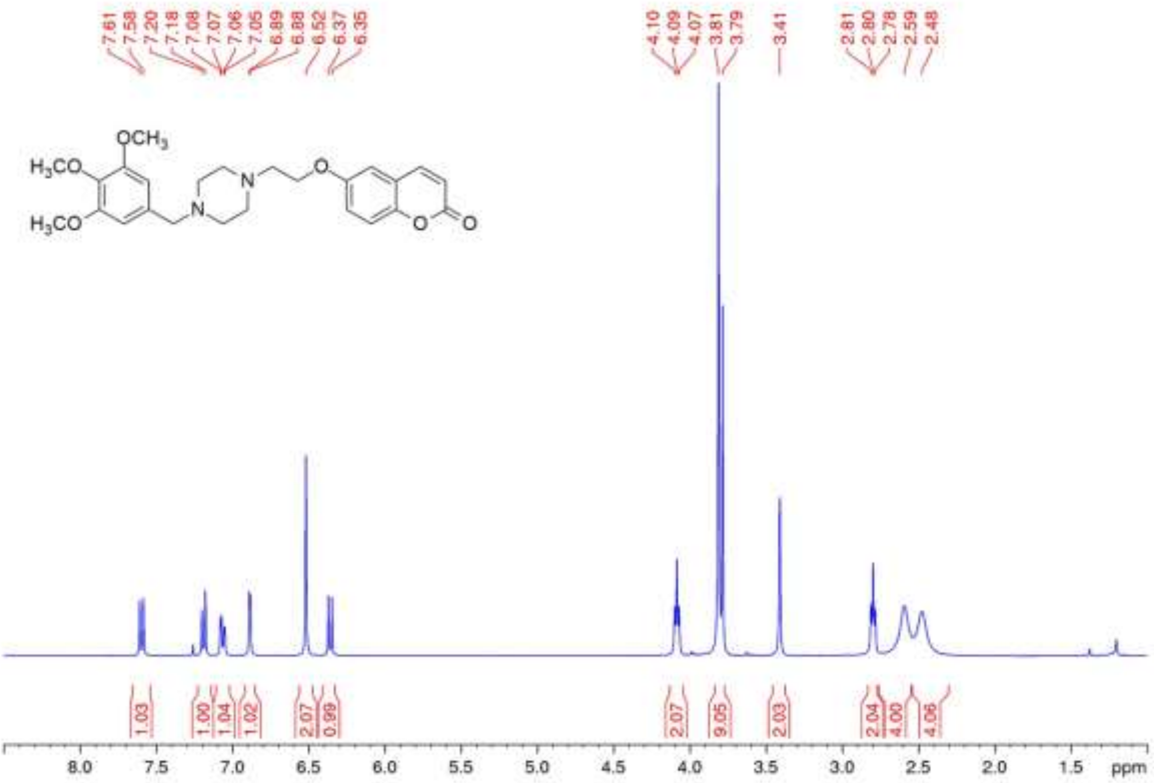


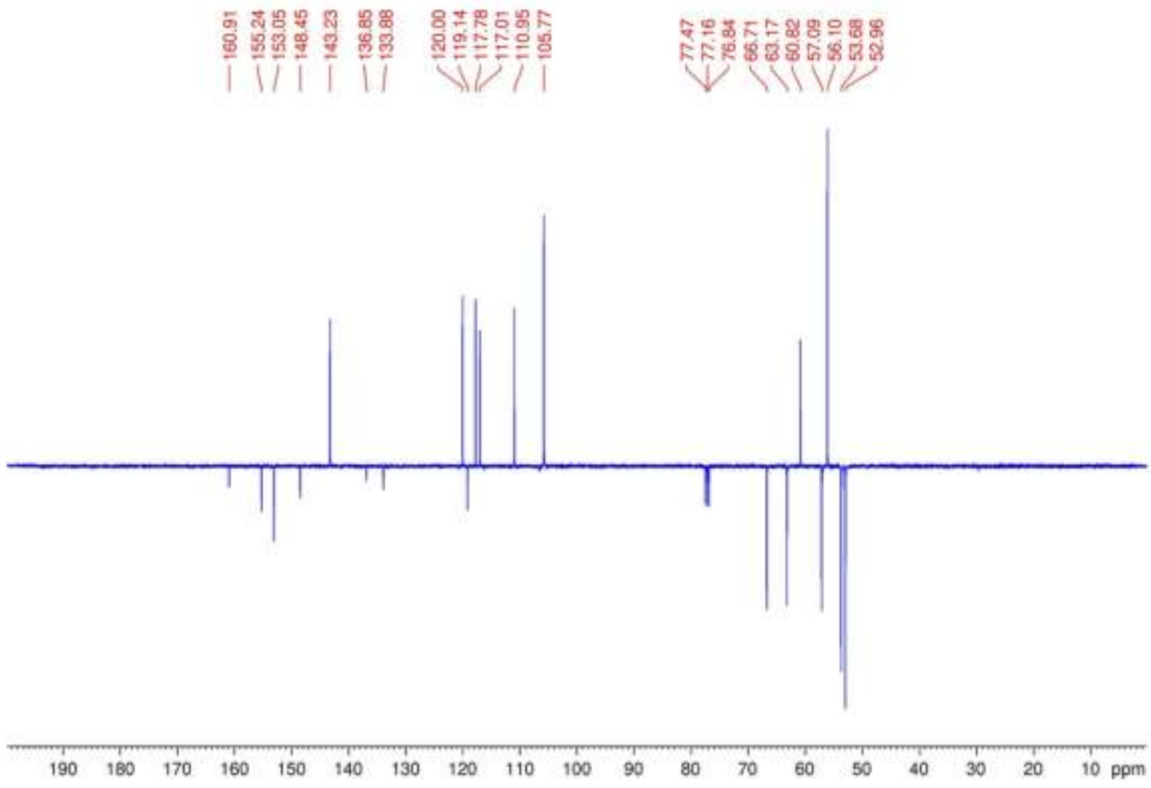

$^{1}$H-NMR and $^{13}$C-APT-NMR spectra of compound **20**

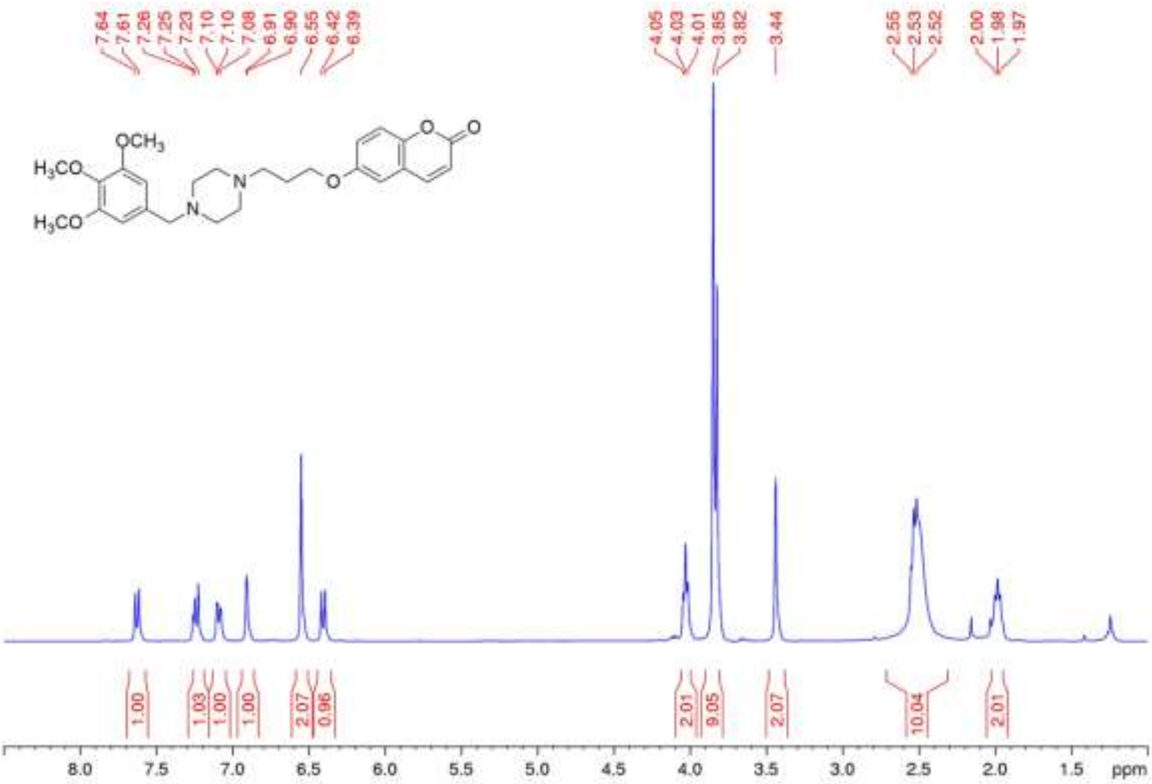


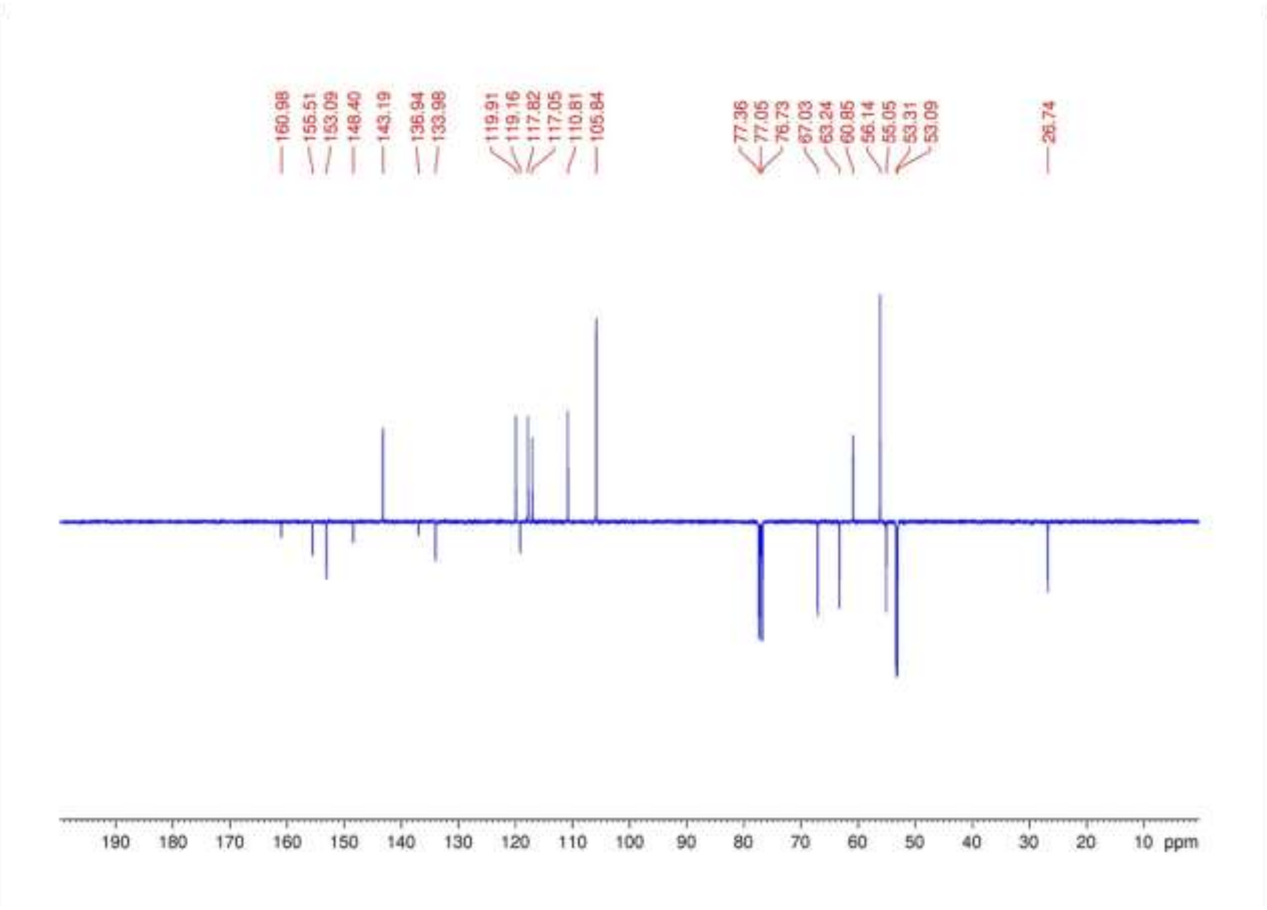

$^{1}$H-NMR and $^{13}$C-APT-NMR spectra of compound **21**

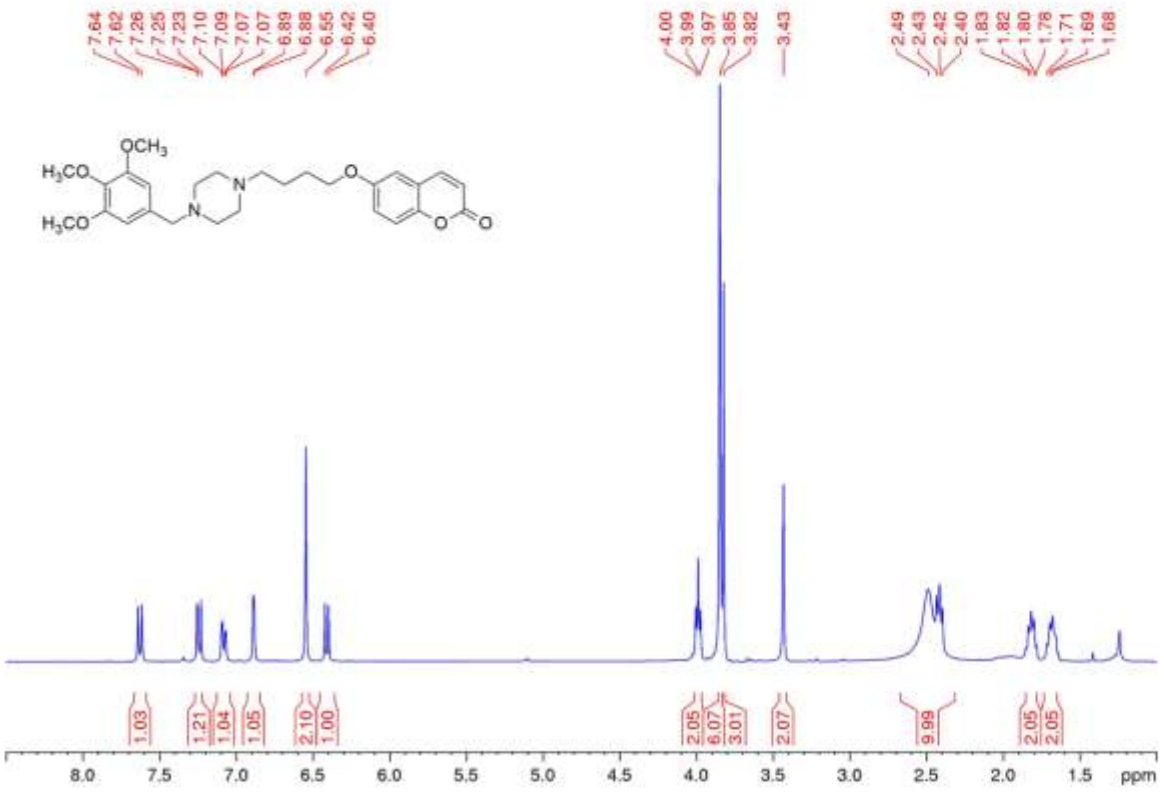

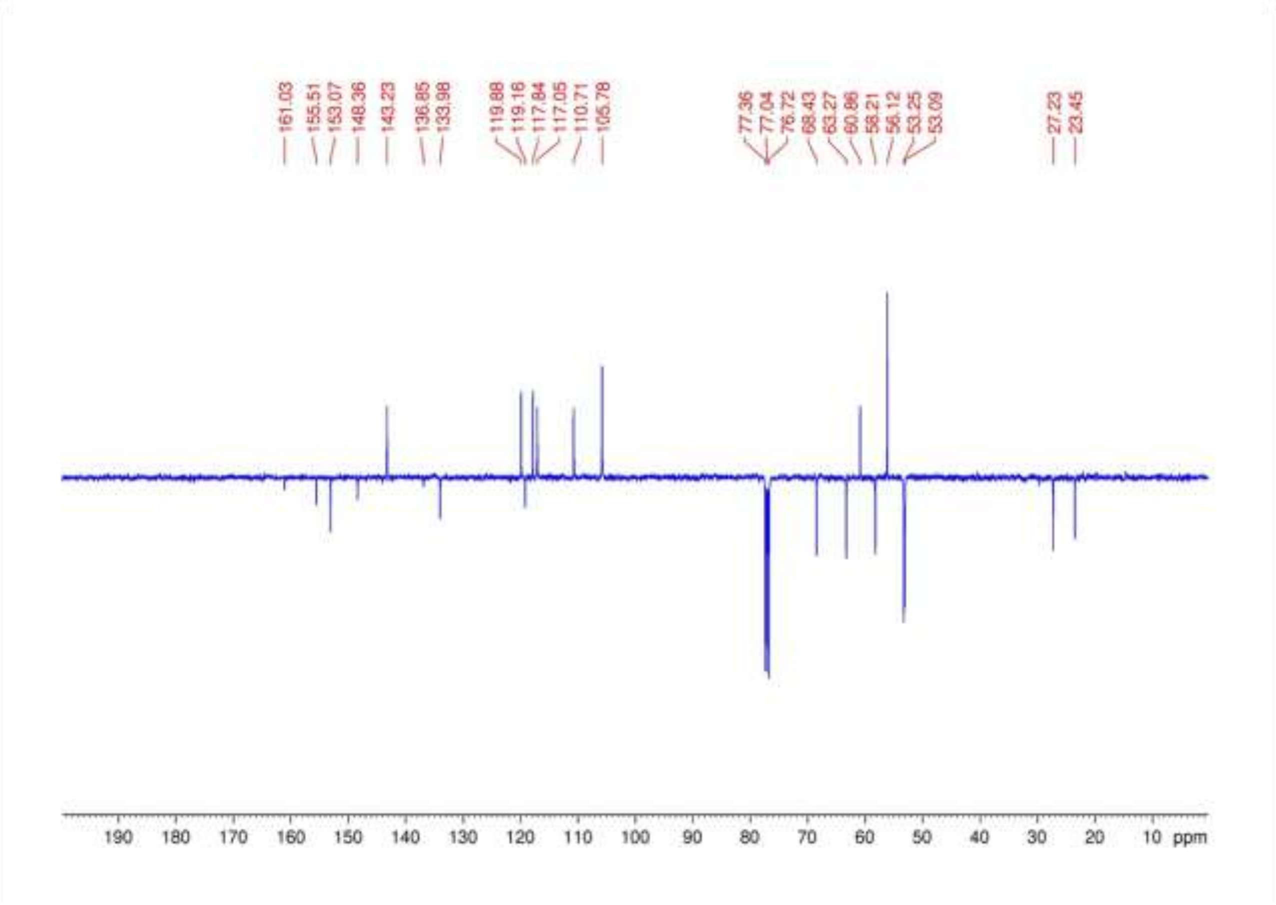

$^{1}$H-NMR and $^{13}$C-APT-NMR spectra of compound **22**

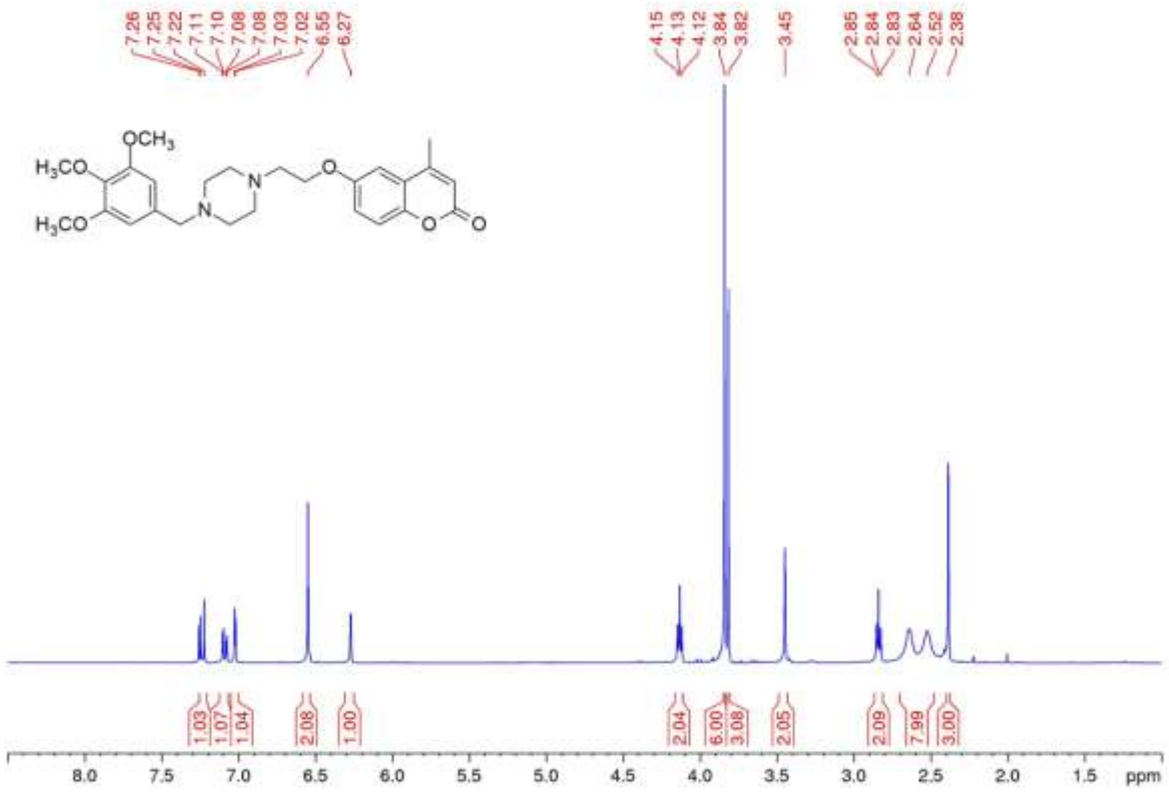


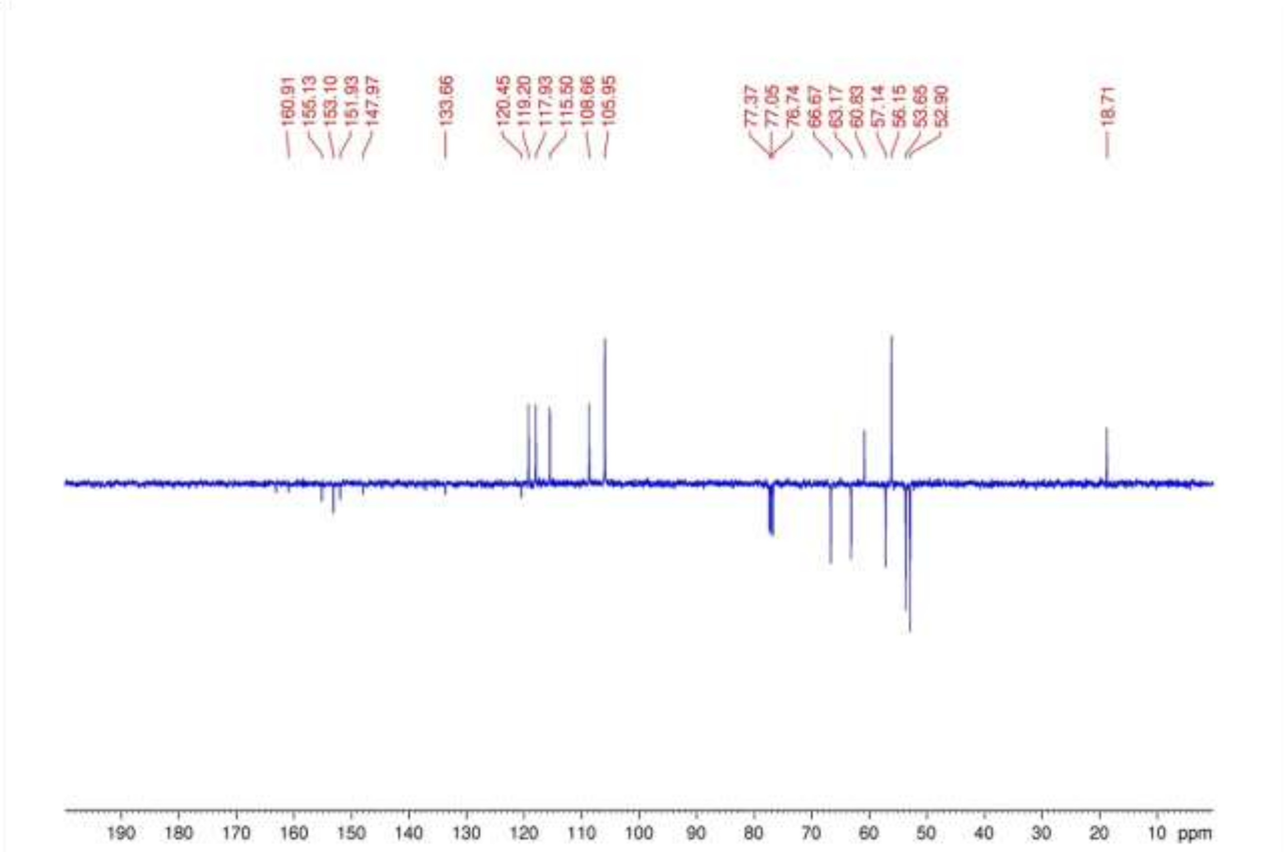

$^{1}$H-NMR and $^{13}$C-APT-NMR spectra of compound **23**

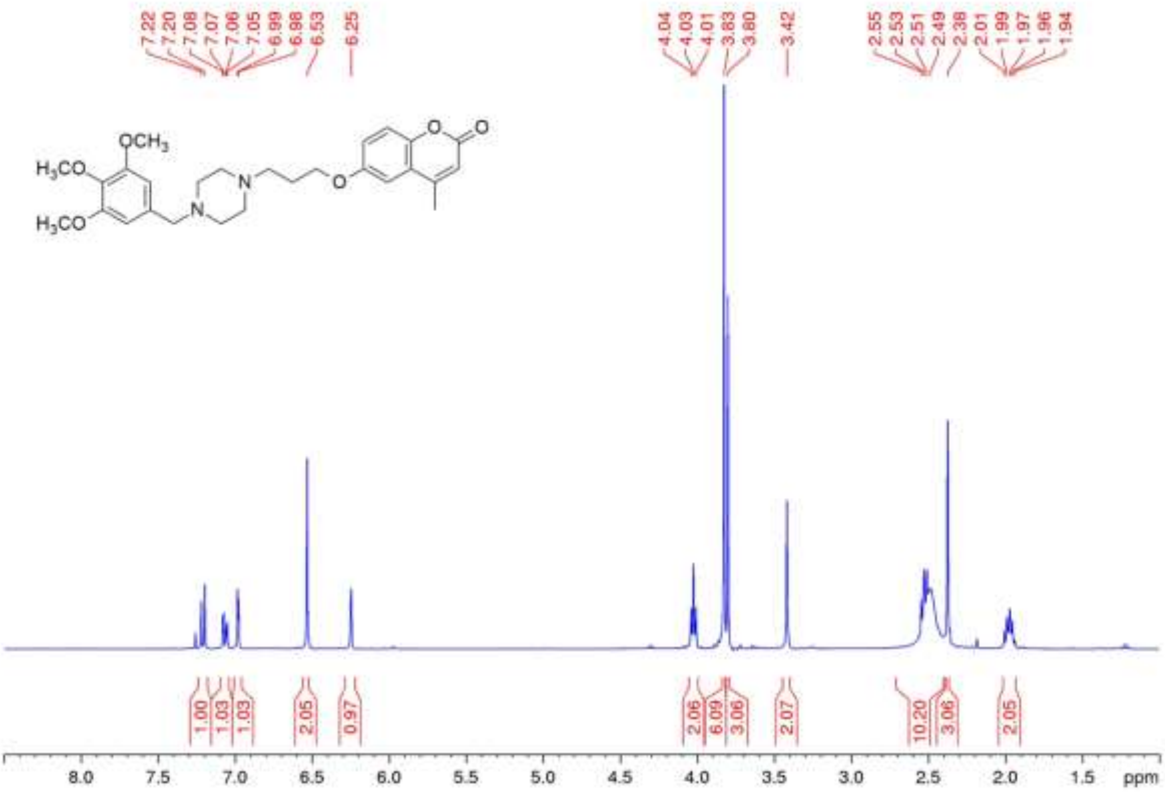


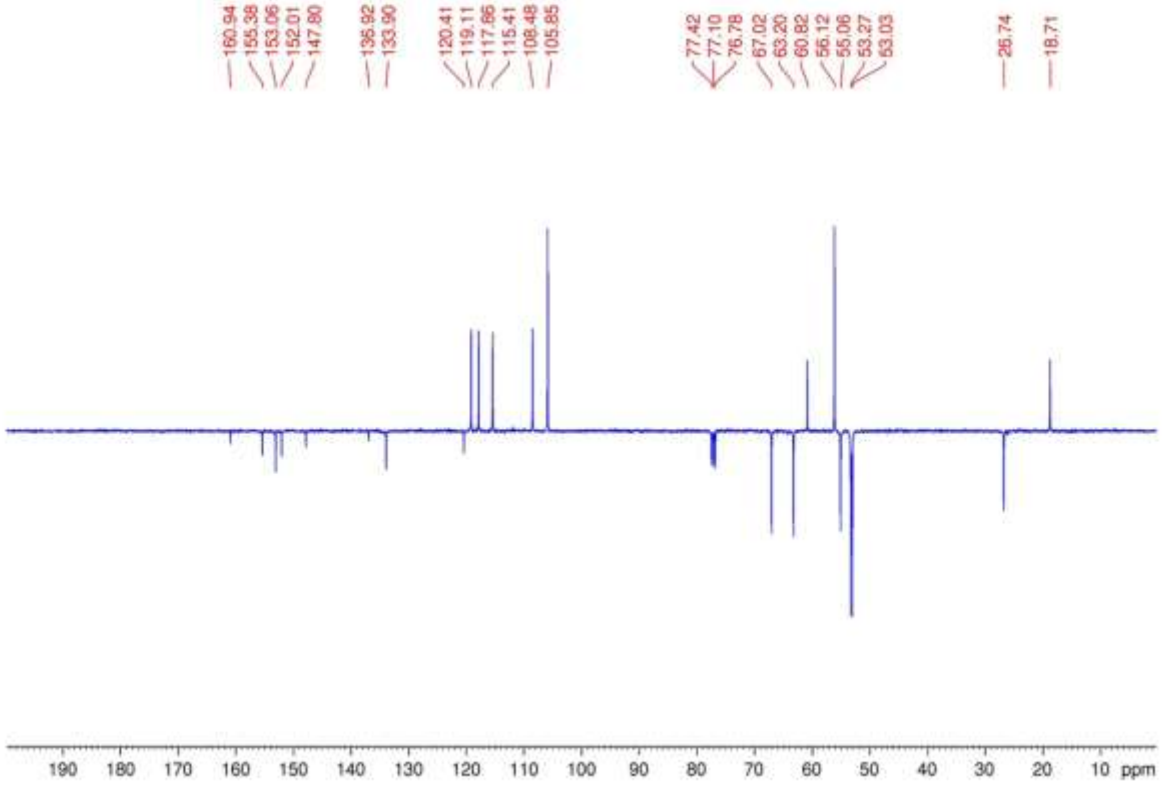

$^{1}$H-NMR and $^{13}$C-APT-NMR spectra of compound **24**

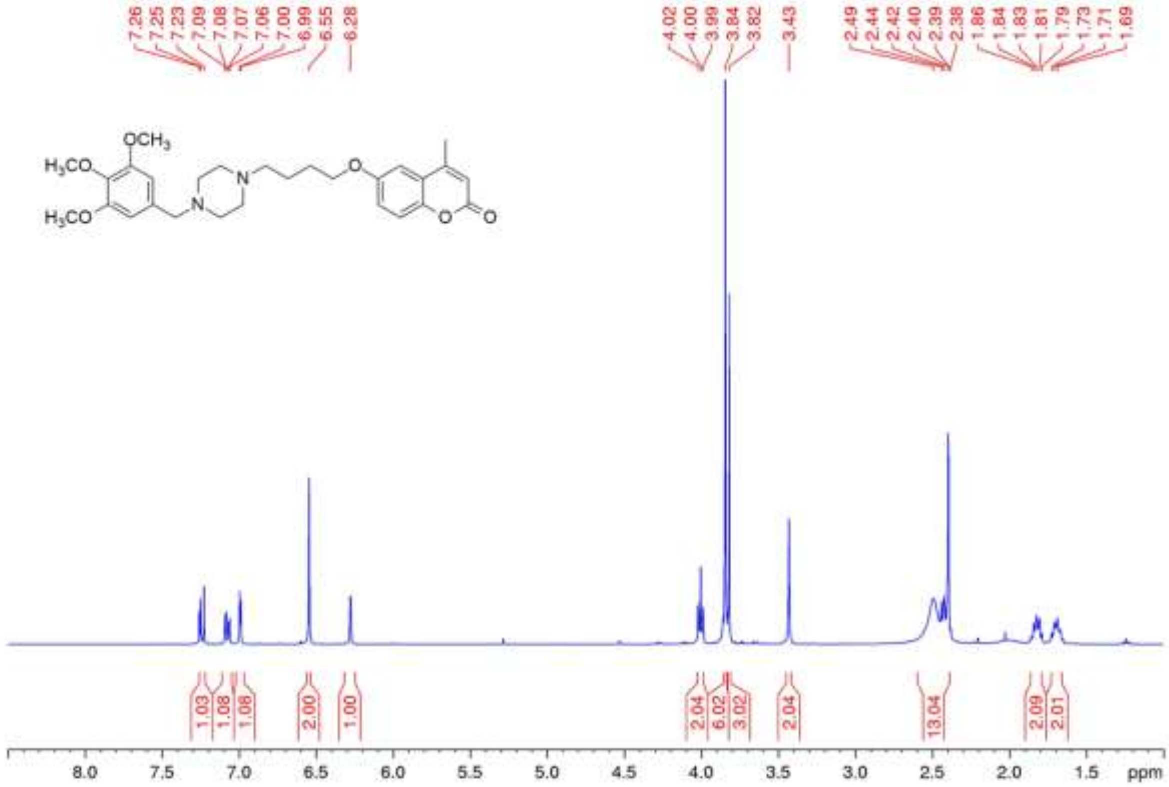


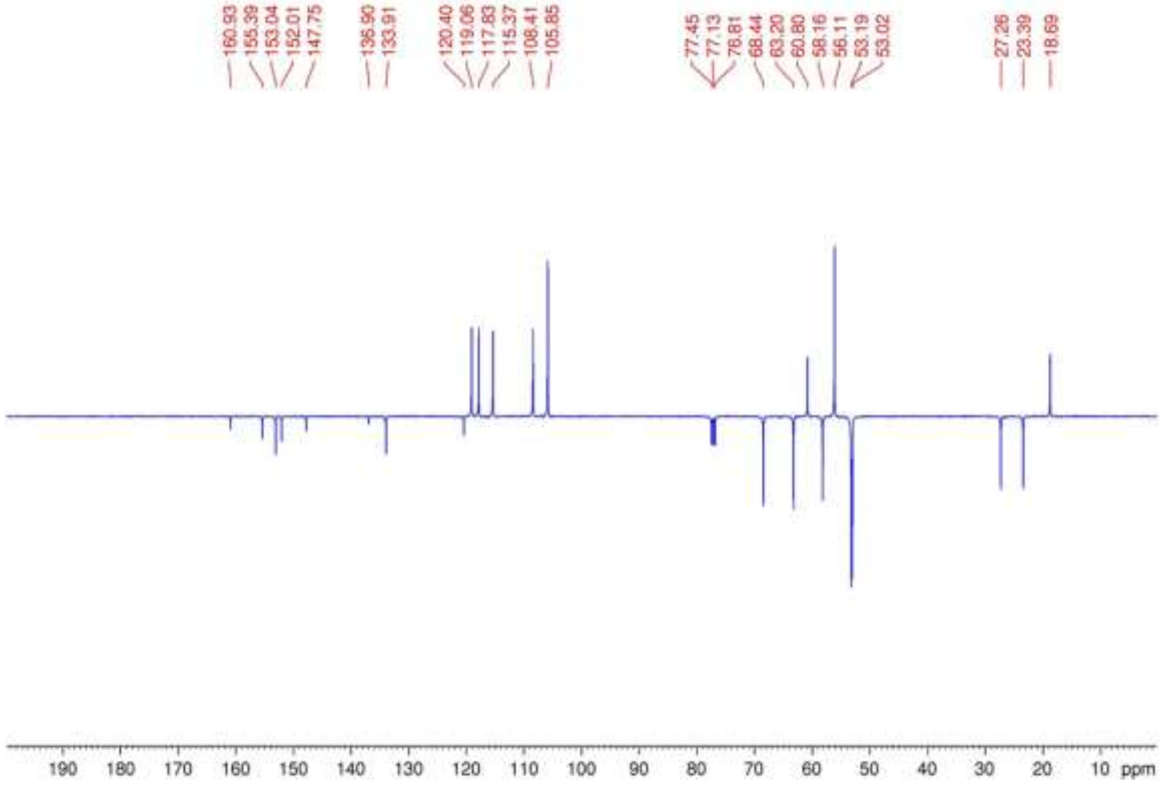

$^{1}$H-NMR and $^{13}$C-APT-NMR spectra of compound **25**

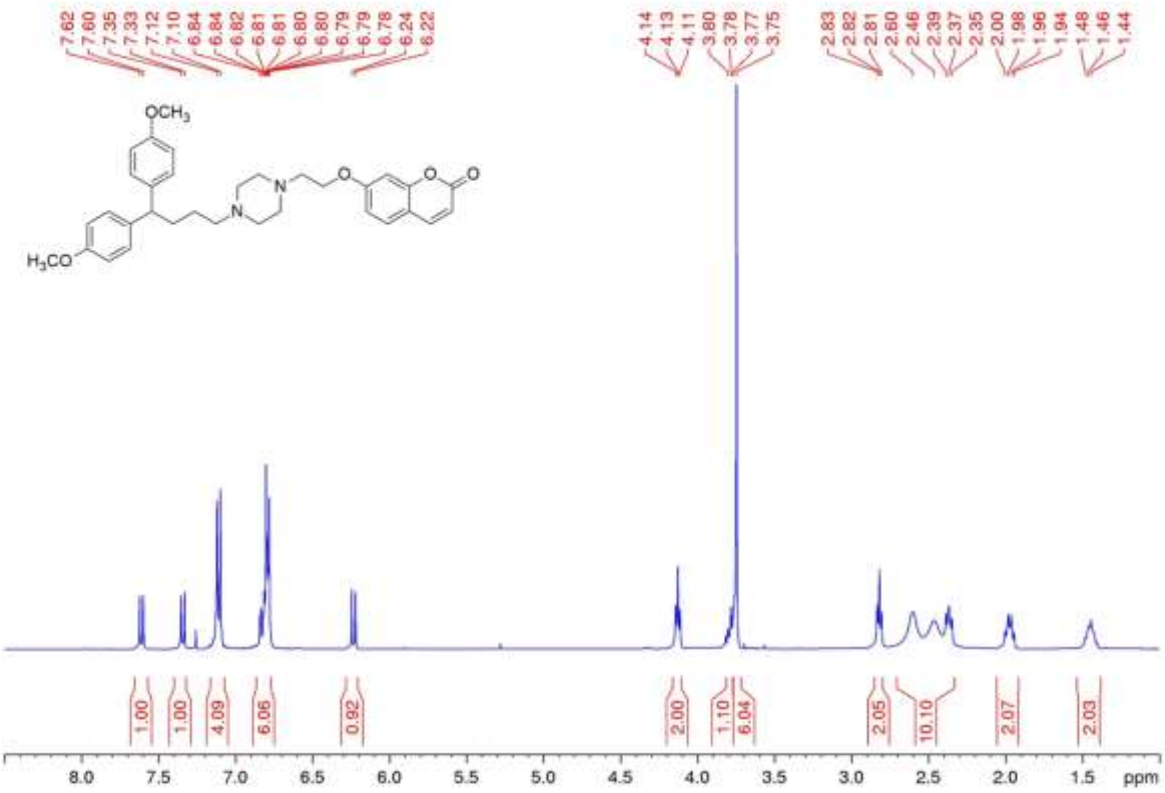


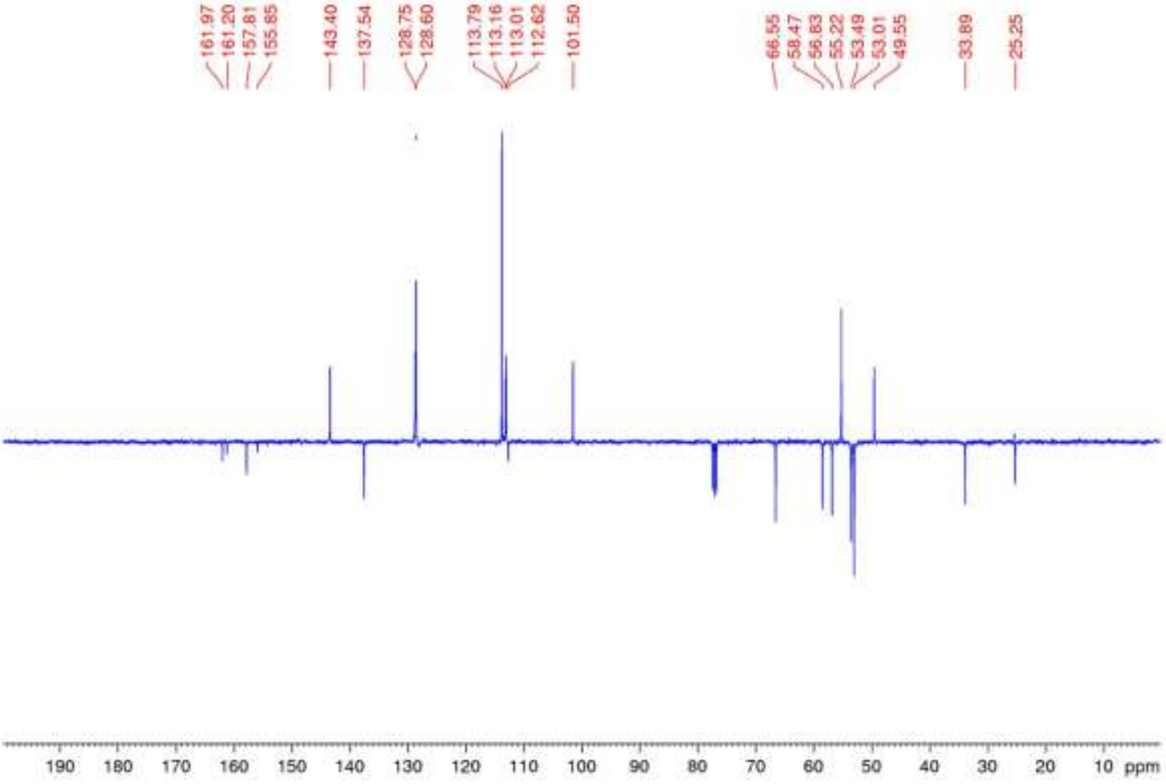

$^{1}$H-NMR and $^{13}$C-APT-NMR spectra of compound **26**

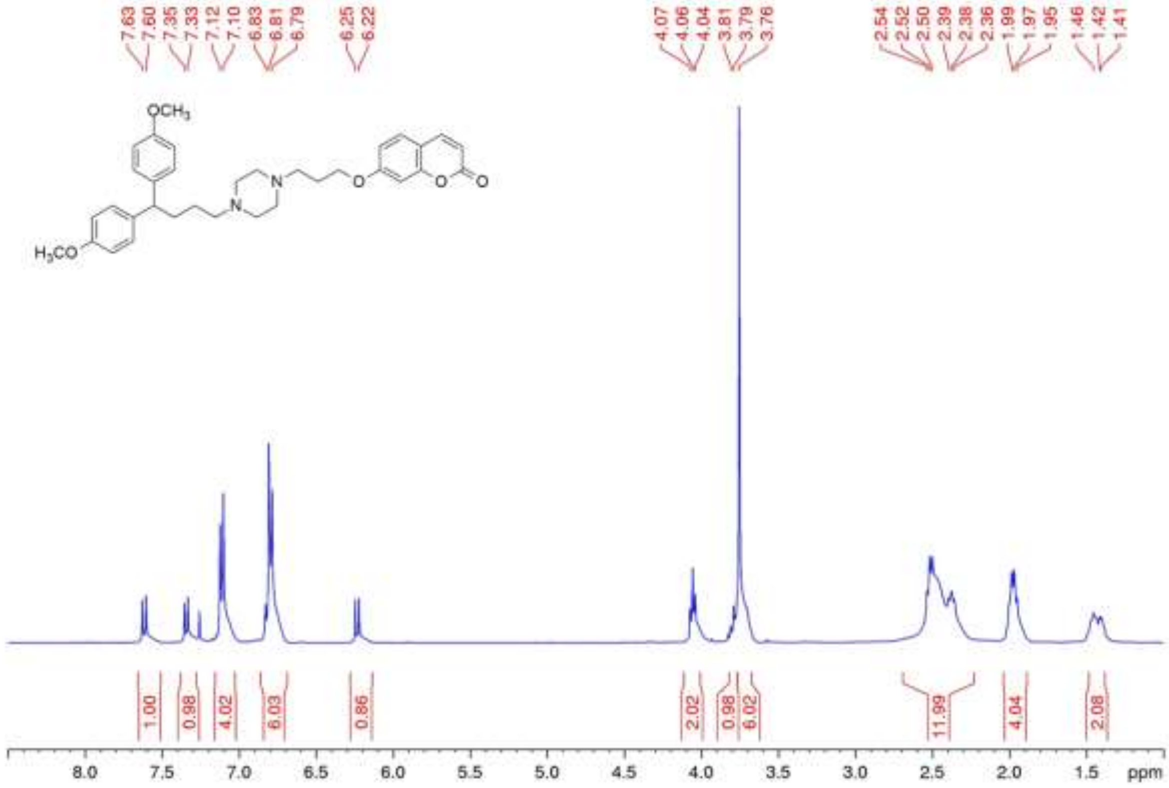


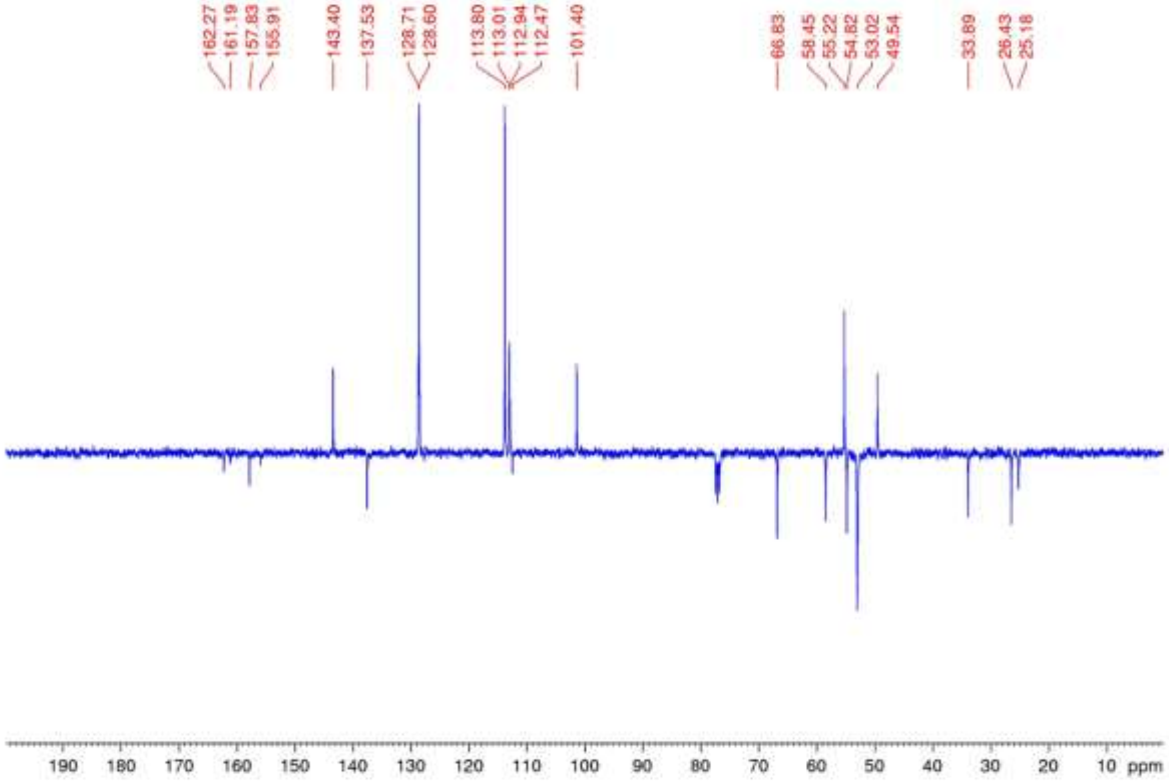

$^{1}$H-NMR and $^{13}$C-APT-NMR spectra of compound **27**

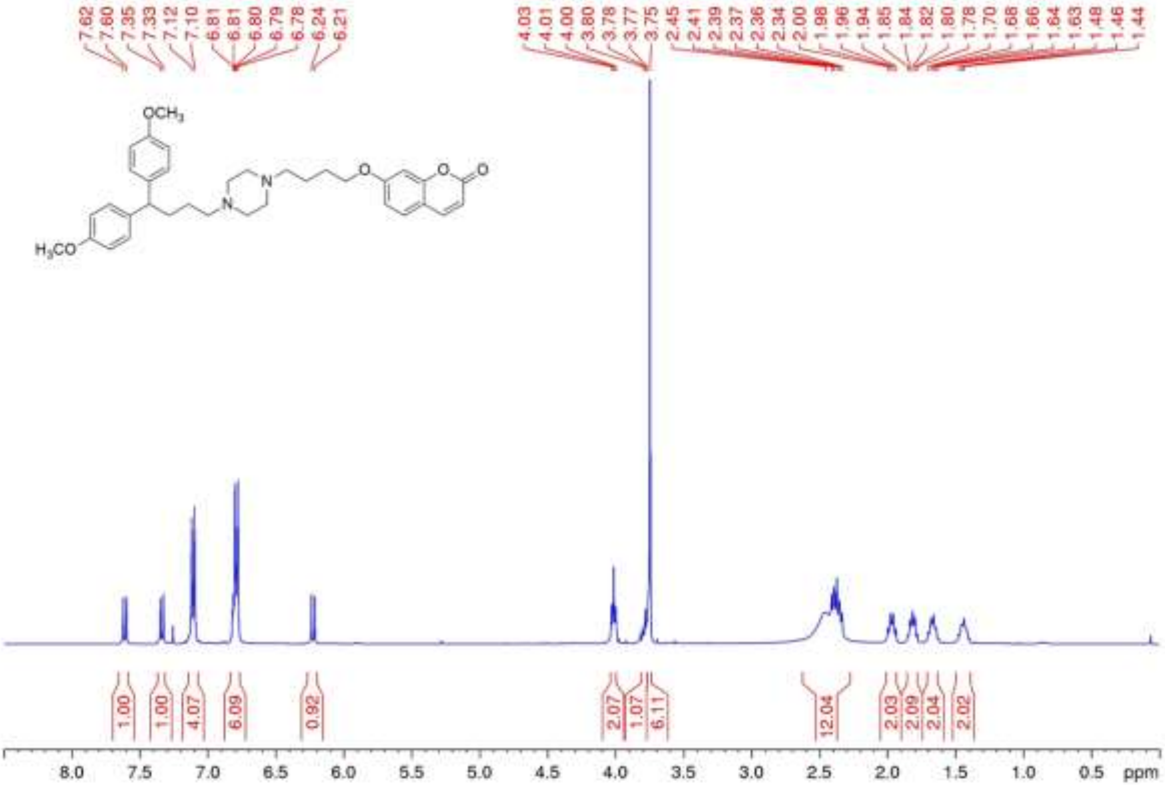


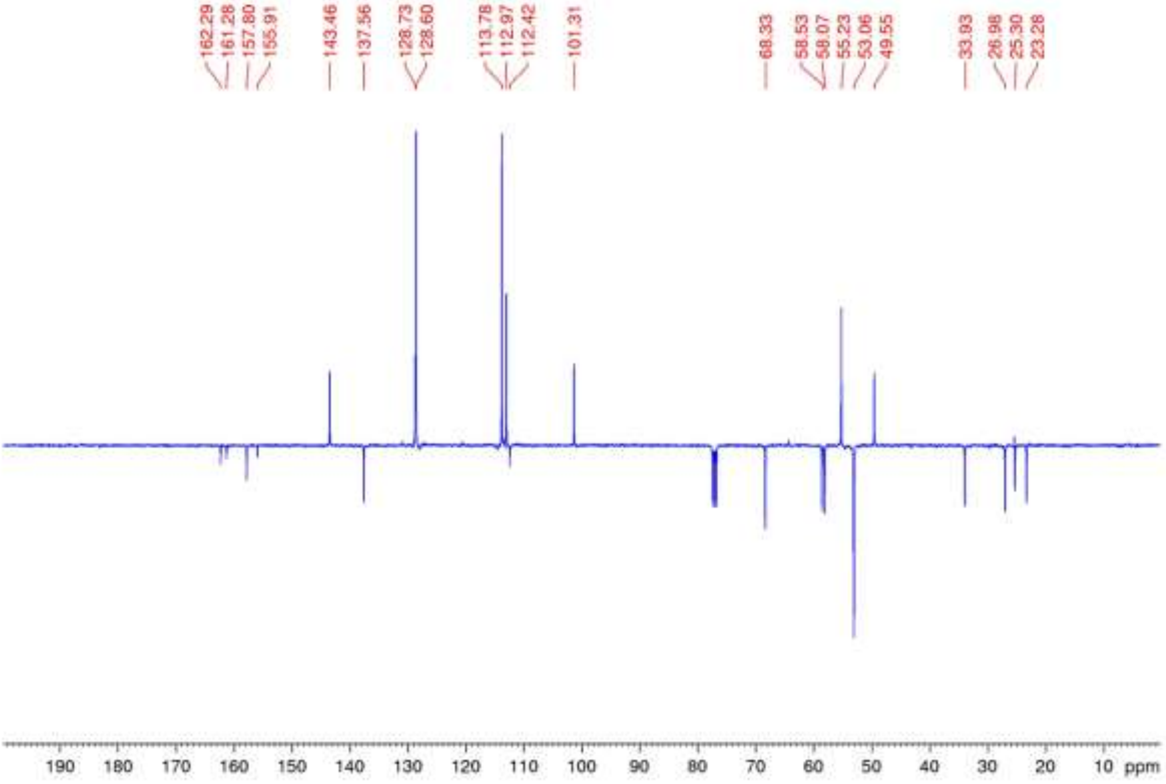

$^{1}$H-NMR and $^{13}$C-APT-NMR spectra of compound **28**

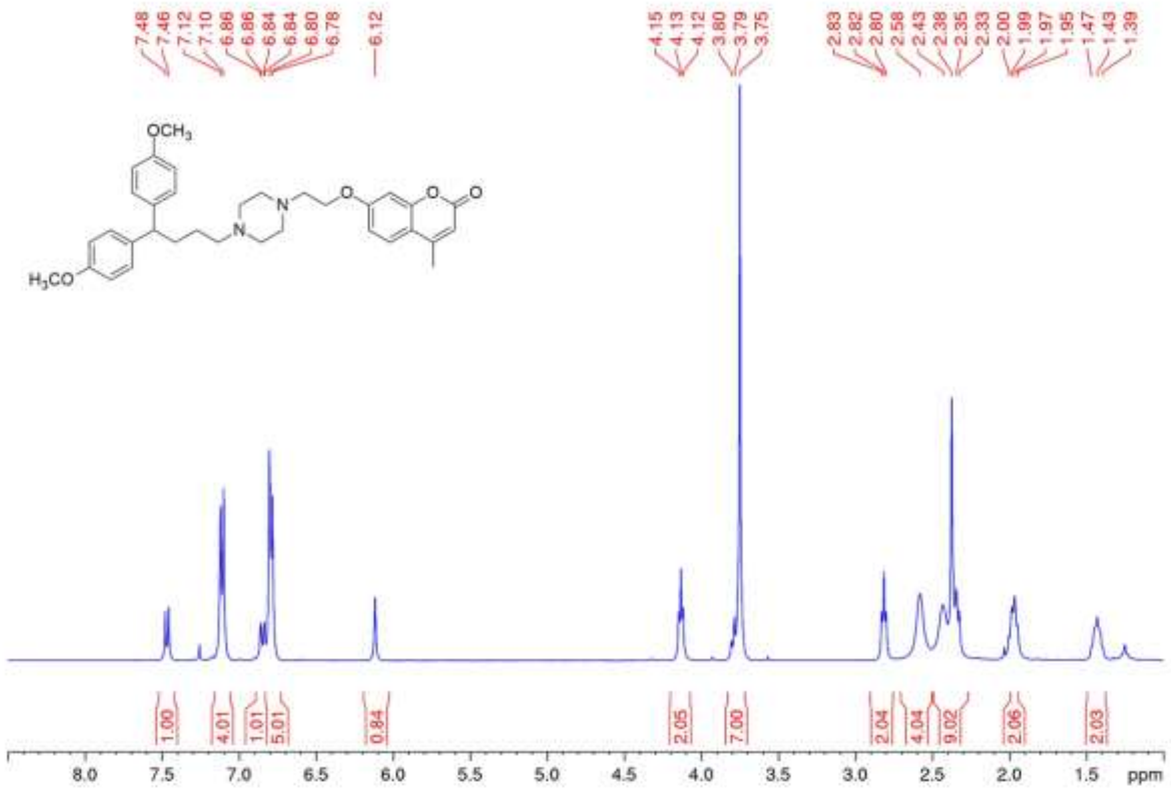


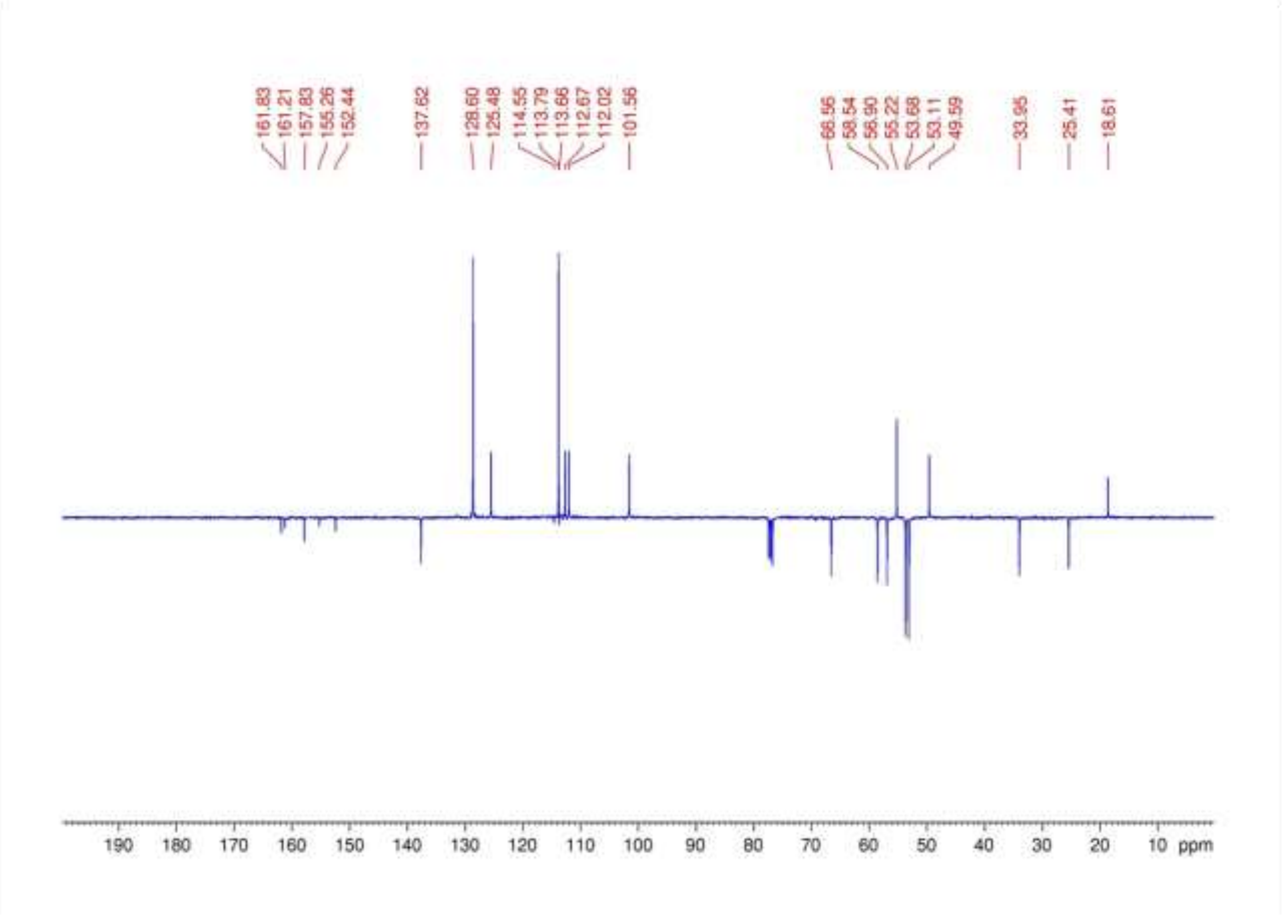

$^{1}$H-NMR and $^{13}$C-APT-NMR spectra of compound **29**

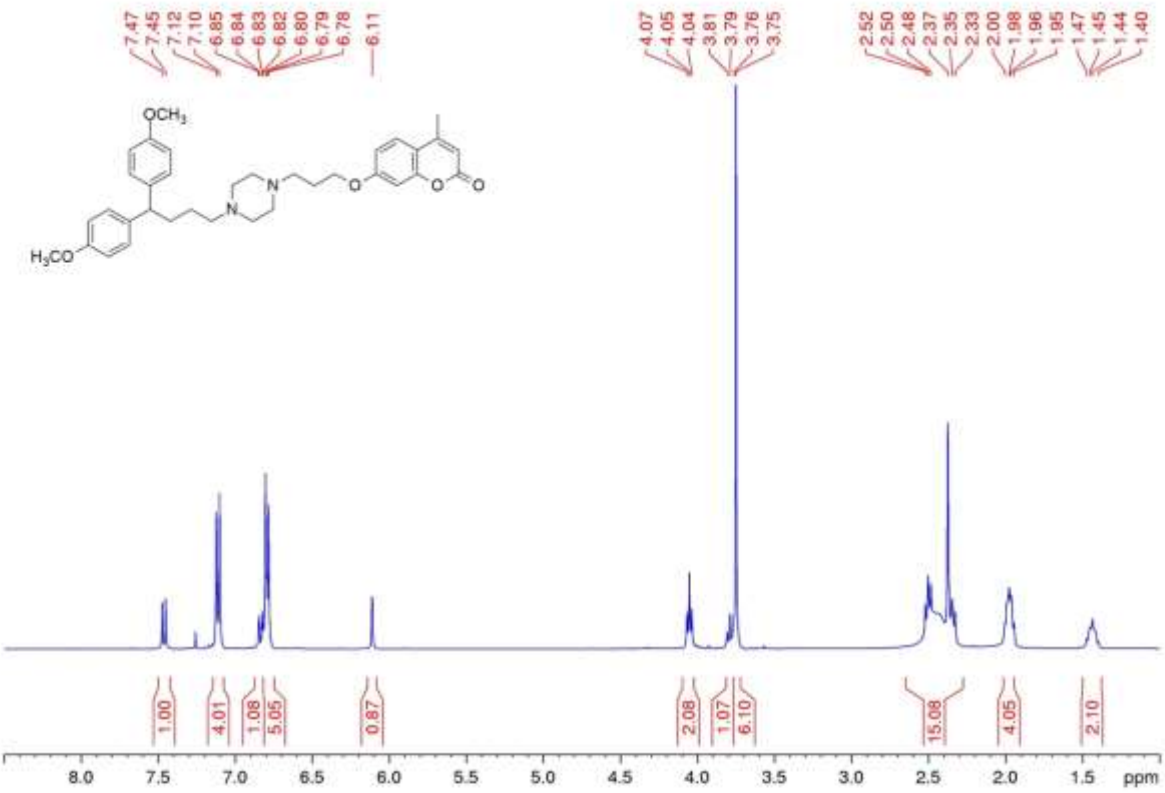

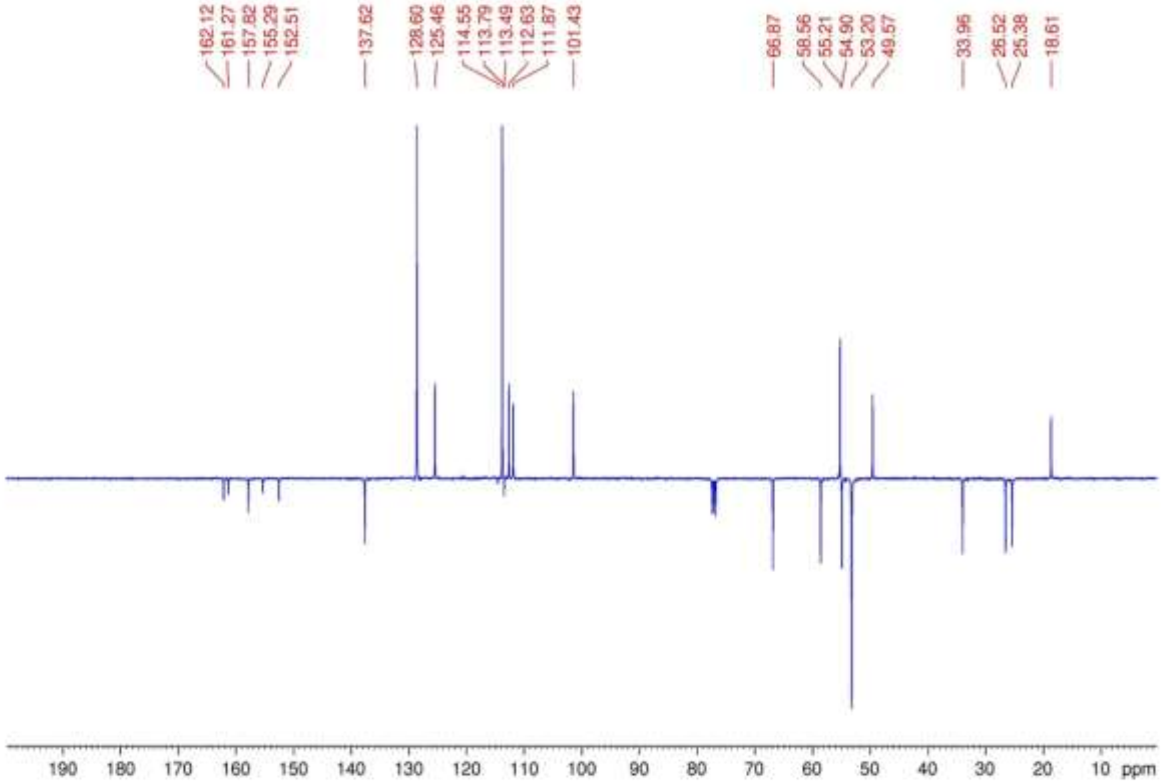

$^1$H-NMR and $^{13}$C-APT-NMR spectra of compound **30**

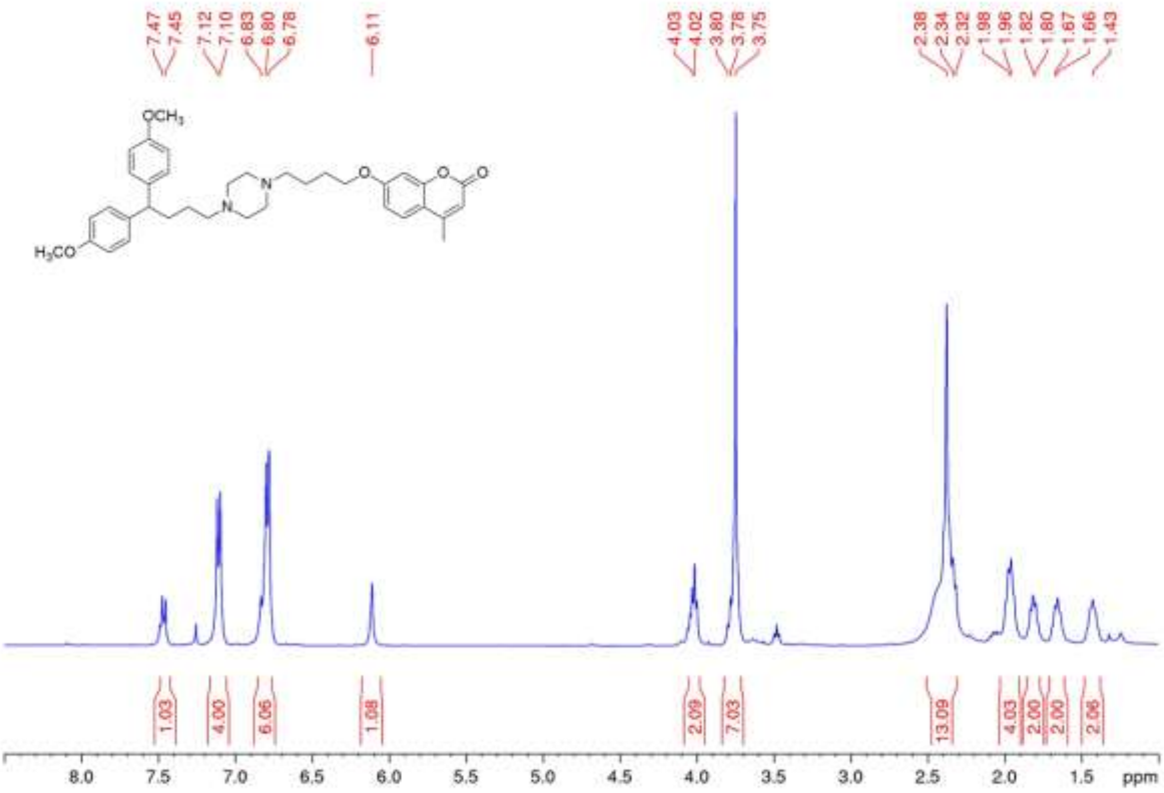


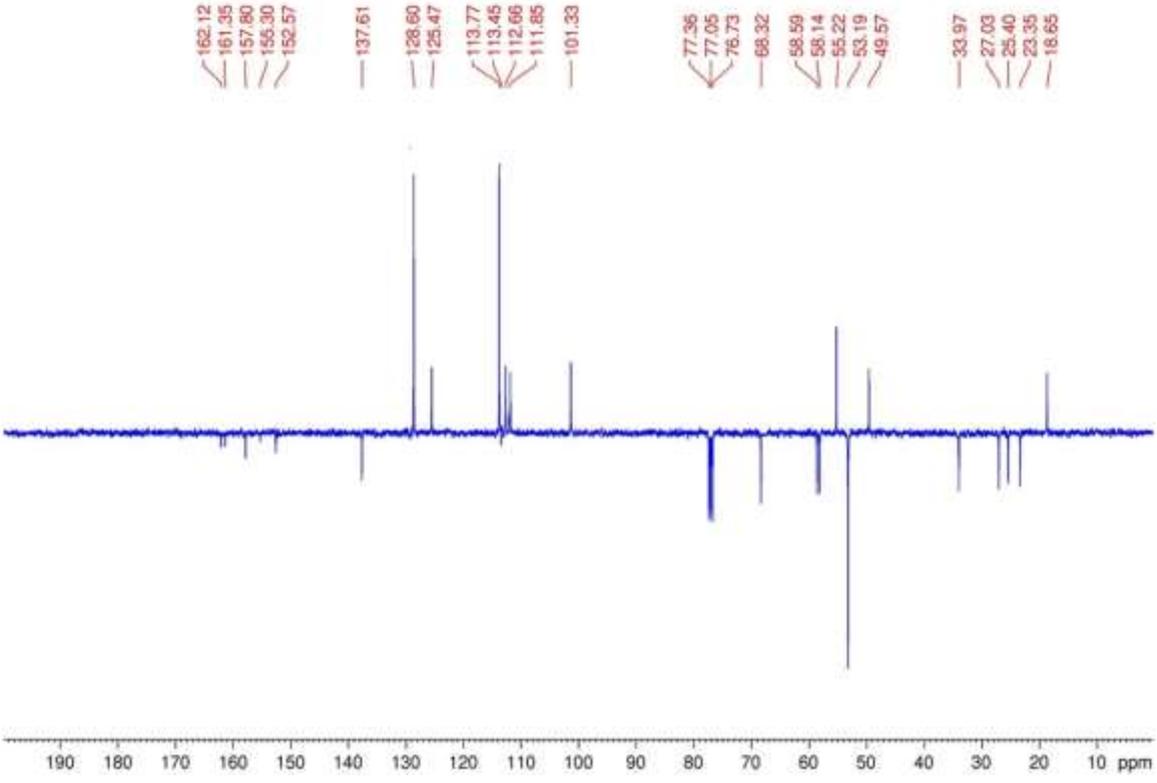

$^{1}$H-NMR and $^{13}$C-APT-NMR spectra of compound **31**

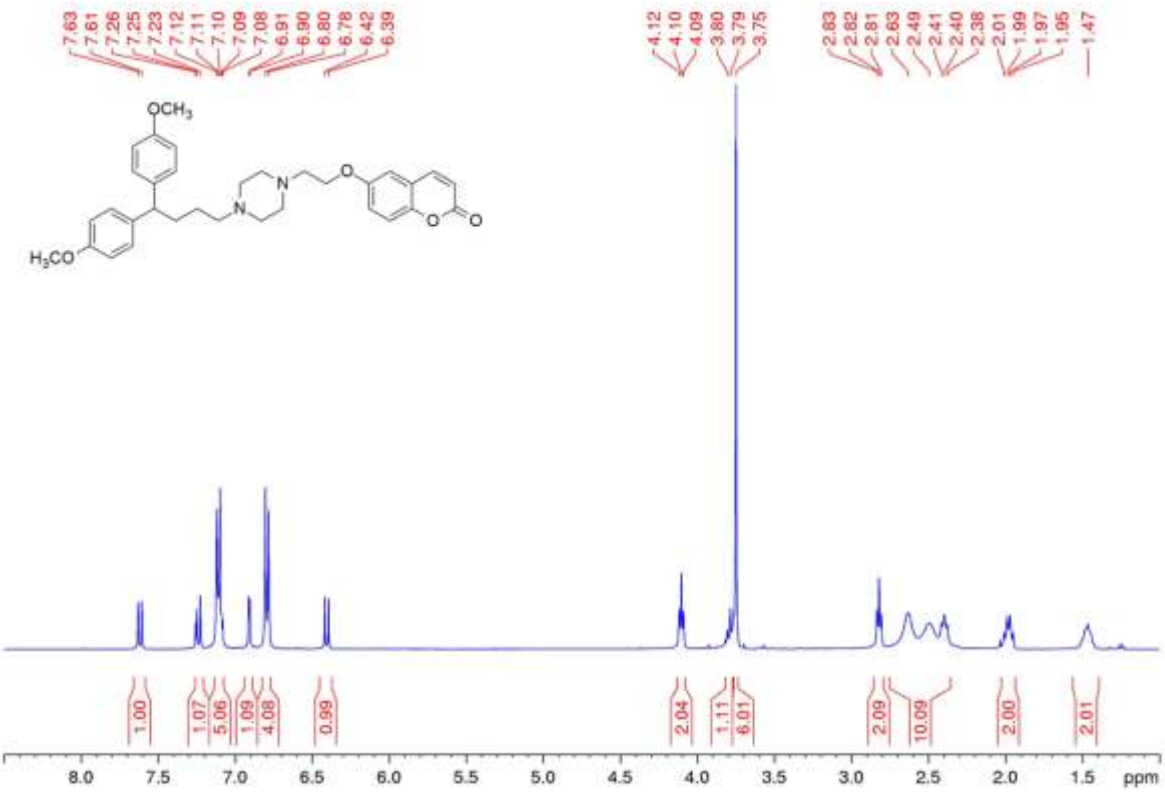


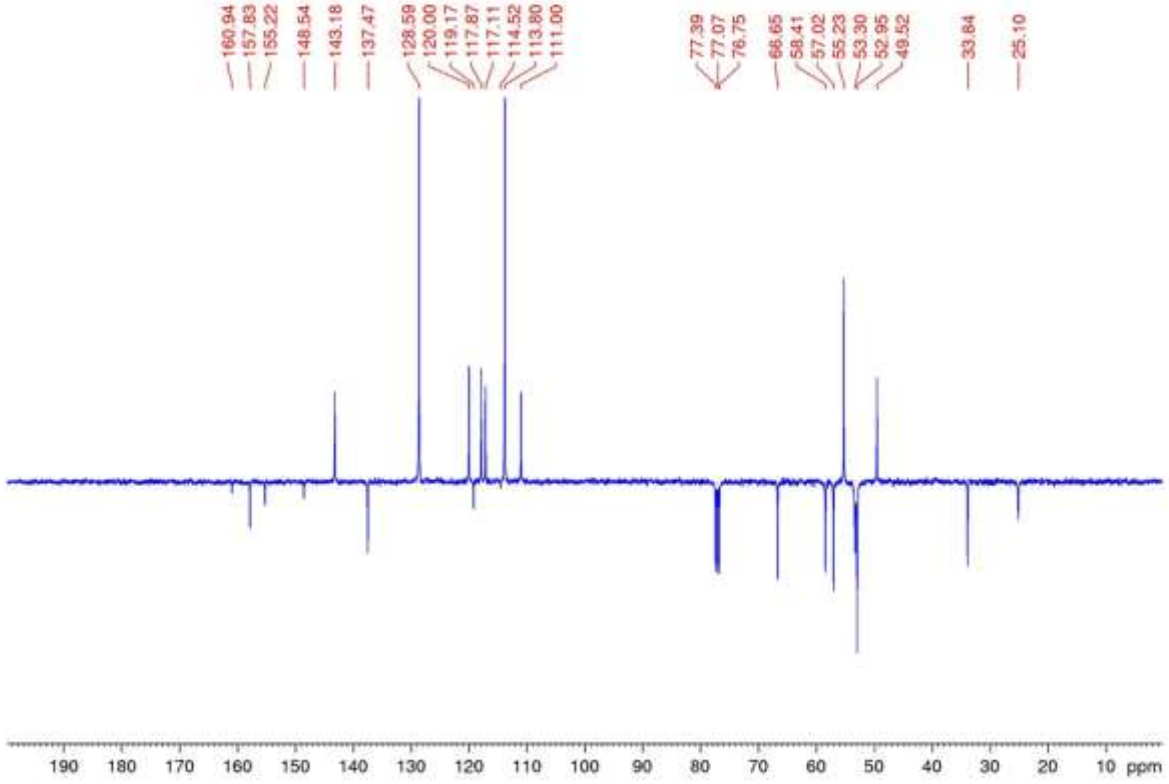

$^{1}H$-NMR and $^{13}C$-APT-NMR spectra of compound **32**

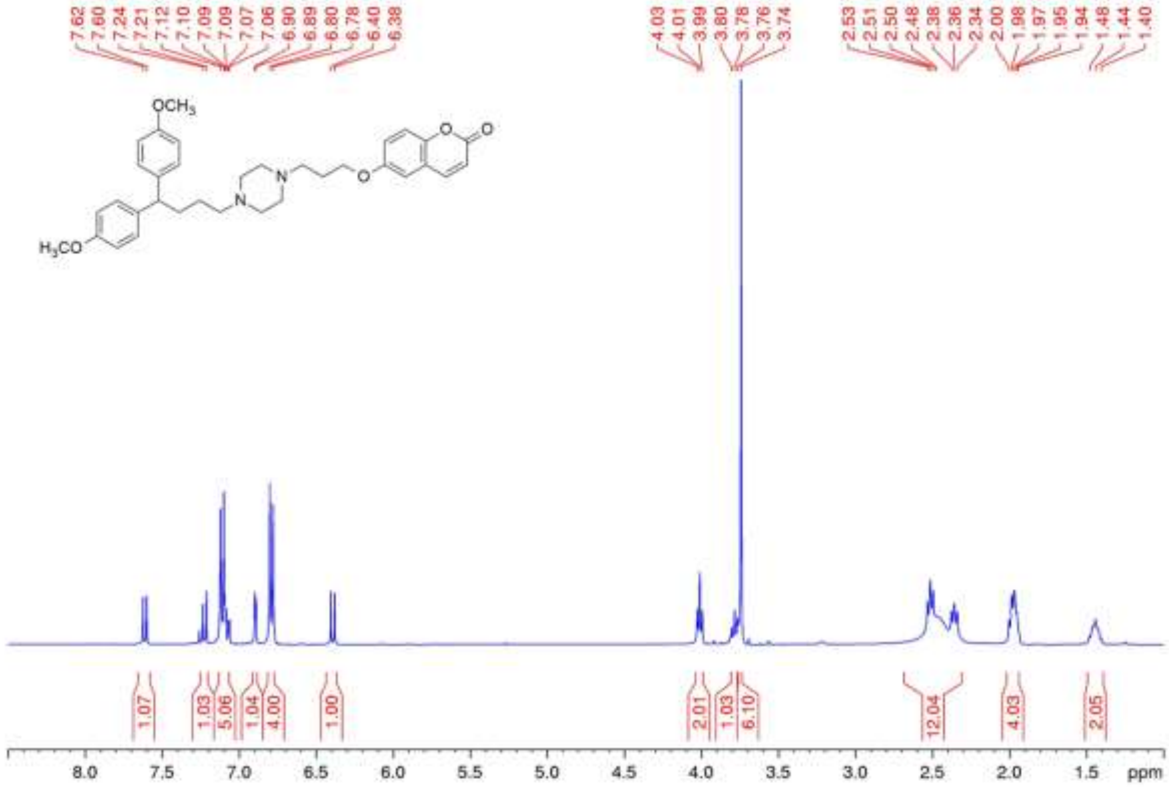


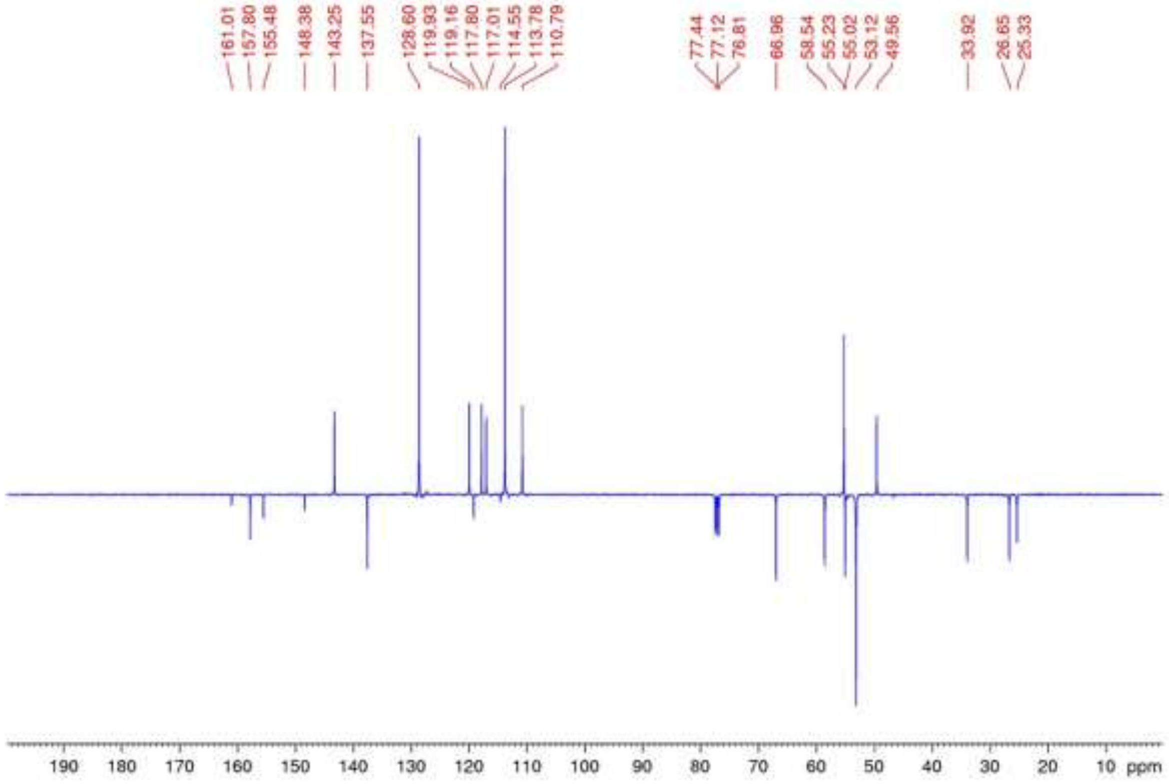

$^{1}H$-NMR and $^{13}C$-APT-NMR spectra of compound **33**

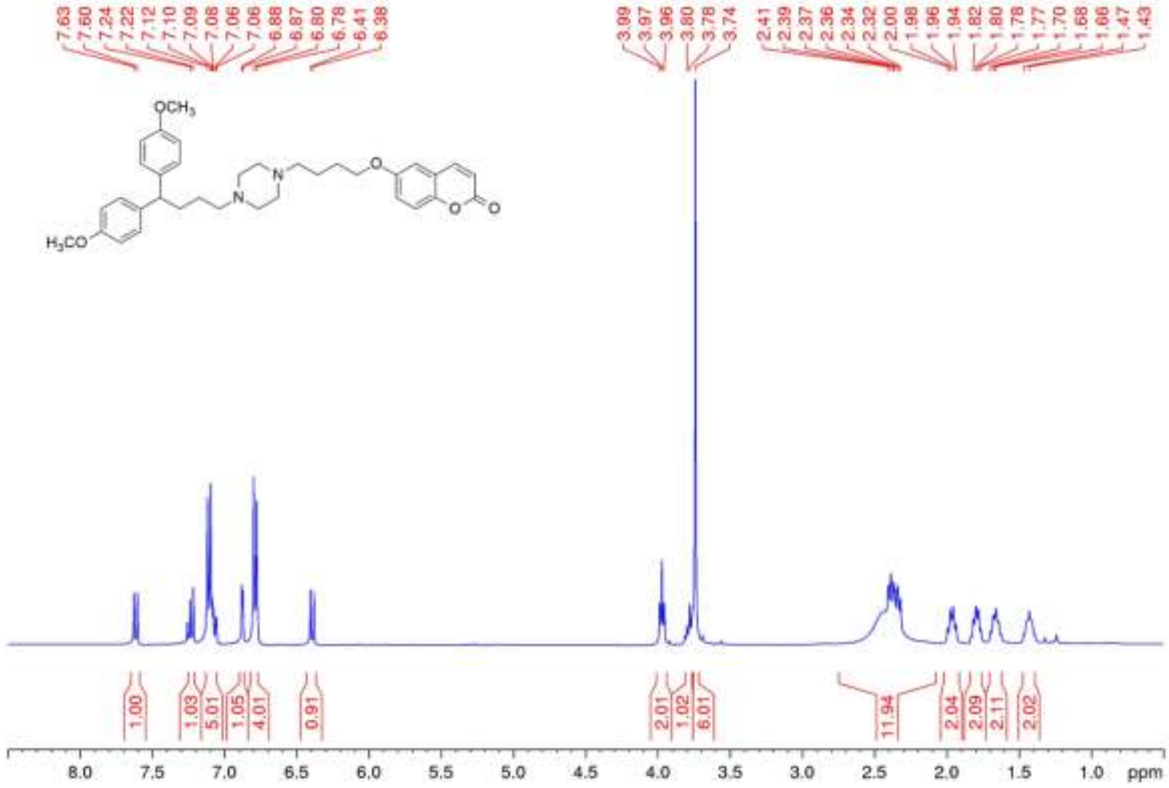


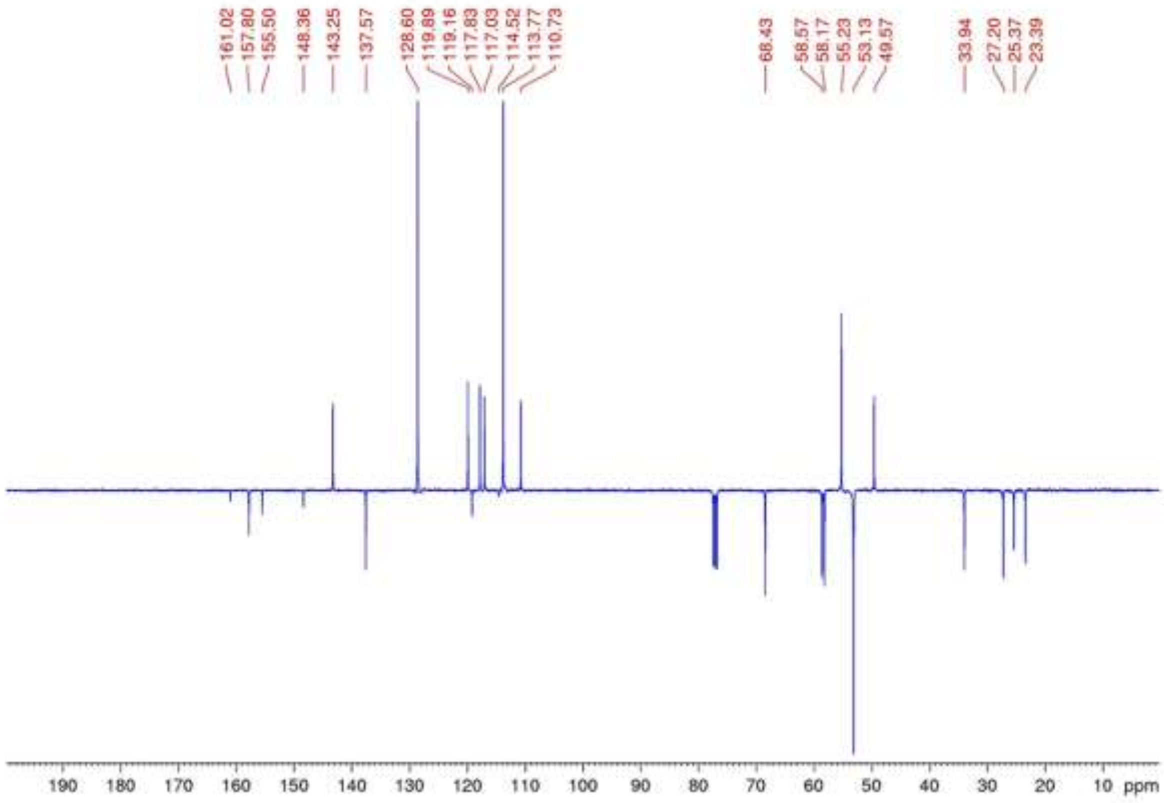

$^{1}$H-NMR and $^{13}$C-APT-NMR spectra of compound **34**

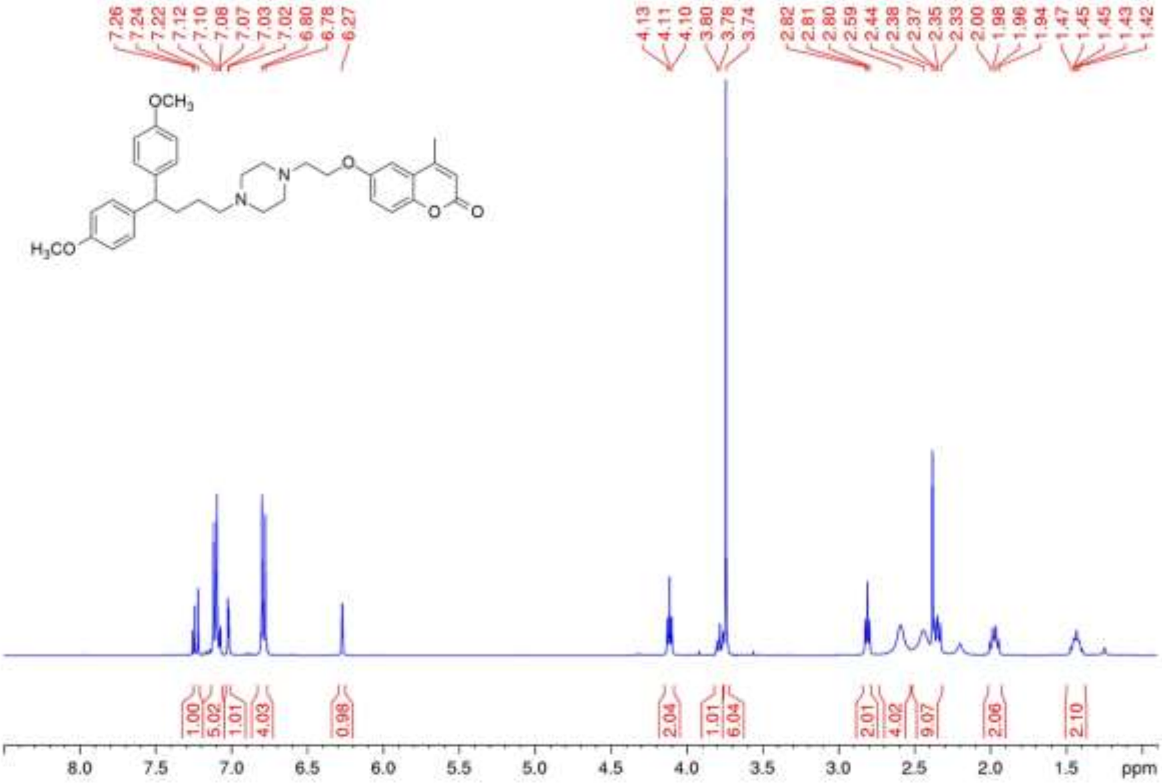


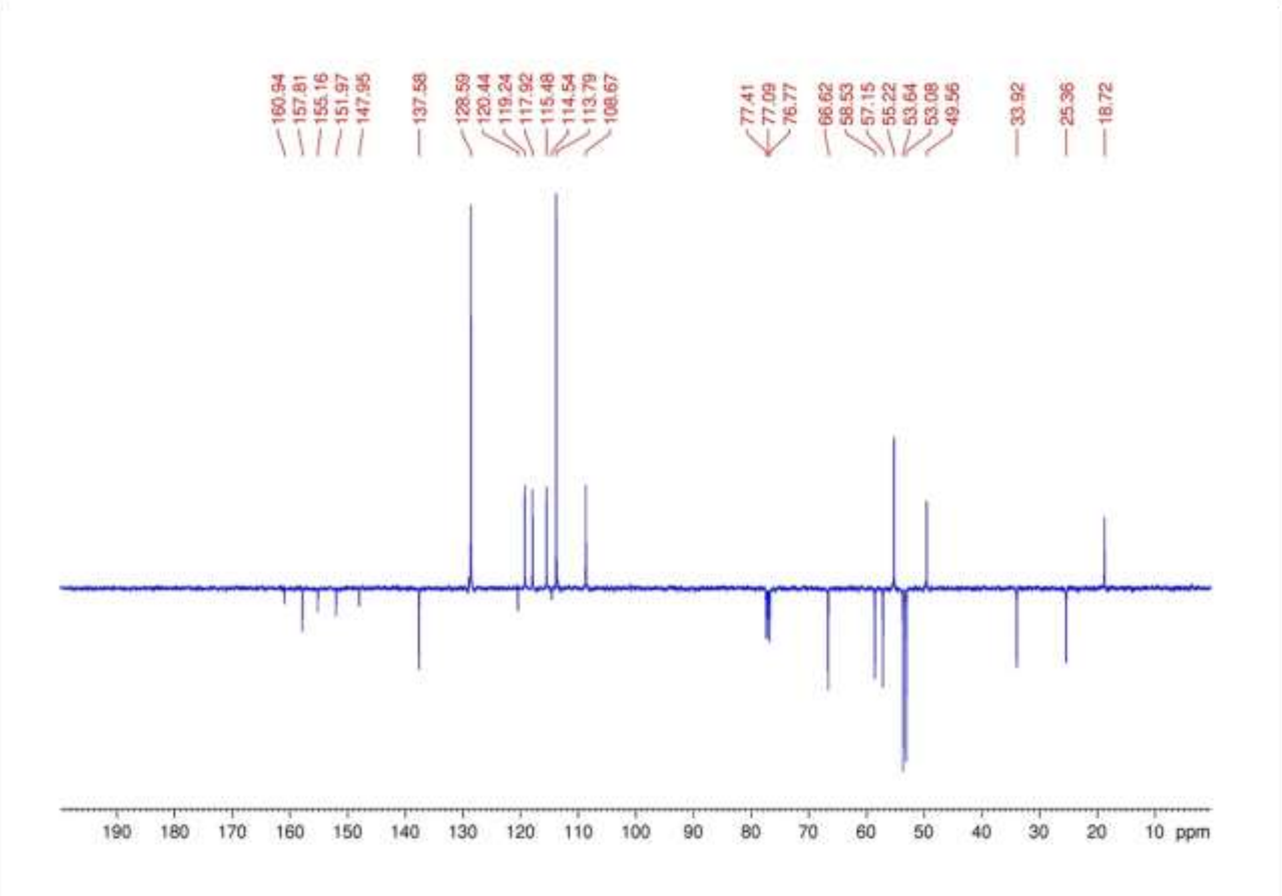

$^{1}$H-NMR and $^{13}$C-APT-NMR spectra of compound **35**

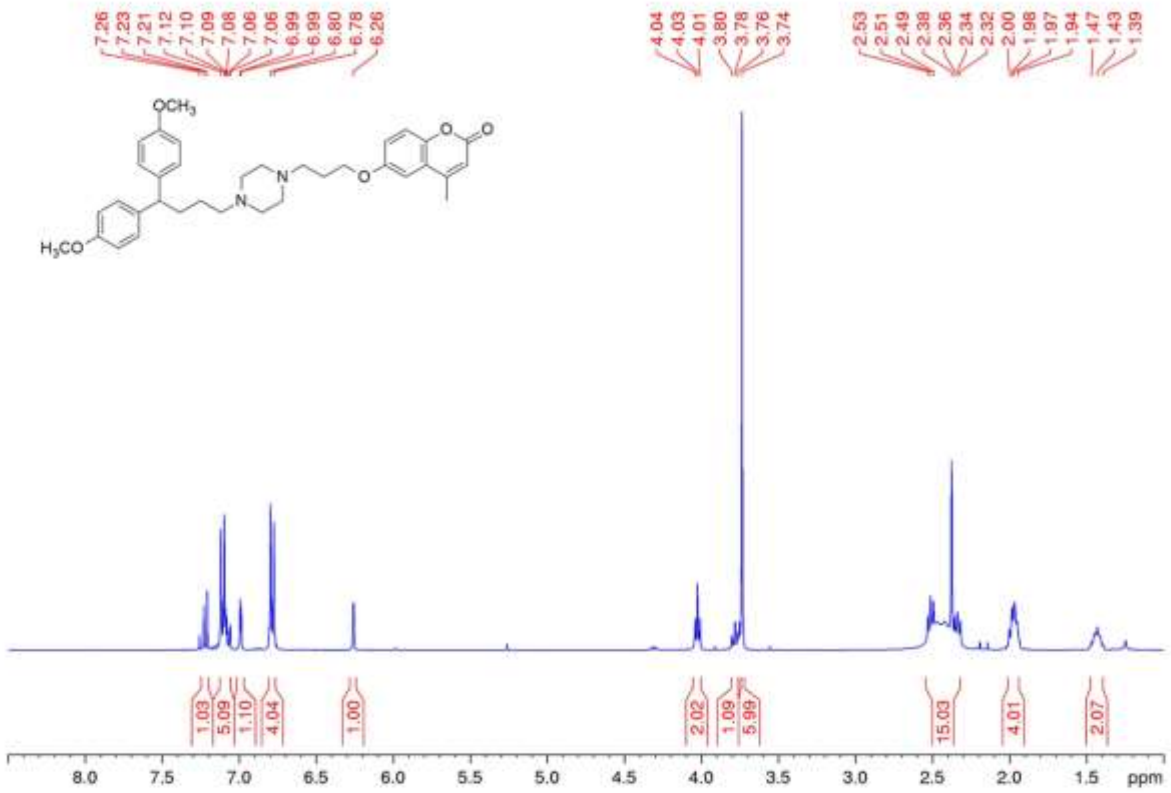


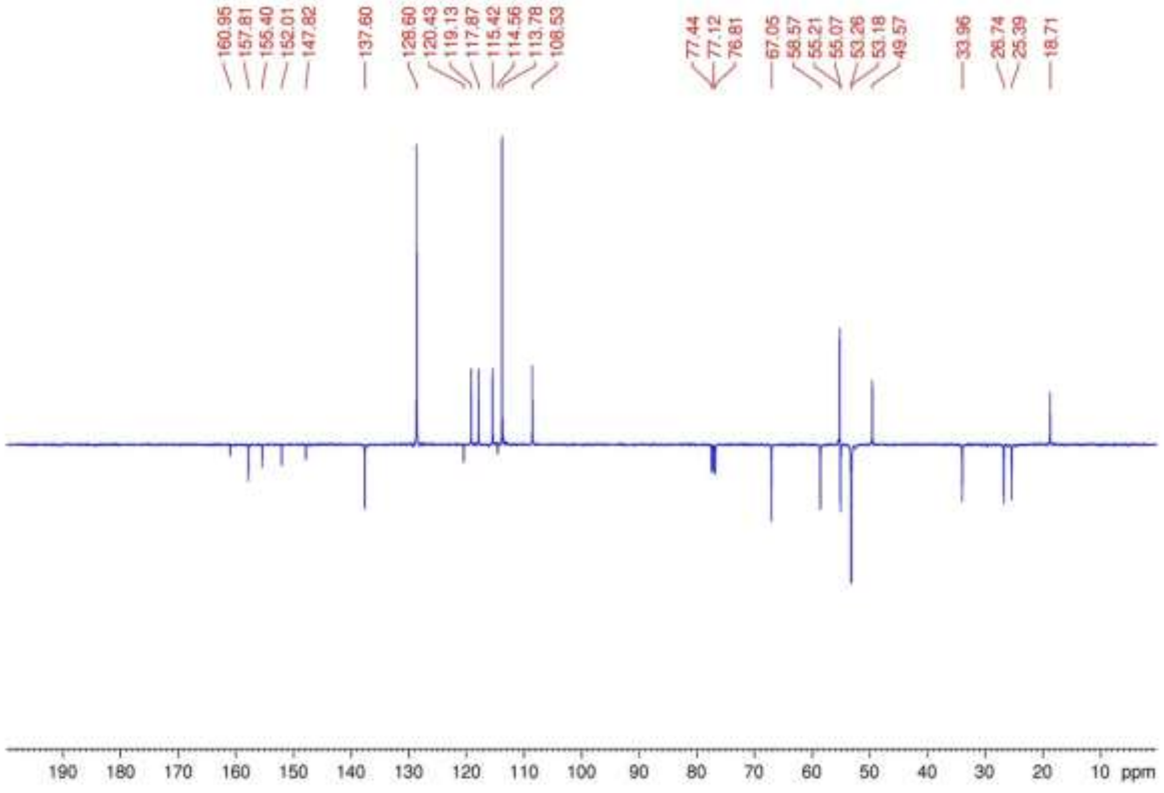

$^{1}$H-NMR and $^{13}$C-APT-NMR spectra of compound **36**

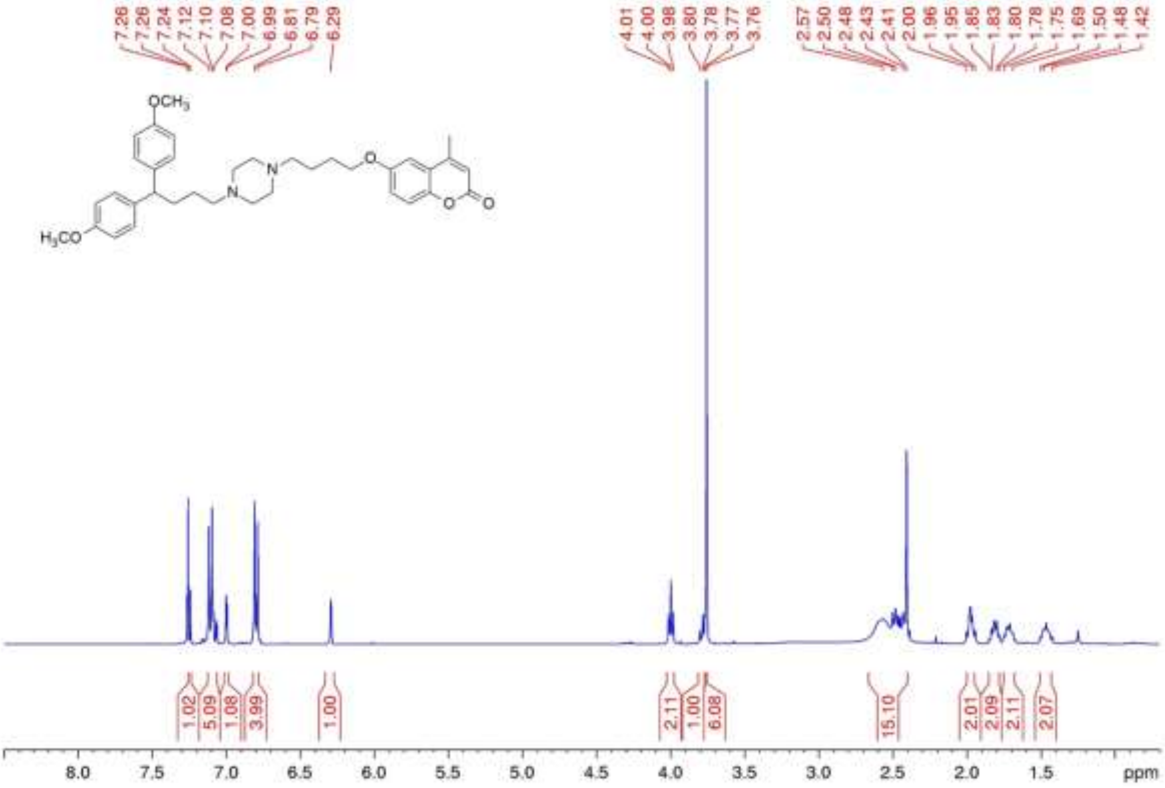


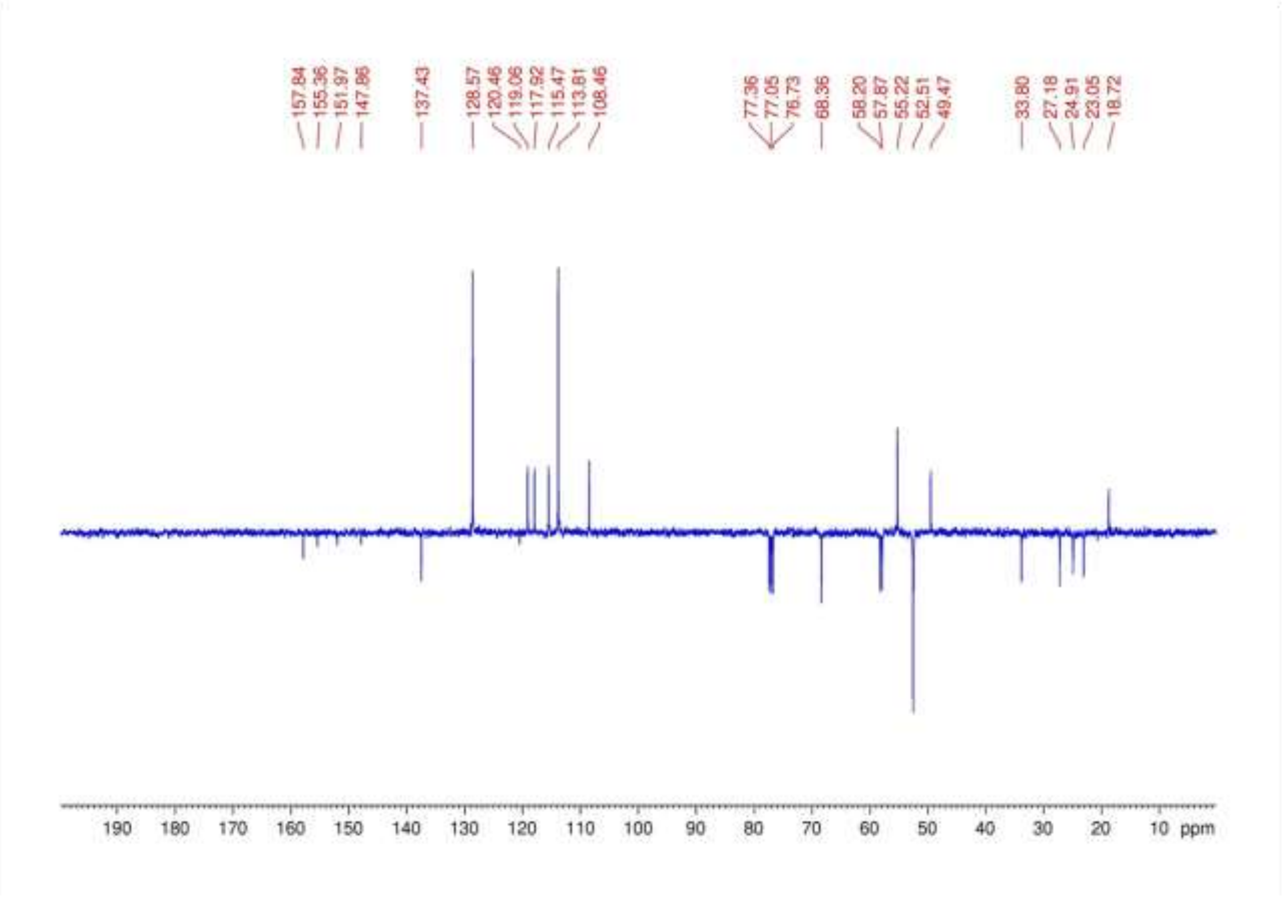

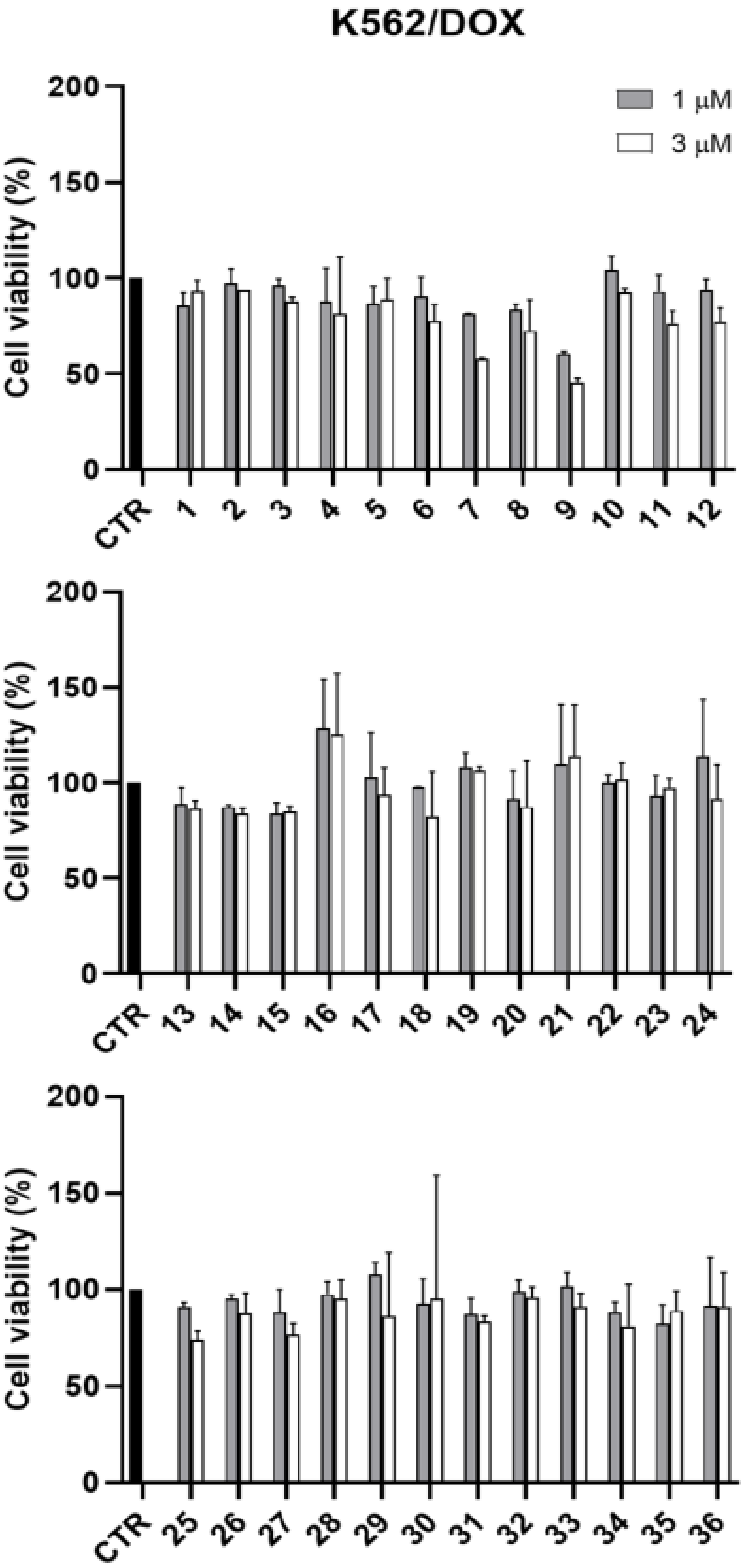


**Figure S1.** Viability of K562/DOX cells incubated for 72 h with compounds **1-36** at 1 and 3 μM, measured by the MTT assay, in triplicates. Data are the means + SD (n= 3). Control (CTR) is 100% cell growth.

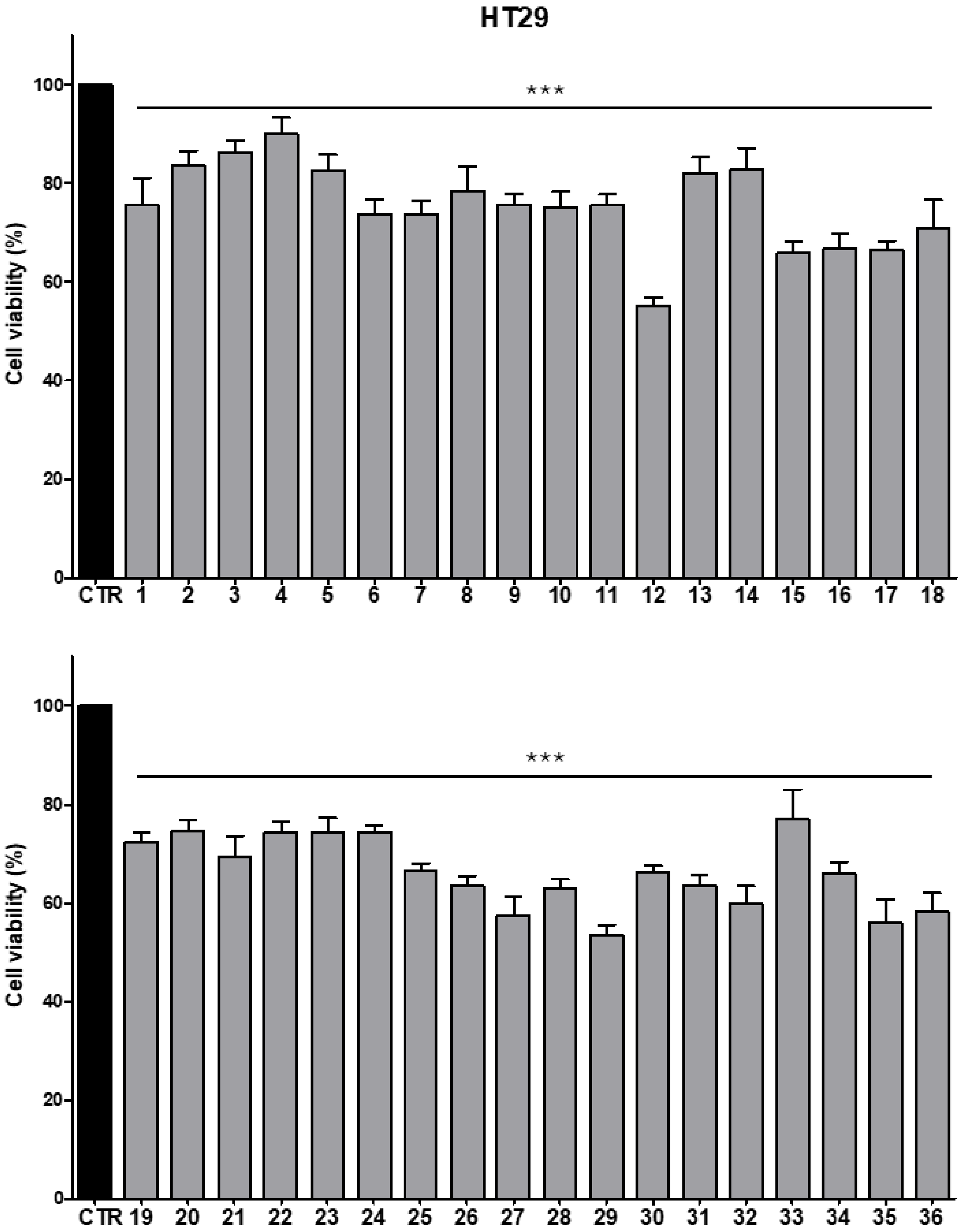


**Figure S2.** Viability of HT29 cells incubated for 48 h with compounds **1-36** at 1 μM, measured by the MTT assay, in triplicates. Data are the means ± SD (n= 3). Control (CTR) is 100% cell growth. One-way analysis of variance (ANOVA) analysis: ***p < 0.0001 *vs* control.

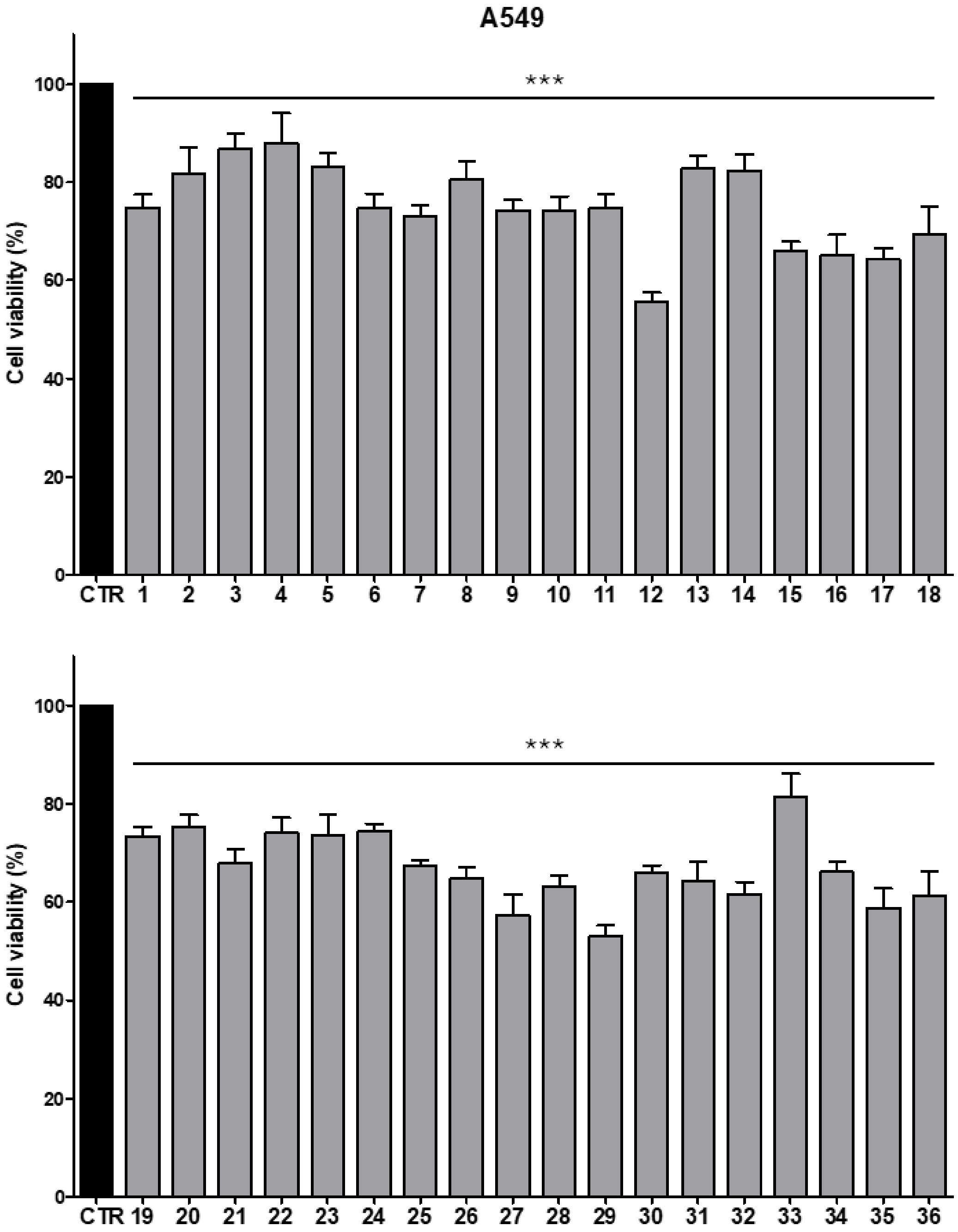


**Figure S3.** Viability of A549 cells incubated for 48 h with compounds **1-36** at 1 μM, measured by the MTT assay, in triplicates. Data are the means ± SD (n= 3). Control (CTR) is 100% cell growth. One-way analysis of variance (ANOVA) analysis: ***p < 0.0001 *vs* control.

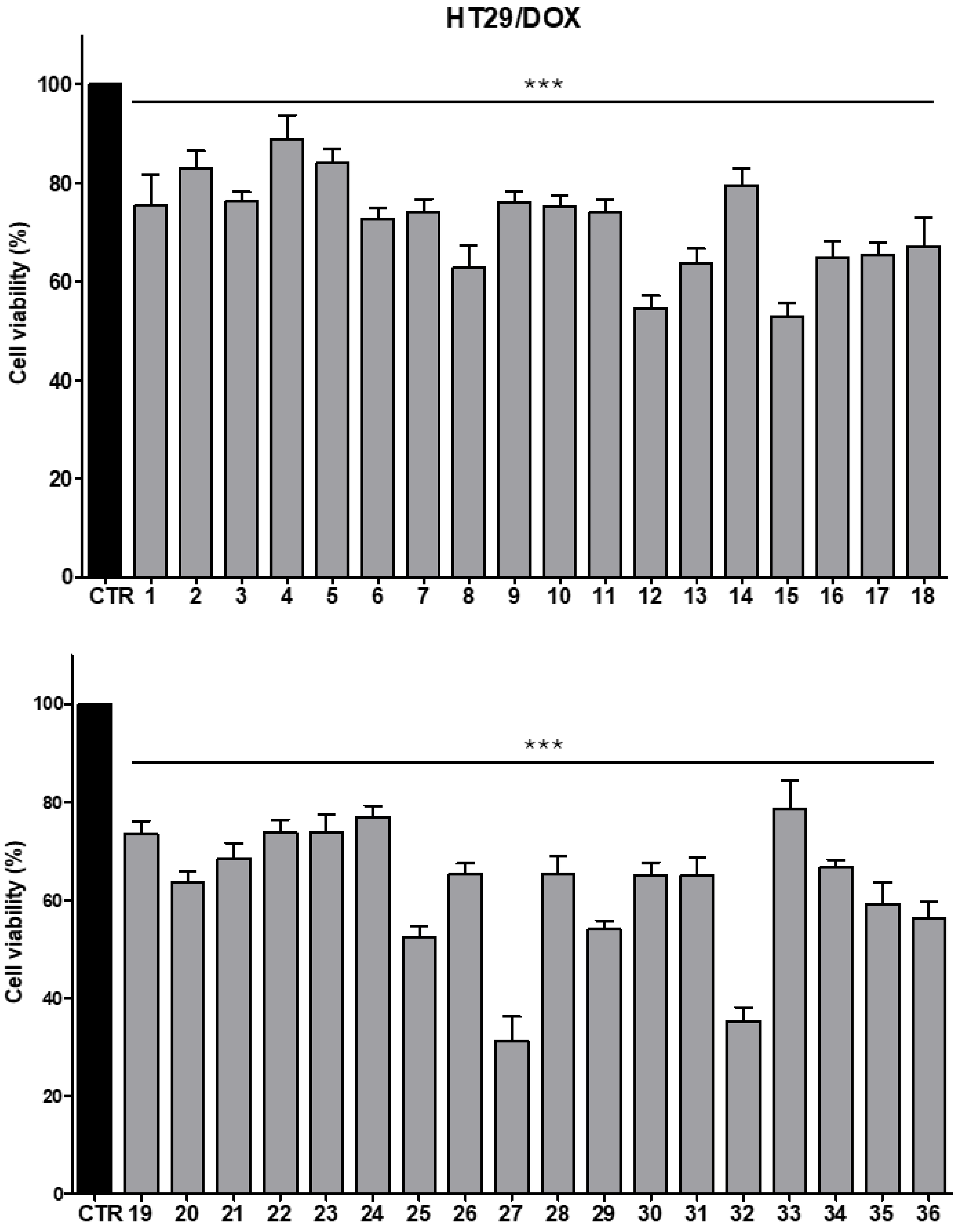


**Figure S4.** Viability of the resistant HT29/DOX cells incubated for 48 h with compounds **1-36** at 1 μM, measured by the MTT assay, in triplicates. Data are the means ± SD (n= 3). Control (CTR) is 100% cell growth. One-way analysis of variance (ANOVA) analysis: ***$p < 0.0001$ *vs* control.

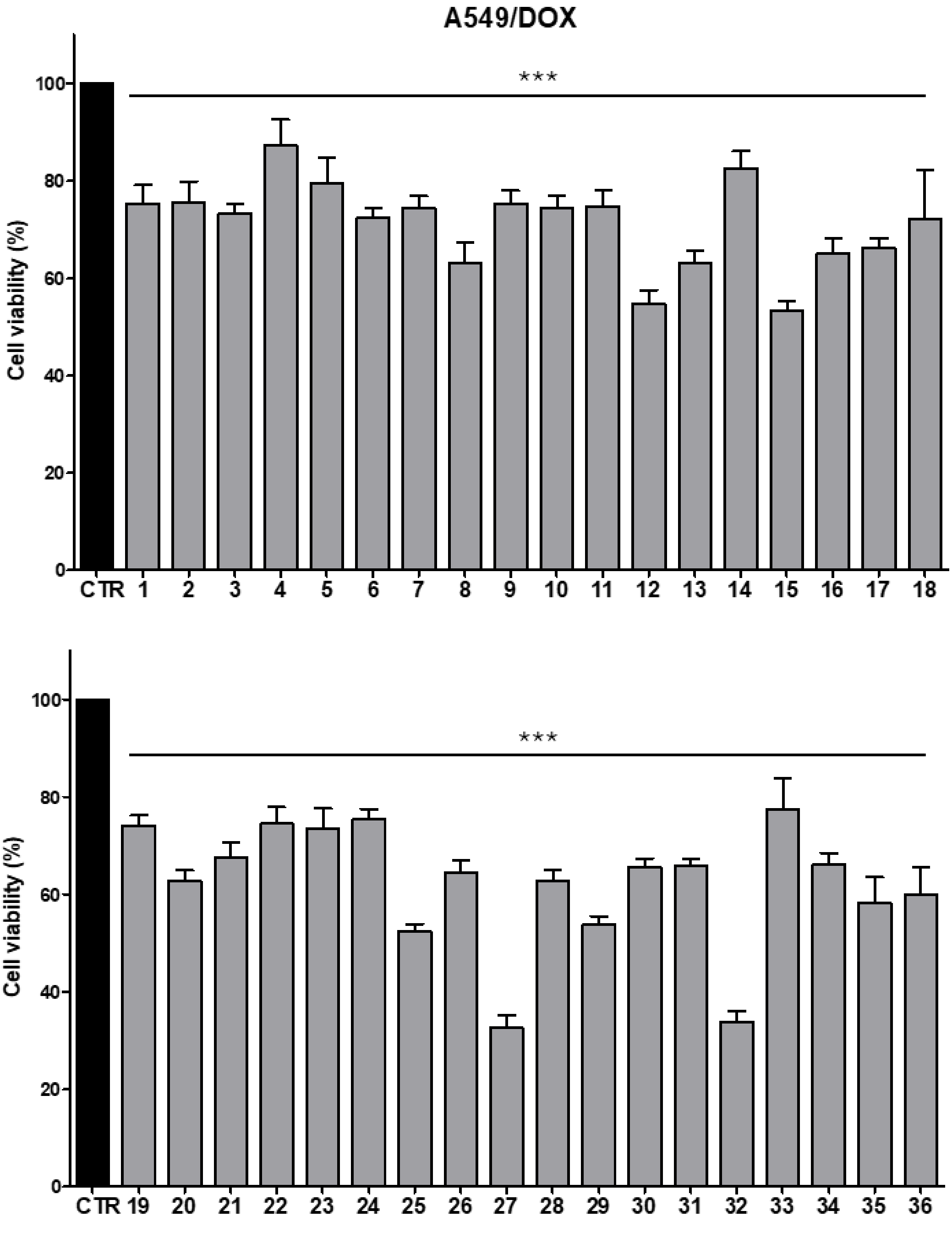


**Figure S5.** Viability of the resistant A549/DOX cells incubated for 48 h with compounds **1-36** at 1 μM, measured by the MTT assay, in triplicates. Data are the means ± SD (n= 3). Control (CTR) is 100% cell growth. One-way analysis of variance (ANOVA) analysis: ***p < 0.0001 *vs* control.

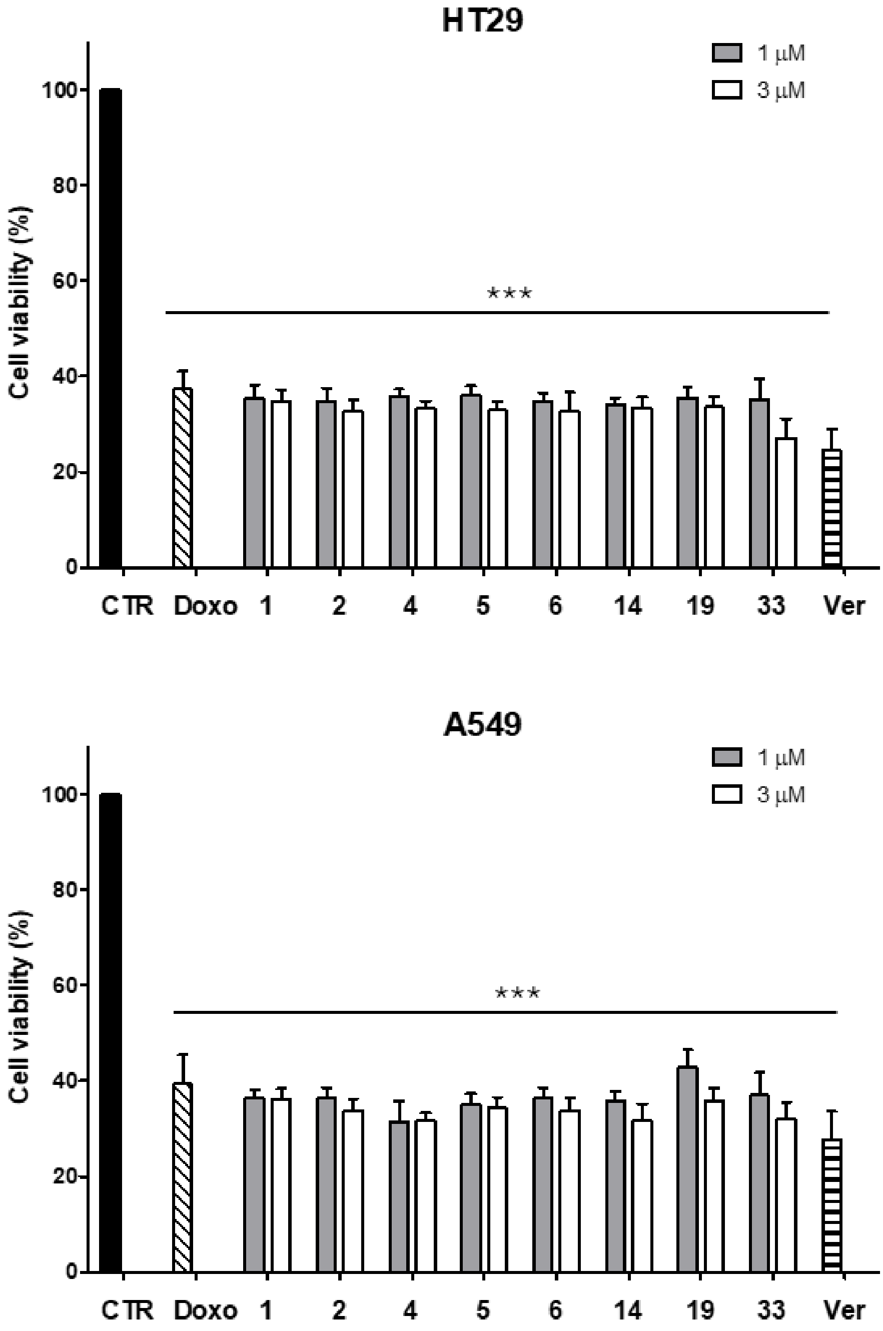


**Figure S6.** Antiproliferative activity in HT29 (top) and A549 (bottom) cells of doxorubicin (Doxo) at 5 μM, alone and in co-administration with selected derivatives (**1**, **2**, **4-6**, **14**, **19**, **33**) at 1 μM and 3 μM, or verapamil (Ver) at 1 mM, measured after 48 h. Each bar represents the mean ± SD of three independent experiments, with technical triplicates. Control (CTR) is 100% cell growth. One-way analysis of variance (ANOVA) analysis: ***$p < 0.0001$ *vs* control.

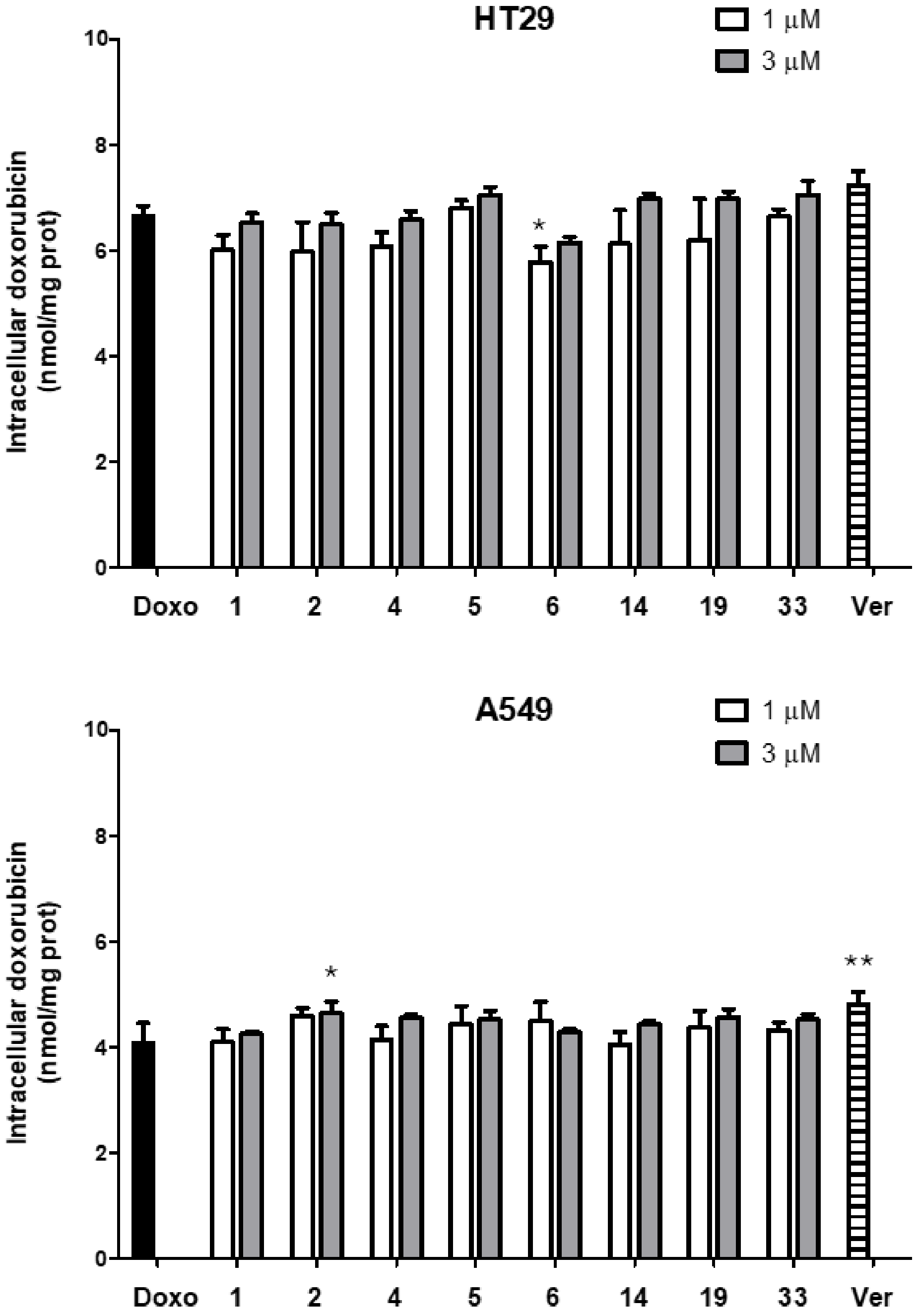


**Figure S7.** Intracellular accumulation of doxorubicin in HT29 (top) and A549 (bottom) cells, incubated 24 h with doxorubicin (Doxo) at 5 µM alone and in co-administration with selected derivatives (**1**, **2**, **4-6**, **14**, **19**, **33**) at 1 µM and 3 µM, or verapamil (Ver) at 1 mM. Each bar represents the mean ± SD of three independent experiments, with technical duplicates. One-way analysis of variance (ANOVA) analysis: *p < 0.05, **p < 0.001 *vs* doxorubicin alone.

**HPLC-DAD method for purity analysis**

The employed chromatographic parameters to check the purity of representative compounds were reported as follows:

- column, Pursuit C18 length = 100 mm, internal diameter = 2 mm; particle size = 3 μm purchased from Agilent Technologies (Palo Alto, CA, USA)
- acidic mobile phase, composed by 5 mM of ammonium formate and 10 mM of formic acid in mQ water: acetonitrile 90:10 (v/v) solution (solvent A), 10 mM of ammonium formate and 5 mM of formic acid in mQ water: acetonitrile 10:90 (v/v) solution (solvent B).
- flow rate and the injection volume were 0.35 mL $min^{-1}$ and 5 μL respectively.
- DAD detection set up was in the UV range between 210 to 400 nm. The chromatographic profile of each analyte was monitored at the λ of highest absorbance of its characteristic chromophore.

The elution gradient is shown in Table S1.

**Table S1**: Elution gradient of mobile phase used for HPLC-DAD analysis.

| Time (min) | A (%) |
|---|---|
| 0.00 | 90 |
| 8.00 | 10 |
| 13.00 | 10 |
| 13.01 | 90 |
| 18.00 | 90 |

The sample solution of each analyte/compound was prepared at 100 μg $mL^{-1}$ in mQ water: acetonitrile 50:50 and analyzed by the HPLC-DAD method described above.

In order to evaluate the purity of the studied compounds (≥ 95 %) a proper integration area threshold was set.

Chromatographic profiles of HPLC-DAD analysis of final compounds **1-36** were reported in Figures S8-S43.

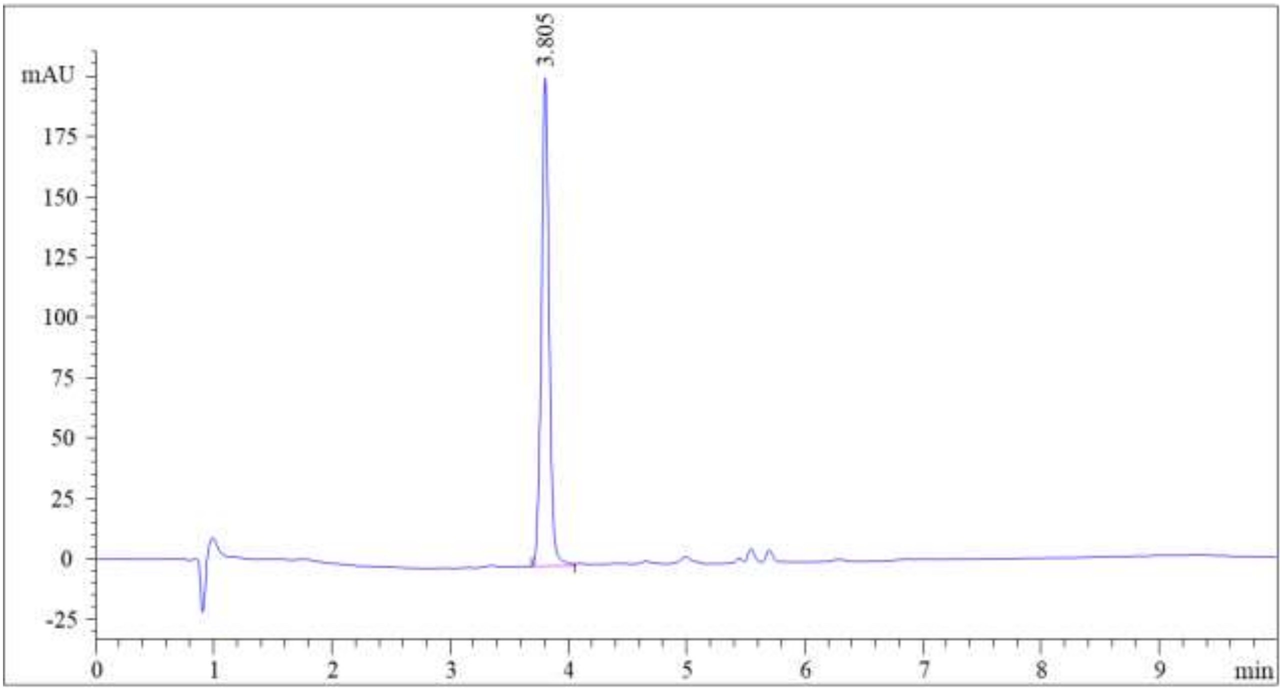


**Figure S8:** Chromatographic profile of compound **1** monitored at λ=270 nm.

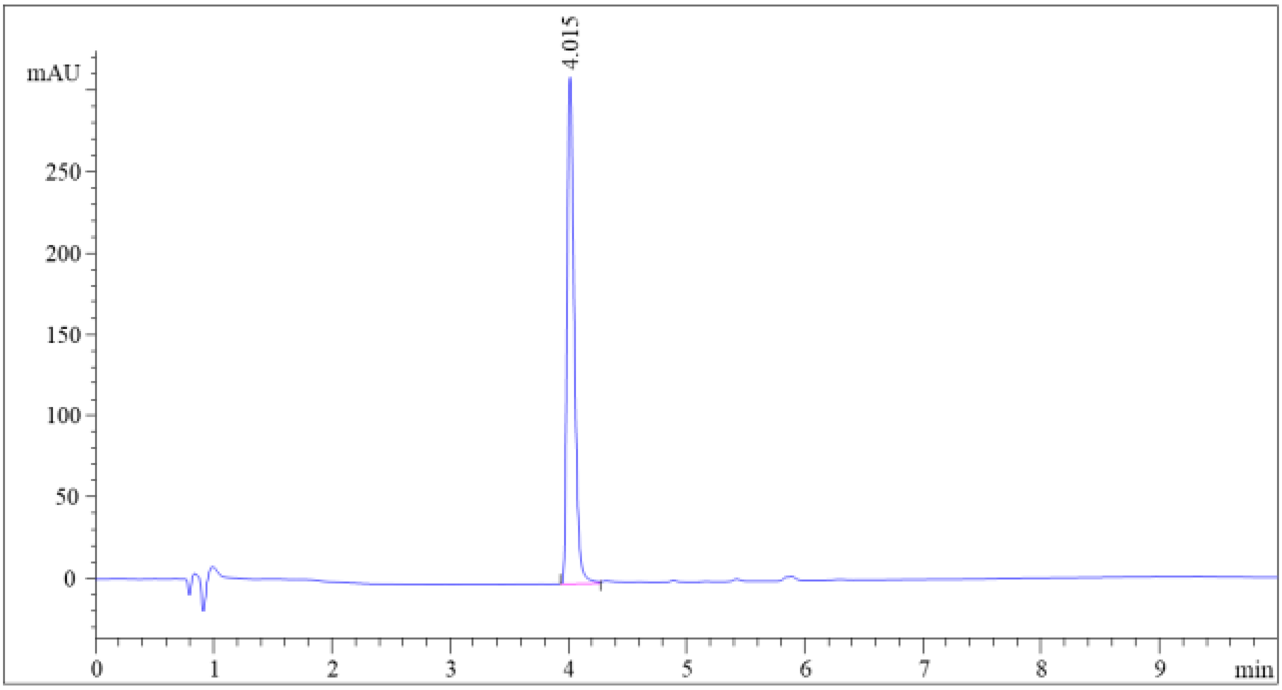


**Figure S9:** Chromatographic profile of compound **2** monitored at λ=270 nm.

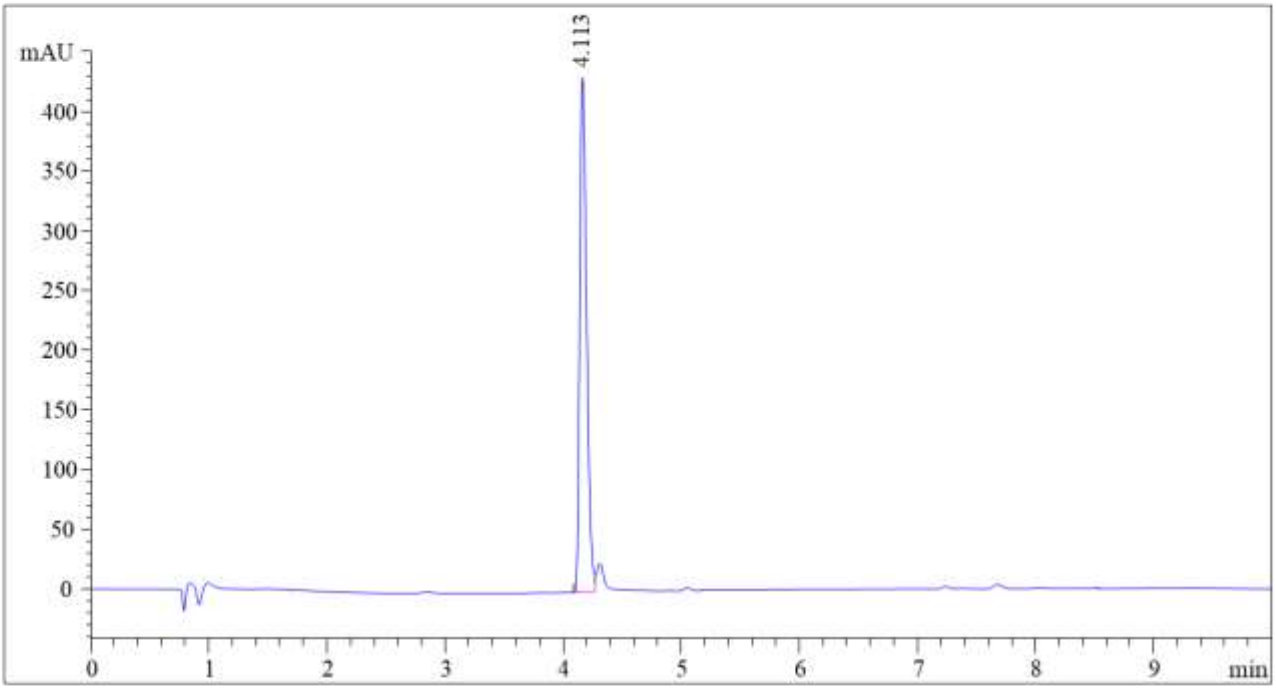


**Figure S10:** Chromatographic profile of compound **3** monitored at λ=270 nm.

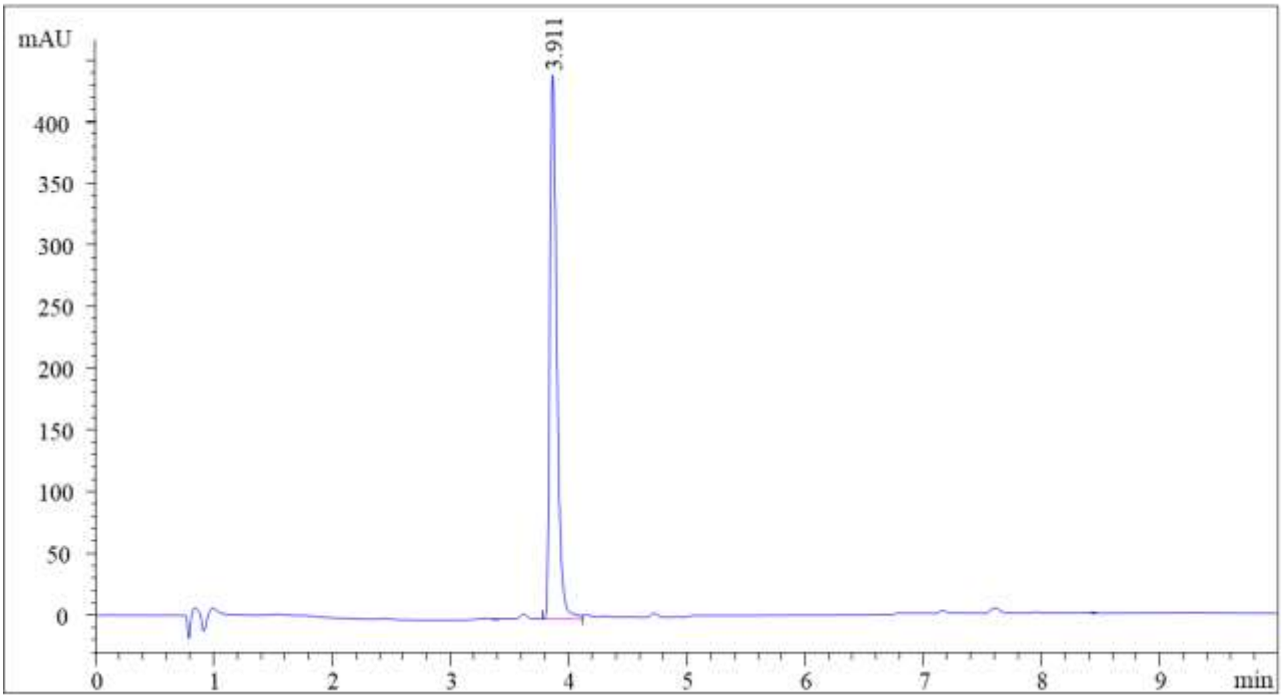


**Figure S11:** Chromatographic profile of compound **4** monitored at λ=270 nm.

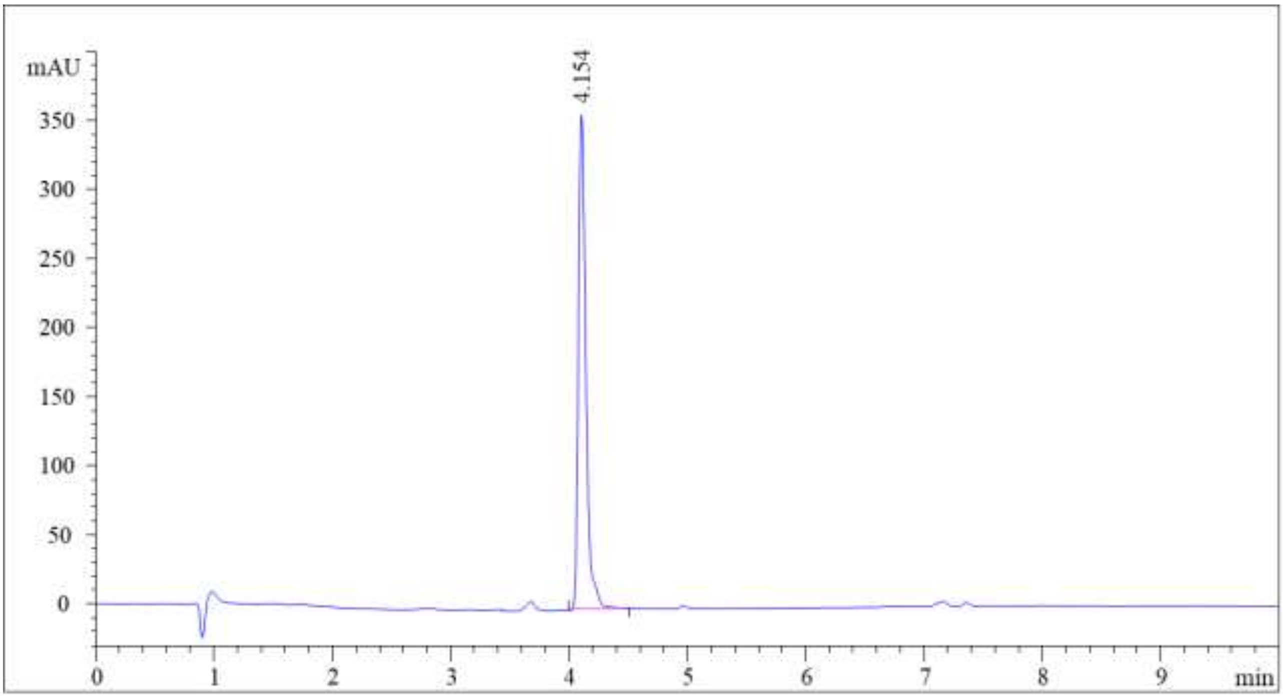


**Figure S12:** Chromatographic profile of compound **5** monitored at λ=270 nm.

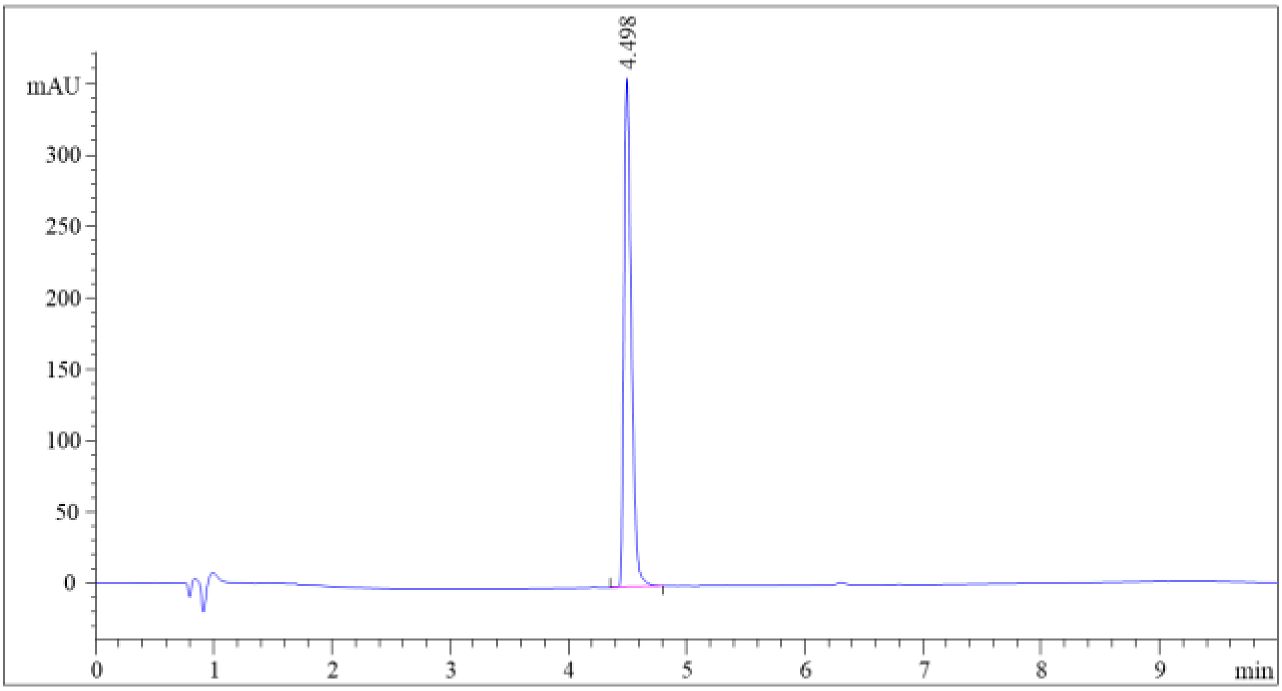


**Figure S13:** Chromatographic profile of compound **6** monitored at λ=270 nm.

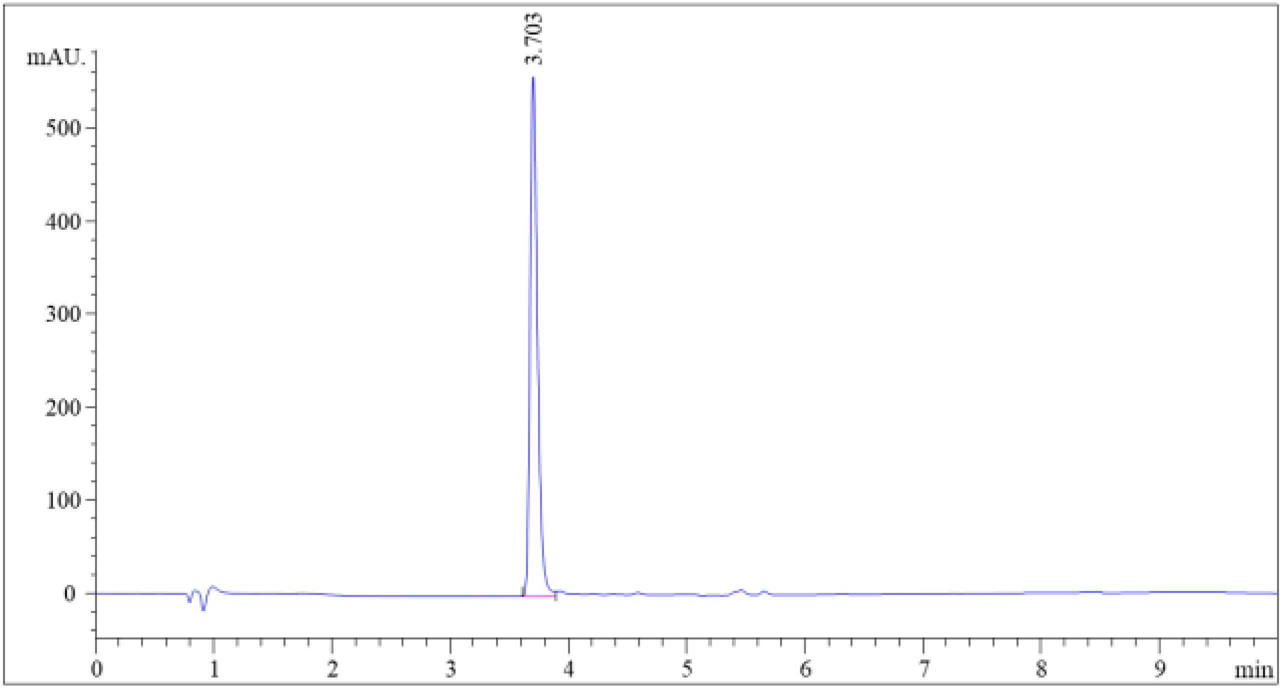


**Figure S14:** Chromatographic profile of compound **7** monitored at λ=270 nm.

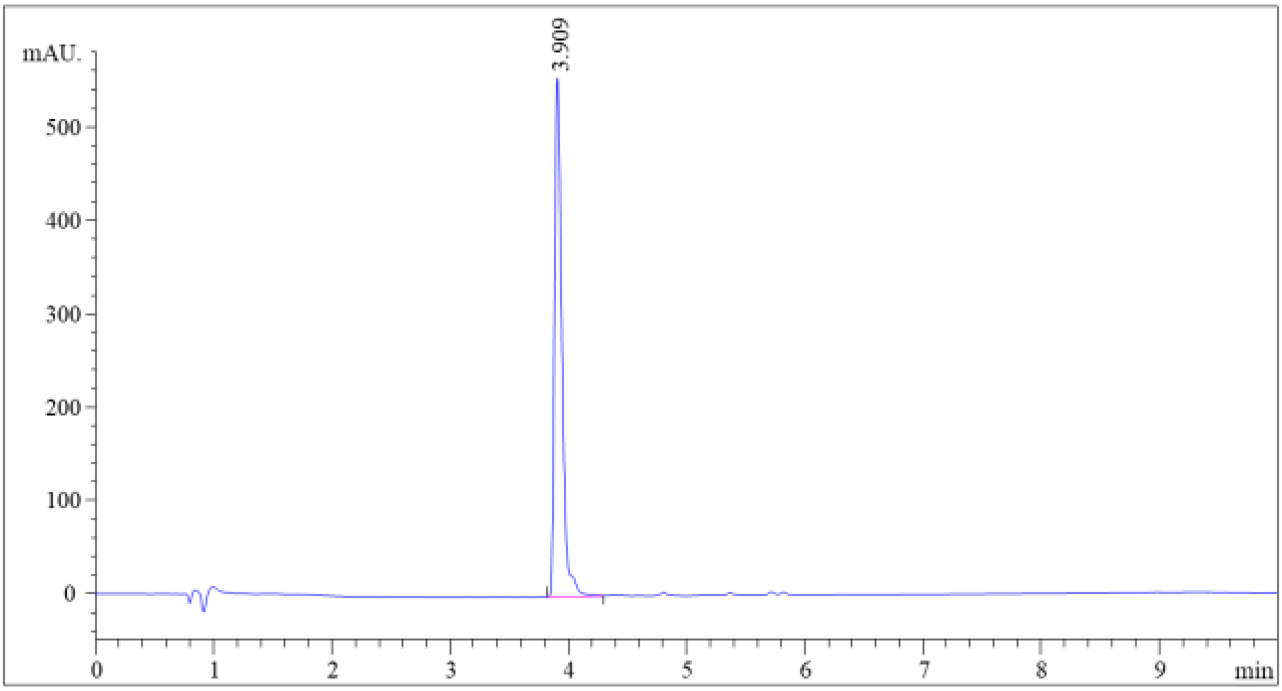


**Figure S15:** Chromatographic profile of compound **8** monitored at λ=270 nm.

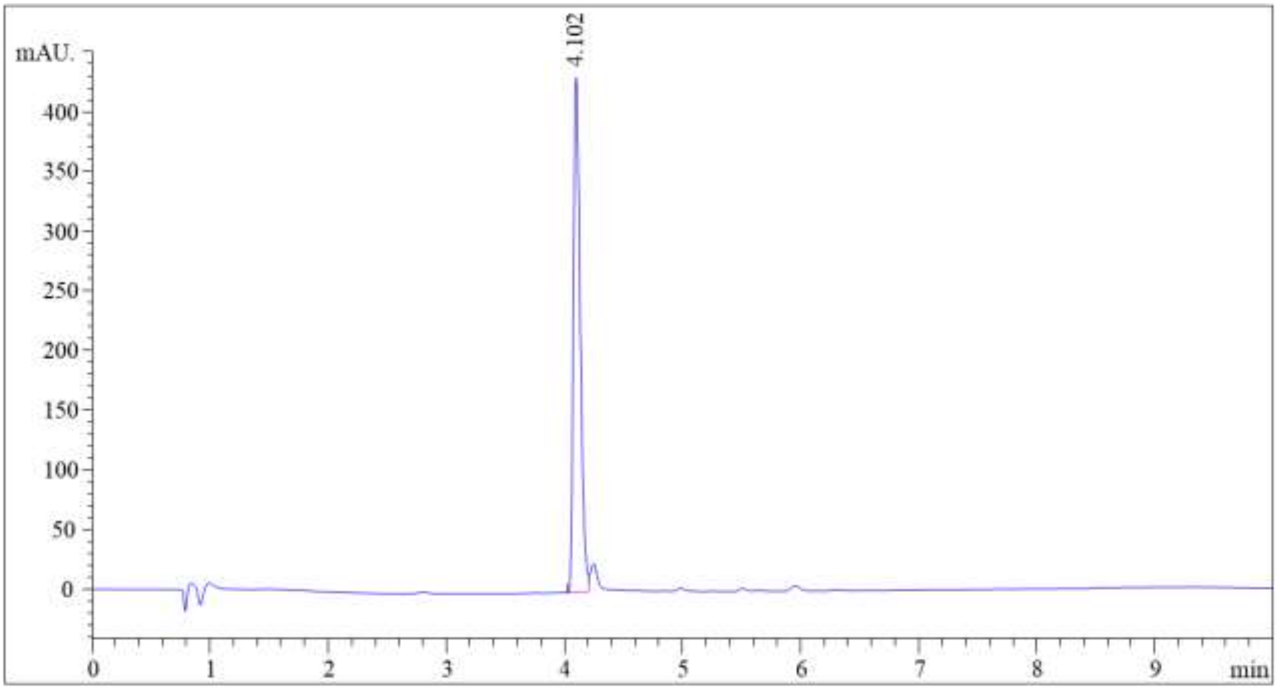


**Figure S16:** Chromatographic profile of compound **9** monitored at λ=270 nm.

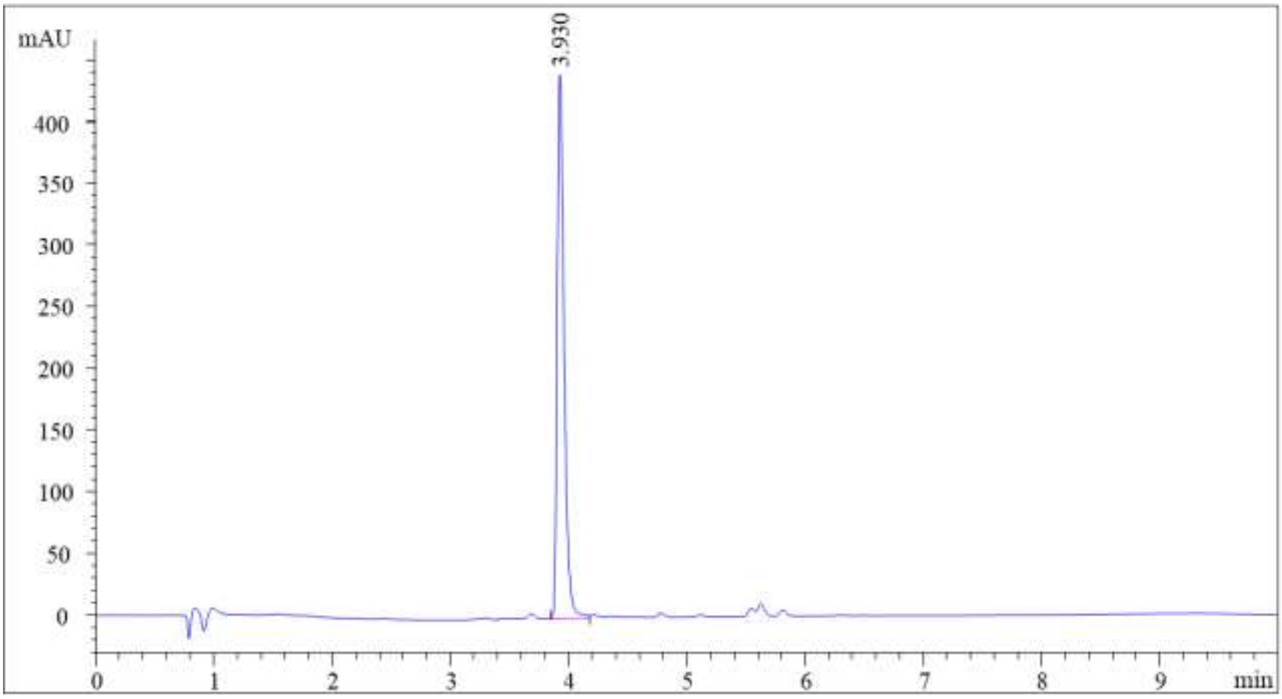


**Figure S17:** Chromatographic profile of compound **10** monitored at λ=270 nm.

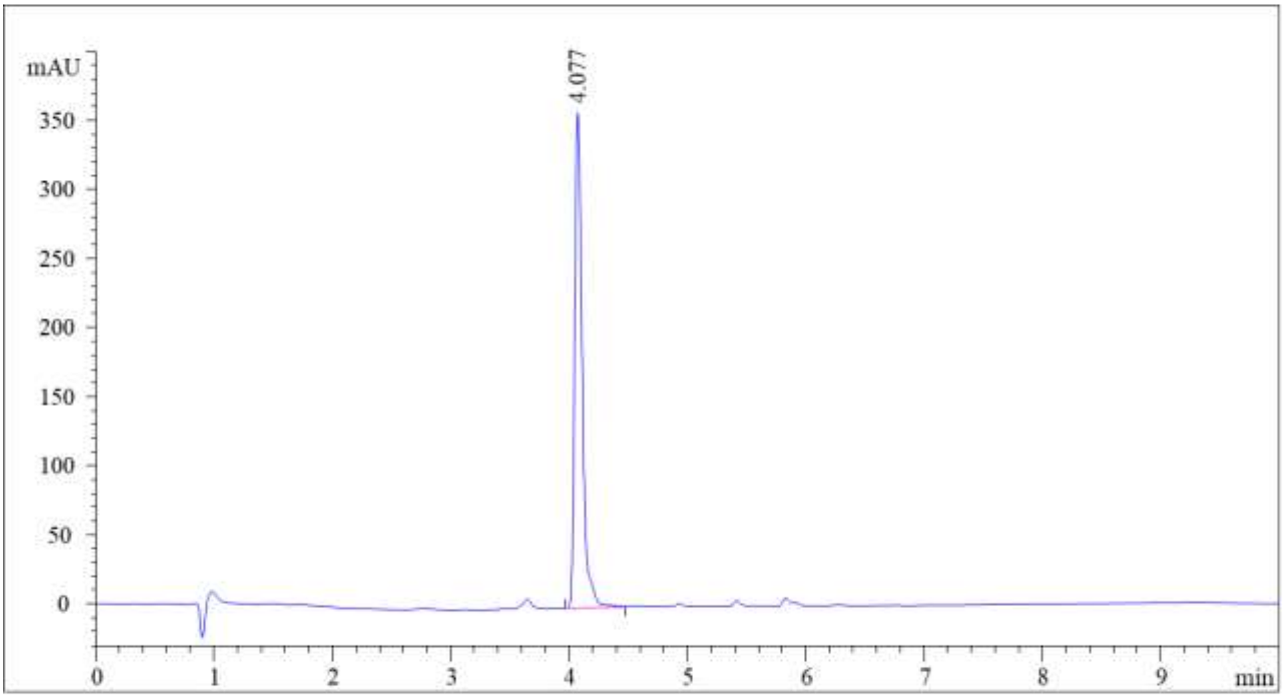


**Figure S18:** Chromatographic profile of compound **11** monitored at λ=270 nm.

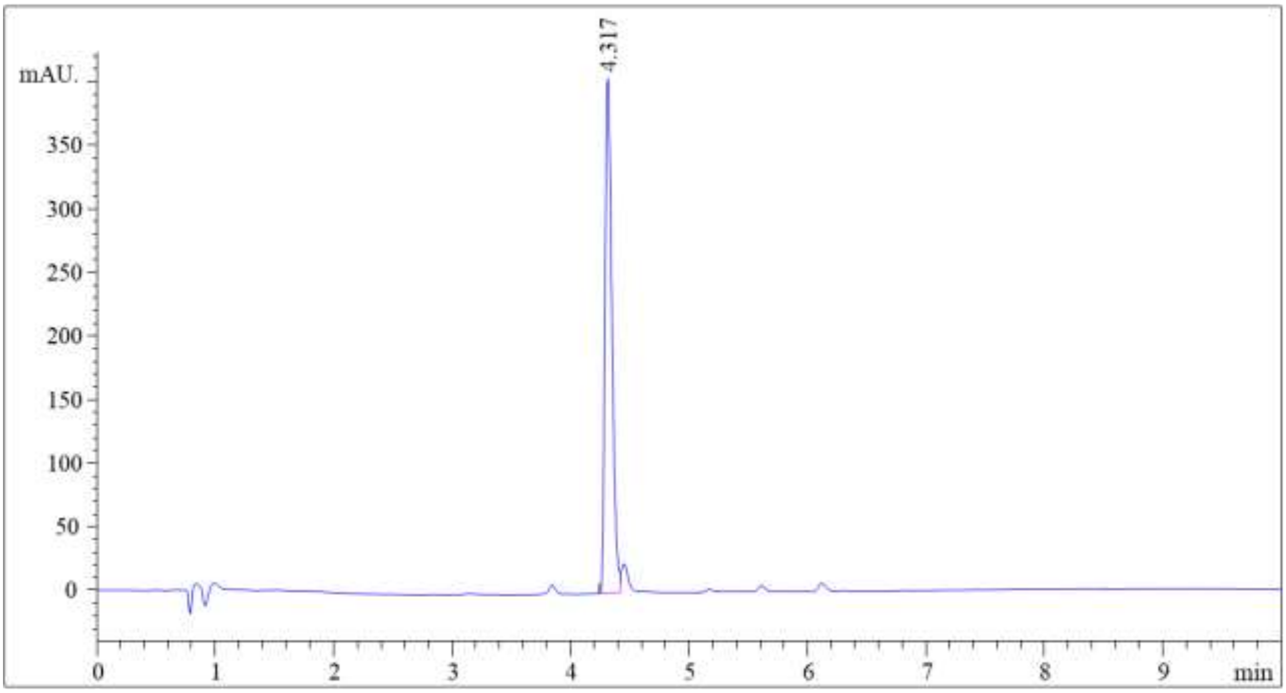


**Figure S19:** Chromatographic profile of compound **12** monitored at λ=270 nm.

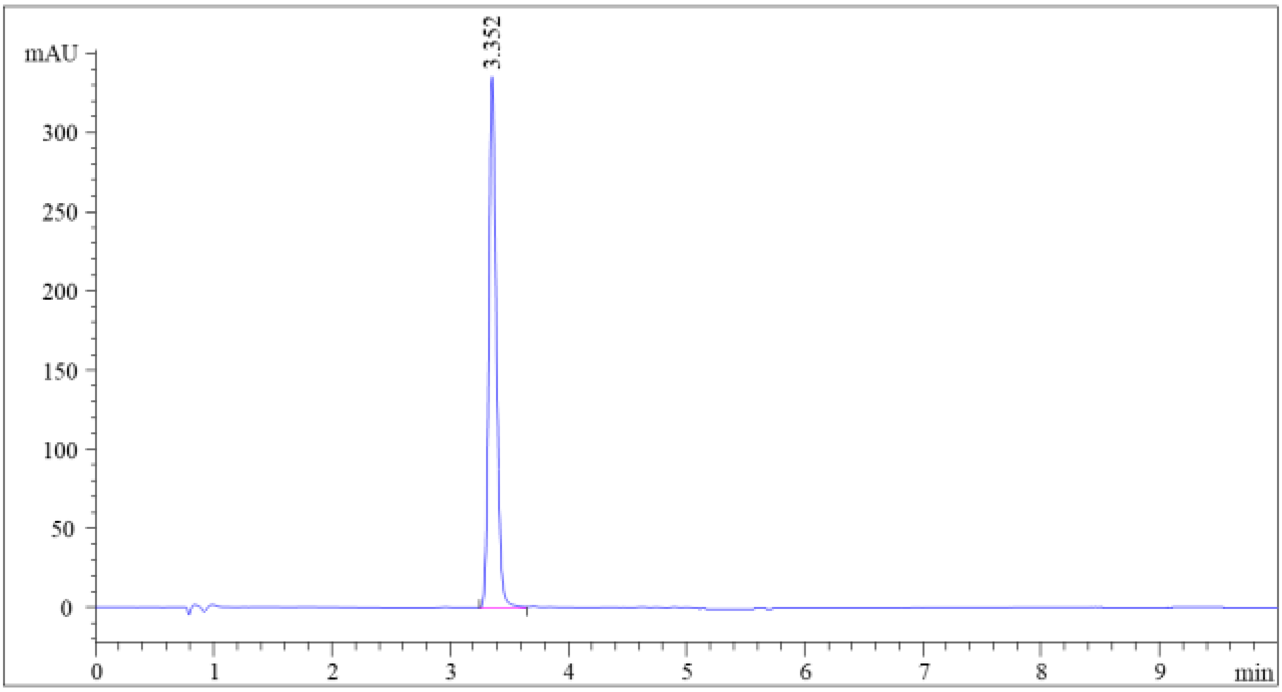


**Figure S20:** Chromatographic profile of compound **13** monitored at λ=320 nm.

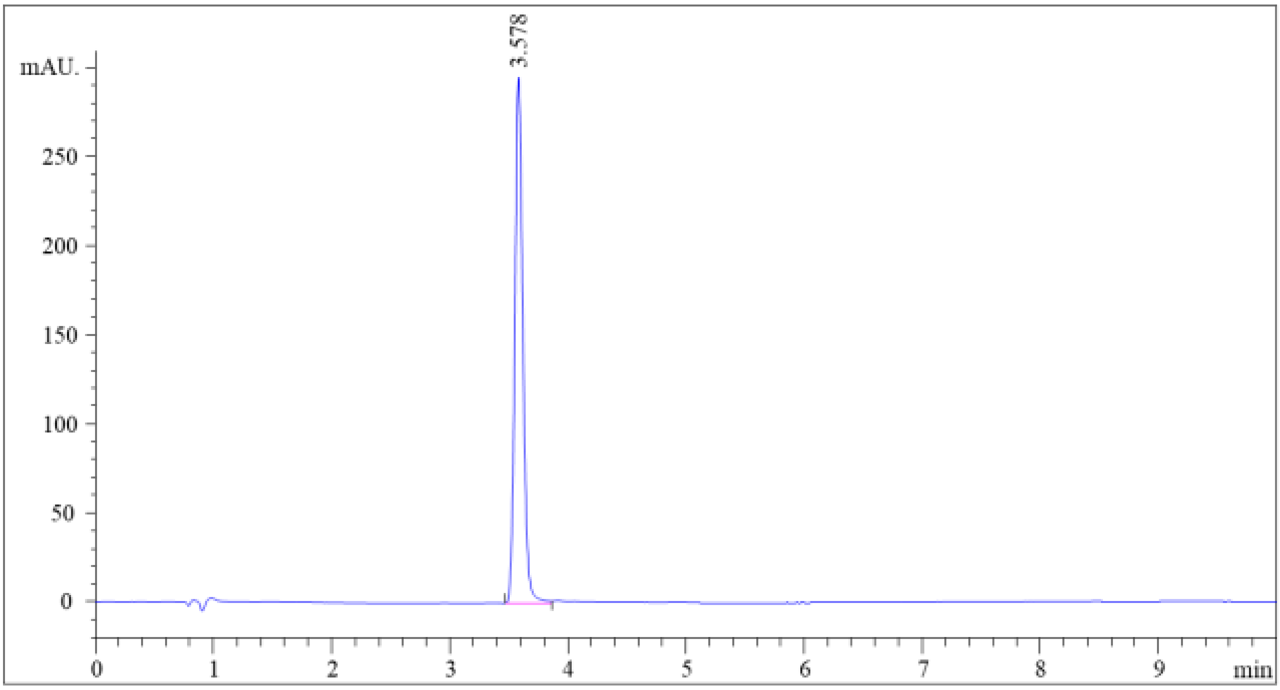


**Figure S21:** Chromatographic profile of compound **14** monitored at λ=320 nm.

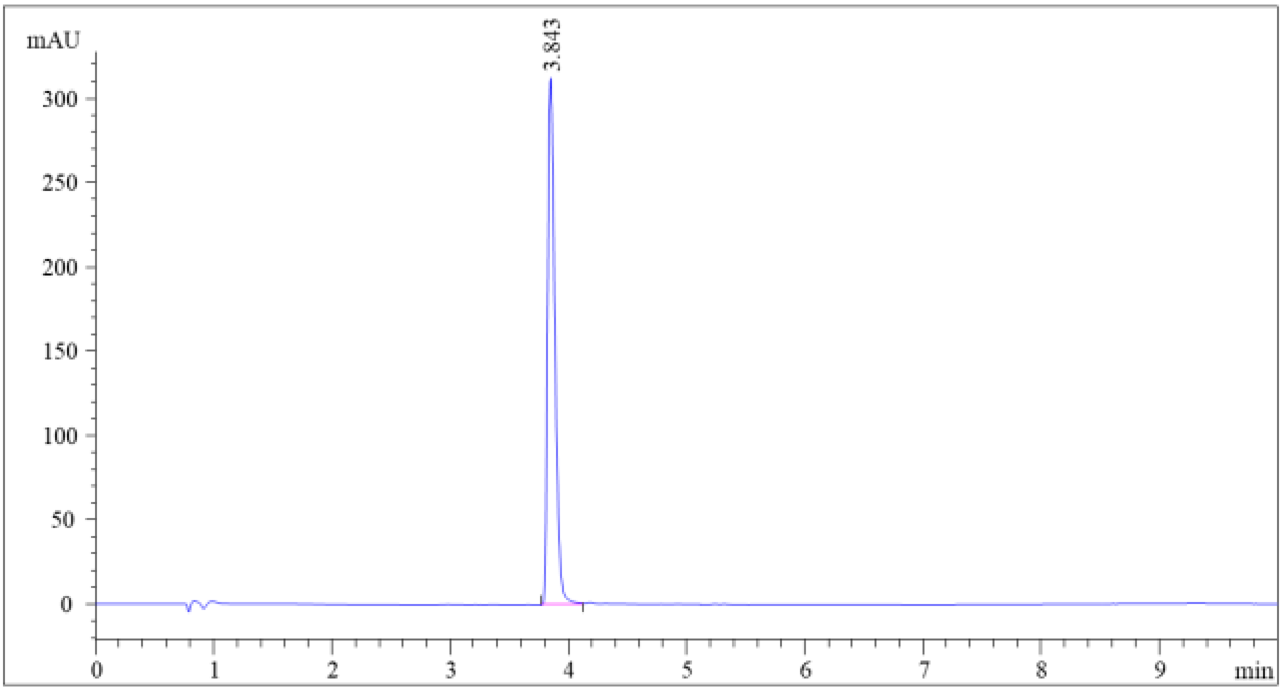


**Figure S22:** Chromatographic profile of compound **15** monitored at λ=320 nm.

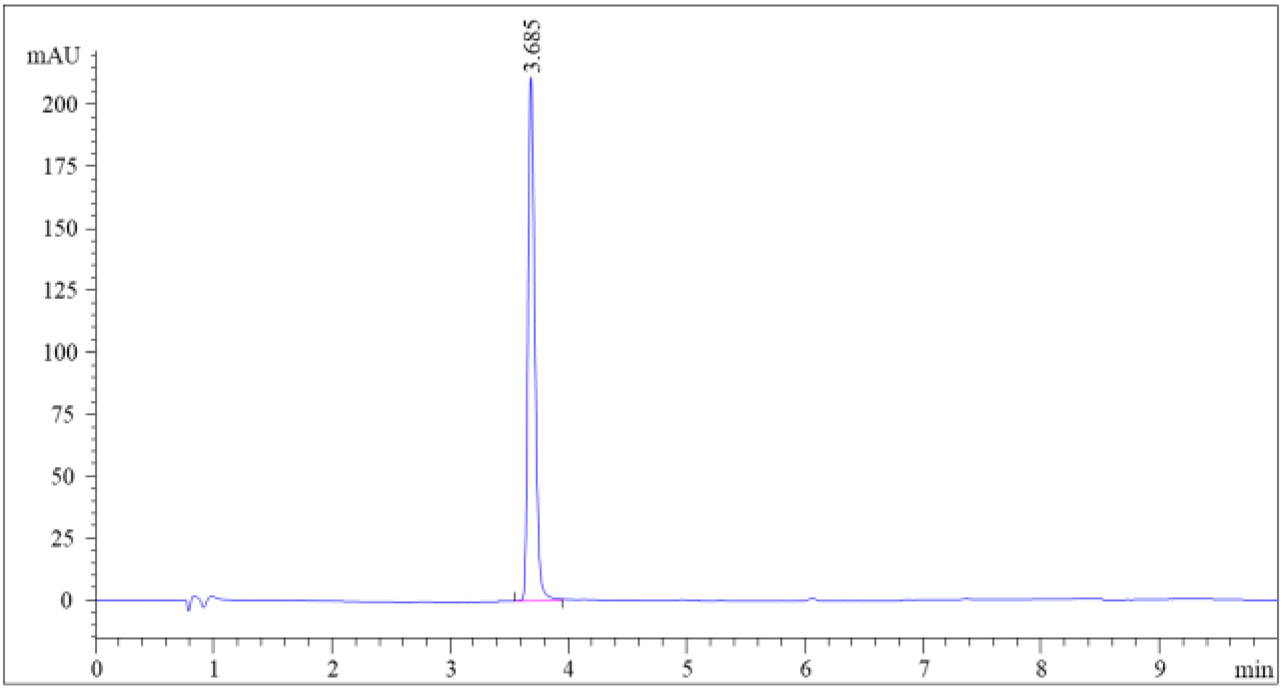


**Figure S23:** Chromatographic profile of compound **16** monitored at λ=320 nm.

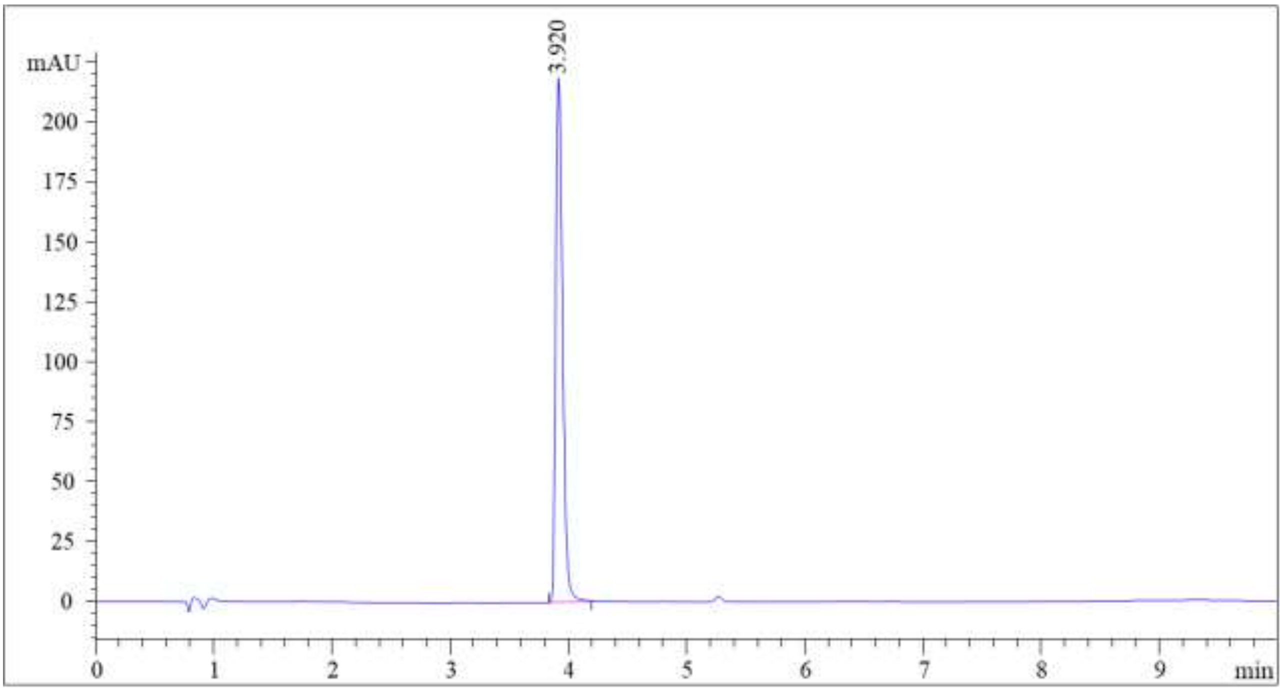


**Figure S24:** Chromatographic profile of compound **17** monitored at λ=320 nm.

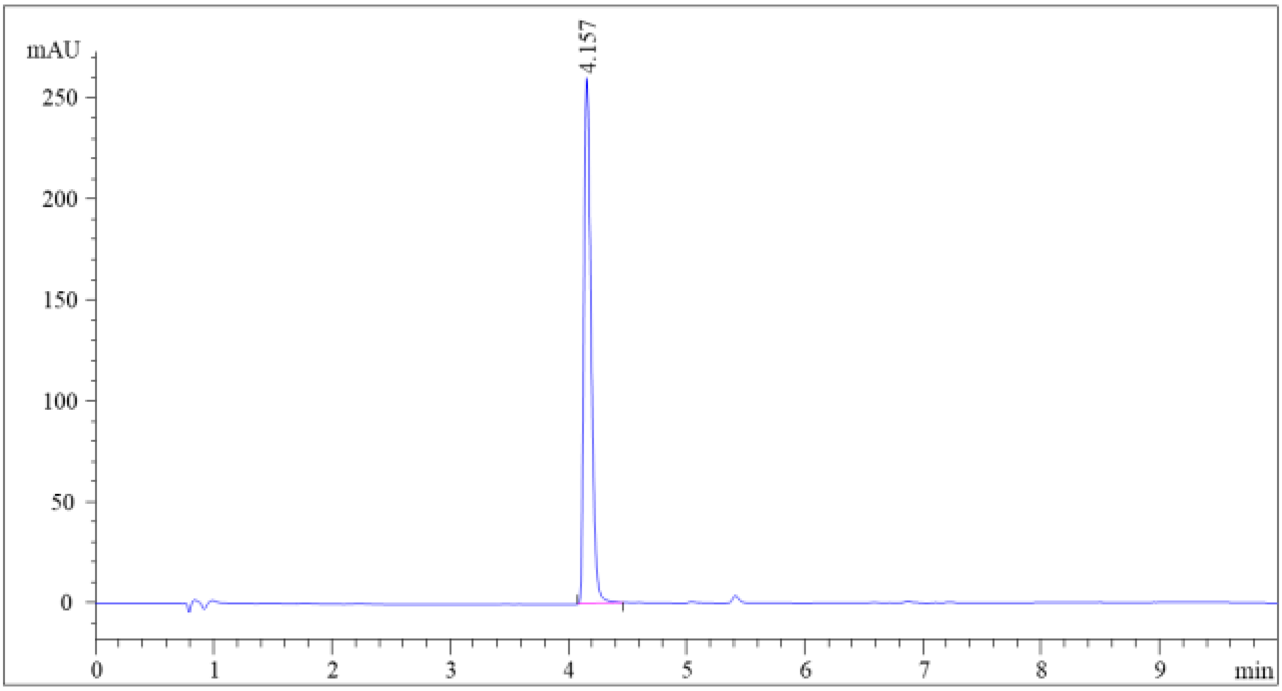


**Figure S25:** Chromatographic profile of compound **18** monitored at λ=320 nm.

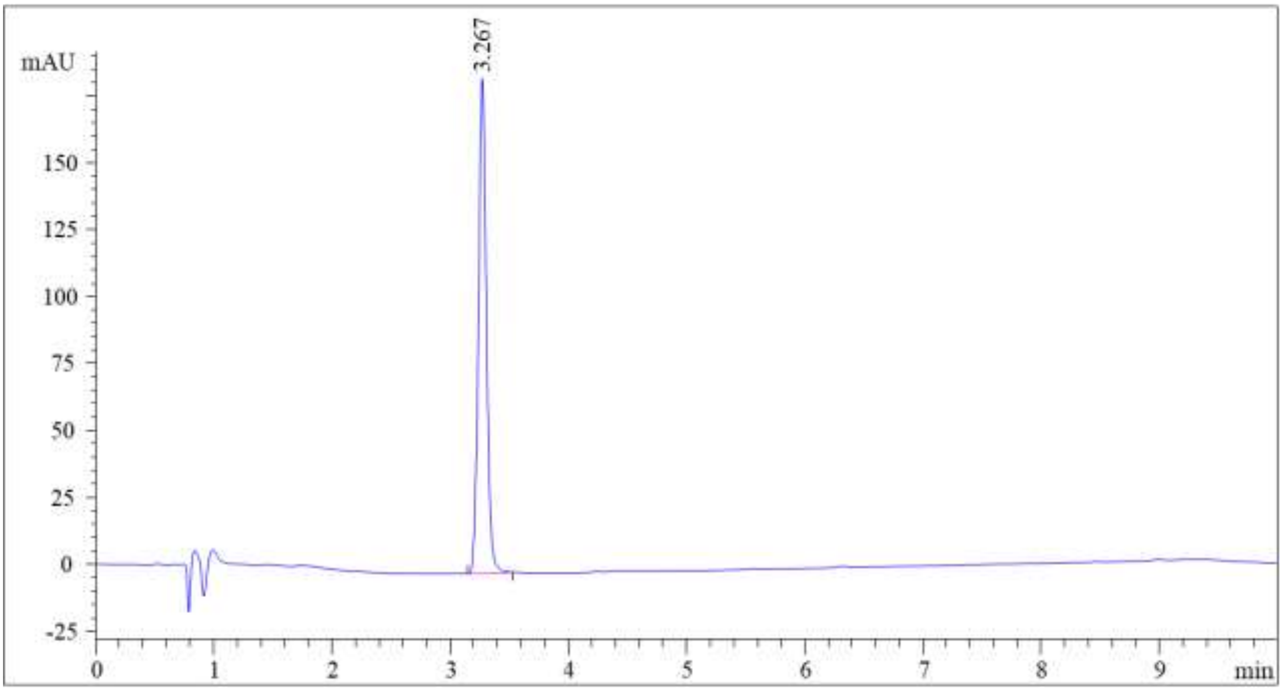


**Figure S26:** Chromatographic profile of compound **19** monitored at λ=270 nm.

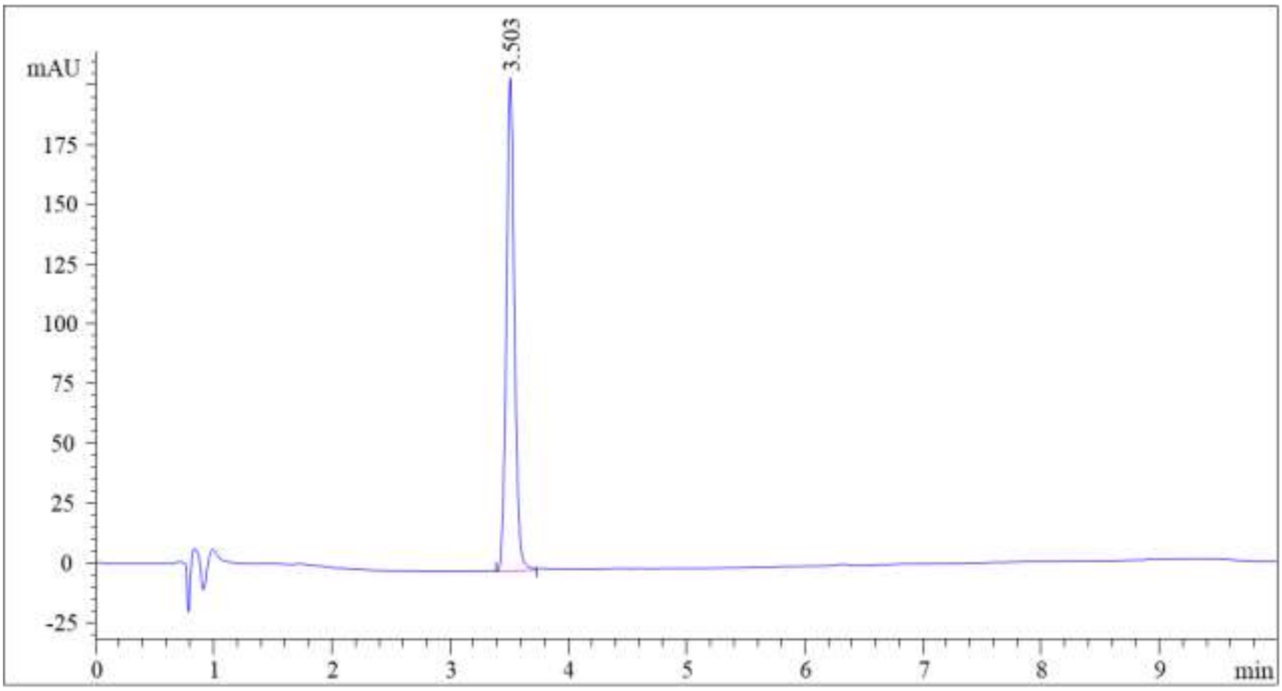


**Figure S27:** Chromatographic profile of compound **20** monitored at λ=270 nm.

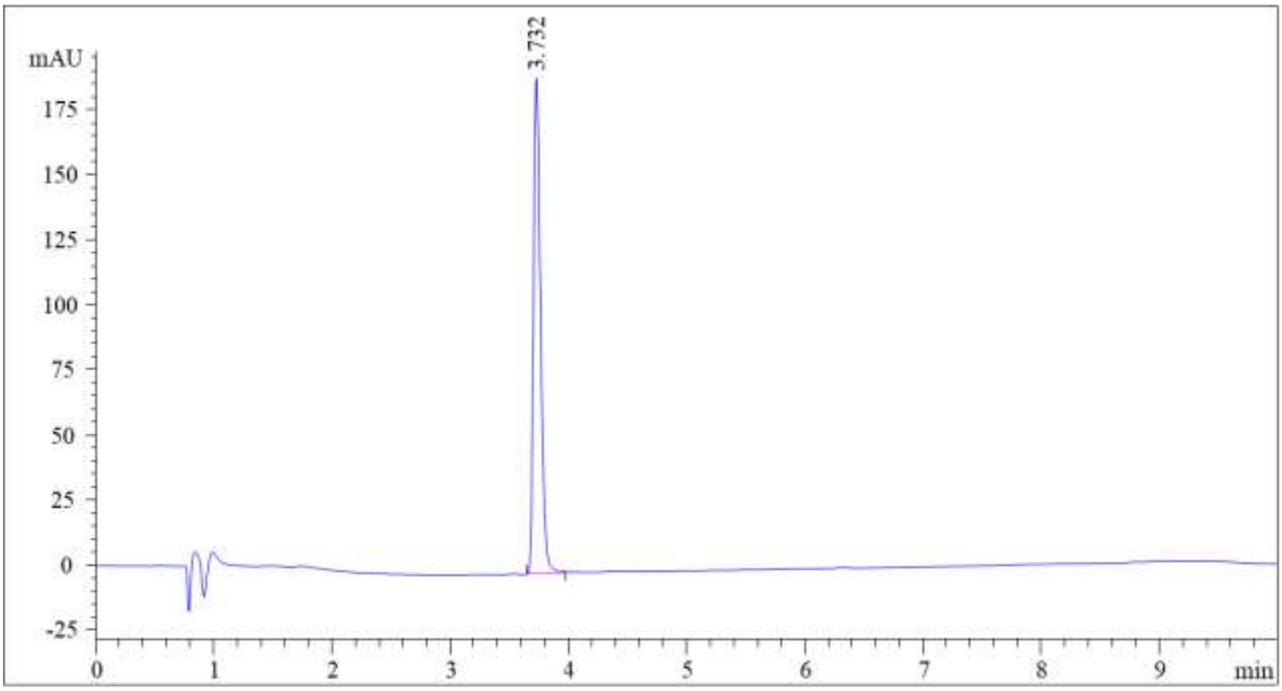


**Figure S28:** Chromatographic profile of compound **21** monitored at λ=270 nm.

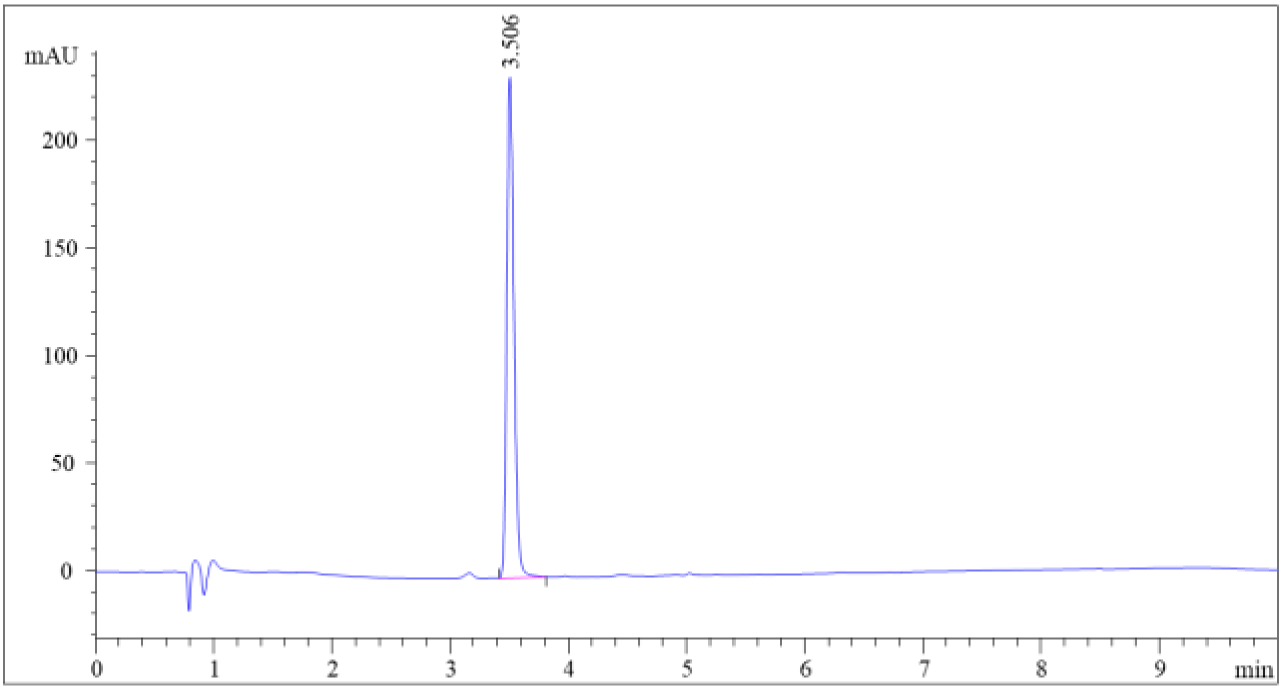


**Figure S29:** Chromatographic profile of compound **22** monitored at λ=270 nm.

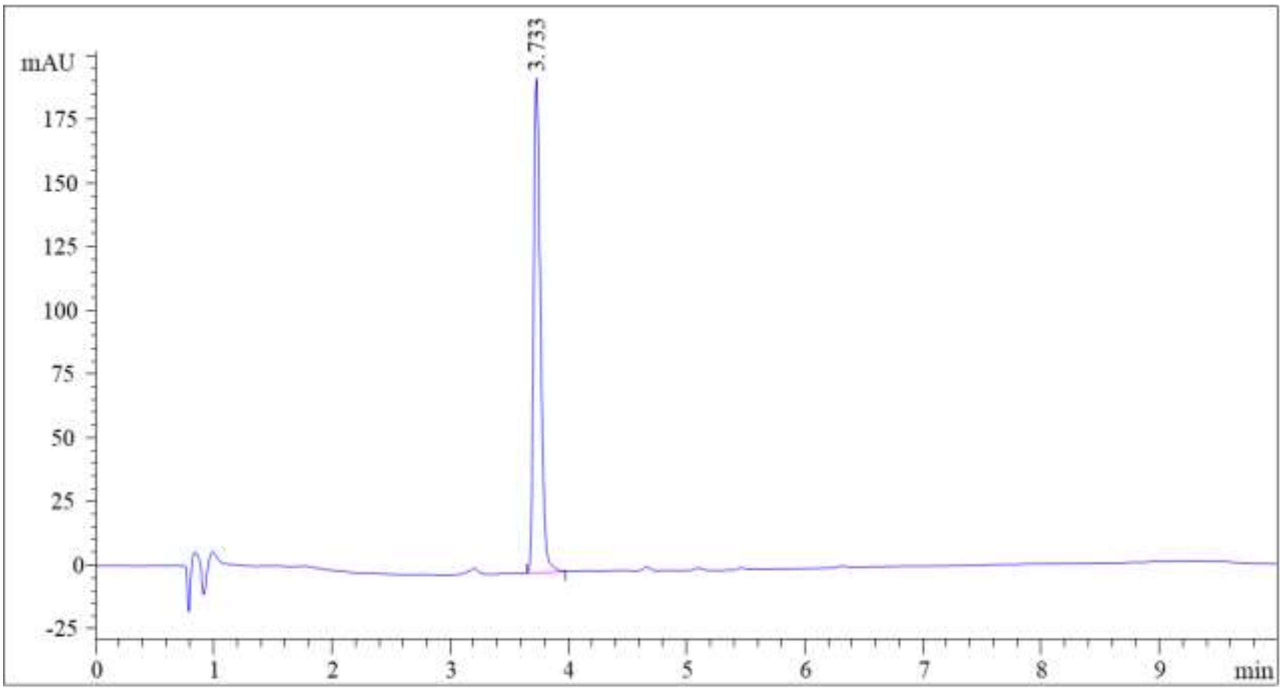


**Figure S30:** Chromatographic profile of compound **23** monitored at λ=270 nm.

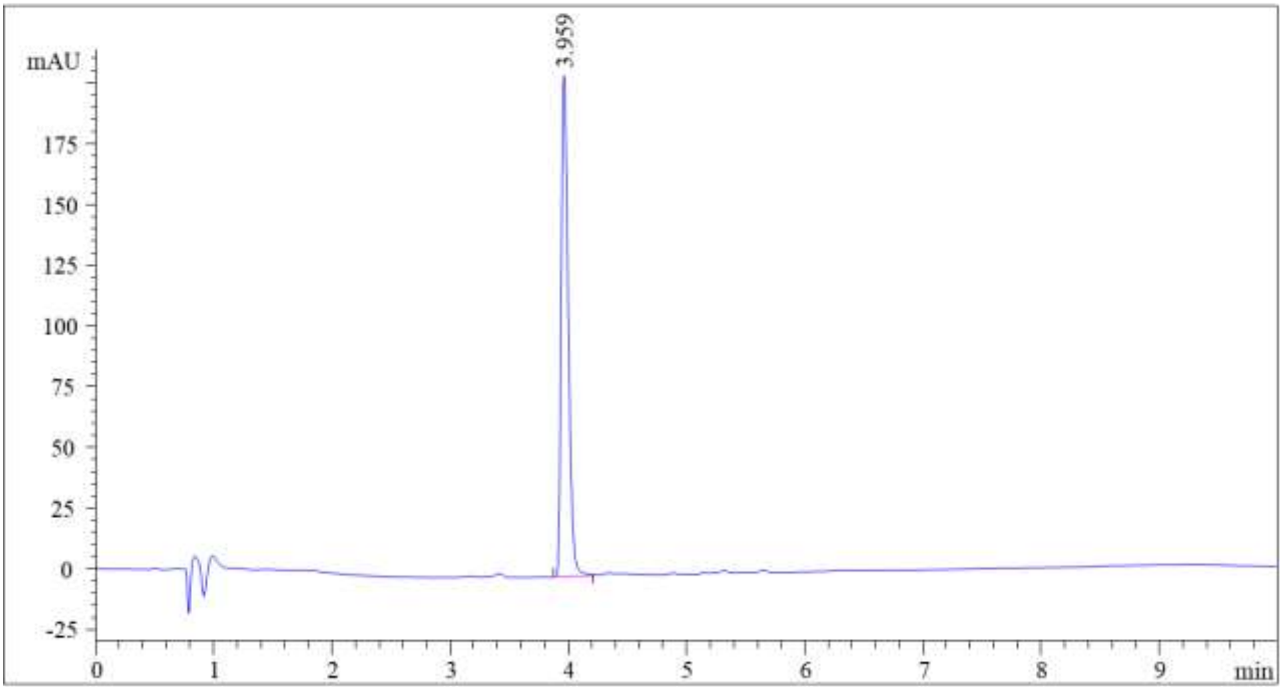


**Figure S31:** Chromatographic profile of compound **24** monitored at λ=270 nm.

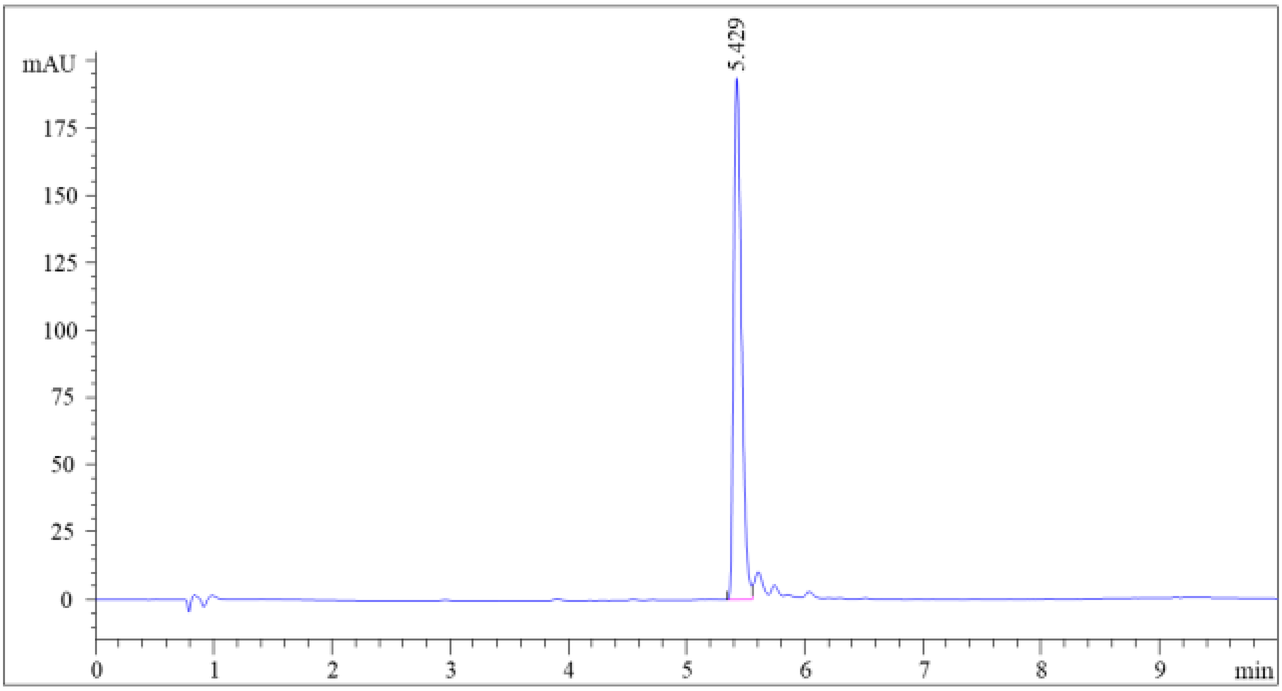


**Figure S32:** Chromatographic profile of compound **25** monitored at λ=320 nm.

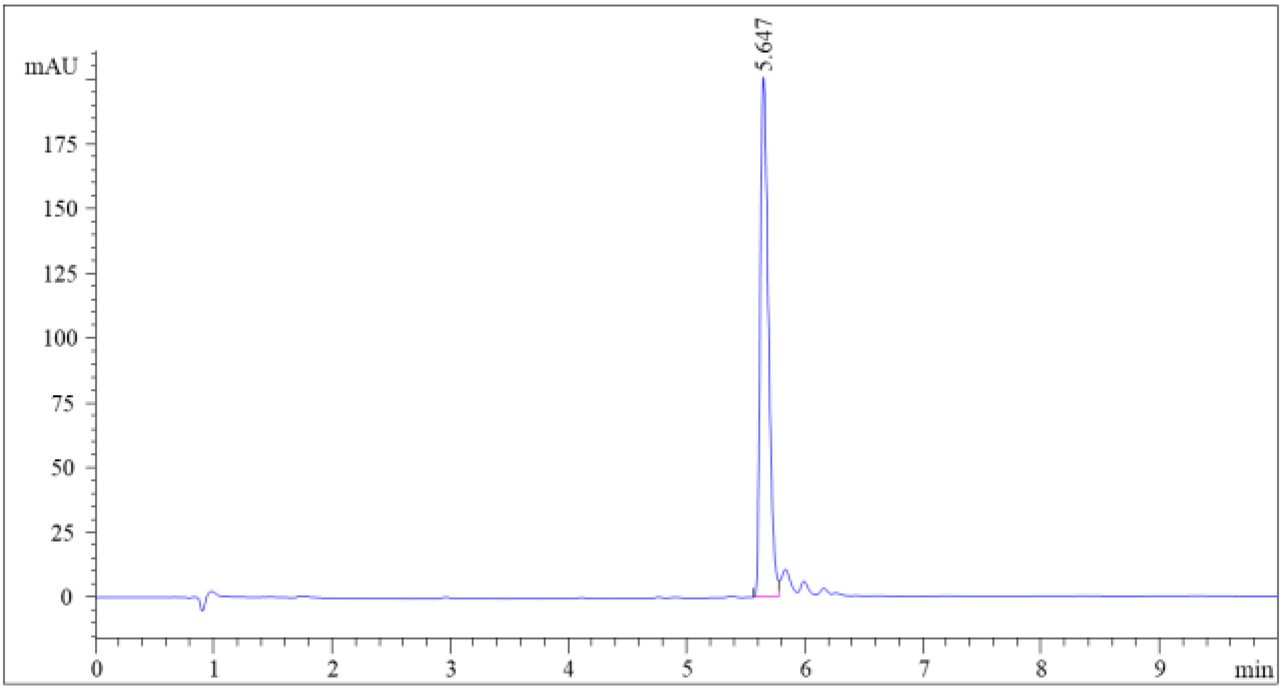


**Figure S33:** Chromatographic profile of compound **26** monitored at λ=320 nm.

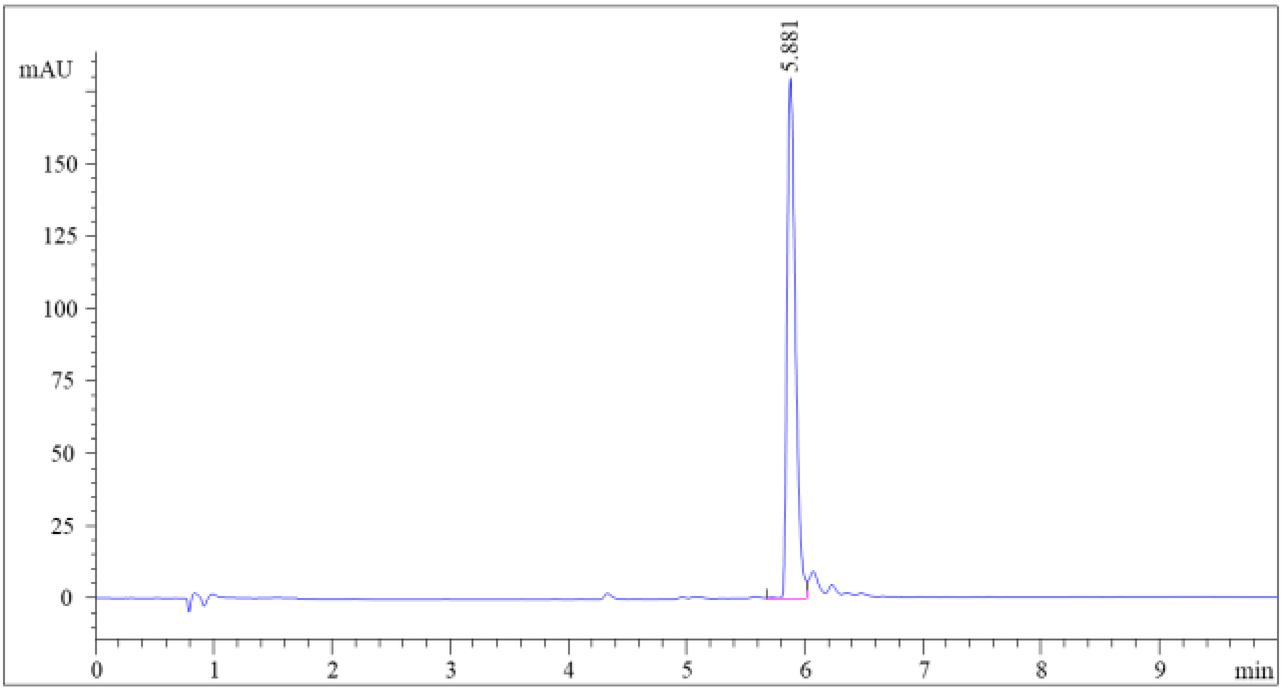


**Figure S34:** Chromatographic profile of compound **27** monitored at λ=320 nm.

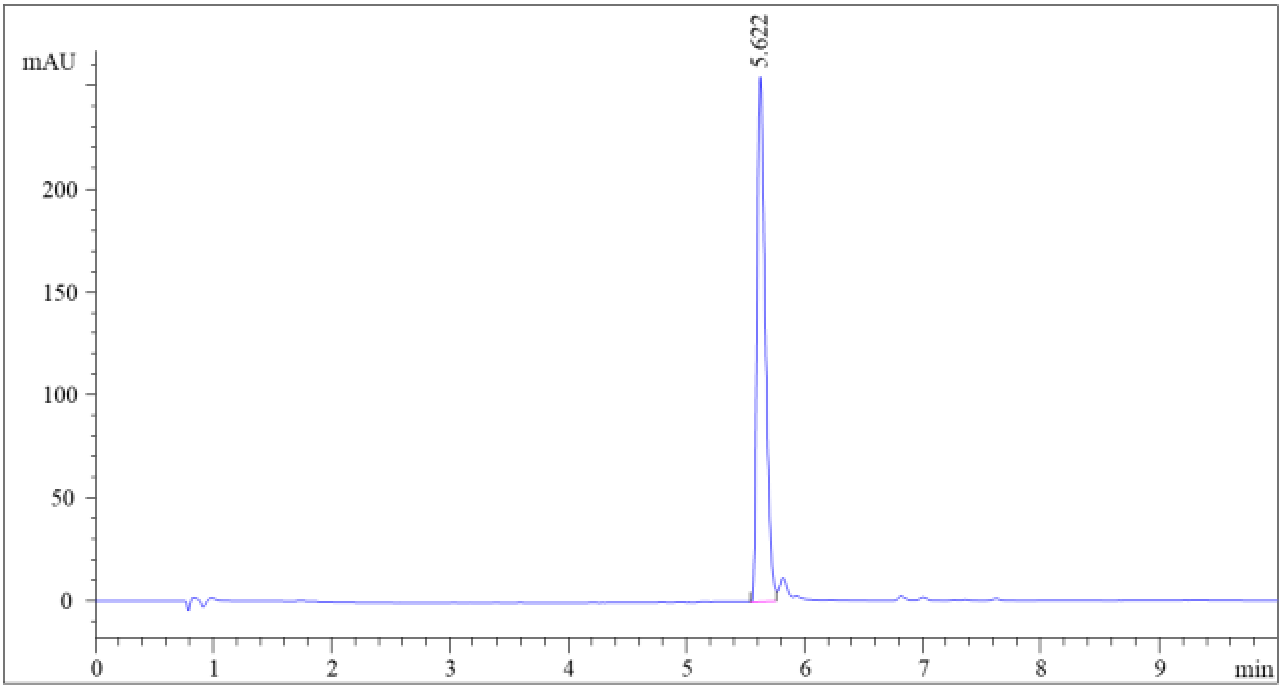


**Figure S35:** Chromatographic profile of compound **28** monitored at λ=320 nm.

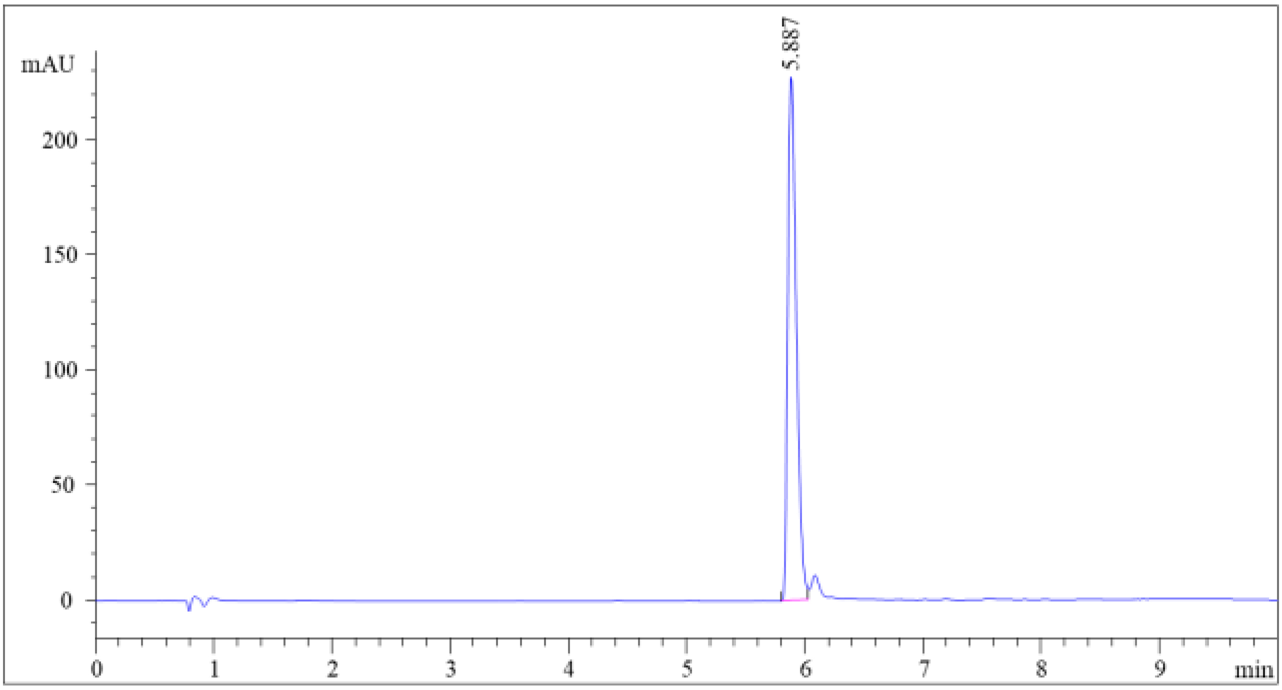


**Figure S36:** Chromatographic profile of compound **29** monitored at λ=320 nm.

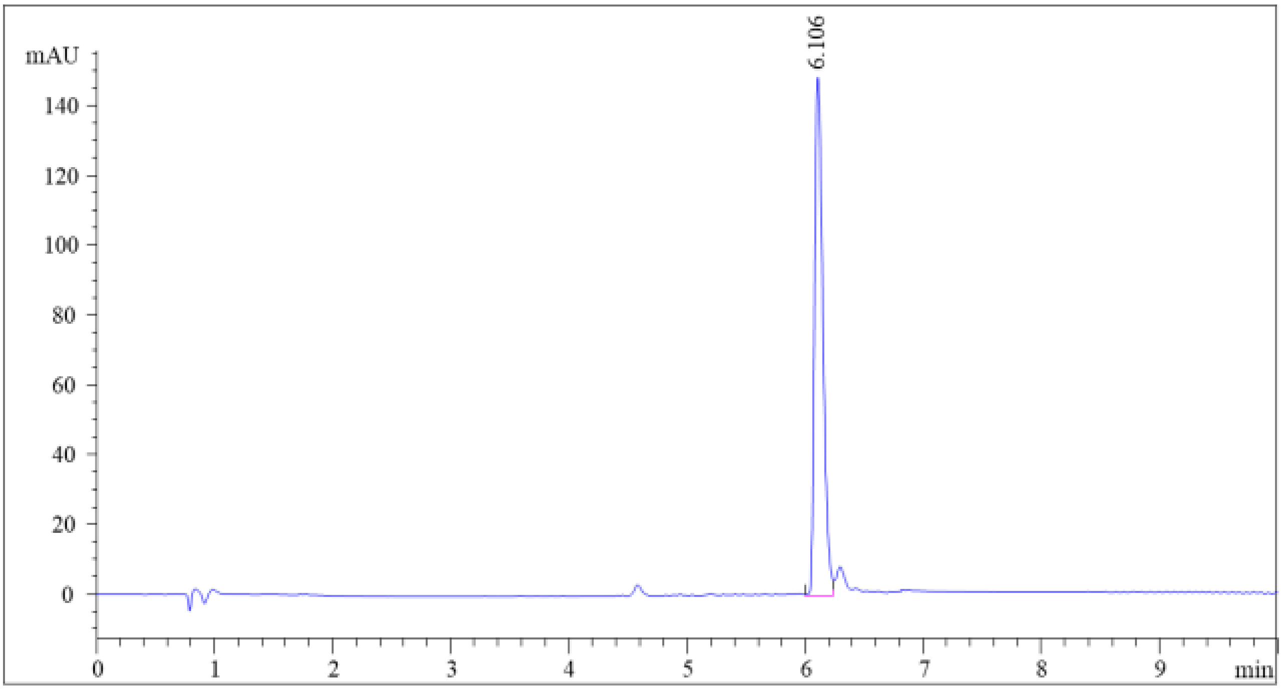


**Figure S37:** Chromatographic profile of compound **30** monitored at λ=320 nm.

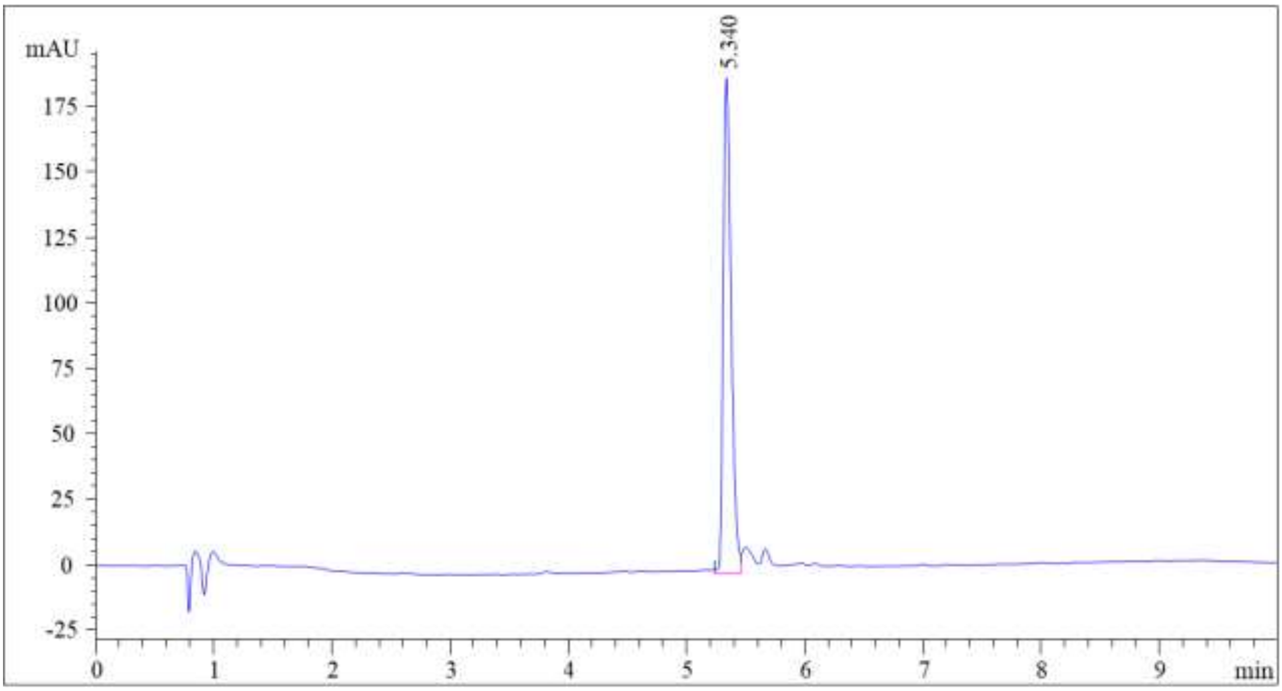


**Figure S38:** Chromatographic profile of compound **31** monitored at λ=270 nm.

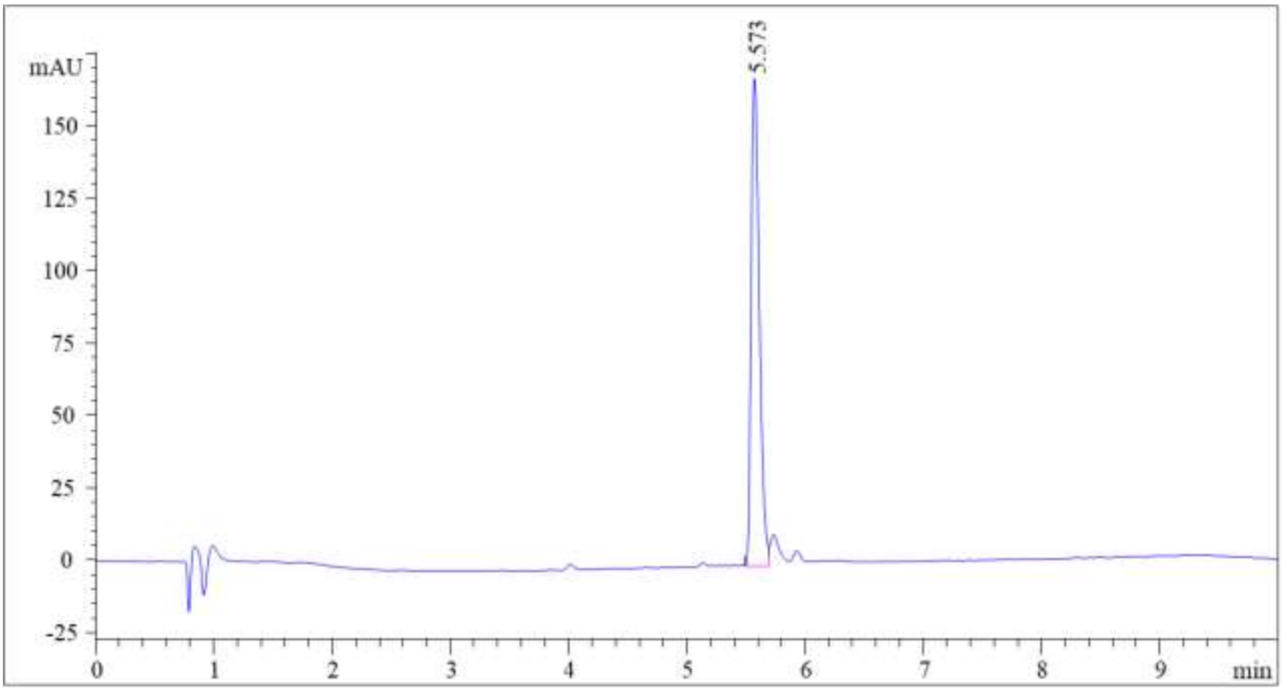


**Figure S39:** Chromatographic profile of compound **32** monitored at λ=270 nm.

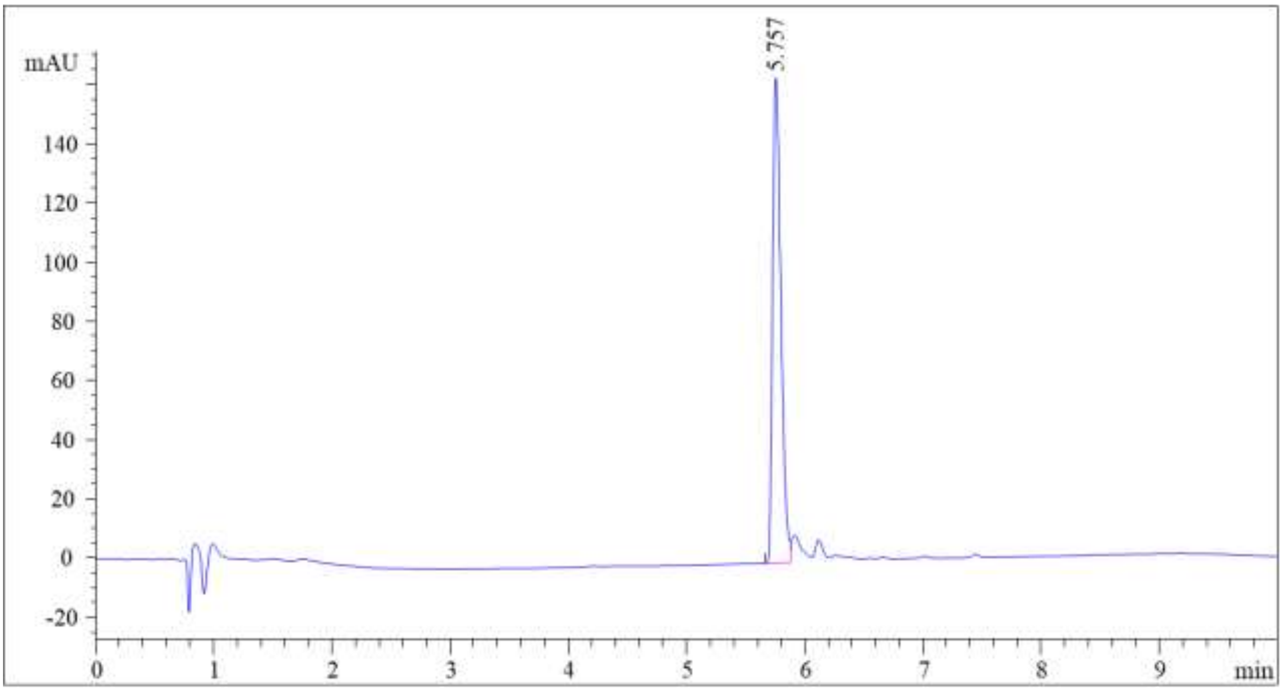


**Figure S40:** Chromatographic profile of compound **33** monitored at λ=270 nm.

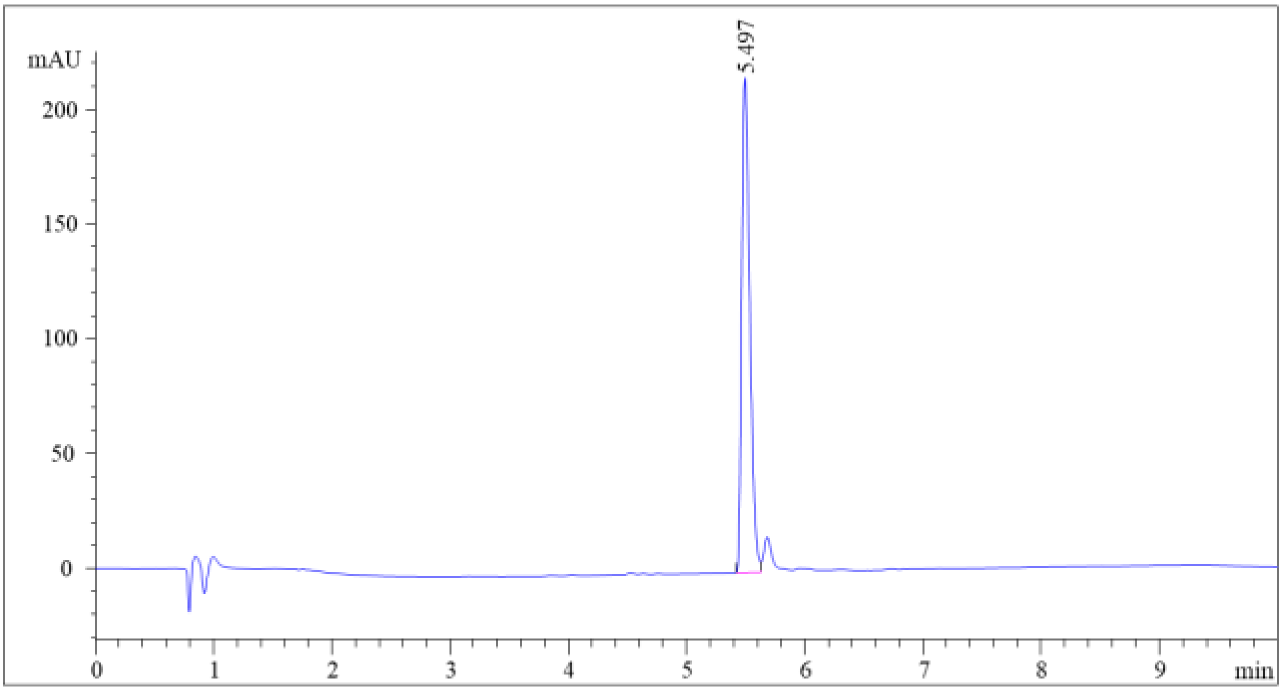


**Figure S41:** Chromatographic profile of compound **34** monitored at λ=270 nm.

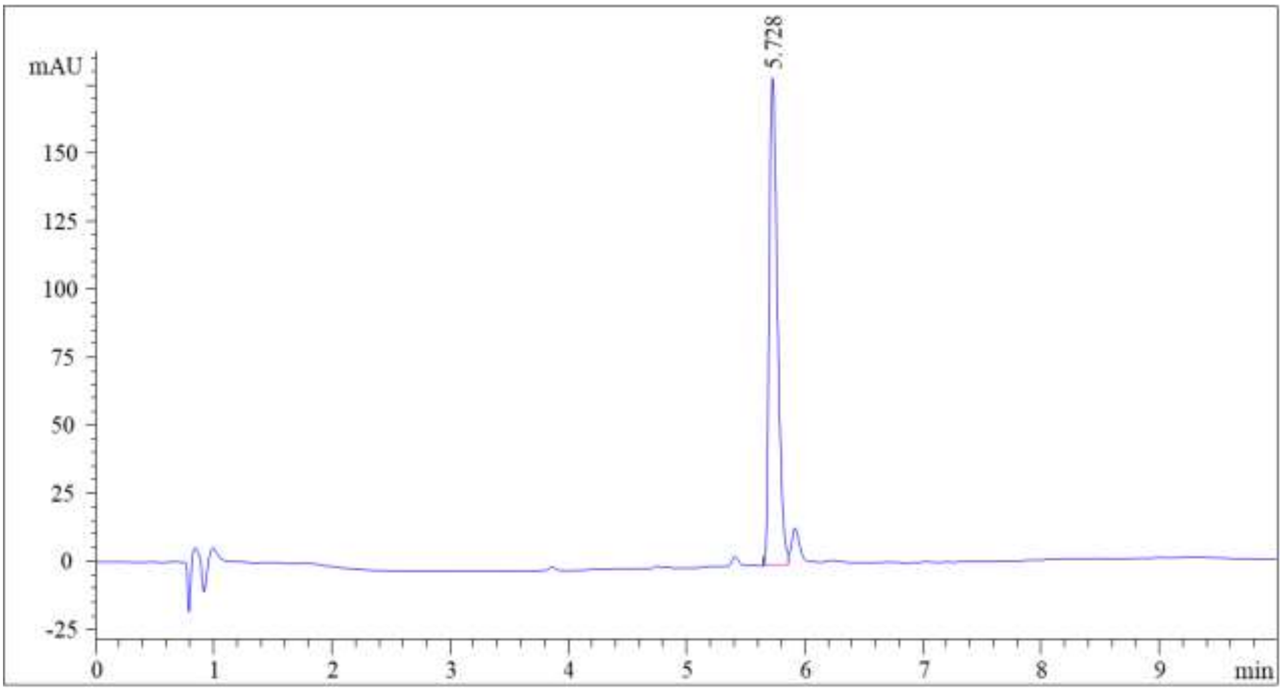


**Figure S42:** Chromatographic profile of compound **35** monitored at λ=270 nm.

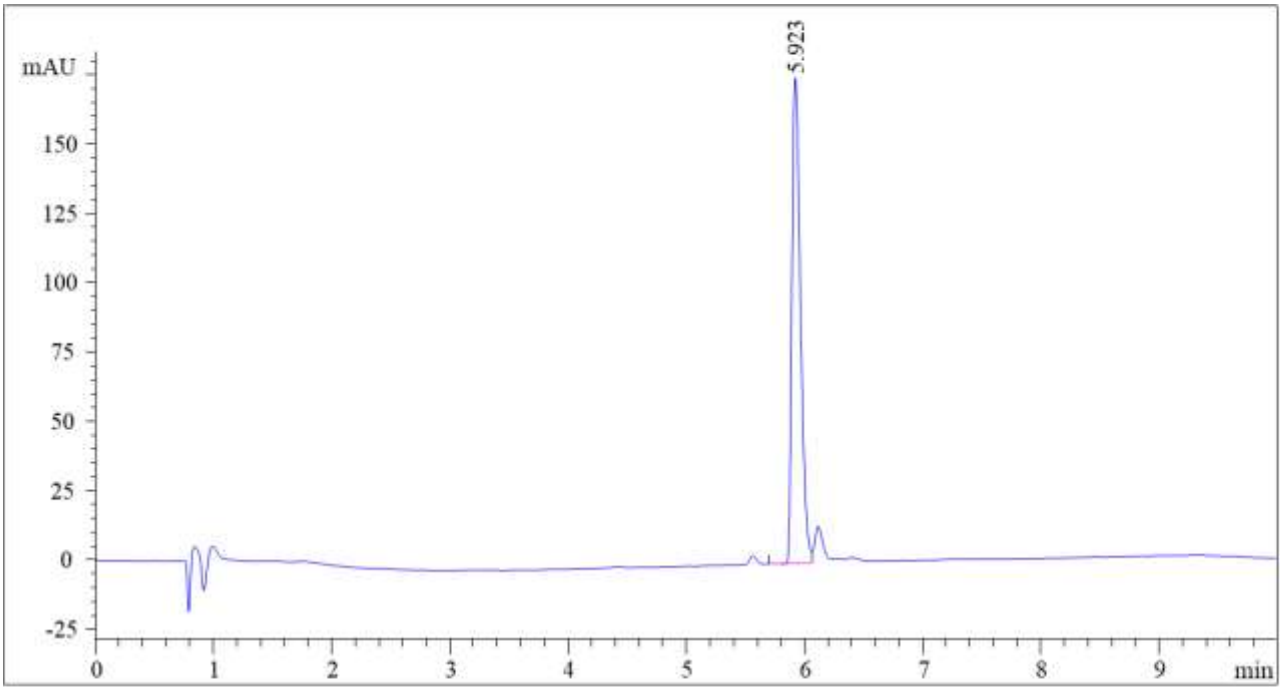


**Figure S43:** Chromatographic profile of compound **36** monitored at λ=270 nm.

UV spectra of compounds **1-36** were reported in Figures S44-S47.

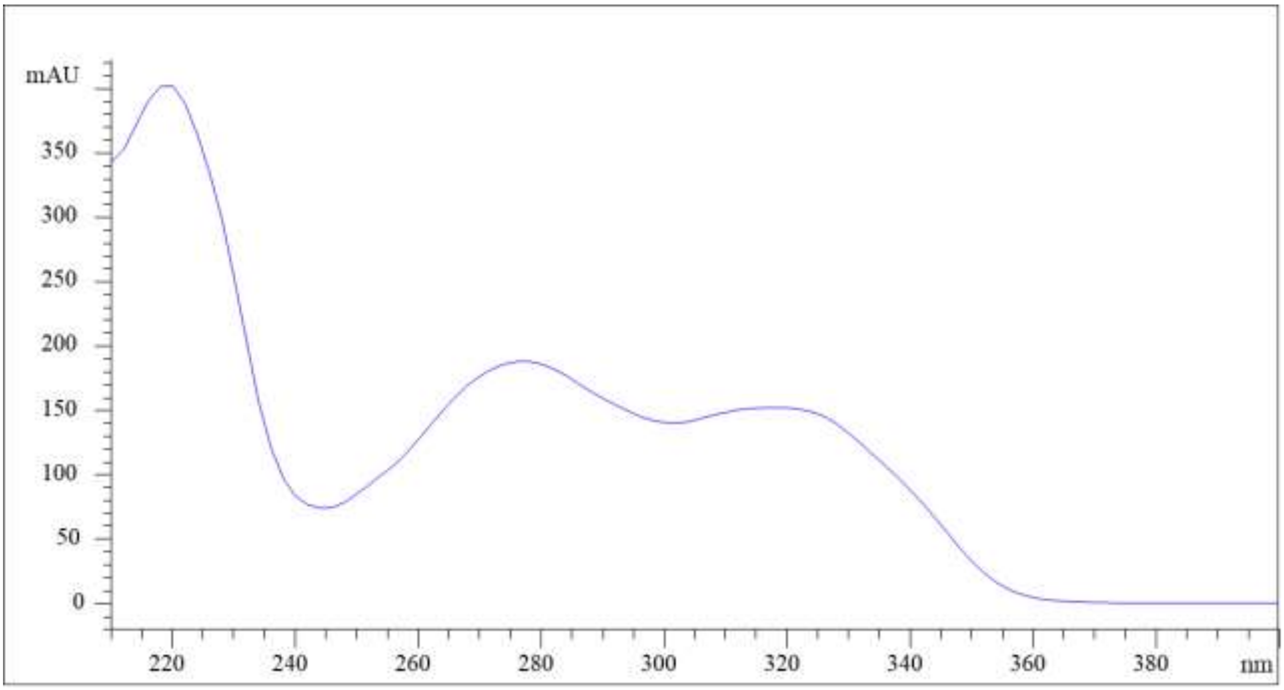


**Figure S44:** UV spectrum of compounds **1-6**.

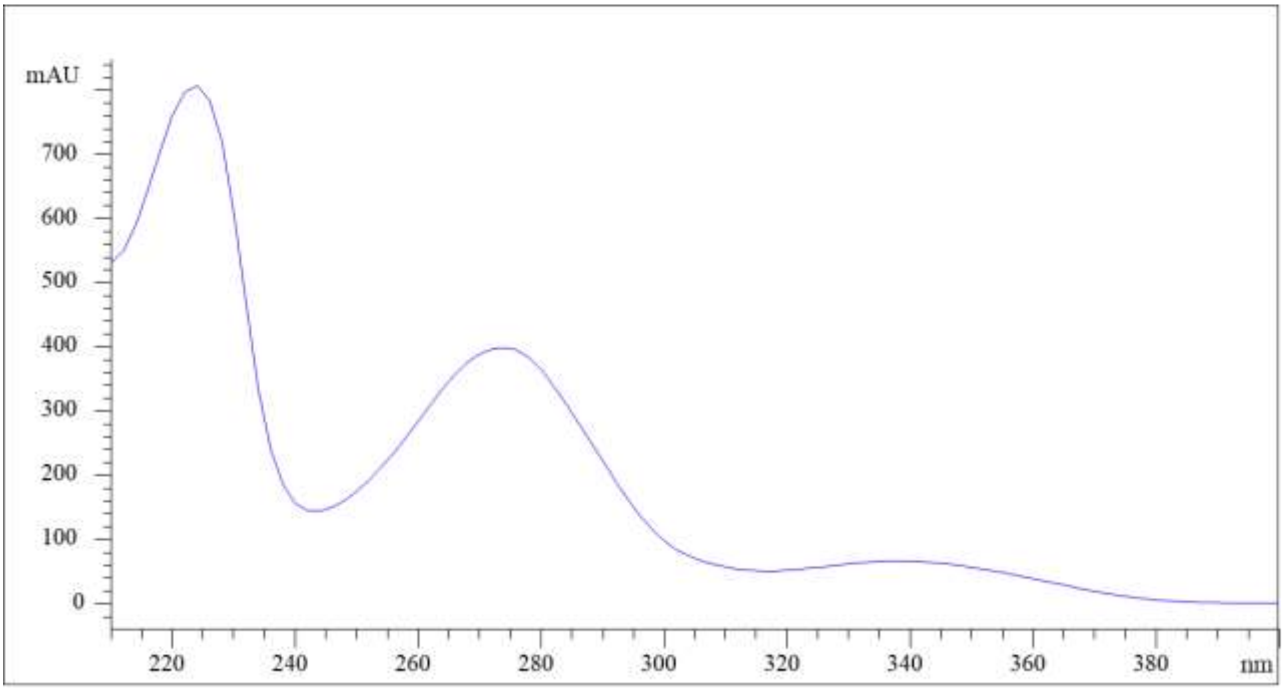


**Figure S45:** UV spectrum of compounds **7-12**, **19-24** and **31-36**.

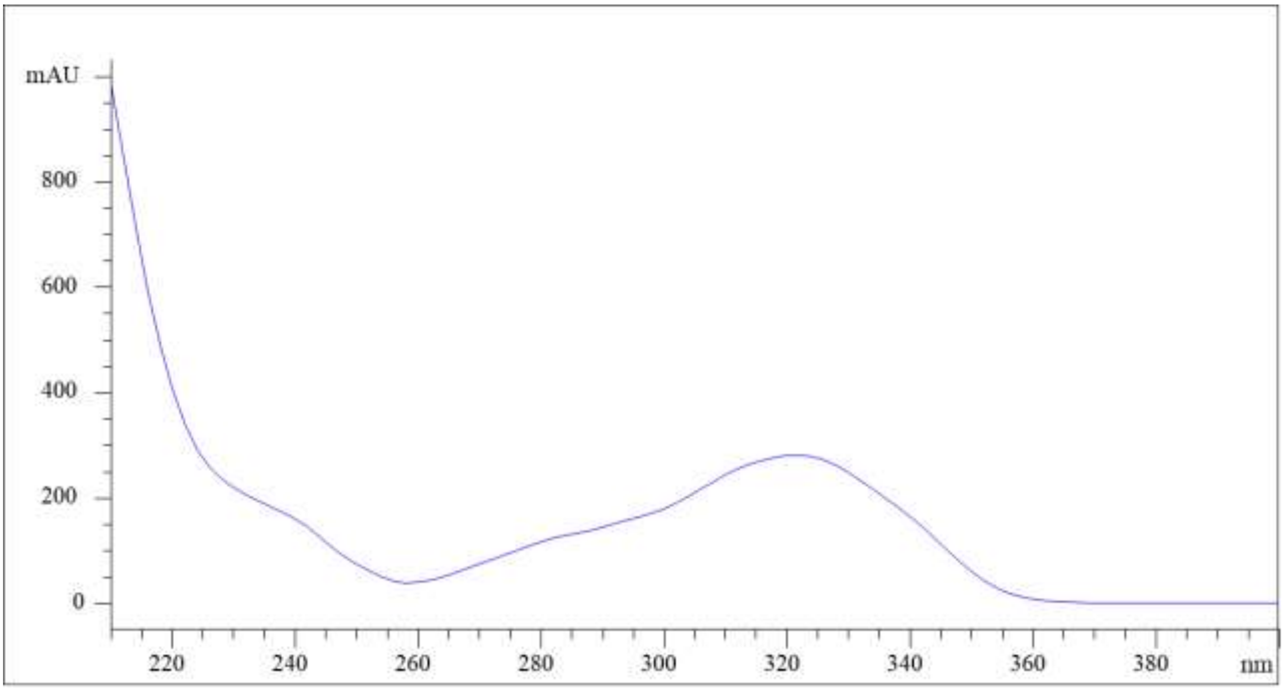


**Figure S46:** UV spectrum of compounds **13-18**.

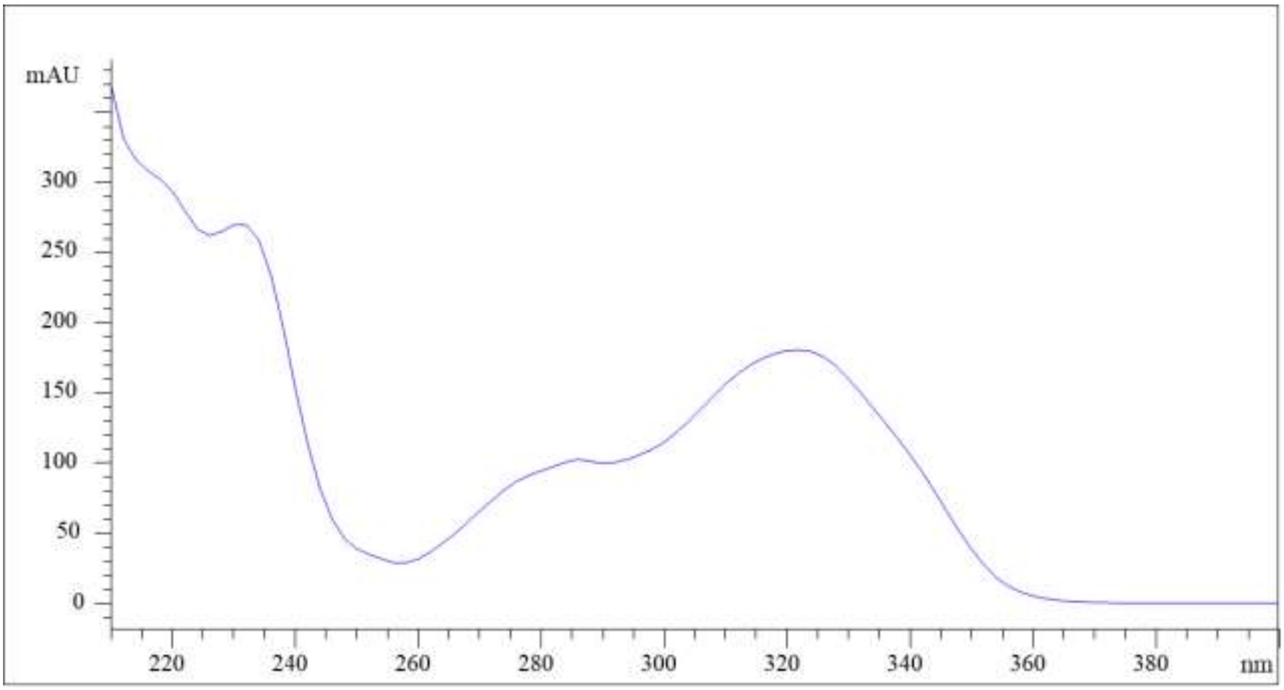


**Figure S47:** UV spectrum of compounds **25-30**.
